# Diversity and prevalence of ANTAR RNAs across actinobacteria

**DOI:** 10.1186/s12866-021-02234-x

**Published:** 2021-05-29

**Authors:** Dolly Mehta, Arati Ramesh

**Affiliations:** 1 National Centre for Biological Sciences, Tata Institute of Fundamental Research, GKVK Campus, Bellary Road, Bangalore, 560065 India; 2 SASTRA University, Tirumalaisamudram, Thanjavur, 613401 India

**Keywords:** ANTAR protein, RNA regulatory system, Structured RNA, Actinobacteria

## Abstract

**Background:**

Computational approaches are often used to predict regulatory RNAs in bacteria, but their success is limited to RNAs that are highly conserved across phyla, in sequence and structure. The ANTAR regulatory system consists of a family of RNAs (the ANTAR-target RNAs) that selectively recruit ANTAR proteins. This protein-RNA complex together regulates genes at the level of translation or transcriptional elongation. Despite the widespread distribution of ANTAR proteins in bacteria, their target RNAs haven’t been identified in certain bacterial phyla such as actinobacteria.

**Results:**

Here, by using a computational search model that is tuned to actinobacterial genomes, we comprehensively identify ANTAR-target RNAs in actinobacteria. These RNA motifs lie in select transcripts, often overlapping with the ribosome binding site or start codon, to regulate translation. Transcripts harboring ANTAR-target RNAs majorly encode proteins involved in the transport and metabolism of cellular metabolites like sugars, amino acids and ions; or encode transcription factors that in turn regulate diverse genes.

**Conclusion:**

In this report, we substantially diversify and expand the family of ANTAR RNAs across bacteria. These findings now provide a starting point to investigate the actinobacterial processes that are regulated by ANTAR.

**Supplementary Information:**

The online version contains supplementary material available at 10.1186/s12866-021-02234-x.

## Background

Actinobacteria is a ubiquitous bacterial phylum, widely distributed across terrestrial and aquatic ecosystems [1]. The phylum consists of very diverse bacteria, ranging from defensive mutualists dwelling in varied habitats to gastrointestinal commensals that provide beneficial properties to their host. They are also the largest source of novel natural antibiotics, enzymes and secondary metabolites. In addition to their immense environmental and industrial impact, this phylum also consists of pathogens such as species from *Corynebacterium*, *Nocardia*, *Mycobacterium* and *Rhodococcus*, which cause disease in humans, animals and plants [2].

The diversity of environmental niches seen within the actinobacteria phylum argues for diverse mechanisms of gene regulation that would allow an efficient response to environmental changes. While a body of literature now places non-coding RNAs and RNA-protein based mechanisms as a major mode of gene-regulation in several model bacteria (reviewed in [3–9]), our knowledge of RNA-based regulatory mechanisms in actinobacteria remains limited ([10–14], and reviewed in [15, 16]).

One approach to identifying regulatory RNAs in actinobacteria, has been using deep sequencing of the transcriptome coupled with 5′-RACE mapping, to identify potential RNAs that map to the untranslated regions (UTRs). These RNAs are then subjected to structure prediction tools [17–19] and compared against known RNA families to confirm the presence of regulatory RNAs. This approach in *Corynebacterium and Streptomyces* under exponential growth conditions has led to the identification of new regulatory RNAs such as the 6C RNA family, 6S RNA family, T-box leader element, novel sRNAs and trans-encoded RNAs [11, 20]. In addition this approach has helped to identify several known metabolite-responsive RNAs such as Mn^2+^ sensing riboswitches (yybP-ykoY), thiamine pyrophosphate (TPP) riboswitches, flavin mononucleotide (FMN) riboswitches, S-adenosyl methionine (SAM)-dependent riboswitches and cobalamin riboswitches (binds to adenosylcobalamin) [11, 20]. A similar approach led to the discovery of 75 novel small RNAs in *Rhodococcus sp.* when grown in glucose and pyrene as sole carbon sources, a small fraction of which have now been assigned functions [12]. Such an approach requires cells to be grown under specific conditions of interest, and do not identify the repertoire of RNAs that the cell can produce in response to unknown signals and cues.

Computational methods have also been successfully employed, to identify regulatory RNAs in actinobacteria. In one study, homologs of genes were first identified and their upstream intergenic regions were aligned and searched for patterns/ motifs using RNA secondary structure prediction tools such as RNA-pattern [18] and PAT (A.V.Seliverstov, unpublished). This led to the identification of LEU element [10], T-box [10] and B12 [21] riboswitches in several actinobacteria. More generally, the RNA family database (Rfam) employs covariance analysis, wherein bacterial genome sequences are scanned for conserved base-pairing patterns, to identify structurally conserved RNA families in the genome. Based on this, the Rfam database suggests the presence of ~ 90 cis-regulatory RNA families in one or more actinobacteria (Rfam v14.2). While these approaches have identified RNAs in actinobacteria, they are mostly limited to RNA families that are highly conserved in sequence and structure, where homologs from different bacterial phyla closely resemble each other.

For some RNA families, the highly GC rich actinobacterial genomes may result in RNA sequences that are diverged from their firmicute or proteobacterial homologs, and hence not easily identified through routine sequence based or structure based searches. One such example is the 6S RNA family, which could only be identified in actinobacteria using a clustering method wherein the sub-optimal RNA structures were used to find functionally relevant motifs [13]. Known 6S RNAs from related bacterial species of proteobacteria, firmicutes and cyanobacteria were analyzed for similarity based on sequence and minimum free energy (MFE) structures. Despite a common function, these RNAs lack sequence and structure similarity. Instead of MFE structure when sub-optimal structures were analyzed, these RNAs fell into different clusters, 3 of which represented most of the 6S RNAs. Information from these 3 clusters was used to identify 6S RNAs across genomes. Through this clustering method, several 6S RNAs were obtained in *Mycobacteria* and *Streptomyces* species, representative of actinobacteria.

We observed a similar discrepancy in an important family of RNAs known to be targets of the ANTAR RNA-binding protein. RNAs bound by ANTAR proteins are conserved in structure and are widespread among firmicutes and proteobacteria [22–27]. In actinobacteria, however, despite the widespread presence of ANTAR protein domains (Pfam: PF03861), their target RNAs remained unidentified. Only recently, in a study focusing on *Mycobacteria*, these RNAs were identified using a genome-wide covariance search approach combined with clustering [28]. A search model (structure based sequence alignment) enriched in firmicute and proteobacterial RNAs showed very high sequence and structure similarity and as a consequence failed to predict RNAs in actinobacteria. When diverse RNAs from different firmicutes and proteobacteria were added to the search model, they separated into several clusters based on sequence and structure similarity. This clustering resulted in a search model that successfully identified RNAs in *Mycobacteria* by removing the bias imposed by highly similar or highly dissimilar RNAs. Notably, neither the firmicute [27] nor the mycobacterial search models [28] were effective in finding ANTAR RNAs across the actinobacterial phylum.

Here, we identify the repertoire of ANTAR-target RNAs across actinobacteria. To identify these RNAs we first developed an actinobacteria-centric search model which when used to search against all actinobacterial genomes, successfully identified ANTAR-target RNAs. We find that the family of ANTAR-target RNAs is present across all actinobacteria and co-occurs with ANTAR proteins. There are only a few examples of bacteria where despite the presence of ANTAR proteins, we are unable to identify RNA targets. These RNAs resemble ‘cis’ regulatory RNAs in their genomic locations, typically residing in the untranslated region (UTR) or near the start of a coding region. COG (Cluster of Orthologous Genes) database is a tool to functionally annotate protein sequences based on homology to known protein sequences. COG analysis of the genes distal to ANTAR-target RNAs reveals that these RNAs are associated with transport and metabolism of small molecule metabolites, ranging from amino acids to metal ions to diverse sugar substrates. Additionally, ANTAR-target RNAs also appear linked to genes encoding transcription factors that are known to modulate the expression of several transporters. Our study underlines the presence of the ANTAR protein-RNA regulatory system in actinobacteria, and its importance in governing the uptake and metabolism of a variety of nutrients. This approach of scanning an existing RNA family for sequence diversity and using that to find homologs in distant phyla may be broadly applicable to other RNA families.

## Results

### Identifying ANTAR-target RNAs across phylum actinobacteria

**Fig. 1:**
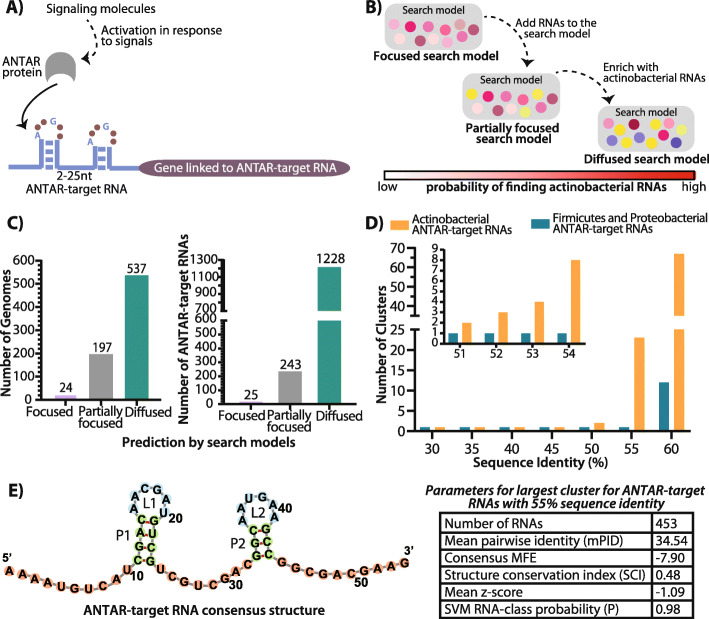
Improvised search model to predict ANTAR-target RNAs in actinobacteria. **A** Cartoon showing the ANTAR protein-RNA regulatory system. Specific signals activate the ANTAR protein (grey), which upon activation binds the dual stem loop ANTAR-target RNA (blue). This results in regulation of the downstream gene (gene linked to ANTAR-target RNA, shown in purple). **B** Schematic shows the steps performed to identify ANTAR-target RNAs using a covariance-based computational search. Previously reported search models with too little diversity (focused) did not yield any results in actinobacteria, while a search model with only moderate diversity (partially focused) identified ~ 243 RNAs in actinobacteria, with a bit score threshold≥14. 30 actinobacterial representative RNAs from this set were used to enrich the search model further and this actionbacteria centric search model (diffused search model) resulted in a comprehensive list of ANTAR-target RNAs in actinobacteria. The probability of finding RNAs in actinobacteria is represented as a bar (red indicates high probability). **C** Bar plot (left) shows the total number of actinobacterial genomes where RNAs are predicted using three different search models (purple, gray and green). Bar plot (right) shows the total number of RNAs predicted using three different search models. The diffused search model is able to predict RNAs in more than 60% of actinobacterial genomes as compared to the focused and partially focused search model. **D** RNA sets from firmicutes/ proteobacteria and actinobacteria were clustered using cmbuild. Bar plot shows the number of clusters obtained with varying sequence identity cut-offs imposed using cmbuild. Clusters obtained using 51–54% sequence identity cut-off are shown as an inset. **E** Consensus structure obtained for the actinobacterial ANTAR-target RNA sequences from the largest cluster with 55% sequence identity is visualized using Forna (Left). Stems (green) while the internal loops (blue) and the unpaired nucleotides (pink) are shown. Parameters obtained from RNAz for the largest cluster with 55% sequence identity are shown (Right)

Analysis of the previously reported ANTAR RNAs revealed that ~ 400 ANTAR-target RNAs are known in firmicutes and proteobacteria [27], and they are conserved in secondary structure with dual stem loops separated by a linker (Fig. 1A). Each stem possesses a hexanucleotide loop where the first and fourth positions are conserved in sequence as an adenine (A1) and guanine (G4) respectively (Fig. 1A). More recently, in a study focusing on ANTAR RNAs in *Mycobacteria*, a covariance-based computational approach was used to search for ANTAR RNAs. Here it was shown that a focused search model (a set of RNAs aligned based on similar secondary structure and sequence) consisting of highly similar firmicute/proteobacterial RNAs was unable to predict RNAs in *Mycobacteria*. This is likely due to a divergence of mycobacterial ANTAR RNAs from their firmicute/proteobacterial homologs. Only when the search model was modified to include more diversity that expands the sequence space (partially focused search model), was the search capable of finding RNAs in *Mycobacteria*. This resulted in ~ 90 ANTAR-target RNAs identified across all mycobacterial species [28].

To further identify ANTAR-target RNAs in actinobacteria, we used the partially focused search model developed in the mycobacterial study (Fig. 1B) and performed a covariance-based RNA search against all sequenced (~ 720) actinobacterial genomes. This search could identify ~ 243 ANTAR-target RNAs with high confidence. However, these newly found RNAs were restricted to less than 30% (197 of 720) sequenced actinobacterial genomes (Fig. 1B-C).

In order to improve the search and predict RNAs more comprehensively across actinobacteria, we picked 30 representative RNAs from the initial 243 hits and created a new and fully actinobacterial search model (Additional file 1: Table S1). This model was then used as input in a covariance search against the 720 genomes. The RNA hits obtained from this search were filtered through a bit score ≥ 15. The bit score reports on the similarity of each RNA hit to the consensus derived from the search model as compared to a null model of non-homologous sequences. We have also manually examined each individual RNA hit to ensure that the reported RNAs do possess the known ANTAR target RNA features. This includes the dual stem-loop structured motif with hexanucleotide loops and a linker region that lies between 2 to 25 nucleotides. After manual curation, ~ 1228 RNAs were predicted with high confidence (Additional file 1: Table S2, S3), and importantly- RNAs were found in nearly 74.5% of sequenced actinobacteria (Fig. 1B-C). Additionally, we took the 30 actinobacterial RNA sequences from the diffused search model and shuffled each sequence to result in 15,000 new sequences (500 sequences from each RNA). Shuffling was performed using *fasta-shuffle-letters* from the MEME-suite with all positions shuffled or maintaining the dinucleotide frequencies (https://meme-suite.org/meme/doc/fasta-shuffle-letters.html). These shuffled sequences serve as negative control data sets (Additional file 1: Fig. S1A). Notably, our search identified only two RNA hits with a bit score ≥ 15 from these sets suggesting that our false positive rates are extremely low. Comparing the performance statistics of the searches from the 3 search models (focused/ partially focused/ diffused) reveals a significant advantage gained by the diffused model over the other two models (Fig. 1C, Additional file 1: Fig. S1B). Hence the RNAs identified through the diffused search model were considered for further analyses.

Removal of identical RNAs from different strains of a species resulted in ~ 611 unique ANTAR-target RNAs. Moreover, the 243 RNAs predicted initially, were also recovered in this search. This includes ANTAR-target RNAs predicted in mycobacterial species, which have been experimentally validated as binders of ANTAR proteins [28].

We additionally analyzed this search model using the cmbuild program [29], which creates a statistical profile of alignments and thus reports on the extent of sequence conservation and base-pairing potential (co-variation) within the aligned RNAs. Based on sequence (42% sequence identity) and structure (Covariance Model, CM score = 0.48), the actinobacterial seed alignment shows significantly higher variation than the partially focused firmicute/proteobacteria seed (51% sequence identity and a CM score of 0.61). These results indicate that an actinobacteria-enriched search model that allows higher sequence/structural diversity while maintaining the core defining features of the RNA family is ideal for identifying new RNAs in actinobacteria.

In order to understand the characteristics of ANTAR-target RNAs in actinobacteria, we compared the 611 predicted actinobacterial RNAs with the previously reported 306 ANTAR-target RNAs from firmicutes and proteobacteria [27]. Using cmbuild the RNAs from each set (actinobacterial versus firmicute-proteobacteria) were clustered at increasing sequence identity thresholds (Fig. 1D). We find a stark difference between the two sets of RNAs. The actinobacterial RNAs start to separate out as clusters at a much lower sequence identity threshold (50%) when compared to firmicutes and proteobacteria (55%). This shows inherent diversity within the actinobacterial RNAs, possessing less than 50% sequence identity. We further analyzed the largest cluster of RNAs from each set for the extent of structural conservation. Even here, RNAs that are similar in sequence and hence clustered together showed a low CM (Covariance Model) score of ~ 0.44 when compared to the firmicutes and proteobacterial set (CM score: ~ 0.60). This confirms that actinobacterial RNAs allow for significantly higher sequence and structure variations (Additional file 1: Fig. S1C).

Next we subjected all the RNA hits to analysis using RNAz [17, 30] which computes a consensus secondary structure. We find that these RNAs, as expected fold into a dual stem-loop motif maintaining the core ANTAR-target RNA structural features. The consensus secondary structure for these RNAs shows more than 50% conservation of adenine and guanine in loop positions 1 and 4 respectively, and ~ 50% conservation within the stems (Additional file 1: Fig. S2A). The largest cluster of RNAs (~ 75% of all the predicted RNAs) shows a minimum free energy of − 7.90 and a structure conservation index (SCI) of 0.48 (Fig. 1E). The mean z-score of − 1.09 obtained for these RNAs indicates that the structure motif observed is a stable true motif and does not occur by chance. The test for functionality based on SCI and z-score indicates that these RNAs belong to ‘functional RNA’ class (*P* > 0.5). A similar RNAz analysis for all actinobacterial RNA hits is summarized in Additional file 1: Fig. S2B.

### Distribution of ANTAR proteins and target-RNAs in actinobacteria

**Fig. 2:**
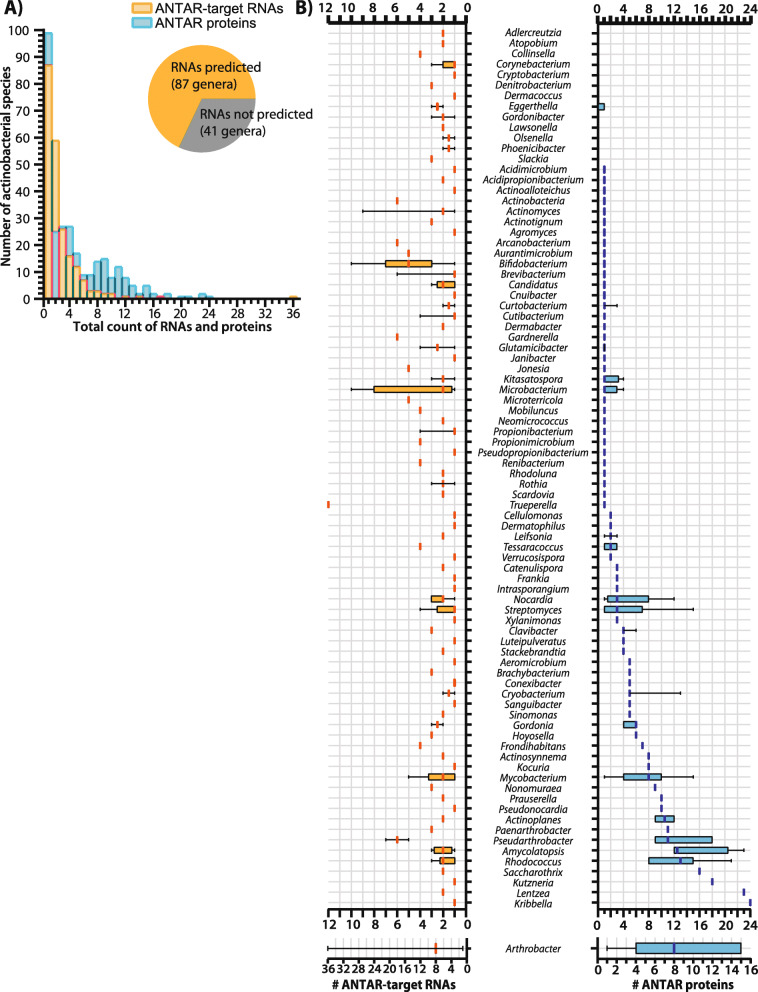
Distribution of ANTAR-target RNAs identified in Actinobacteria. **a** Distribution of ANTAR-target RNAs (yellow) or ANTAR proteins (blue) in actinobacterial species. Inset pie-chart shows the number of actinobacterial genera where ANTAR-target RNAs are predicted. **b** Distribution of ANTAR-target RNAs (left) and ANTAR proteins (right) in actinobacterial genera are shown as box-whisker plots. Median (vertical line), interquartile range (box) and 1.5 times the inter-quartile range (whiskers) are shown

With a comprehensive list of ~ 611 ANTAR-target RNAs identified, we looked at their distribution in the 128 known genera of actinobacteria and found that RNAs were predicted in genomes representing 87 genera which include 219 species (Fig. 2A, inset). The majority of actinobacterial species possess 1 to 3 RNAs per genome (Fig. 2A), while some species of *Actinomyces, Microbacterium, Bifidobacterium, Trupurella and Arthrobacter* appear to possess nearly 10 or even up to 36 different RNAs in the same genome (Fig. 2A-B).

The ANTAR domain is an RNA-binding domain and proteins containing this domain are known to selectively recognize and bind RNAs of this family. Hence we asked if the distribution of RNAs reflected the distribution of the ANTAR proteins. To this end, we performed an HMMsearch using the ANTAR domain HMM model from the protein family database (Pfam: PF03861). With an e-value threshold set to 1e-4, we identified ~ 1459 ANTAR-domain containing proteins in 245 species of actinobacteria. As seen for the RNAs, the distribution of ANTAR proteins too shows high variation, ranging from 1 to greater than 10 ANTAR proteins in a genome (Fig. 2B, Additional file 1: Fig. S3). Interestingly, within the same genome, we do not always see a one to one correlation between the number of RNAs predicted and the number of ANTAR proteins present (Fig. 2B). For example, in *Xylanimonas* there are 3 distinct ANTAR domain proteins with unique domain architectures. However, we predict only one ANTAR target RNA here, suggesting that the same RNA may act as a hub through which many different ANTAR proteins may act, towards different cellular outcomes. In contrast, *Trueperella* appears to possess a single ANTAR domain protein but 12 predicted RNAs, suggesting that many convergent processes may be controlled by ANTAR in *Trueperella*.

We found examples (< 30% of species) where no RNAs were predicted despite the presence of one or more ANTAR proteins in the genome. Similarly, in a few examples no ANTAR proteins are present in a genome even though ANTAR-target RNAs are predicted with high confidence. Whether or not ANTAR proteins and RNAs have an active role in these organisms, or if alternate approaches are required to find RNAs and proteins in these organisms remains to be seen (Fig. 2B, Additional file 1: Fig. S3). Regardless, these analyses imply that within phylum actinobacteria there is diversity of ANTAR function and mechanism. While the presence of RNAs is not sufficient to indicate active association with the ANTAR protein, we note examples from previous studies where an RNA-binding activity for actinobacterial ANTAR proteins has been reported [28, 31].

### ANTAR-target RNAs are located in untranslated and coding regions of mRNAs

Previous studies have shown that ANTAR proteins, upon activation (through phosphorylation) bind to their target-RNAs and regulate downstream gene expression in *cis* [22, 24, 27, 28, 31–33]. Hence we analyzed the genomic locations and contexts of the predicted RNAs.

**Fig. 3:**
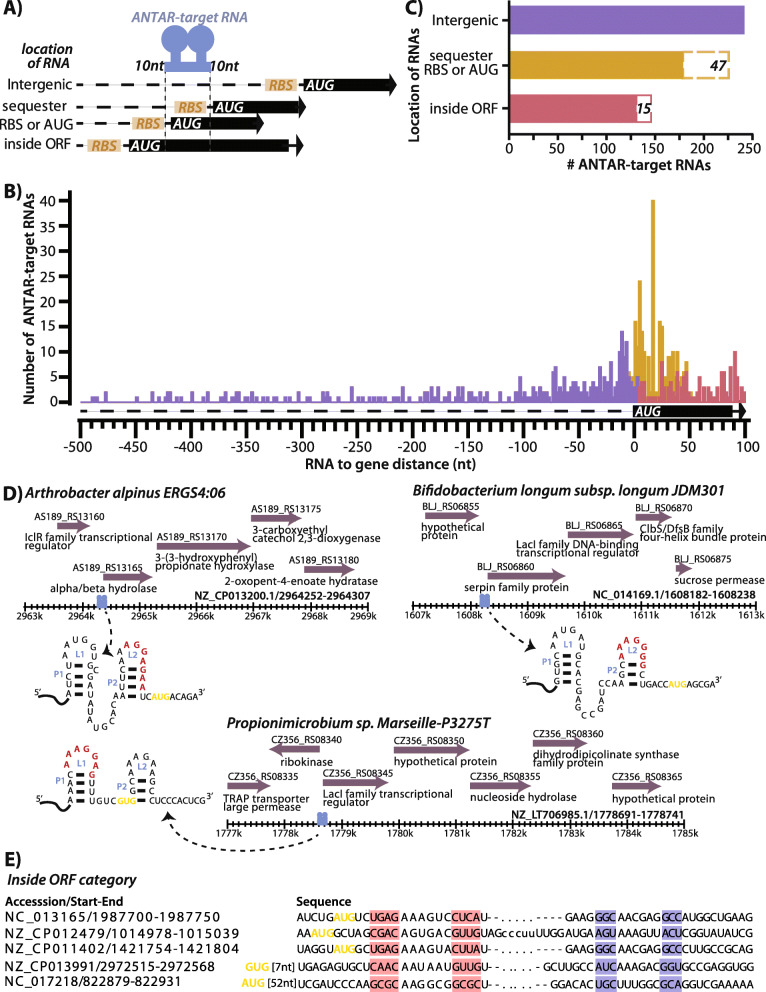
Locations of ANTAR-target RNAs within their genomic context. **a** Schematic shows the location of ANTAR-target RNAs. RNAs are grouped in three categories- intergenic’ for RNAs that lie at a distance> 15 nt from start of ORF, ‘sequester RBS or AUG’ for RNAs which overlap with the ribosome binding site or start codon and ‘inside ORF’ for RNAs which lie after the start codon. 10-nucleotide flanking regions on either side of the dual stem loop structure are included in the distance calculations. **b** Histogram shows distribution of RNAs versus their distance from the respective ORF. Several RNAs are found near the ORF start site, sequestering either RBS or AUG (yellow). **c** Plot shows total number of predicted RNAs in three categories as described in panel A. 47 RNAs (dashed brown box) in the ‘sequester RBS or AUG’ category and 15 RNAs (dashed red box) in the ‘inside ORF category were assigned based on alternate ORF predictions. **d** Representative RNAs from ‘sequester RBS or AUG’ category are shown with the ANTAR-target RNA structure marked. Potential RBS (red) and start codon (yellow) are shown. Genomic context of these RNAs (blue) are shown with ORFs (purple) with their NCBI gene annotations. **E** Representative RNAs from the ‘inside ORF’ category are shown. The dual stems of the ANTAR-target RNA are highlighted in pink and blue. Start codon is marked in yellow

Based on genomic location, RNAs were categorized as: 1) intergenic (RNA lies 15 nt–500 nt upstream to an ORF), 2) sequester RBS or AUG (RNA harbors the ribosome-binding site (RBS) or the start codon or 3) inside ORF (RNA resides after the ORF start-site and lies within ≤100 nt of the ORF start-site) (Fig. 3A-B).

We find that from a total of 611 RNAs analyzed, ~ 39% RNAs are intergenic with a majority lying immediately upstream of an ORF, possibly in the 5’UTR of the corresponding mRNA (Fig. 3C, Additional file 2: Table S4). These RNAs were subjected to rho-independent terminator prediction using TransTermHP v2.08 [34] but only few of the RNAs appear to reside upstream of a terminator, with the second stem loop showing alternate base-pairing with the terminator (Additional file 1: Fig. S4). These few examples are reminiscent of ANTAR-target RNAs in firmicutes and proteobacteria, where binding by the ANTAR protein stabilizes the two-stem loop anti-terminator structure, allowing transcription of the downstream gene. With high GC genomes, it is possible that terminator predictions are inaccurate for these bacteria and hence other approaches may be required to ascertain the mode of transcriptional regulation.

Nearly ~ 37% of the actinobacterial target-RNAs overlap directly with the RBS or start codon (Fig. 3C, Additional file 2: Table S4). In a recent report it was shown that in *M. tuberculosis* and *M. smegmatis,* binding of activated ANTAR protein to such target RNAs, represses translation of the downstream mRNA, possibly by occluding the ribosome from binding the RBS [28]. We see similar features in these RNAs. For example, in *Arthrobacter alpinus* and *Bifidobacterium longum*, the RBS is sequestered within the second stem-loop, whereas in *Propionimicrobium species* both the RBS and the ORF start site lie within the ANTAR-target RNA (Fig. 3D).

The ‘inside ORF’ category consists of ~ 24% ANTAR-target RNAs (Fig. 3C, representatives shown in Fig. 3E). Several studies on non-coding RNAs [35–42] have shown that structured motifs within the mRNA transcript may influence mRNA stability or regulate translation. It is possible that these ANTAR-target RNAs also control gene expression, though the detailed mechanism needs to be uncovered.

### Cellular pathways and genes associated with actinobacterial ANTAR-target RNAs

We next asked what cellular processes are linked to ANTAR in actinobacteria. Studies in *Enterococcus, Pseudomonas, Klebsiella, Acinetobacter* and *Geobacter* reveal that ANTAR-target RNAs are linked to nitrogen utilization [22, 23, 27, 33]. Only few studies in actinobacteria have investigated the role of ANTAR. In *Mycobacteria*, ANTAR mediated gene regulation might influence lipid and related redox processes [28] while a recent study in *Streptomyces*, show that the deletion of ANTAR-protein (*SSDG_04087*) impairs the developmental process and antibiotic production [43].

For this analysis, we considered all 3 categories of RNAs (UTR, sequestering RBS or AUG, inside ORF). Specifically, where the RNA hit resides in the UTR or overlaps with the RBS/AUG, we consider the gene immediately distal to the dual stem loop as the gene linked to ANTAR-target RNAs. For inside ORF category, the gene inside which the RNA lies is considered to be linked to ANTAR. Taking these genes as input, we performed COG analyses using the eggNOGmapper server. eggNOGmapper is a tool that performs a protein sequence homology search against precomputed eggNOG protein database to identify orthologs using a BLAST-like approach, and assigns the COG functional categories, KEGG pathways and gene ontology terms from orthologs to the query [44, 45].

**Fig. 4:**
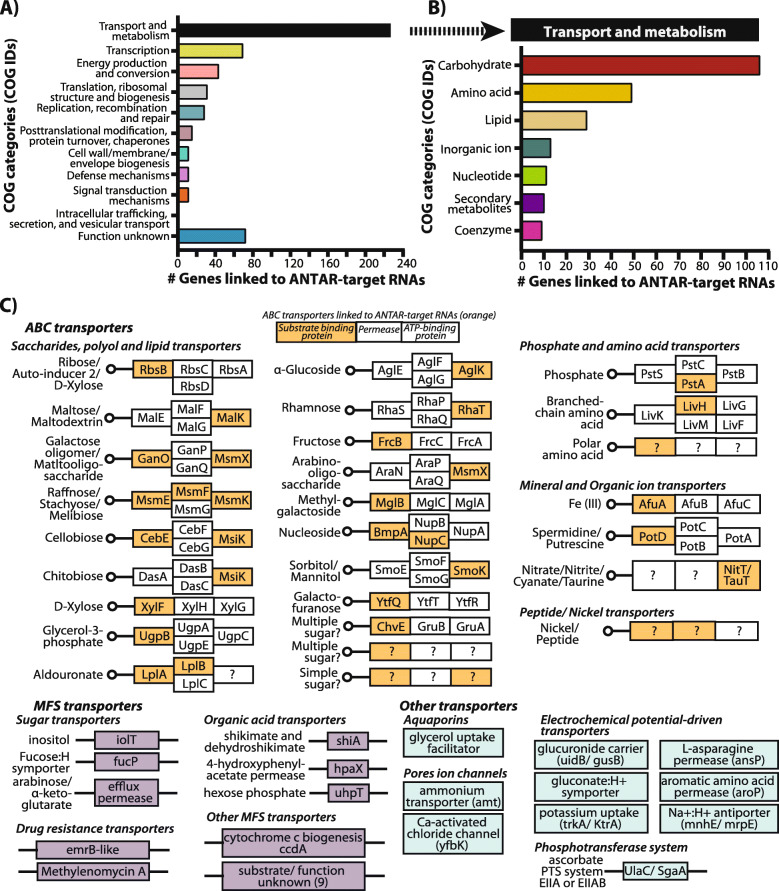
COG analysis of genes linked to ANTAR-target RNAs in actinobacteria. **a** Genes linked to ANTAR-target RNAs, analysed using EggNOG-mapper, get assigned to 11 COG categories. Bar plot shows distribution of genes linked to ANTAR-target RNAs, in each COG category. **b** Bar plot shows distribution of genes linked to ANTAR-target RNAs, within the ‘transport and metabolism’ COG category. Carbohydrate and amino-acid transport and metabolism are the major processes represented by the targets. **c** ABC transporters with the substrate binding protein, membrane bound permease and ATP-binding components (boxes) are shown. Components of the transporter whose functions are not known are marked (?). Transporter components whose transcripts harbor an ANTAR-target RNA are marked in orange. Genes linked to ANTAR-target RNAs, encoding MFS transporters (purple) and other transporters (blue) are shown

Our analysis showed that ~ 85% of genes linked to ANTAR-target RNAs belong to 17 different COG categories, while 15% are genes of yet unknown function (Fig. 4A-B, Additional file 1: Fig. S5A-B, Additional file 2: Table S4). The majority of genes encode proteins involved in transport and metabolism of compounds, with a smaller subset restricted to enzymes involved in energy production. Core cellular processes including transcription, translation, replication and DNA repair also appear to be linked to ANTAR-target RNAs, and make up the next largest categories of COGs (Fig. 4A, Additional file 1: Fig. S5A). Additionally, we find a diversity of metabolites whose transport and metabolism would be linked to ANTAR (Fig. 4B, Additional file 1: Fig. S5B), with carbohydrate, amino-acid and lipids standing out as preferred metabolites.

We next asked if a cellular process or function linked to ANTAR was restricted to any particular branch within the actinobacterial phylogenetic tree (Additional file 1: Fig. S6). Some processes such as replication, recombination and repair and transcription, are ubiquitously seen linked to ANTAR, in most genera. In contrast, intracellular trafficking, secretion and vesicular transport process appear restricted to *Gordonia* species while translation related processes and lipid transport and metabolism are largely restricted to non-pathogenic *Mycobacterium* and *Nocardia* species respectively. Energy production and conversion is found to be conserved in species of *Pseudarthrobacter, Renibacterium, Sinomonas, Rhodococcus, Gordonia and Mycobacterium*.

We checked if closely related genera have co-opted ANTAR for similar processes. Indeed, several species from *Bifidobacterium* and *Gardnerella*, have processes such as carbohydrate transport and metabolism, transcription, translation related processes and cell-membrane biogenesis linked to ANTAR. Similarly, six processes including cell energy production and conversion process, transcription and translation related processes, signal transduction mechanisms and amino-acid and carbohydrate transport and metabolism are linked to ANTAR in closely related *Arthrobacter* and *Pseudoarthrobacter* species (Additional file 1: Fig. S6).

KEGG pathway and KEGG BRITE analysis of transporters whose genes are linked to ANTAR, show that they belong to the ABC transporter, MFS sugar transporter and Aquaporin families (Additional file 3: Table S5). The ABC transporter complex consists of multiple components: a periplasmic substrate-binding protein, one or more trans-membrane permeases, an ATP-binding protein and occasionally a substrate-specific enzyme [46]. Interestingly, we find that different components of the transporters, especially the substrate recognizing proteins harbor the ANTAR-target RNA in their mRNA (Fig. 4C). This makes intuitive sense since transporters are often under tight regulation and the different components are made only upon sensing the presence of the cognate sugar/metabolite.

The second highest COG category is that of transcription with over 19 different transcription factor families linked to ANTAR-target RNAs (Additional file 1: Fig. S7A). Remarkably, the majority of these transcription factors are known to regulate the expression of transporter proteins, once again tying back ANTAR-target RNAs to the transport of small molecule metabolites. ~ 29% of transcription factor encoding genes linked to an ANTAR-target RNA belong to the LacI type transcription factor that are major regulators of sugar catabolic genes (Additional file 1: Fig. S7A). For example, in *Streptomyces lydicus,* a LacI transcription factor (TF) carrying an ANTAR-target RNA is present upstream of transporter components involved in ribose uptake (Additional file 1: Fig. S7B). In *Corynebacterium glutamicum*, the homologous TF (*cg1410*) is reported to regulate the downstream *rbsDACBK* operon in response to ribose availability [47]. Similarly, even in *Bifidobacterium dentium*, *Gardnerella vaginalis* and *Microbacterium sp.* and *Cryobacterium arcticum,* ANTAR-target RNAs are also linked to LacI transcription factors that regulate other sugar transporters and sugar related genes (Additional file 1: Table S2, Additional file 2: Table S4). These results suggest that in several actinobacteria sugar transporters, as well as the proteins regulating sugar transport and metabolism are under the influence of ANTAR regulation.

TetR family TFs are also linked to ANTAR-target RNAs in several actinobacterial species (Additional file 1: Fig. S7A). Transcription factors belonging to this family typically regulate the expression of enzymes from different catabolic pathways or proteins involved in multi-drug resistance (Additional file 1: Table S2 and Additional file 2: Table S4) [48, 49]. An ANTAR-target RNA in *Mycobacterium marinum* is found upstream to the *MMAR_RS11360* gene encoding a TetR family transcription factor (Additional file 1: Fig. S6B). Its *M. tuberculosis* homolog, *Rv1474c* is found to cotranscribe with the upstream aconitase gene and regulates aconitase expression in response to iron [50]. A conserved operon in *Streptomyces* species is predicted with an ANTAR-target RNA upstream to a *SufR* encoding gene, *SACTE_RS06635* (Additional file 1: Fig. S7A-B). *SufR* is an ArsR family transcription factor and a repressor of the downstream *sufBCDS* operon, the primary Fe-S assembly cluster system, that responds to the availability of Fe-S cluster required as protein cofactors in many cellular processes [51]. Both these examples underline that an additional layer of post-transcription gene regulation is likely imposed by virtue of ANTAR-target RNAs.

## Discussion

In this study, we identify the repertoire of ANTAR-target RNAs in phylum actinobacteria. Key to our findings was the development of a novel computational search model that was effective in identifying these structured dual stem loop RNA motifs. Covariance search programs rely on both the sequence and the base-pairing information within a search model, to find similar RNA motifs in a genome. Previously reported ANTAR RNA search models [27, 28] either failed or were only partially successful in predicting RNAs in actinobacterial genomes due to a lack of diversity in sequence and base-pairing potential. Removing the bias from highly similar or dissimilar sequences, the new search model developed in this study shows more sequence and structure diversity as compared to the previous models and this was key in identifying ANTAR-target RNA motifs in actinobacteria.

Analysis of the genomic locations of ANTAR-target RNAs from actinobacteria reveals many examples where the RNA is next to the ORF start site, either sequestering the RBS or start codon within the dual stem motif of the ANTAR RNA. A similar genomic arrangement of ANTAR RNAs was seen previously in *Mycobacteria* [28], was shown to function via translational repression. Here, RNAs bound by activated ANTAR protein were shown to repress translation, possibly by preventing ribosomes from accessing the RBS. Our analysis indicates that translational control via ANTAR-target RNAs may be a prominent mode of regulation in actinobacteria.

Analysis of cellular processes likely to be controlled by ANTAR-target RNAs revealed a link between these RNAs and the transport and metabolism of small molecule compounds, especially carbohydrates, amino acids and lipids. Certain species of *Bifidiobacterium*, *Gardnerella* and *Scardovia* show conservation of ANTAR-target RNAs in transcripts encoding carbohydrate transport and metabolism proteins. For these genera, sugar utilization is intricately linked to physiology. For example, *Bifidobacteria* are sacchrolytic intestinal bacteria detected in human and animals [52], while *Scardovia* is detected in human dental caries and adeptly use carbohydrate fermentation pathways to lower the pH of the oral biofilm and likely induce caries progression in the host [53, 54]. Pathogenic *Gardnerella vaginalis* have the ability to degrade glycans in the host mucosal epithelial layers to invade and colonize in the host [55]. Species belonging to genus *Nocardia* shows that ANTAR-target RNAs might regulate lipid transport and metabolism similar to to that seen in *Mycobacteria*. Our results link ANTAR-target RNAs to metabolite transport and utilization in these organisms, possibly indicating that ANTAR regulation may contribute to their growth and survival within their host.

An important finding from our study is the association of ANTAR-target RNAs with mRNAs encoding transcription factors. Transcription factors themselves are regulators of gene-expression, often regulating multiple target genes. By controlling the expression of a transcription factor, even a single ANTAR-target RNA in the genome could indirectly control the expression of multiple genes. We also observed that many of the transcription factors whose mRNAs harbor ANTAR-target RNAs, in fact regulate sugar and other metabolite transport. This implies that the scope of ANTAR-based control of metabolite transport is much broader.

In a recent study in *Streptomyces pristinaespiralis*, deletion of the ANTAR protein *SSDG_04087* led to a bald phenotype (loss of hyphae formation) and reduced production of the antibiotic pristinamycin [43]. In our study, we identify four ANTAR-target RNAs in *S. pristinaespiralis*, one of which lies in the transcript of a sugar (fructose) transporter protein (*SPRI_RS32325*). The uptake of complex sugars by *Streptomyces* favors development (sporulation) and production of antibiotics [56–58]. In fact, perturbation of glycolysis/ gluconeogenesis pathways is a standard method by which to increase the production of antibiotics by *Streptomyces*, for industrial applications [59–62]. Another ANTAR-target RNA is found in the mRNA for the enzyme agmatinase (*SPRI_RS23705*), that converts arginine to putrescine. Putrescine is a precursor of succinate [63, 64] that can feed into the TCA cycle and the synthesis of various amino acids, which are directly involved in the production of the antibiotic pristinamycin [65, 66]. The discovery of these ANTAR-target RNAs in *Streptomyces* thus implicates gene *SPRI_RS32325* and *SPRI_RS23705* as possible candidates that might be investigated to understand the observed phenotype. Our comprehensive description of ANTAR-target RNAs and ANTAR proteins in actinobacteria now provides a resource for microbiologists to mine.

## Conclusion

Our work shows that sequence and structural diversity when introduced in search models, aids in predicting high confidence dual stemloop motifs across phylum actinobacteria. This expands the RNA family that can bind to ANTAR proteins. Actinobacterial ANTAR-target RNAs are distant from the firmicutes and proteobacterial RNAs, yet the core features of ANTAR-target RNAs are conserved across bacteria, highlighting the diversity that can exist within the RNA family. Extensive analyses of the repertoire of ANTAR-target RNAs show that these RNAs can regulate translation of genes involved in metabolite transport, thus underlining the importance of ANTAR in actinobacteria.

## Methods

### Actinobacterial genomes used in this study

720 actinobacterial genomes, with their corresponding gene annotations and proteomes are listed as “Complete genomes” in NCBI (RefSeq v92). These were considered in this study. Corresponding taxon IDs for these organisms were taken from NCBI and a taxonomy tree was retrieved in Phylip format from NCBI Batch Entrez (https://www.ncbi.nlm.nih.gov/sites/batchentrez). The phylogenetic tree visualization was carried out using iTOL [67].

### Predicting ANTAR-target RNAs in actinobacteria using covariance

A search model previously reported for identifying ANTAR-target RNAs in *Mycobacteria* (partially focused search model) [28], was taken and an initial covariance search with a bit score threshold of 10.0 was carried out against actinobacterial genomes using Infernal v1.0.2 [29]. High confidence RNAs with a bit score ≥ 14.0 and showing a dual stem loop structure (with at least 3 base-pairs in each stem and hexanucleotide loops allowing a single point variation) were considered as putative ANTAR-target RNAs. 30 of these predicted RNAs from actinobacteria were taken to form an actinobacteria centric search model (diffused search model). cmbuild analysis of the partially diffused and diffused search models reports on the CM (Covariance model) score where a higher CM score was taken as an indication of highly similar sequences. To identify ANTAR-target RNAs in actinobacteria, 720 genomes representing 315 actinobacterial species were subjected to covariance search using the diffused search model. Hits with a bit score threshold≥15 and lying between 500 nt upstream to 100 nt downstream of the nearest ORF were retained. RNAs that are identical to the search model or are single-nucleotide point variants were considered for further analyses. Redundant identical RNAs from strains were removed and unique RNAs from each species were considered as a representative. We used cmbuild [29] and RNAz [17, 30] to analyze the predicted ANTAR-target RNAs for their sequence and structure similarity. Using the cmaxid option of cmbuild implemented in Infernal v1.0.2, we performed a clustering analysis. Sequence identity cut-off ranging from 30 to 60% was imposed during clustering such that any two RNAs that have sequence identity more than the cut-off, will form a cluster reported with a corresponding CM score. Any group with < 2 RNAs was not considered. The -cdump option of cmbuild writes the multiple sequence alignment for the clusters. The multiple sequence alignment of the largest cluster formed with 55% sequence identity cut-off, was further checked for functionality using RNAz. RNAz calculates i) the structure similarity of the individual RNAs to the consensus structure, reported as structure conservation index (SCI) ii) z-score that describes the standard deviation of the structures formed by the RNAs in a cluster against the structures for a random set of RNAs with same length and base composition, where the negative z-score indicates a true stable structure and has not occurred by chance. Based on these two measures, the RNAs with conserved and stable structures (*P* > 0.5) are considered as a ‘functional RNA’ class. Consensus RNA structure for the largest cluster was visualized using forna [68] and nucleotide-level resolution of the consensus structure was obtained using R2R [69] and statistically significant covarying positions were identified using R-scape [70].

### Distribution of ANTAR domain containing proteins in actinobacteria

An HMM model for the ANTAR domain was taken from Pfam v33.0 (PF03861) and HMMsearch (hmmer v3.2.1) was performed against all actinobacterial proteomes with e-value threshold 1e-4. E-value threshold was determined from previous studies [71–75]. This identified proteins having ANTAR domains. Proteomes where the HMMsearch failed to identify ANTAR proteins, were further searched for sequences homologous to the Rv1626 ANTAR domain using BLASTp with evalue threshold 1e-3. E-value threshold was determined from previous studies [76–79].

### Categorizing ANTAR-target RNAs based on location within the genomic context

ANTAR-target RNAs were grouped into 3 categories based on their distance from ORFs. RNAs (including 10 nt flanking region) that are 15 nt upstream from start of ORF, were assigned to ‘intergenic’ group. RNAs that completely reside within the ORF were assigned to ‘inside ORF’ group. RNAs that harbor a potential RBS as part of the RNA structure, are grouped as ‘sequester RBS or AUG’. RNAs were also subjected to alternate ORF (altORFs) prediction using standalone NCBI ORFfinder (https://www.ncbi.nlm.nih.gov/orffinder/) with default parameters allowing for ATG or any alternate start codons. Predicted ORFs which harbor a potential ribosome-binding site (RBS) with a 4-6 nt AG-rich region and reside 0-15 nt upstream of the start codon are considered as putative altORFs. RNAs from the ‘intergenic’ group were further subjected to Rho-independent terminator prediction. Here, target-RNA sequences along with 40 nt downstream sequences were given to TransTermHP [34] with parameters uwin-require = 0 and min-conf = 50.

### COG and KEGG pathway analyses for ANTAR targets

Protein sequences of genes linked to ANTAR-target RNAs were subjected to COG analysis using EggNOG mapper v4.5.1 (http://eggnogdb.embl.de/#/app/emapper). A minimum 70% query coverage and e-value default threshold 1e-3 was used to assign COG categories and KEGG orthologs (KO) based on sequence homology. E-value threshold was determined from previous studies [80–82]. Independently, these protein sequences were given as input to KofamKOALA (https://www.genome.jp/tools/kofamkoala/) with e-value default threshold 1e-2, which reports on top KEGG orthologs using an HMMsearch. E-value threshold was determined from previous studies [83, 84]. Orthologs for genes linked to ANTAR-target RNAs, were mapped using EggNOG and/or KofamKOALA (Additional file 2: Table S4). These KOs were then given to KEGGmapper (“KEGG reconstruct pathway” and “KEGG search and color pathway”) for pathway analyses. Visualization of data was carried out in iTOL and the pathway graphs were obtained using KEGG and modified using Adobe Illustrator. All plots were obtained using Graphpad Prism v8.0.

## Supplementary Information

**Additional file 1: Supplementary Figures and Tables.** Data linked to Figs. 1-4 are given in Supplementary Figs. S1-S7, Supplementary Table S1, Supplementary Table S2 and Supplementary Table S3.

**Additional file 2: Supplementary Table S4.** Supplementary Table S4 linked to data in Fig. 4 is given.

**Additional file 3: Supplementary Table S5.** Supplementary Table S5 linked to data in Fig. 4 is given.

## Data Availability

All RNA sequences reported in this study are given in supplementary files (Table S2 and Table S3). Accession IDs from publicly available NCBI database: https://www.ncbi.nlm.nih.gov/, genomic locations and RNA sequences for these RNAs are also given in Table S2 and Table S3. ANTAR proteins and ANTAR-linked genes are referred to with protein accession numbers which are taken from NCBI database. The files are also available from the corresponding author on reasonable request.

## Abbreviations

UTR: Untranslated regions
MFE: Minimum free energy
Rfam: RNA family database
Pfam: Protein family database
CM score: Covariance model score
SCI: Structure conservation index
RBS: Ribosome binding site
altORFs: Alternate open reading frames
COG: Cluster of Orthologous groups
KO: KEGG Ortholog
TF(s): Transcription factor(s)

## References

[1] Parks DH, Chuvochina M, Waite DW, Rinke C, Skarshewski A, Chaumeil PA, Hugenholtz P (2018). A standardized bacterial taxonomy based on genome phylogeny substantially revises the tree of life. Nat Biotechnol.

[2] Ventura M, Canchaya C, Tauch A, Chandra G, Fitzgerald GF, Chater KF, van Sinderen D (2007). Genomics of Actinobacteria: tracing the evolutionary history of an ancient phylum. Microbiol Mol Biol Rev.

[3] Barrick JE, Breaker RR (2007). The distributions, mechanisms, and structures of metabolite-binding riboswitches. Genome Biol.

[4] Kazanov MD, Vitreschak AG, Gelfand MS (2007). Abundance and functional diversity of riboswitches in microbial communities. BMC Genomics.

[5] Hör J, Gorski SA, Vogel J (2018). Bacterial RNA biology on a genome scale. Mol Cell Cell Press.

[6] Updegrove TB, Zhang A, Storz G. Hfq: The flexible RNA matchmaker. Curr Opin Microbiol Elsevier Ltd. 2016:133–8. 10.1016/j.mib.2016.02.003. 10.1016/j.mib.2016.02.003 PMC4821791 26907610

[7] Durand S, Tomasini A, Braun F, Condon C, Romby P (2015). sRNA and mRNA turnover in gram-positive bacteria. FEMS Microbiol Rev. Oxford University Press.

[8] Repoila F, Darfeuille F (2009). Small regulatory non-coding RNAs in bacteria: physiology and mechanistic aspects. Biol Cell.

[9] Hoeppner MP, Gardner PP, Poole AM (2012). Comparative Analysis of RNA Families Reveals Distinct Repertoires for Each Domain of Life. Wilke CO, editor. PLoS Comput Biol.

[10] Seliverstov AV, Putzer H, Gelfand MS, Lyubetsky VA (2005). Comparative analysis of RNA regulatory elements of amino acid metabolism genes in Actinobacteria. BMC Microbiol.

[11] Mentz A, Neshat A, Pfeifer-Sancar K, Pühler A, Rückert C, Kalinowski J (2013). Comprehensive discovery and characterization of small RNAs in Corynebacterium glutamicum ATCC 13032. BMC Genomics.

[12] Peng T, Kan J, Hu J, Hu Z. Genes and novel sRNAs involved in PAHs degradation in marine bacteria Rhodococcus sp. P14 revealed by the genome and transcriptome analysis. 3 Biotech. 2020;10: 140. doi:10.1007/s13205-020-2133-6 10.1007/s13205-020-2133-6 PMC7044393 32206489

[13] Pánek J, Krásný L, Bobek J, Ježková E, Korelusová J, Vohradský J (2011). The suboptimal structures find the optimal RNAs: homology search for bacterial non-coding RNAs using suboptimal RNA structures. Nucleic Acids Res.

[14] Engel F, Ossipova E, Jakobsson P-J, Vockenhuber M-P, Suess B (2020). sRNA scr5239 involved in feedback loop regulation of Streptomyces coelicolor central metabolism. Front Microbiol.

[15] Taneja S, Dutta T. On a stake-out: Mycobacterial small RNA identification and regulation. Noncoding RNA Res KeAi Communications Co. 2019:86–95. 10.1016/j.ncrna.2019.05.001. 10.1016/j.ncrna.2019.05.001 PMC7017587 32083232

[16] Heueis N, Vockenhuber M-P, Suess B (2014). Small non-coding RNAs in streptomycetes. RNA Biol.

[17] Washietl S, Hofacker IL, Stadler PF (2005). Fast and reliable prediction of noncoding RNAs. Proc Natl Acad Sci U S A.

[18] Vitreschak AG, Mironov A, Gelfand M (2001). The RNApattern program: searching for RNA secondary structure by the pattern rule.

[19] Lorenz R, Bernhart SH, Hoener C, Siederdissen Z, Tafer H, Flamm C (2011). ViennaRNA package 2.0 algorithms for molecular biology ViennaRNA package 2.0. Algorithms Mol Biol.

[20] Vockenhuber MP, Sharma CM, Statt MG, Schmidt D, Xu Z, Dietrich S, Liesegang H, Mathews DH, Suess B (2011). Deep sequencing-based identification of small non-coding RNAs in Streptomyces coelicolor. RNA Biol.

[21] Vitreschak AG, Rodionov DA, Mironov AA, Gelfand MS (2003). Regulation of the vitamin B12 metabolism and transport in bacteria by a conserved RNA structural element. RNA.

[22] Chai W, Stewart V (1998). NasR, a novel RNA-binding protein, mediates nitrate-responsive transcription antitermination of the Klebsiella oxytoca M5al nasF operon leader in vitro. J Mol Biol.

[23] Drew R, Lowe N (1989). Positive control of Pseudomonas aeruginosa amidase synthesis is mediated by a transcription anti-termination mechanism. J Gen Microbiol.

[24] Wilson SA, Wachira SJ, Norman RA, Pearl LH, Drew RE (1996). Transcription antitermination regulation of the Pseudomonas aeruginosa amidase operon. EMBO J.

[25] Goldman BS, Lin JT, Stewart V (1994). Identification and structure of the nasR gene encoding a nitrate- and nitrite-responsive positive regulator of nasFEDCBA (nitrate assimilation) operon expression in Klebsiella pneumoniae M5al. J Bacteriol.

[26] Ueki T, Lovley DR (2010). Novel regulatory cascades controlling expression of nitrogen-fixation genes in Geobacter sulfurreducens. Nucleic Acids Res.

[27] Ramesh A, DebRoy S, Goodson JR, Fox KA, Faz H, Garsin DA (2012). The Mechanism for RNA Recognition by ANTAR Regulators of Gene Expression. Burkholder WF, editor. PLoS Genet.

[28] Mehta D, Koottathazhath A, Ramesh A (2020). Discovery of ANTAR-RNAs and their mechanism of action in mycobacteria. J Mol Biol.

[29] Nawrocki EP, Kolbe DL, Eddy SR (2009). Infernal 1.0: inference of RNA alignments. Bioinformatics.

[30] Altman RB, Dunker AK, Hunter L, Murray TA, Klein TE, GRUBER AR, et al. RNAZ 2.0: Biocomputing 2010. WORLD SCIENTIFIC. 2009:69–79. 10.1142/9789814295291_0009.

[31] Weber AM, Kaiser J, Ziegler T, Pilsl S, Renzl C, Sixt L, Pietruschka G, Moniot S, Kakoti A, Juraschitz M, Schrottke S, Lledo Bryant L, Steegborn C, Bittl R, Mayer G, Möglich A (2019). A blue light receptor that mediates RNA binding and translational regulation. Nat Chem Biol.

[32] Fox KA, Ramesh A, Stearns JE, Bourgogne A, Reyes-Jara A, Winkler WC, Garsin DA (2009). Multiple posttranscriptional regulatory mechanisms partner to control ethanolamine utilization in Enterococcus faecalis. Proc Natl Acad Sci U S A.

[33] Malaka De Silva P, Patidar R, Graham CI, AKC B, Kumar A (2020). A response regulator protein with antar domain, avnr, in acinetobacter baumannii ATCC 17978 impacts its virulence and amino acid metabolism. Microbiol (United Kingdom).

[34] Kingsford CL, Ayanbule K, Salzberg SL (2007). Rapid, accurate, computational discovery of rho-independent transcription terminators illuminates their relationship to DNA uptake. Genome Biol.

[35] Tapsin S, Sun M, Shen Y, Zhang H, Lim XN, Susanto TT, Yang SL, Zeng GS, Lee J, Lezhava A, Ang EL, Zhang LH, Wang Y, Zhao H, Nagarajan N, Wan Y (2018). Genome-wide identification of natural RNA aptamers in prokaryotes and eukaryotes. Nat Commun.

[36] Del Campo C, Bartholomäus A, Fedyunin I, Ignatova Z (2015). Secondary Structure across the Bacterial Transcriptome Reveals Versatile Roles in mRNA Regulation and Function. Toledo-Arana A, editor. PLoS Genet.

[37] Tsuchihashi Z, Kornberg A. Translational frameshifting generates the gamma subunit of DNA polymerase III holoenzyme. Proc Natl Acad Sci USA. 1990;87(7):2516–20. 10.1073/pnas.87.7.2516. 10.1073/pnas.87.7.2516 PMC53720 2181440

[38] Chen C, Zhang H, Broitman SL, Reiche M, Farrell I, Cooperman BS, Goldman YE (2013). Dynamics of translation by single ribosomes through mRNA secondary structures. Nat Struct Mol Biol.

[39] Gorochowski TE, Ignatova Z, Bovenberg RAL, Roubos JA (2015). Trade-offs between tRNA abundance and mRNA secondary structure support smoothing of translation elongation rate. Nucleic Acids Res.

[40] Murat P, Zhong J, Lekieffre L, Cowieson NP, Clancy JL, Preiss T, Balasubramanian S, Khanna R, Tellam J (2014). G-quadruplexes regulate Epstein-Barr virus-encoded nuclear antigen 1 mRNA translation. Nat Chem Biol.

[41] Caliskan N, Peske F, Rodnina MV (2015). Changed in translation: MRNA recoding by −1 programmed ribosomal frameshifting. Trends in Biochemical Sciences. Elsevier Ltd.

[42] Giedroc DP, Cornish PV (2009). Frameshifting RNA pseudoknots: structure and mechanism. Virus Res.

[43] Li L, Zhao Y, Ma J, Tao H, Zheng G, Chen J, Jiang W, Lu Y (2020). The orphan histidine kinase PdtaS-p regulates both morphological differentiation and antibiotic biosynthesis together with the orphan response regulator PdtaR-p in Streptomyces. Microbiol Res.

[44] Huerta-Cepas J, Szklarczyk D, Heller D, Hernández-Plaza A, Forslund SK, Cook H, Mende DR, Letunic I, Rattei T, Jensen LJ, von Mering C, Bork P (2019). EggNOG 5.0: a hierarchical, functionally and phylogenetically annotated orthology resource based on 5090 organisms and 2502 viruses. Nucleic Acids Res.

[45] Huerta-Cepas J, Forslund K, Coelho LP, Szklarczyk D, Jensen LJ, Von Mering C (2017). Fast genome-wide functional annotation through orthology assignment by eggNOG-mapper. Mol Biol Evol.

[46] Higgins CF. ABC Transporters: From microorganisms to man. Ann Rev Cell Biol. 1992:67–113. 10.1146/annurev.cb.08.110192.000435. 10.1146/annurev.cb.08.110192.000435 1282354

[47] Nentwich SS, Brinkrolf K, Gaigalat L, Hüser AT, Rey DA, Mohrbach T, Marin K, Pühler A, Tauch A, Kalinowski J (2009). Characterization of the LacI-type transcriptional repressor RbsR controlling ribose transport in Corynebacterium glutamicum ATCC 13032. Microbiology..

[48] Ramos JL, Martínez-Bueno M, Molina-Henares AJ, Terán W, Watanabe K, Zhang X (2005). The TetR family of transcriptional repressors. Microbiol Mol Biol Rev.

[49] Bhukya H, Anand R (2017). TetR regulators: a structural and functional perspective. J Indian Inst Sci.

[50] Balakrishnan K, Mohareer K, Banerjee S (2017). Mycobacterium tuberculosis Rv1474c is a TetR-like transcriptional repressor that regulates aconitase, an essential enzyme and RNA-binding protein, in an iron-responsive manner. Tuberculosis.

[51] Cheng Y, Lyu M, Yang R, Wen Y, Song Y, Li J, et al. SufR, a [4Fe-4S] cluster-containing transcription factor, represses the sufRBDCSU operon in Streptomyces avermitilis iron-sulfur cluster assembly. Appl Environ Microbiol. 2020;86(18). 10.1128/AEM.01523-20. 10.1128/AEM.01523-20 PMC7480368 32680866

[52] Pokusaeva K, Fitzgerald GF, Van Sinderen D (2011). Carbohydrate metabolism in Bifidobacteria. Genes Nutr. BioMed Central.

[53] Kressirer CA, Smith DJ, King WF, Dobeck JM, Starr JR, ACR T. *Scardovia wiggsiae* and its potential role as a caries pathogen. Journal of Oral Biosciences. Japanese Assoc Oral Biol. 2017:135–41. 10.1016/j.job.2017.05.002. 10.1016/j.job.2017.05.002 PMC5665406 29104444

[54] Kameda M, Abiko Y, Washio J, Tanner ACR, Kressirer CA, Mizoguchi I, et al. Sugar metabolism of Scardovia wiggsiae, a novel caries-associated bacterium. Front Microbiol. 2020;11. 10.3389/fmicb.2020.00479. 10.3389/fmicb.2020.00479 PMC7109253 32269556

[55] Lewis WG, Robinson LS, Gilbert NM, Perry JC, Lewis AL (2013). Degradation, foraging, and depletion of mucus sialoglycans by the vagina-adapted actinobacterium Gardnerella vaginalis. J Biol Chem.

[56] Rueda B, Miguélez EM, Hardisson C, Manzanal MB (2001). Changes in glycogen and trehalose content of Streptomyces brasiliensis hyphae during growth in liquid cultures under sporulating and non-sporulating conditions. FEMS Microbiol Lett.

[57] Światek MA, Urem M, Tenconi E, Rigali S, van Wezel GP (2012). Engineering of N-acetylglucosamine metabolism for improved antibiotic production in Streptomyces coelicolor A3(2) and an unsuspected role of NagA in glucosamine metabolism. Bioengineered..

[58] Rafieenia R. Effect of nutrients and culture conditions on antibiotic synthesis in Streptomycetes. Asian J Pharm Health Sci. 2013;3(3):810–15.

[59] Butler MJ, Bruheim P, Jovetic S, Marinelli F, Postma PW, Bibb MJ (2002). Engineering of primary carbon metabolism for improved antibiotic production in Streptomyces lividans. Appl Environ Microbiol.

[60] Li R, Townsend CA (2006). Rational strain improvement for enhanced clavulanic acid production by genetic engineering of the glycolytic pathway in Streptomyces clavuligerus. Metab Eng.

[61] Ryu YG, Butler MJ, Chater KF, Lee KJ (2006). Engineering of primary carbohydrate metabolism for increased production of actinorhodin in Streptomyces codicolor. Appl Environ Microbiol.

[62] Huang D, Wen J, Wang G, Yu G, Jia X, Chen Y (2012). In silico aided metabolic engineering of Streptomyces roseosporus for daptomycin yield improvement. Appl Microbiol Biotechnol.

[63] Krysenko S, Okoniewski N, Kulik A, Matthews A, Grimpo J, Wohlleben W, et al. Gamma-glutamylpolyamine synthetase GlnA3 is involved in the first step of polyamine degradation pathway in Streptomyces coelicolor M145. Front Microbiol. 2017;8. 10.3389/fmicb.2017.00726. 10.3389/fmicb.2017.00726 PMC5403932 28487688

[64] Schneider BL, Reitzer L (2012). Pathway and enzyme redundancy in putrescine catabolism in Escherichia coli. J Bacteriol.

[65] Voelker F, Altaba S. Nitrogen source governs the patterns of growth and pristinamycin production in “Streptomyces pristinaespiralis.” Microbiol. 2001;147:2447–59. 10.1099/00221287-147-9-2447. 10.1099/00221287-147-9-2447 11535785

[66] Zhang LJ, Jin ZH, Chen XG, Jin QC, Feng MG (2012). Glycine feeding improves pristinamycin production during fermentation including resin for in situ separation. Bioprocess Biosyst Eng.

[67] Letunic I, Bork P (2016). Interactive tree of life (iTOL) v3: an online tool for the display and annotation of phylogenetic and other trees. Nucleic Acids Res.

[68] Kerpedjiev P, Hammer S, Hofacker IL (2015). Forna (force-directed RNA): simple and effective online RNA secondary structure diagrams. Bioinformatics.

[69] Weinberg Z, Breaker RR (2011). R2R--software to speed the depiction of aesthetic consensus RNA secondary structures. BMC Bioinformatics.

[70] Rivas E, Clements J, Eddy SR (2016). A statistical test for conserved RNA structure shows lack of evidence for structure in lncRNAs. Nat Methods.

[71] Mudgal R, Sowdhamini R, Chandra N, Srinivasan N, Sandhya S (2014). Filling-in void and sparse regions in protein sequence space by protein-like artificial sequences enables remarkable enhancement in remote homology detection capability. J Mol Biol.

[72] Bradshaw CR, Surendranath V, Henschel R, Mueller MS, Habermann BH (2011). HMMerthread: detecting remote, functional conserved domains in entire genomes by combining relaxed sequence-database searches with fold recognition. PLoS One.

[73] Covert BA, Spencer JS, Orme IM, Belisle JT (2001). The application of proteomics in defining the T cell antigens of Mycobacterium tuberculosis. Proteomics.

[74] Tan C, Liu Z, Huang S, Li C, Ren J, Tang X, Liu W, Peng S, Feng H (2018). Pectin methylesterase inhibitor (PMEI) family can be related to male sterility in Chinese cabbage (Brassica rapa ssp. pekinensis). Mol Gen Genomics.

[75] Câmara GA, Nishiyama-Jr MY, Kitano ES, Oliveira UC, da Silva PI, Junqueira-de-Azevedo IL (2020). A multiomics approach unravels new toxins with possible in silico antimicrobial, antiviral, and Antitumoral activities in the venom of Acanthoscurria rondoniae. Front Pharmacol.

[76] Pi B, Yu D, Dai F, Song X, Zhu C, Li H (2015). A Genomics Based Discovery of Secondary Metabolite Biosynthetic Gene Clusters in Aspergillus ustus. Andersen MR, editor. PLoS One.

[77] Palanisamy N (2018). Identification of putative drug targets and annotation of unknown proteins in Tropheryma whipplei. Comput Biol Chem.

[78] Aherfi S, Andreani J, Baptiste E, Oumessoum A, Dornas FP, Andrade AC dos SP, et al. A Large Open Pangenome and a Small Core Genome for Giant Pandoraviruses Front Microbiol 2018;9: 1486. doi:10.3389/fmicb.2018.01486. 10.3389/fmicb.2018.01486 PMC6048876 30042742

[79] Manivel G, Meyyazhagan A, Durairaj DR, Piramanayagam S (2019). Genome-wide analysis of excretory/secretory proteins in Trypanosoma brucei brucei: insights into functional characteristics and identification of potential targets by immunoinformatics approach. Genomics.

[80] Allioux M, Jebbar M, Slobodkina G, Slobodkin A, Moalic Y, Frolova A, Shao Z, Alain K (2021). Complete genome sequence of Thermosulfurimonas marina SU872T, an anaerobic thermophilic chemolithoautotrophic bacterium isolated from a shallow marine hydrothermal vent. Mar Genomics.

[81] Bergk Pinto B, Maccario L, Dommergue A, Vogel TM, Larose C. Do organic substrates drive microbial community interactions in Arctic snow? Front Microbiol. 2019;10. 10.3389/fmicb.2019.02492. 10.3389/fmicb.2019.02492 PMC6842950 31749784

[82] Kiu R, Caim S, Alexander S, Pachori P, Hall LJ (2017). Probing genomic aspects of the multi-host pathogen Clostridium perfringens reveals significant pangenome diversity, and a diverse array of virulence factors. Front Microbiol.

[83] Neely CJ, Graham ED, Tully BJ (2020). MetaSanity: an integrated microbial genome evaluation and annotation pipeline. Valencia a, editor. Bioinformatics.

[84] Zhao X, Bai S, Li L, Han X, Li J, Zhu Y, Fang Y, Zhang D, Li S (2020). Comparative transcriptome analysis of two Aegilops tauschii with contrasting drought tolerance by RNA-Seq. Int J Mol Sci.

