# Supplementary material for: Diversity and prevalence of ANTAR RNAs across actinobacteria
Source: BMC Microbiol. 2021 May 29;21:159. doi: 10.1186/s12866-021-02234-x (PMC8164766; doi:10.1186/s12866-021-02234-x)
Supplement: Supplementary file 1 — Additional file 1: Supplementary Figures and Tables. Data linked to Figs. 1-4 are given in Supplementary Figs. S1-S7, Supplementary Table S1, Supplementary Table S2 and Supplementary Table S3. [file 12866_2021_2234_MOESM1_ESM.pdf]

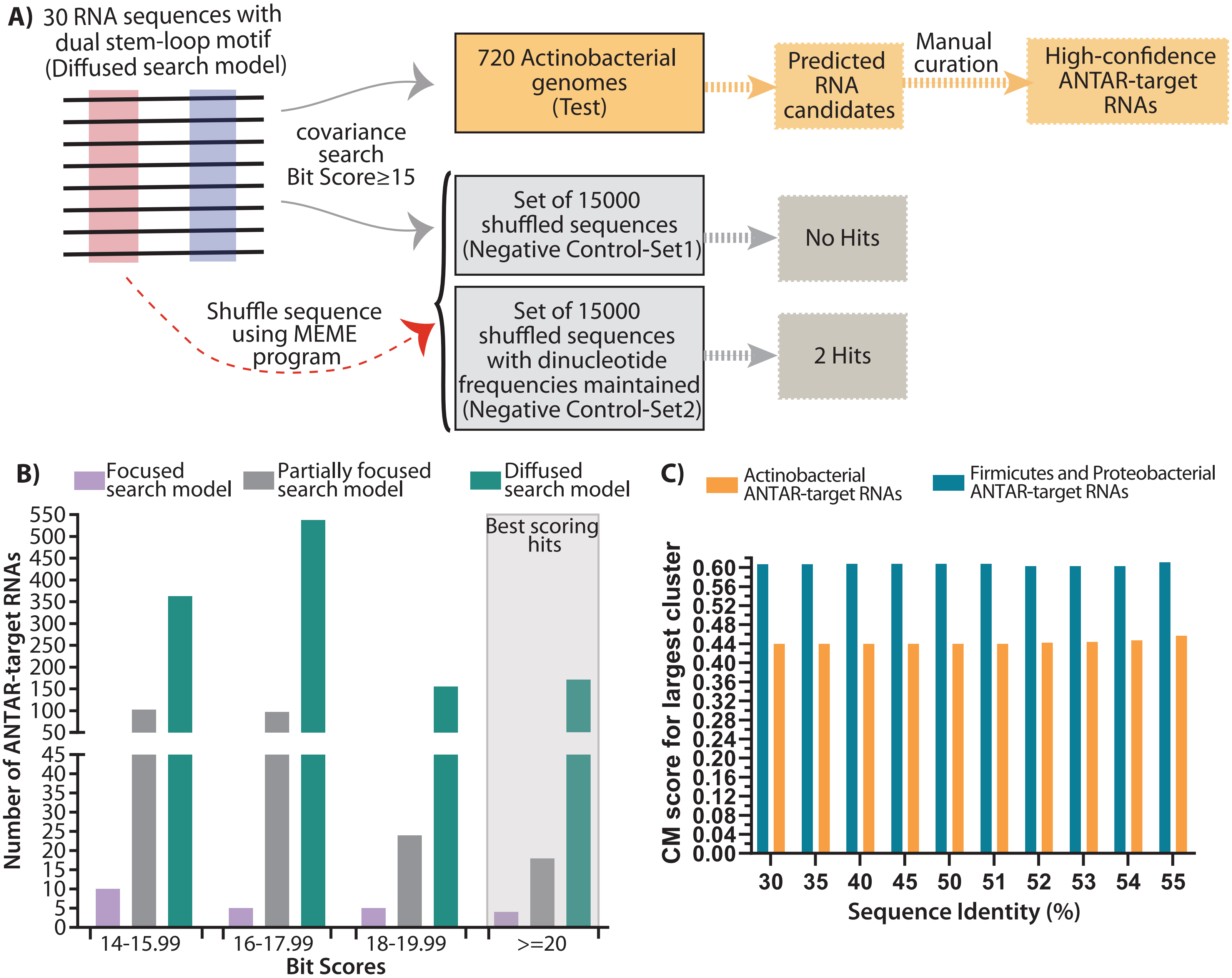

Fig S1. A) Schematic shows the steps followed to identify ANTAR-target RNAs across phylum actinobacteria. Diffused search model consisting of 30 sequences harboring the dual stem-loop (Red and Blue) structure motif was used for covariance searches. As a negative control, the sequences in the diffused search model were taken and shuffled to create 2 sets (gray). First negative control set (Set1) was obtained where the nucleotides in each sequence were shuffled (using kmer=1) and Set2 was obtained by shuffling the sequences such that the dinucleotide frequencies are maintained (using kmer=2). Covariance search was performed using diffused search model against actinobacterial genomes (test set, yellow) and negative control sets with a bit score threshold of 15.00. Our search identified RNA candidates from the test actinobacterial genomes but not from the negative control sets. Predicted RNA candidates from actinobacteria are manually curated to report on high confidence ANTAR-target RNAs. B) Bar plot shows the distribution of bit scores obtained for actinobacterial ANTAR-target RNAs identified using 3 search models (focused/ partially focused/ diffused). Bit scores report on the similarity of each RNA hit to the search model. Diffused search model yields RNAs of higher bit scores. C) RNA sets from firmicutes/ proteobacteria and actinobacteria were clustered using cmbuild. CM score distribution of the largest cluster obtained at varying sequence identity (30%-60%) is shown as bar plots.

A)

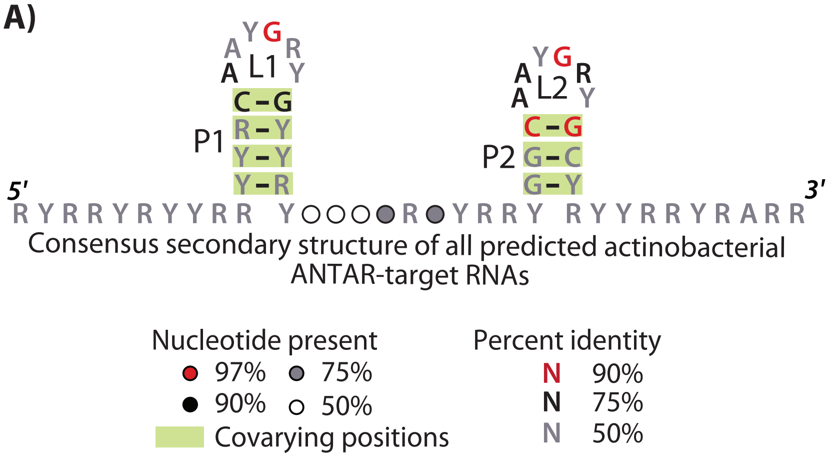

B) ***Parameters for all ANTAR-target RNAs***

	RNA Pool-1	RNA Pool-2
Number of RNAs	305	306
Mean pairwise identity (mPID)	33.38	33.72
Consensus MFE	-7.88	-8.06
Structure conservation index (SCI)	0.49	0.50
Mean z-score	-1.07	-1.04
SVM RNA-class probability (P)	0.98	0.98

Fig S2. A) Consensus is drawn for all actinobacterial ANTAR-target RNA sequences using R2R and significantly covarying positions are mapped using R-scape. B) All the actinobacterial RNA hits were randomly divided into 2 pools (RNA Pool-1 and RNA Pool-2) and analyzed using RNAz. Parameters obtained from RNAz for each of the RNA pools are shown.

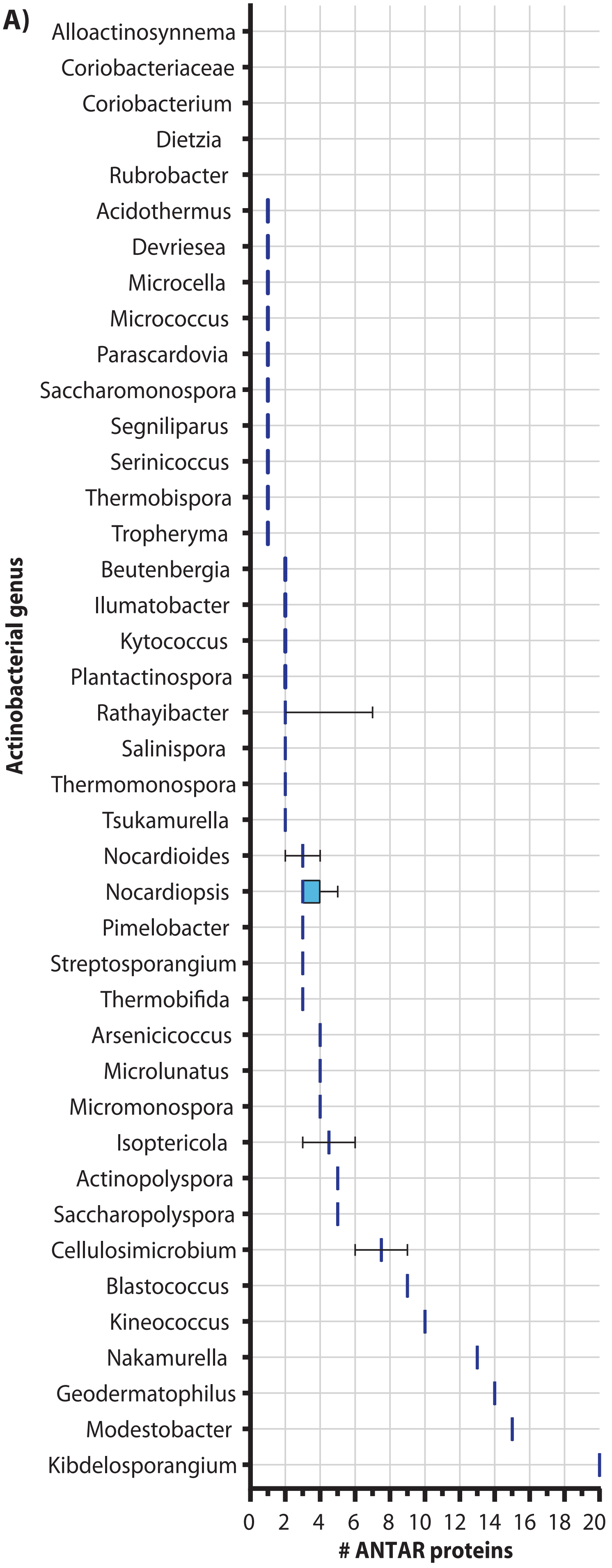

Fig S3. Distribution of ANTAR proteins in actinobacterial genera where ANTAR-target RNAs are not predicted. Box-Whisker plot with median (horizontal line), interquartile range (box) and 1.5 times the interquartile range (whiskers) is shown.

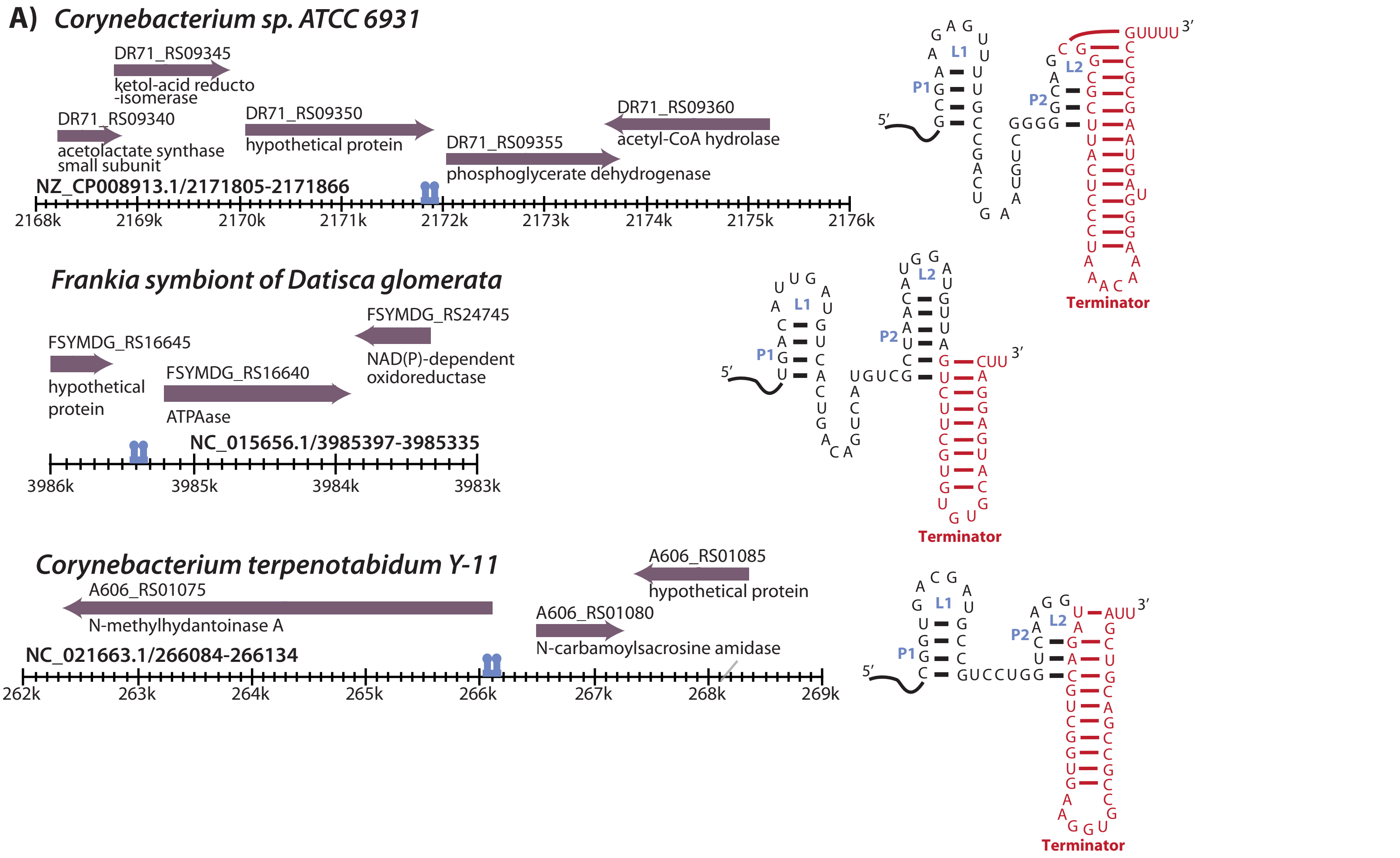

Fig S4: ANTAR-target RNAs categorized as 'intergenic' based on their location, were subjected to rho-independent transcription terminator analyses using TransTerm. Representative RNAs are shown with the predicted transcription terminator (red). The terminator structure is mutually exclusive from the dual stem ANTAR-target RNA structure.

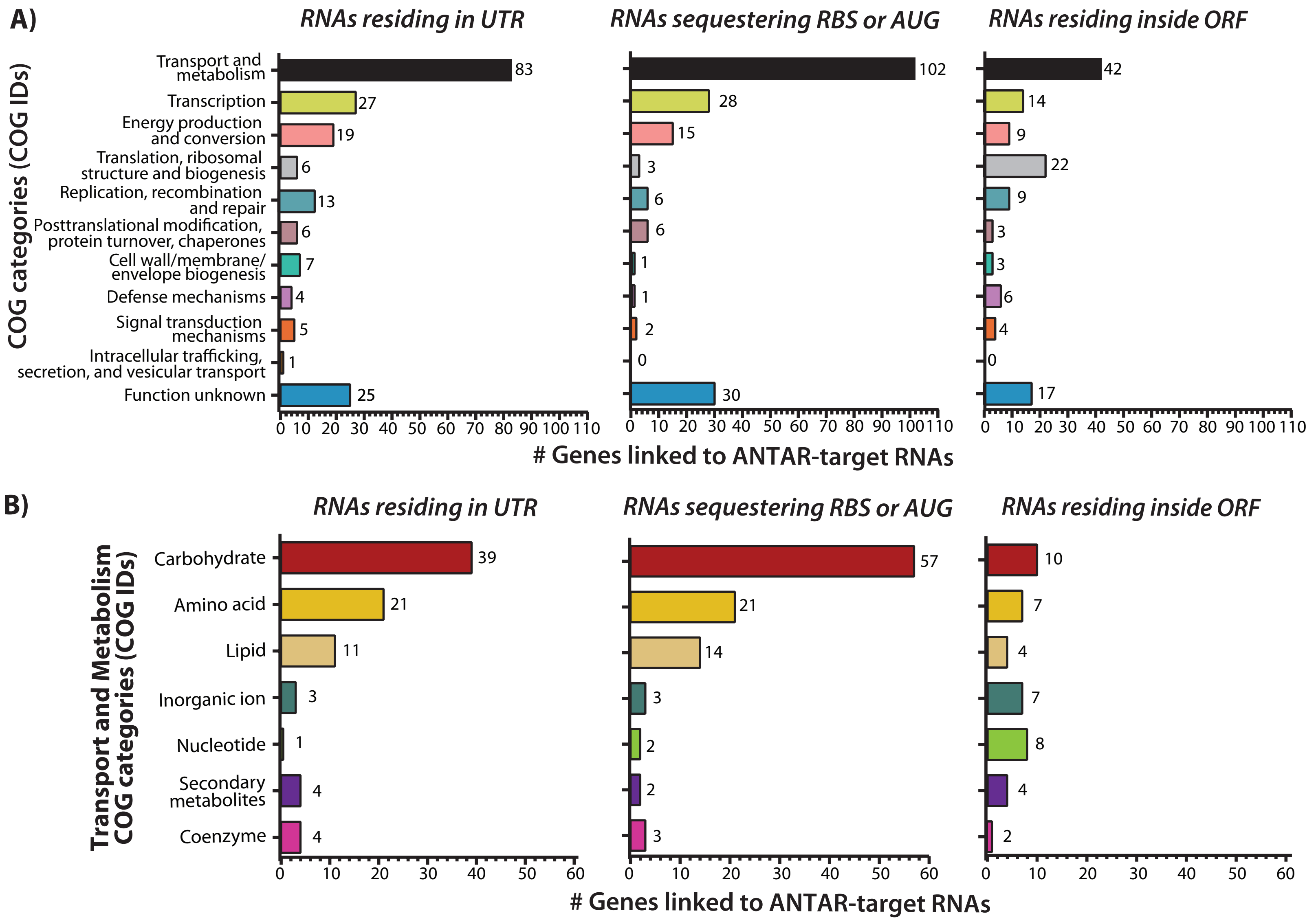

Fig S5. A) Genes linked to ANTAR-target RNAs grouped in UTR, sequester RBS or AUG, inside ORF categories, when analysed individually using EggNOG-mapper, get assigned to 11 COG categories. Bar plot shows distribution of genes linked to ANTAR-target RNAs, in each COG category. B) Bar plot shows distribution of genes linked to ANTAR-target RNAs, within the 'transport and metabolism' COG category. Carbohydrate and amino-acid transport and metabolism are the major processes represented by the targets.

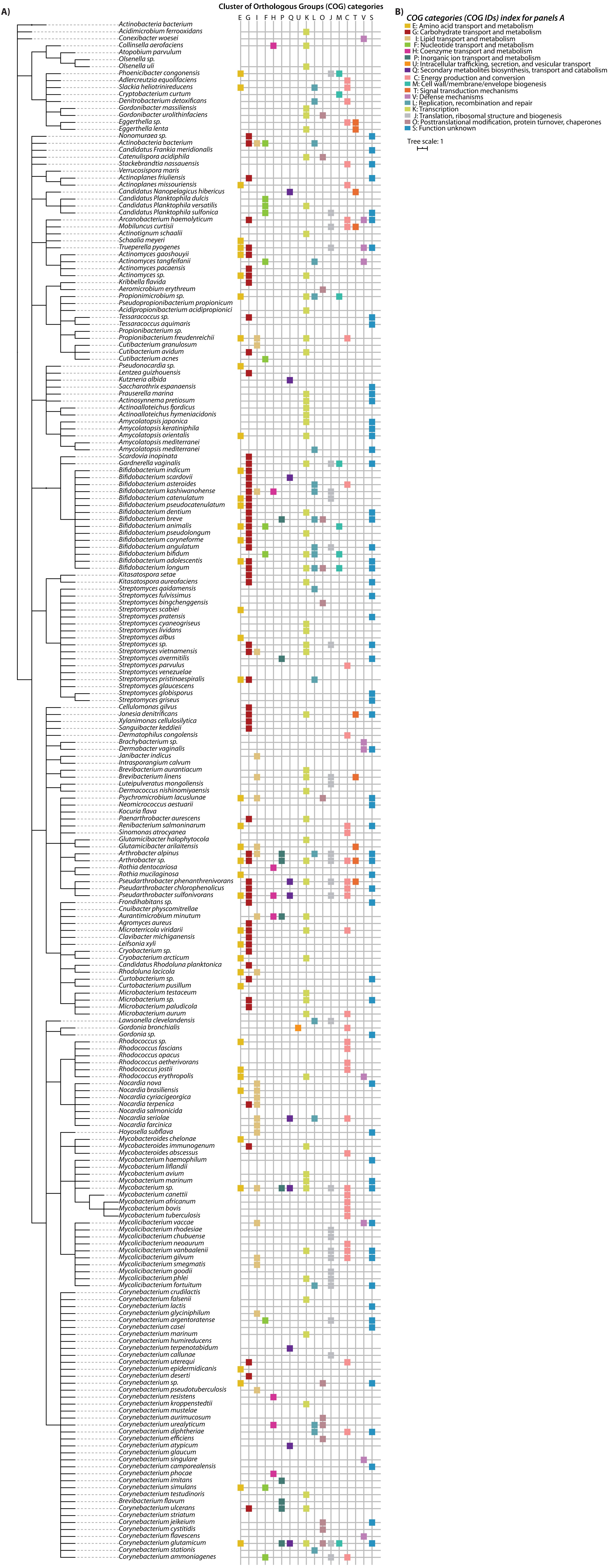

Fig S6. A) Phylogenetic tree obtained from the NCBI taxonomy of actinobacterial species is plotted using iTOL. COG categories of genes linked to ANTAR-target RNAs are plotted using iTOL-binary dataset type with the color notations and abbreviations given in panel B.

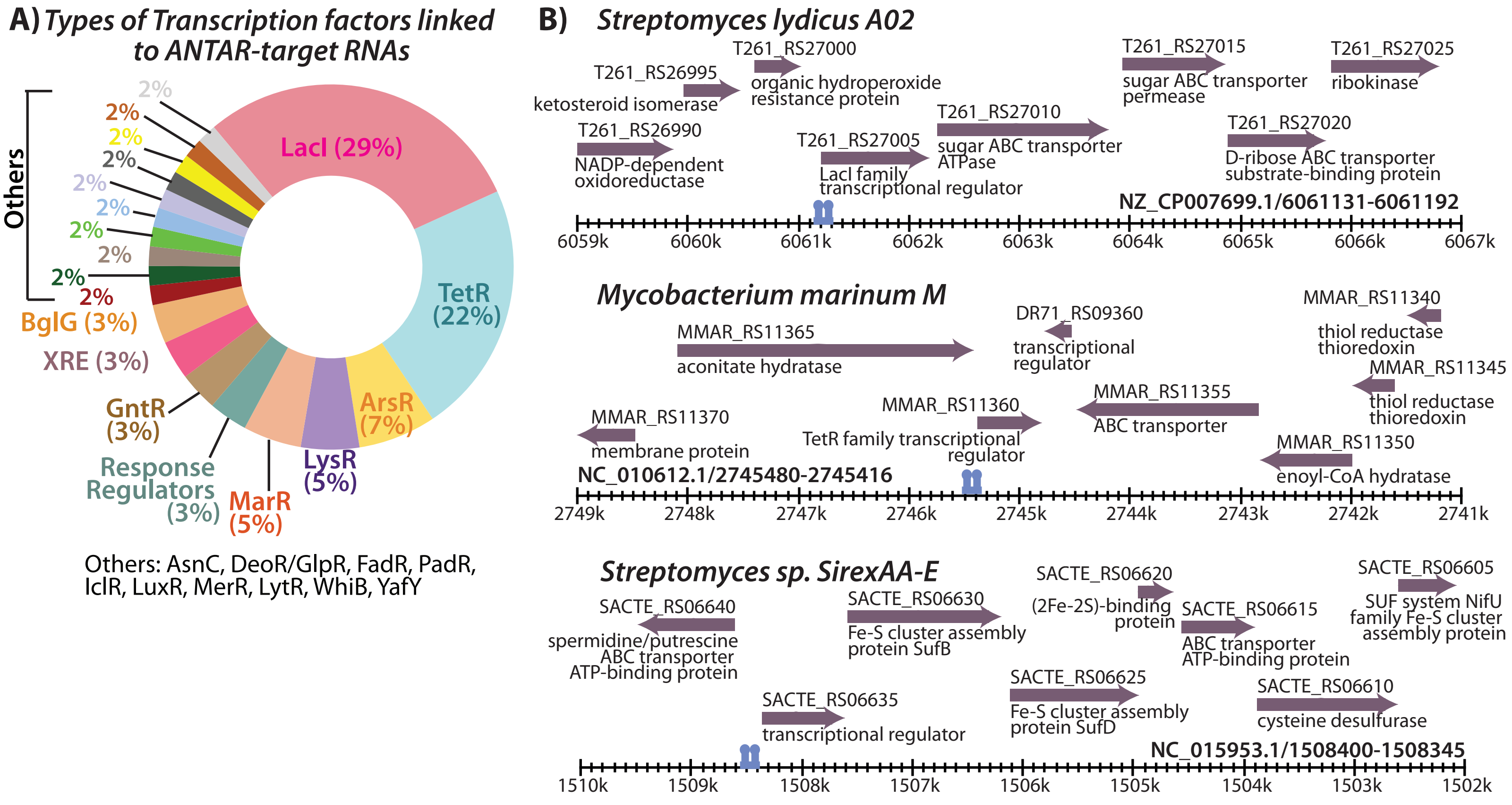

Fig S7. ANTAR-target RNAs linked to genes encoding transcription factors. A) Pie-chart shows the types of transcription factor families linked with ANTAR-target RNAs in actinobacteria. Many of the ANTAR-target RNAs are found upstream to LacI and TetR family transcription factors. (B) Genomic contexts of ANTAR-target RNAs (blue) lying upstream of a transcription factor. ORFs (purple) are shown with their operonic arrangement and labeled with NCBI gene annotations.

Table S1: Diffused search model for ANTAR-target RNA dual stemloop motif.

30 representative RNAs from actinobacteria predicted using partially diffused search model are used to make the improvised search model (Diffused search model). Base paired nucleotides in stems are shaded in pink and blue and secondary structure of the RNA is shown in WUSS notation where '<' and '>' indicates base-pairing positions.

**Accession/Start-End**	**Sequence**
NC_019395/1187953-1188002	.AAAUACAGCuCAGUACUGU-..AUUG--....................UUAUCGGCAUGGACGCCAAUGACGAUG
NC_022567/1325289-1325349	.AAAAAAUCUCUUGCAAUGAU..GCAACGGuc............ggGUUAUCAUAGCAAAGCUGCUUGAGCGGAAG
NZ_CP008953/3466226-3466279	.AAAGUAACAGAUACAACGAC..GUAUC--................-CCUUAUCCAUGACGGUAUGCUCAGCGAAG
NC_014218/792299-792362	.UGCGUGAUAUGCACGAUGAC..GUGCUUUuau.........acgcAUCACUAUGUCAAUGGAGACACUAGAAAAG
NZ_CP013200/2964252-2964307	.AUUUAUAGCAUC-UAAUGGU..GCGAUAU................AUGCACAAUUCAAAGGAGAAUCAUGACAGA
NC_013203/420130-420185	.UUCAUUUCUUACAAAAAGAC..UUGUAG-................AAGUGAUCUGCUAUGAAGCAUAAAAUUUCA
NZ_AP017457/676069-676125	.ACUUACUUCAACAAAAUGGA..UUGUCCG................CGGUUCCGGGCAAAUUCGCCACUAGACUUU
NZ_CP007443/1784660-1784721	.AAAACGGCGACCGCAAAGCU..GCGGUUGcc...........guaACAGAUACGGCAAUGGGGCCGGAAAACCAG
NZ_CP009244/915923-915974	.UAUAACCUAUGGACAAAGAG..GUUGU--................---CAGUAGGCAAUGAAGCCGGUGCACAAC
NC_015738/2403432-2403484	.UCAAUGAUCCCAACAGUGAA..GUUGC--................--CAAAAAGGCAGCGAAGCUUCAAACGGAA
NZ_CP014513/1628135-1628207	.UGACGCCGAUUUACAACGAU..GUAAUAGucgauggagcacccacCCCUACGAAGCAAUGGAGCUCCAUGUCGAC
NC_013721/312045-312095	.AAGAUGAUUGCAGCGAAGAU..GCUGA--................----CGAAAGCAAAGAUGCUGAUAGCAAAA
NC_015564/4535773-4535832	.AAUGAACUAUCUACAACGAU..GUAGAAUu.............ggUGAACUCAUGCAAAGGAGCAUGCGAUGCCA
NZ_CP007155/7090364-7090418	.AAAAUGCAUUCGUGAACGAA..CACGAA-................-AAAUGAUCGCAAUGUAGCGGUUUUUGACA
NZ_CP012390/604046-604102	.AGGUUCUCGACCGCAUUGGU..GUGAUCG................UUGGACAGGGCAACGAUGCCAUGAACGACA
NZ_CP014761/2225592-2225649	.AUCAUGGUCUCUACAACGUU..GUAGUAC...............aACGUUGUAGACAAGGGUGUCACACACCGCG
NC_000962/2187261-2187311	.ACAAAUUCCCAUGCAAAGAA..GCACUU-................-----GUGUACAACGAAGUAUCUUGGUAGU
NZ_CP016779/284217-284271	.GAAAUAACUAAAUCAAUAUC..GGUUCC-................-AAUAAUUGGCAUUGGCGCCGGCAAUGAUU
NZ_CP017717/9630535-9630594	.GAUGCUCUGGCGGCGGUGAC..GCCGACAc.............guCAUUAAAAGGCAAUGGAGCCUGGGAAAAUU
NZ_CP016777/10943-11014	.AACAAAUUCUUAACAAGGCU..GUUGUCCacggcaa.aguaauuuUGUCUUCAGGCAAAGAGGCUGAUUACUACG
NZ_CP018002/1543107-1543167	.AUGAUUUCGGCAACAAUGGU..GUUGUG-.........AAAAACAUGACGGAGCUCGGAGCGGAGCGCCCCAA..
NC_011886/676358-676423	.AUAUUCGCAACCAGAAUGAC..CUGGGUCacga.......aagccGGAUUACAUGCGAAGGGGCAUCCAGCCAUG
NZ_CP012182/279030-279077	.AUCGAAUUUGCCCCACGGGU..GGGG---................------UAGGCAAGGACGCCGCGGAGCAGA
NC_010168/1485495-1485548	.CAGCAAAGACCGCCAAAGAG..GGCGA--................-UUUUGUGGGCAAGGAAGCCUUGGCAAAGA
NC_013521/337151-337201	.----------UUACAACGUU..GUAAGUGggauu.....cccugaAAGGACACCGCAAUGAAGCGCAU-------
NC_013165/1700444-1700501	.GUACGUGAAAGAGCAACGGU..GCUUUUU...............gAAGGCAAGAGCAAUGGUGGUUCUAUAAAAG
NC_013947/4871519-4871574	.UUGAAUUGAGUCGCAAAGGA..GCGAAG-................AUGUCUGUGGCCAAGUUGCCCAGUGCCGGU
NC_016582/1325572-1325634	.ACAAUAAAACUCACAAAGGU..GUUGUUUuau..........agcAUUGAGCAUGAAUCGGAUCACUCUGUGAAG
NZ_CP019606/2506764-2506818	ACUCUGAUAAUGA-CAGCGGUGUGAUU-..................-UGGACGUAGCAUGGGCGCUAUGUCUGAGG
NZ_CP012649/324715-324777	.AAAAAGCCGUAGCCAAAGCU..GUUUAUGcga..........aagGAAACAAGAACAAUGACGUUUUUGGUUAAC
RF	.AaaAaaaauagcACAAaGgc..GUgcUuu................uauuaaAaAGCAAuGaAGCUcauaaaaAaa
SS_cons	.::::::::::<<<<______..>>>>---................--------<<<______>>>::::::::::

Table S2: ANTAR-target RNAs identified with diffused search model using Infernal v1.0.2 are shown for each organism given with the genome accession ID. Bit score is the value assigned by the covariance search to indicate similarity to the search model. A higher score suggests that the predicted RNA is more similar to the search model. RNAs with bit score threshold≥15.00 are reported here. RNA co-ordinates are as predicted by Infernal. Genes in cis to the predicted RNA (gene linked to ANTAR-target RNA) is indicated as NCBI gene annotations and its corresponding distance from the RNAs.

Organism (Genome ID)	Bit score	ANTAR-target RNA co-ordinates	Strand	Annotation of the gene linked to ANTAR-target RNAs (Locus Tag)	Distance (nt) of RNA from gene linked to ANTAR-target RNA
Brevibacterium flavum strain ATCC 15168 (NZ_CP011309.1)	16.55	641856-641800	-1	UDP-glucose 4-epimerase GalE (YH66_RS03105)	64
	15.4	2690553-2690500	-1	phosphate ABC transporter, permease protein PstA (YH66_RS12975)	5
	15.31	1296793-1296849	1	DEAD/DEAH box helicase (YH66_RS06270)	7
	15.24	1195547-1195604	1	MFS transporter (YH66_RS05805)	43
Brevibacterium flavum ZL-1 (NZ_CP004046.1)	15.4	2724657-2724605	-1	phosphate ABC transporter, permease protein PstA (C628_RS12840)	47
	15.31	1333526-1333582	1	ATP-dependent RNA helicase (C628_RS06330)	7
	15.24	1235481-1235538	1	MFS transporter (C628_RS05870)	43
Acidimicrobium ferrooxidans DSM 10331 (NC_013124.1)	15.19	1724947-1725012	1	DNA-binding protein (AFER_RS08470)	42
Acidipropionibacterium acidipropionici ATCC 4875 (NC_019395.1)	19.36	2388629-2388576	-1	HTH domain-containing protein (PACID_RS11080)	19
	18.01	1188010-1187953	-1	TetR family transcriptional regulator (PACID_RS05275)	18
Acidipropionibacterium acidipropionici strain ATCC 55737 (NZ_CP014352.1)	19.36	963025-962972	-1	transcriptional regulator (AXH35_RS04620)	19
	19.36	2384398-2384451	1	transcriptional regulator (ASQ49_RS10630)	19

Organism (Genome ID)	Bit score	ANTAR-target RNA co-ordinates	Strand	Annotation of the gene linked to ANTAR-target RNAs (Locus Tag)	Distance (nt) of RNA from gene linked to ANTAR-target RNA
	18.01	3485203-3485260	1	TetR family transcriptional regulator (ASQ49_RS15825)	18
Acidipropionibacterium acidipropionici strain F3E8 (NZ_CP015970.1)	19.36	1261666-1261613	-1	HTH domain-containing protein (A8L58_RS06085)	19
	19.36	905699-905646	-1	Fe-S cluster assembly protein SufB (BWX38_RS04285)	9
	18.01	3442803-3442746	-1	ABC transporter ATP-binding protein (BWX38_RS15680)	602
Actinoalloteichus hymeniacidonis strain HPA177(T) (=DSM 45092(T)) (NZ_CP014859.1)	15.43	4943167-4943111	-1	YafY family transcriptional regulator (TL08_RS20635)	254
Actinoalloteichus sp. ADI127-7 (NZ_CP016076.1)	15.27	5692023-5692090	1	LysR family transcriptional regulator (UA74_RS24200)	17
Actinoalloteichus sp. GBA129-24 (NZ_CP016077.1)	15.27	5778466-5778533	1	LysR family transcriptional regulator (UA75_RS24715)	17
Actinobacteria bacterium IMCC25003 (NZ_CP015603.1)	16.63	195172-195101	-1	orotate phosphoribosyltransferase (IMCC25003_RS00950)	25
Actinobacteria bacterium IMCC26077 (NZ_CP015606.1)	15.46	213250-213181	-1	sugar ABC transporter ATP-binding protein (IMCC26077_RS01135)	98
Actinobacteria bacterium IMCC26103 (NZ_CP015604.1)	15.22	718815-718864	1	hypothetical protein (IMCC26103_RS03680)	41
Actinobacteria bacterium IMCC26256 (NZ_CP011489.1)	18.1	1875820-1875760	-1	hypothetical protein (IMCC26256_RS08800)	68
	16.24	1614744-1614691	-1	HNH endonuclease (IMCC26256_RS07585)	43
	15.67	878644-878705	1	hypothetical protein (IMCC26256_RS04175)	86
Actinomyces gaoshouyii strain	19.82	414788-414732	-1	MFS transporter (B6G06_RS01630)	11

Organism (Genome ID)	Bit score	ANTAR-target RNA co-ordinates	Strand	Annotation of the gene linked to ANTAR-target RNAs (Locus Tag)	Distance (nt) of RNA from gene linked to ANTAR-target RNA
pika_114 (NZ_CP020468.1)	16.29	646951-647003	1	sugar ABC transporter substrate-binding protein (B6G06_RS02565)	14
	15.63	2104690-2104738	1	serine/threonine protein kinase (B6G06_RS08790)	17
Actinomyces meyeri strain W712 (NZ_CP012072.1)	17.3	276999-277055	1	glutamine synthetase (ADJ76_RS01245)	3
Actinomyces sp. Chiba101 DNA (NZ_AP017896.1)	16.32	593765-593708	-1	MFS transporter (CHIBA101_RS02465)	10
	15.67	773606-773660	1	2-keto-myo-inositol dehydratase (CHIBA101_RS03265)	4
	15.63	313776-313728	-1	serine/threonine protein kinase (CHIBA101_RS01290)	17
	15.25	2027467-2027415	-1	sugar ABC transporter substrate-binding protein (CHIBA101_RS08425)	14
Actinomyces sp. Marseille-P2985 strain Marseille-P2985T contig00001 (NZ_LT635457.1)	15.2	581940-581874	-1	alpha-mannosidase (BQ7111_RS02600)	13
Actinomyces sp. oral taxon 414 strain F0588 (NZ_CP012590.1)	24.28	627231-627295	1	LacI family transcriptional regulator (AM609_RS02480)	91
Actinomyces sp. VUL4_3 (NZ_CP017812.1)	21.6	1397314-1397258	-1	recombinase RecA (BK816_RS05805)	79
	17.19	254992-255054	1	phosphoribosylformylglycinamidine synthase subunit PurL (BK816_RS00900)	477
	15.89	2085482-2085428	-1	hypothetical protein (BK816_RS08520)	83
Actinoplanes friuliensis DSM 7358 (NC_022657.1)	18.23	9254943-9254997	1	hypothetical protein (AFR_RS42690)	5
	15.3	7354175-7354127	-1	alpha-1,2-mannosidase (AFR_RS33845)	10
Actinoplanes missouriensis 431 DNA (NC_017093.1)	15.82	7097523-7097475	-1	tryptophan 2,3-dioxygenase (AMIS_RS32425)	6
	15.42	7744888-7744823	-1	electron transfer flavoprotein subunit beta	19

Organism (Genome ID)	Bit score	ANTAR-target RNA co-ordinates	Strand	Annotation of the gene linked to ANTAR-target RNAs (Locus Tag)	Distance (nt) of RNA from gene linked to ANTAR-target RNA
				(AMIS_RS35245)	
Actinoplanes sp. N902-109 (NC_021191.1)	15.77	1672608-1672658	1	ribosome silencing factor (L083_RS07555)	53
Actinosynnema pretiosum strain X47 (NZ_CP023445.1)	20.39	4013130-4013182	1	LacI family transcriptional regulator (CNX65_RS17145)	17
	17.87	3895160-3895225	1	hypothetical protein (CNX65_RS16655)	2
Actinotignum schaalii strain CCUG 27420 (NZ_CP008802.1)	21.11	1251722-1251774	1	LacI family transcriptional regulator (FB03_RS05325)	2
	15.9	1434577-1434519	-1	hypothetical protein (FB03_RS06045)	209
	15.18	1274459-1274527	1	LuxR family transcriptional regulator (FB03_RS05415)	356
Adlercreutzia equolifaciens DSM 19450 DNA (NC_022567.1)	32.04	1325349-1325289	-1	tRNA-Pro (AEQU_RS05195)	15
	18.26	1426207-1426157	-1	3-methyl-2-oxobutanoate dehydrogenase subunit VorB (AEQU_RS05670)	5
Aeromicrobium erythreum strain AR18 (NZ_CP011502.1)	15.47	3448673-3448731	1	NAD(P)/FAD-dependent oxidoreductase (Aeryth_RS16260)	3
Agromyces aureus strain AR33 (NZ_CP013979.1)	16.26	3880103-3880043	-1	hypothetical protein (ATC03_RS17315)	67
Amycolatopsis japonica strain MG417-CF17 (NZ_CP008953.1)	23.53	3466279-3466226	-1	TetR/AcrR family transcriptional regulator (AJAP_RS16240)	12
	16.49	1154197-1154251	1	hypothetical protein (AJAP_RS05720)	26
Amycolatopsis keratiniphila strain HCCB10007 (NC_021252.1)	15.72	7469565-7469511	-1	DUF385 domain-containing protein (AORI_RS74380)	26
	15.68	4847596-4847645	1	helix-turn-helix domain-containing protein (AORI_RS63105)	120
Amycolatopsis mediterranei RB (NC_022116.1)	16.07	9478426-9478370	-1	gas vesicle protein (B737_RS43515)	37

Organism (Genome ID)	Bit score	ANTAR-target RNA co-ordinates	Strand	Annotation of the gene linked to ANTAR-target RNAs (Locus Tag)	Distance (nt) of RNA from gene linked to ANTAR-target RNA
Amycolatopsis mediterranei S699 (NC_017186.1)	16.23	6403001-6403057	1	IS110 family transposase (RAM_RS48360)	213
	16.07	9468570-9468514	-1	gas vesicle protein (RAM_RS43460)	37
	16.23	6412928-6412984	1	IS110 family transposase (AMES_RS48400)	213
	16.07	9478482-9478426	-1	gas vesicle protein (AMES_RS43515)	37
Amycolatopsis mediterranei U32 (NC_014318.1)	16.07	9468510-9468454	-1	gas vesicle protein GvpA (AMED_8633)	37
Amycolatopsis orientalis strain B-37 (NZ_CP016174.1)	15.11	3249576-3249634	1	shikimate dehydrogenase (SD37_RS14565)	36
	15.02	7928691-7928746	1	tripartite tricarboxylate transporter TctB family protein (SD37_RS34325)	9
Arcanobacterium haemolyticum DSM 20595 (NC_014218.1)	26.85	792299-792362	1	galactose/glucose-binding lipoprotein (ARCH_RS03540)	8
	22.68	625569-625621	1	hexose-6-phosphate:phosphate antiporter (ARCH_RS02785)	1
	19.77	40752-40799	1	exo-alpha-sialidase (ARCH_RS09165)	4
	17.2	88902-88960	1	glycerophosphoryl diester phosphodiesterase (ARCH_RS00310)	0
	17.2	704515-704578	1	hypothetical protein (ARCH_RS03150)	36
	16.57	123085-123135	1	ATP-binding protein (ARCH_RS00470)	2
Arsenicicoccus sp. oral taxon 190 (NZ_CP012070.1)	15.63	2479486-2479551	1	30S ribosomal protein S1 (ADJ73_RS11475)	91
Arthrobacter alpinus strain ERGS4:06 (NZ_CP013200.1)	23.49	2964252-2964307	1	alpha/beta hydrolase (AS189_RS13165)	7
	19.15	2477270-2477335	1	30S ribosomal protein S1 (AS189_RS10870)	49
	17.81	1608213-1608269	1	hypothetical protein (AS189_RS07130)	3

Organism (Genome ID)	Bit score	ANTAR-target RNA co-ordinates	Strand	Annotation of the gene linked to ANTAR-target RNAs (Locus Tag)	Distance (nt) of RNA from gene linked to ANTAR-target RNA
	15.34	3870920-3870873	-1	DNA alkylation repair protein (AS189_RS17180)	239
	15.31	1273631-1273692	1	heavy metal translocating P-type ATPase (AS189_RS05655)	17
	15.25	4027396-4027453	1	hypothetical protein (AS189_RS17870)	345
	15.21	473061-473110	1	DUF3427 domain-containing protein (AOC05_RS02030)	5
	15.1	125174-125110	-1	ABC transporter substrate-binding protein (AOC05_RS00465)	72
Arthrobacter arilaitensis RE117 (NC_014550.1)	20.23	976792-976738	-1	acyl-CoA carboxylase subunit beta (AARI_RS04880)	351
	15.82	3030192-3030140	-1	amino acid decarboxylase (AARI_RS14455)	35
Arthrobacter sp. ATCC 21022 (NZ_CP014196.1)	18.98	506425-506490	1	hypothetical protein (AUT26_RS02425)	17
	17.62	523749-523796	1	MFS transporter (AUT26_RS02490)	5
	17.11	549492-549436	-1	oxidoreductase (AUT26_RS02610)	80
	16.98	2015651-2015589	-1	sugar ABC transporter substrate-binding protein (AUT26_RS09085)	3
	16.04	2275221-2275286	1	30S ribosomal protein S1 (AUT26_RS10230)	94
	15.76	4370883-4370828	-1	AUT26_RS20010 (AUT26_RS20010)	47
	15.14	3630293-3630349	1	hypothetical protein (AUT26_RS16410)	11
Arthrobacter sp. ERGS1:01 isolate water (NZ_CP012479.1)	17.89	1014978-1015039	1	hypothetical protein (AL755_RS08425)	2
	15.86	386851-386793	-1	DUF4245 domain-containing protein (AL755_RS05765)	209
	15.38	3422948-3422884	-1	universal stress protein (AL755_RS19430)	25

Organism (Genome ID)	Bit score	ANTAR-target RNA co-ordinates	Strand	Annotation of the gene linked to ANTAR-target RNAs (Locus Tag)	Distance (nt) of RNA from gene linked to ANTAR-target RNA
	15.26	98837-98904	1	aldehyde dehydrogenase (AL755_RS04450)	107
Arthrobacter sp. ERGS1:01 isolate water plasmid unnamed2 (NZ_CP012477.1)	19.54	315774-315838	1	ABC transporter substrate-binding protein (AL755_RS01495)	17
	16.68	285368-285421	1	ABC transporter ATP-binding protein (AL755_RS01335)	31
Arthrobacter sp. FB24 plasmid 2 (NC_008538.1)	18.43	84098-84153	1	ferredoxin (ARTH_RS22340)	17
Arthrobacter sp. FB24 (NC_008541.1)	24.08	399351-399411	1	hypothetical protein (ARTH_RS01920)	1
	23	3967947-3968005	1	gluconate kinase (ARTH_RS17885)	17
	15.34	2327690-2327755	1	30S ribosomal protein S1 (ARTH_RS10440)	94
Arthrobacter sp. IHBB 11108 (NZ_CP011005.1)	19.22	1447414-1447470	1	amino acid permease (UM93_RS06735)	280
	18.47	507759-507816	1	death-on-curing protein (UM93_RS02365)	178
	16.97	2933785-2933728	-1	hypothetical protein (UM93_RS13745)	25
	15.36	3142426-3142370	-1	long-chain-fatty-acid--CoA ligase (UM93_RS14870)	5
Arthrobacter sp. LS16 (NZ_CP012171.1)	19.15	1710406-1710341	-1	glyceraldehyde-3-phosphate dehydrogenase (AFL94_RS07635)	50
Arthrobacter sp. QXT-31 (NZ_CP019304.1)	19.08	3170621-3170678	1	ABC transporter substrate-binding protein (BWQ92_RS14325)	2
	18.85	4938603-4938520	-1	AcrB/AcrD/AcrF family protein (BWQ92_RS22540)	19
	17.46	4972446-4972392	-1	sugar ABC transporter substrate-binding protein (BWQ92_RS22690)	66
	17.43	1250002-1250061	1	hypothetical protein (BWQ92_RS05620)	28
	15.41	2070139-2070197	1	gfo/Idh/MocA family oxidoreductase	44

Organism (Genome ID)	Bit score	ANTAR-target RNA co-ordinates	Strand	Annotation of the gene linked to ANTAR-target RNAs (Locus Tag)	Distance (nt) of RNA from gene linked to ANTAR-target RNA
				(BWQ92_RS09390)	
Arthrobacter sp. Rue61a (NC_018531.1)	18.16	2363737-2363795	1	sugar ABC transporter substrate-binding protein (ARUE_RS10720)	88
	17.57	648665-648604	-1	MFS transporter (ARUE_RS03145)	83
	15.44	4204106-4204050	-1	LacI family transcriptional regulator (ARUE_RS19180)	420
	15.34	2452695-2452760	1	30S ribosomal protein S1 (ARUE_RS11125)	94
Arthrobacter sp. U41 (NZ_CP015732.1)	20.48	54483-54428	-1	PLP-dependent transferase (ASPU41_RS00255)	4
	16.64	1628239-1628298	1	hypothetical protein (ASPU41_RS07570)	2
	15.96	3306175-3306228	1	LacI family DNA-binding transcriptional regulator (ASPU41_RS15065)	17
	15.85	1748515-1748566	1	acetone carboxylase subunit alpha (ASPU41_RS08160)	17
	15.54	3982498-3982554	1	hypothetical protein (ASPU41_RS18175)	48
	15.27	3740542-3740604	1	CsbD family protein (ASPU41_RS17020)	10
Arthrobacter sp. YC-RL1 (NZ_CP013297.1)	19.15	2161698-2161633	-1	glyceraldehyde-3-phosphate dehydrogenase (ATC04_RS10120)	50
Arthrobacter sp. YN (NZ_CP022436.1)	21.09	2248139-2248195	1	FAD-dependent oxidoreductase (CGK93_RS10185)	23
	15.96	1910566-1910516	-1	sensor histidine kinase (CGK93_RS08610)	359
	15.88	4224508-4224564	1	hypothetical protein (CGK93_RS19300)	11
	15.34	2675478-2675543	1	30S ribosomal protein S1 (CGK93_RS12080)	94
Arthrobacter sp. ZXY-2	17.62	3552929-3552882	-1	MFS transporter (ARZXY2_RS16210)	5

Organism (Genome ID)	Bit score	ANTAR-target RNA co-ordinates	Strand	Annotation of the gene linked to ANTAR-target RNAs (Locus Tag)	Distance (nt) of RNA from gene linked to ANTAR-target RNA
(NZ_CP017421.1)	17.11	3527274-3527330	1	oxidoreductase (ARZXY2_RS16090)	80
	16.04	1988906-1988841	-1	30S ribosomal protein S1 (ARZXY2_RS09175)	94
	15.76	4114296-4114351	1	MFS transporter (ARZXY2_RS18885)	8
	15.75	304935-304879	-1	hypothetical protein (ARZXY2_RS01460)	11
	15.05	3269070-3269014	-1	DNA-binding response regulator (ARZXY2_RS14910)	64
Arthrobacter sp. ZXY-2 plasmid pZXY21 (NZ_CP017422.1)	15.66	154094-154033	-1	FAD-binding oxidoreductase (ARZXY2_RS21385)	2
Atopobium parvulum DSM 20469 (NC_013203.1)	19.43	420185-420130	-1	hypothetical protein (APAR_RS01890)	17
	16.32	432964-433017	1	hypothetical protein (APAR_RS01945)	26
Aurantimicrobium minutum DNA, strain: KNC (NZ_AP017457.1)	19.38	676125-676069	-1	hypothetical protein (AUMI_RS03320)	28
	16.5	1187659-1187715	1	NINE protein (AUMI_RS05890)	64
	16.44	1240808-1240861	1	type I pantothenate kinase (AUMI_RS06085)	330
	15.75	521488-521549	1	ammonium transporter (AUMI_RS02580)	2
	15.3	749840-749893	1	propionyl-CoA synthetase (AUMI_RS03660)	2
Bifidobacterium adolescentis ATCC 15703 DNA (NC_008618.1)	21.38	1609525-1609471	-1	sugar ABC transporter permease (BAD_RS06830)	5
	16.01	534675-534736	1	sugar ABC transporter substrate-binding protein (BAD_RS02255)	38
	15.16	1278132-1278189	1	mannan endo-1,4-beta-mannosidase (BAD_RS05480)	4
Bifidobacterium adolescentis strain 22L (NZ_CP007443.1)	27.95	1784660-1784721	1	HAD family hydrolase (BADO_RS07460)	48
	21.86	2169133-2169079	-1	ABC transporter substrate-binding protein	5

Organism (Genome ID)	Bit score	ANTAR-target RNA co-ordinates	Strand	Annotation of the gene linked to ANTAR-target RNAs (Locus Tag)	Distance (nt) of RNA from gene linked to ANTAR-target RNA
				(BADO_RS08855)	
	16.14	542863-542924	1	sugar ABC transporter substrate-binding protein (BADO_RS02270)	38
	15.88	217067-217115	1	hypothetical protein (BADO_RS00830)	71
Bifidobacterium adolescentis strain BBMN23 (NZ_CP010437.1)	25.23	2041274-2041213	-1	HAD family hydrolase (BBMN23_RS08555)	14
	17.56	431761-431813	1	MFS transporter (BBMN23_RS01715)	13
	17.47	550782-550840	1	sugar ABC transporter substrate-binding protein (BBMN23_RS02285)	38
	15.88	227134-227182	1	hypothetical protein (BBMN23_RS00850)	71
Bifidobacterium angulatum DSM 20098 = JCM 7096 DNA (NZ_AP012322.1)	20.47	1863117-1863064	-1	DUF2142 domain-containing protein (BBAG_RS07490)	17
	20	787294-787346	1	recombinase RecA (BBAG_RS03180)	40
	16.36	1197777-1197844	1	YraN family protein (BBAG_RS04860)	132
	16.14	439087-439148	1	sugar ABC transporter substrate-binding protein (BBAG_RS01745)	38
	15.61	854184-854242	1	D-tyrosyl-tRNA(Tyr) deacylase (BBAG_RS03410)	147
	15.37	606590-606650	1	ABC transporter substrate-binding protein (BBAG_RS02440)	17
	15.15	565546-565592	1	sugar ABC transporter permease (BBAG_RS02280)	17
Bifidobacterium angulatum strain GT102 (NZ_CP014241.1)	20	1353009-1353061	1	recombinase RecA (Bang102_RS05340)	40
	16.14	951500-951561	1	sugar ABC transporter substrate-binding protein (Bang102_RS03735)	38
	15.59	1152771-1152827	1	MFS transporter (Bang102_RS04535)	93

Organism (Genome ID)	Bit score	ANTAR-target RNA co-ordinates	Strand	Annotation of the gene linked to ANTAR-target RNAs (Locus Tag)	Distance (nt) of RNA from gene linked to ANTAR-target RNA
	15.15	1122454-1122500	1	sugar ABC transporter permease (Bang102_RS04410)	17
Bifidobacterium animalis strain A6 (NZ_CP010433.1)	17.58	1704615-1704565	-1	mannan endo-1,4-beta-mannosidase (BAA6_RS07205)	12
	17.28	1662665-1662716	1	hypothetical protein (BAA6_RS07010)	1
	16.16	357064-357123	1	mannose-1-phosphate guanylyltransferase (BAA6_RS01455)	176
	16.16	357063-357123	1	mannose-1-phosphate guanylyltransferase (BAA6_RS01455)	176
Bifidobacterium animalis strain BL3 (NZ_CP017098.1)	17.58	1704823-1704773	-1	mannan endo-1,4-beta-mannosidase (BGL50_RS07385)	12
	17.28	1662873-1662924	1	hypothetical protein (BGL50_RS07190)	1
	16.16	357118-357177	1	mannose-1-phosphate guanylyltransferase (BGL50_RS01545)	176
Bifidobacterium animalis strain RH (NZ_CP007755.1)	17.58	1693954-1693904	-1	mannan endo-1,4-beta-mannosidase (EN10_RS07170)	12
	17.28	1652004-1652055	1	hypothetical protein (EN10_RS06975)	1
	16.16	357421-357480	1	mannose-1-phosphate guanylyltransferase (EN10_RS01455)	176
Bifidobacterium animalis subsp. animalis ATCC 25527 (NC_017834.1)	17.61	1905476-1905417	-1	sn-glycerol-3-phosphate ABC transporter ATP-binding protein UgpC (BANAN_RS07810)	77
	16.47	363482-363548	1	mannose-1-phosphate guanylyltransferase (BANAN_RS01505)	176
	16.18	573407-573353	-1	hypothetical protein (BANAN_RS08175)	89
	16.07	1890249-1890201	-1	alpha-galactosidase (BANAN_RS07760)	7
	15.35	1206973-1206922	-1	hypothetical protein (BANAN_RS08255)	228

Organism (Genome ID)	Bit score	ANTAR-target RNA co-ordinates	Strand	Annotation of the gene linked to ANTAR-target RNAs (Locus Tag)	Distance (nt) of RNA from gene linked to ANTAR-target RNA
Bifidobacterium animalis subsp. animalis strain YL2 (NZ_CP015407.2)	18.25	352928-352862	-1	mannose-1-phosphate guanylyltransferase (A4U98_RS01585)	176
	17.61	750467-750526	1	sn-glycerol-3-phosphate ABC transporter ATP-binding protein UgpC (A4U98_RS03285)	77
	17.28	1036659-1036608	-1	hypothetical protein (A4U98_RS04475)	1
	16.18	148239-148293	1	hypothetical protein (A4U98_RS00670)	89
	16.07	765693-765741	1	alpha-galactosidase (A4U98_RS03340)	7
	16.07	894076-894146	1	nucleoside hydrolase (A4U98_RS03835)	6
	15.35	1538414-1538465	1	hypothetical protein (A4U98_RS06450)	228
Bifidobacterium animalis subsp. lactis AD011 (NC_011835.1)	17.58	830093-830043	-1	mannan endo-1,4-beta-mannosidase (BLA_RS03390)	12
	17.28	788300-788351	1	hypothetical protein (BLA_RS03195)	1
	16.16	356849-356908	1	mannose-1-phosphate guanylyltransferase (BLA_RS01455)	176
Bifidobacterium animalis subsp. lactis ATCC 27673 (NC_022523.1)	18.72	1727752-1727699	-1	secreted beta-mannosidase (BLAC_RS07285)	30
	17.28	1678573-1678624	1	hypothetical protein (BLAC_RS07090)	1
	16.16	357221-357280	1	mannose-1-phosphate guanylyltransferase (BLAC_RS01485)	176
Bifidobacterium animalis subsp. lactis B420 (NC_017866.1)	17.58	1699160-1699110	-1	mannan endo-1,4-beta-mannosidase (W7Y_RS07195)	12
	17.28	1657210-1657261	1	hypothetical protein (W7Y_RS07000)	1
	16.16	357061-357120	1	mannose-1-phosphate guanylyltransferase (W7Y_RS01460)	176
Bifidobacterium animalis subsp. lactis BB-12 (NC_017214.1)	17.58	396461-396411	-1	mannan endo-1,4-beta-mannosidase (BIF_RS01600)	12

Organism (Genome ID)	Bit score	ANTAR-target RNA co-ordinates	Strand	Annotation of the gene linked to ANTAR-target RNAs (Locus Tag)	Distance (nt) of RNA from gene linked to ANTAR-target RNA
	17.28	354579-354630	1	hypothetical protein (BIF_RS01405)	1
	16.16	992545-992604	1	mannose-1-phosphate guanylyltransferase (BIF_RS03905)	176
Bifidobacterium animalis subsp. lactis Bi-07 (NC_017867.1)	17.58	1699379-1699329	-1	mannan endo-1,4-beta-mannosidase (W91_RS07185)	12
	17.28	1657429-1657480	1	hypothetical protein (W91_RS06990)	1
	16.16	357063-357122	1	mannose-1-phosphate guanylyltransferase (W91_RS01455)	176
Bifidobacterium animalis subsp. lactis Bl-04 (NC_012814.1)	17.58	1699324-1699274	-1	mannan endo-1,4-beta-mannosidase (BALAC_RS07185)	12
	17.28	1657373-1657424	1	hypothetical protein (BALAC_RS06990)	1
	16.16	357062-357121	1	mannose-1-phosphate guanylyltransferase (BALAC_RS01455)	176
Bifidobacterium animalis subsp. lactis Bl12 (NC_021593.1)	17.58	1698948-1698898	-1	mannan endo-1,4-beta-mannosidase (BL12_RS07185)	12
	17.28	1656999-1657050	1	hypothetical protein (BL12_RS06990)	1
	16.16	356921-356980	1	mannose-1-phosphate guanylyltransferase (BL12_RS01455)	176
Bifidobacterium animalis subsp. lactis BLC1 (NC_017216.2)	17.58	1698969-1698919	-1	mannan endo-1,4-beta-mannosidase (BLC1_RS07185)	12
	17.28	1657019-1657070	1	hypothetical protein (BLC1_RS06990)	1
	16.16	356927-356986	1	mannose-1-phosphate guanylyltransferase (BLC1_RS01455)	176
Bifidobacterium animalis subsp. lactis CNCM I-2494 (NC_017215.1)	17.58	1703911-1703861	-1	mannan endo-1,4-beta-mannosidase (BALAC2494_RS07200)	12
	17.28	1661994-1662045	1	hypothetical protein (BALAC2494_RS07005)	1

Organism (Genome ID)	Bit score	ANTAR-target RNA co-ordinates	Strand	Annotation of the gene linked to ANTAR-target RNAs (Locus Tag)	Distance (nt) of RNA from gene linked to ANTAR-target RNA
	16.16	357074-357133	1	mannose-1-phosphate guanylyltransferase (BALAC2494_RS01455)	176
Bifidobacterium animalis subsp. lactis DSM 10140 (NC_012815.1)	17.58	1699041-1698991	-1	mannan endo-1,4-beta-mannosidase (BALAT_RS07180)	12
	17.28	1657091-1657142	1	hypothetical protein (BALAT_RS06985)	1
	16.16	357063-357122	1	mannose-1-phosphate guanylyltransferase (BALAT_RS01455)	176
Bifidobacterium animalis subsp. lactis KLDS2.0603 (NZ_CP007522.1)	17.58	1698786-1698736	-1	mannan endo-1,4-beta-mannosidase (U723_RS07185)	12
	17.28	1656836-1656887	1	hypothetical protein (U723_RS06990)	1
	16.16	356896-356955	1	mannose-1-phosphate guanylyltransferase (U723_RS01450)	176
Bifidobacterium animalis subsp. lactis strain BF052 (NZ_CP009045.1)	17.58	1698958-1698908	-1	mannan endo-1,4-beta-mannosidase (GU89_RS07185)	12
	17.28	1657008-1657059	1	hypothetical protein (GU89_RS06990)	1
	16.16	356897-356956	1	mannose-1-phosphate guanylyltransferase (GU89_RS01450)	176
Bifidobacterium animalis subsp. lactis V9 (NC_017217.1)	17.58	1704610-1704560	-1	mannan endo-1,4-beta-mannosidase (BALV_RS07195)	12
	17.28	1662660-1662711	1	hypothetical protein (BALV_RS07000)	1
	16.16	357064-357123	1	mannose-1-phosphate guanylyltransferase (BALV_RS01455)	176
	16.16	357063-357123	1	mannose-1-phosphate guanylyltransferase (BAA6_RS01455)	176
Bifidobacterium asteroides PRL2011 (NC_018720.1)	18.66	1405400-1405348	-1	MFS transporter (BAST_RS05485)	9
	16.49	389675-389732	1	NAD(P)/FAD-dependent oxidoreductase	79

Organism (Genome ID)	Bit score	ANTAR-target RNA co-ordinates	Strand	Annotation of the gene linked to ANTAR-target RNAs (Locus Tag)	Distance (nt) of RNA from gene linked to ANTAR-target RNA
				(BAST_RS01530)	
	15.55	2013830-2013775	-1	type II toxin-antitoxin system antitoxin, RelB/DinJ family (BAST_RS08055)	62
Bifidobacterium asteroides strain DSM 20089 (NZ_CP017696.1)	18.66	1186153-1186101	-1	MFS transporter (BA20089_RS04650)	87
	16.49	170394-170451	1	NAD(P)/FAD-dependent oxidoreductase (BA20089_RS00705)	79
	15.55	1794587-1794532	-1	type II toxin-antitoxin system antitoxin, RelB/DinJ family (BA20089_RS07265)	62
Bifidobacterium bifidum ATCC 29521 = JCM 1255 = DSM 20456 DNA (NZ_AP012323.1)	18.3	63952-63901	-1	IS256 family transposase (BBBF_RS00250)	27
	17.02	1140470-1140529	1	PadR family transcriptional regulator (BBBF_RS04740)	103
	16.89	686128-686184	1	LysM peptidoglycan-binding domain-containing protein (BBBF_RS02700)	105
	16.42	1744975-1744913	-1	thymidylate synthase (BBBF_RS07360)	102
Bifidobacterium bifidum BGN4 (NC_017999.1)	18.45	647226-647282	1	LysM peptidoglycan-binding domain-containing protein (BBB_RS02605)	105
	17.02	1082856-1082915	1	PadR family transcriptional regulator (BBB_RS04385)	103
	16.42	1733401-1733339	-1	thymidylate synthase (BBB_RS07205)	102
Bifidobacterium bifidum PRL2010 (NC_014638.1)	17.02	1134880-1134939	1	family transcriptional regulator PadR (BBPR_0951)	103
	16.89	681907-681963	1	hypothetical protein (BBPR_0543)	105
	16.42	1740876-1740814	-1	thymidylate synthase (BBPR_1477)	102
Bifidobacterium bifidum S17 (NC_014616.1)	18.3	480016-480067	1	IS256 family transposase (BBIF_RS02050)	27
	17.02	1104648-1104707	1	PadR family transcriptional regulator	103

Organism (Genome ID)	Bit score	ANTAR-target RNA co-ordinates	Strand	Annotation of the gene linked to ANTAR-target RNAs (Locus Tag)	Distance (nt) of RNA from gene linked to ANTAR-target RNA
				(BBIF_RS04590)	
	16.42	1713278-1713216	-1	thymidylate synthase (BBIF_RS07240)	102
Bifidobacterium bifidum strain BF3 (NZ_CP010412.1)	17.02	1159989-1160048	1	PadR family transcriptional regulator (RY70_RS04590)	103
	16.42	1806969-1806907	-1	thymidylate synthase (RY70_RS07425)	102
	15.09	732893-732949	1	LysM peptidoglycan-binding domain-containing protein (RY70_RS02830)	105
Bifidobacterium breve 12L (NZ_CP006711.1)	20.6	1516065-1516101	1	serpin family protein (B12L_RS06350)	31
	17.59	775796-775729	-1	Txe/YoeB family addiction module toxin (B12L_RS03070)	59
	16.26	1347579-1347527	-1	recombinase RecA (B12L_RS05660)	55
Bifidobacterium breve 689b (NZ_CP006715.1)	20.83	665275-665329	1	hypothetical protein (B689B_RS02670)	8
	20.6	1592334-1592390	1	serpin family protein (B689B_RS06770)	41
	17.59	872602-872535	-1	Txe/YoeB family addiction module toxin (B689B_RS03565)	59
	17.2	602556-602495	-1	ATPase AAA (B689B_RS02450)	1066
	16.38	258222-258273	1	IS3 family transposase (B689B_RS00960)	183
	16.26	1421977-1421925	-1	recombinase RecA (B689B_RS06075)	55
Bifidobacterium breve ACS-071-V-Sch8b (NC_017218.1)	20.6	652555-652499	-1	serpin family protein (HMPREF9228_RS02650)	5
	17.59	1334083-1334150	1	Txe/YoeB family addiction module toxin (HMPREF9228_RS05665)	59
	17.2	1591103-1591164	1	ATPase AAA (HMPREF9228_RS06745)	1066
	16.38	282198-282249	1	IS3 family transposase (HMPREF9228_RS01080)	183
	16.26	822879-822931	1	recombinase RecA	55

Organism (Genome ID)	Bit score	ANTAR-target RNA co-ordinates	Strand	Annotation of the gene linked to ANTAR-target RNAs (Locus Tag)	Distance (nt) of RNA from gene linked to ANTAR-target RNA
				(HMPREF9228_RS03350)	
Bifidobacterium breve DSM 20213 = JCM 1192 DNA (NZ_AP012324.1)	20.6	1590628-1590684	1	serpin family protein (BBBR_RS06980)	41
	17.59	788421-788354	-1	Txe/YoeB family addiction module toxin (BBBR_RS03210)	59
	17.2	548890-548829	-1	ATPase AAA (BBBR_RS02190)	1066
	16.26	1382408-1382356	-1	recombinase RecA (BBBR_RS05995)	55
Bifidobacterium breve JCM 7017 (NZ_CP006712.1)	20.6	1542383-1542439	1	serpin family protein (B7017_RS06485)	41
	18.02	578576-578515	-1	ATPase AAA (B7017_RS02265)	7
	17.59	816831-816764	-1	Txe/YoeB family addiction module toxin (B7017_RS03260)	59
	16.26	1371305-1371253	-1	recombinase RecA (B7017_RS05775)	55
Bifidobacterium breve JCM 7019 (NZ_CP006713.1)	21.11	1022299-1022374	1	sugar ABC transporter permease (B7019_RS04300)	18
	20.6	1627645-1627701	1	serpin family protein (B7019_RS07170)	41
	18.3	481305-481356	1	IS256 family transposase (B7019_RS02000)	27
	17.67	560496-560435	-1	ATPase AAA (B7019_RS02290)	7
	17.08	2153758-2153815	1	hypothetical protein (B7019_RS09445)	51
	16.26	1449520-1449468	-1	recombinase RecA (B7019_RS06425)	55
Bifidobacterium breve NCFB 2258 (NZ_CP006714.1)	20.6	1543740-1543796	1	serpin family protein (B2258_RS06500)	41
	17.59	819838-819771	-1	Txe/YoeB family addiction module toxin (B2258_RS03295)	59
	17.2	580193-580132	-1	ATPase AAA (B2258_RS02275)	1066
	16.38	253813-253864	1	IS3 family transposase (B2258_RS00940)	183
	16.26	1371275-1371223	-1	DNA recombination/repair protein RecA	55

Organism (Genome ID)	Bit score	ANTAR-target RNA co-ordinates	Strand	Annotation of the gene linked to ANTAR-target RNAs (Locus Tag)	Distance (nt) of RNA from gene linked to ANTAR-target RNA
				(B2258_RS05785)	
Bifidobacterium breve S27 (NZ_CP006716.1)	20.6	1587761-1587817	1	serpin family protein (BS27_RS06675)	41
	17.59	860748-860681	-1	Txe/YoeB family addiction module toxin (BS27_RS03455)	59
	17.2	624084-624023	-1	ATPase AAA (BS27_RS02455)	1066
	16.38	285280-285331	1	IS3 family transposase (BS27_RS01055)	183
	16.26	1420569-1420517	-1	recombinase RecA (BS27_RS05990)	55
Bifidobacterium breve strain BR3 (NZ_CP010413.1)	20.6	521221-521165	-1	serpin family protein (RY69_RS02295)	41
	17.2	1598844-1598905	1	ATPase AAA (RY69_RS07155)	1066
	16.13	1051877-1051923	1	hypothetical protein (RY69_RS04810)	208
	16.01	681548-681600	1	recombinase RecA (RY69_RS02960)	55
Bifidobacterium breve strain LMC520 (NZ_CP019596.1)	20.6	1647336-1647392	1	serpin family protein (BBL520_RS07465)	41
	17.2	611214-611153	-1	ATPase AAA (BBL520_RS02640)	1066
	16.13	1120327-1120281	-1	hypothetical protein (BBL520_RS04915)	208
	16.01	1476706-1476654	-1	recombinase RecA (BBL520_RS06735)	55
Bifidobacterium breve UCC2003 (NC_020517.1)	21.11	419361-419436	1	sugar ABC transporter permease (BBR_RS11870)	18
	20.6	1647321-1647377	1	serpin family protein (BBR_RS17245)	41
	18.17	396084-396033	-1	IS256 family transposase (BBR_RS11755)	27
	17.59	907425-907358	-1	Txe/YoeB family addiction module toxin (BBR_RS13945)	59
	17.2	673882-673821	-1	ATPase AAA (BBR_RS12970)	1066
	16.38	300568-300619	1	IS3 family transposase (BBR_RS11370)	183
	16.26	1476776-1476724	-1	recombinase RecA (BBR_RS16530)	55

Organism (Genome ID)	Bit score	ANTAR-target RNA co-ordinates	Strand	Annotation of the gene linked to ANTAR-target RNAs (Locus Tag)	Distance (nt) of RNA from gene linked to ANTAR-target RNA
	16.13	1198206-1198160	-1	hypothetical protein (BBR_RS15265)	208
Bifidobacterium catenulatum DSM 16992 = JCM 1194 = LMG 11043 DNA (NZ_AP012325.1)	21.82	603559-603619	1	hypothetical protein (BBCT_RS02540)	11
	20.47	1858333-1858280	-1	DUF2142 domain-containing protein (BBCT_RS07800)	17
	18.36	1553523-1553467	-1	MFS transporter (BBCT_RS06595)	54
	17.79	279728-279782	1	50S ribosomal protein L19 (BBCT_RS01090)	358
	17.56	392162-392214	1	MFS transporter (BBCT_RS01550)	13
	16.14	500565-500623	1	sugar ABC transporter substrate-binding protein (BBCT_RS02095)	38
Bifidobacterium coryneforme strain LMG18911 (NZ_CP007287.1)	23.63	1150582-1150528	-1	MFS transporter (bcor_RS04490)	46
	16.33	1691827-1691880	1	VOC family protein (bcor_RS06865)	17
Bifidobacterium dentium Bd1 (NC_013714.1)	20.03	299280-299223	-1	arabinan endo-1,5-alpha-L-arabinosidase (BDP_RS01135)	39
	19.4	2559866-2559797	-1	alpha-glucosidase (BDP_RS10610)	2
	17.73	1424860-1424909	1	ABC transporter substrate-binding protein (BDP_RS06160)	8
	17.32	166080-166138	1	sugar ABC transporter substrate-binding protein (BDP_RS00645)	121
	16.87	672199-672249	1	ABC transporter substrate-binding protein (BDP_RS02780)	29
	16.19	1524990-1524937	-1	alcohol dehydrogenase (BDP_RS06575)	21
	15.12	212515-212569	1	LacI family DNA-binding transcriptional regulator (BDP_RS00805)	203
Bifidobacterium dentium JCM 1195 = DSM 20436 DNA (NZ_AP012326.1)	20.03	299282-299225	-1	arabinan endo-1,5-alpha-L-arabinosidase (BBDE_RS01135)	39

Organism (Genome ID)	Bit score	ANTAR-target RNA co-ordinates	Strand	Annotation of the gene linked to ANTAR-target RNAs (Locus Tag)	Distance (nt) of RNA from gene linked to ANTAR-target RNA
	19.4	2559168-2559099	-1	threonine ammonia-lyase (BBDE_RS10610)	2
	17.73	1424863-1424912	1	ABC transporter substrate-binding protein (BBDE_RS06170)	8
	17.32	166081-166139	1	sugar ABC transporter substrate-binding protein (BBDE_RS00645)	121
	16.87	672200-672250	1	ABC transporter substrate-binding protein (BBDE_RS02785)	29
	16.19	1524993-1524940	-1	alcohol dehydrogenase (BBDE_RS06580)	21
	15.12	212517-212571	1	LacI family transcriptional regulator (BBDE_RS00805)	203
Bifidobacterium indicum LMG 11587 = DSM 20214 (NZ_CP006018.1)	23.23	1129914-1129860	-1	MFS transporter (BINDI_RS04395)	46
	16.33	1664773-1664826	1	VOC family protein (BINDI_RS06740)	17
Bifidobacterium kashiwanohense JCM 15439 = DSM 21854 DNA (NZ_AP012327.1)	23.71	2246953-2246903	-1	sugar ABC transporter substrate-binding protein (BBKW_RS09475)	154
Bifidobacterium kashiwanohense JCM 15439 = DSM 21854 DNA (NZ_AP012327.1)	16.87	2142313-2142256	-1	alpha/beta hydrolase (BBKW_RS09080)	15
	16.87	2142324-2142256	-1	alpha/beta hydrolase (BBKW_RS09080)	15
	16.14	557790-557851	1	sugar ABC transporter substrate-binding protein (BBKW_RS02345)	38
	15.53	552772-552725	-1	O-acetylhomoserine aminocarboxypropyltransferase/cysteine synthase (BBKW_RS02325)	12
	15.53	552810-552725	-1	O-acetylhomoserine aminocarboxypropyltransferase/cysteine synthase (BBKW_RS02325)	12
	15.21	2152210-2152151	-1	sugar ABC transporter substrate-binding protein (BBKW_RS09115)	20

Organism (Genome ID)	Bit score	ANTAR-target RNA co-ordinates	Strand	Annotation of the gene linked to ANTAR-target RNAs (Locus Tag)	Distance (nt) of RNA from gene linked to ANTAR-target RNA
Bifidobacterium kashiwanohense PV20-2 (NZ_CP007456.1)	20.44	334680-334711	1	50S ribosomal protein L19 (AH68_RS01310)	369
	20.21	2161764-2161708	-1	sugar ABC transporter substrate-binding protein (AH68_RS09265)	147
	18.59	1763578-1763522	-1	MFS transporter (AH68_RS07705)	54
	17.57	560584-560537	-1	hypothetical protein (AH68_RS02360)	12
	17.55	2261564-2261614	1	beta-glucosidase (AH68_RS09650)	3
	15.71	1901260-1901199	-1	hypothetical protein (AH68_RS08170)	42
	15.33	830090-830149	1	hypothetical protein (AH68_RS03510)	322
Bifidobacterium longum DJO10A (NC_010816.1)	18.58	1451062-1451004	-1	PEGA domain-containing protein (BLD_RS06365)	10
	15.38	287435-287383	-1	aldose 1-epimerase (BLD_RS01220)	23
Bifidobacterium longum DNA, strain: 105-A (NZ_AP014658.1)	21.19	2161684-2161620	-1	glycosyl hydrolase family 43 (BL105A_RS08940)	31
	18.58	198738-198796	1	serine protease (BL105A_RS00830)	10
	16.34	631129-631190	1	hypothetical protein (BL105A_RS02540)	2
	15.44	2064960-2064904	-1	glycosyltransferase family 2 protein (BL105A_RS08660)	73
	15.38	1353107-1353159	1	aldose 1-epimerase (BL105A_RS05765)	23
Bifidobacterium longum NCC2705 (NC_004307.2)	15.38	261999-261947	-1	hypothetical protein (aldose 1-epimerase) (BL1359)	23
	18.58	208320-208378	1	PEGA domain-containing protein (B624_RS00850)	10
	15.44	2087311-2087255	-1	glycosyltransferase family 2 protein (B624_RS08790)	73
	15.38	892689-892637	-1	aldose 1-epimerase (B624_RS03630)	23

Organism (Genome ID)	Bit score	ANTAR-target RNA co-ordinates	Strand	Annotation of the gene linked to ANTAR-target RNAs (Locus Tag)	Distance (nt) of RNA from gene linked to ANTAR-target RNA
Bifidobacterium longum strain BG7 (NZ_CP010453.1)	18.58	221198-221256	1	serine protease (BBG7_RS00940)	10
	18.3	510354-510303	-1	IS256 family transposase (BBG7_RS02240)	27
	18.21	2252785-2252838	1	cell wall-binding repeat-containing protein (BBG7_RS10960)	22
	15.38	1508758-1508810	1	aldose 1-epimerase (BBG7_RS06550)	23
	15.11	667887-667947	1	hypothetical protein (BBG7_RS02825)	2
Bifidobacterium longum strain BXY01 (NZ_CP008885.1)	20.6	1607611-1607667	1	serpin family protein (GS08_RS06860)	5
	16.71	1867745-1867806	1	gfo/Idh/MocA family oxidoreductase (GS08_RS07885)	36
	16.38	239917-239968	1	IS3 family transposase (GS08_RS10520)	183
	16.01	815425-815493	1	GntR family transcriptional regulator (GS08_RS03335)	168
Bifidobacterium longum subsp. infantis 157F DNA (NC_015052.1)	18.58	201502-201560	1	serine protease (BLIF_RS00825)	10
	16.34	629092-629153	1	DUF2975 domain-containing protein (BLIF_RS02475)	2
	15.38	1482075-1482127	1	aldose 1-epimerase (BLIF_RS06550)	23
Bifidobacterium longum subsp. infantis ATCC 15697 = JCM 1222 = DSM 20088 DNA (NC_017219.1)	22.89	918066-918010	-1	serpin family protein (BLIJ_RS03995)	41
Bifidobacterium longum subsp. infantis ATCC 15697 = JCM 1222 = DSM 20088 DNA (NC_017219.1)	20.21	47851-47907	1	sugar ABC transporter substrate-binding protein (BLIJ_RS00230)	147
	18.17	321440-321389	-1	IS256 family transposase (BLIJ_RS01405)	27
	16.8	2786662-2786609	-1	glycosyl hydrolase (BLIJ_RS12785)	78
	15.91	1483251-1483310	1	hypothetical protein (BLIJ_RS06700)	91

Organism (Genome ID)	Bit score	ANTAR-target RNA co-ordinates	Strand	Annotation of the gene linked to ANTAR-target RNAs (Locus Tag)	Distance (nt) of RNA from gene linked to ANTAR-target RNA
	15.84	683987-683926	-1	gfo/Idh/MocA family oxidoreductase (BLIJ_RS03070)	36
Bifidobacterium longum subsp. infantis ATCC 15697 (NC_011593.1)	22.89	918275-918219	-1	serpin family protein (BLON_RS03995)	5
	20.21	48060-48116	1	sugar ABC transporter substrate-binding protein (BLON_RS00235)	147
	18.17	321649-321598	-1	IS256 family transposase (BLON_RS01410)	27
	16.8	2790661-2790608	-1	glycosyl hydrolase (BLON_RS12795)	78
	15.91	1483460-1483519	1	hypothetical protein (BLON_RS06700)	91
	15.84	684195-684134	-1	gfo/Idh/MocA family oxidoreductase (BLON_RS03065)	36
Bifidobacterium longum subsp. infantis genome assembly Bifidobacterium longum subsp. infantis CECT7210 (NZ_LN824140.1)	18.58	2159815-2159873	1	PEGA domain-containing protein (BN1726_RS09745)	10
	16.34	153738-153799	1	hypothetical protein (BN1726_RS00600)	2
	16.01	278919-278987	1	GntR family transcriptional regulator (BN1726_RS01165)	168
	15.38	1010982-1011034	1	aldose 1-epimerase (BN1726_RS04805)	23
Bifidobacterium longum subsp. infantis strain BT1 (NZ_CP010411.1)	18.3	1097527-1097476	-1	IS256 family transposase (RY67_RS04575)	27
	18.3	1998526-1998475	-1	IS256 family transposase (RY67_RS08410)	27
	17.24	343541-343452	-1	sugar ABC transporter permease (RY67_RS01365)	29
	16.8	686952-686899	-1	glycosyl hydrolase (RY67_RS02785)	78
	16.38	1001169-1001220	1	IS3 family transposase (RY67_RS04170)	183
	16.19	1838541-1838489	-1	aldose epimerase (RY67_RS07740)	23

Organism (Genome ID)	Bit score	ANTAR-target RNA co-ordinates	Strand	Annotation of the gene linked to ANTAR-target RNAs (Locus Tag)	Distance (nt) of RNA from gene linked to ANTAR-target RNA
	16.01	1702086-1702033	-1	serpin family protein (RY67_RS07215)	19
	15.84	1434174-1434113	-1	gfo/Idh/MocA family oxidoreductase (RY67_RS06135)	36
	15.37	473578-473639	1	fucose isomerase (RY67_RS01995)	7
	15.11	76204-76144	-1	hypothetical protein (RY67_RS00360)	2
Bifidobacterium longum subsp. longum BBMN68 (NC_014656.1)	21.19	1762029-1762093	1	glycosyl hydrolase family 43 (BBMN68_RS07380)	31
	18.58	1432813-1432755	-1	PEGA domain-containing protein (BBMN68_RS06090)	10
	16.62	304709-304657	-1	aldose 1-epimerase (BBMN68_RS01270)	23
Bifidobacterium longum subsp. longum GT15 (NZ_CP006741.1)	18.58	282168-282226	1	serine protease (BLGT_RS01135)	10
	15.38	1526578-1526630	1	aldose 1-epimerase (BLGT_RS06480)	23
	15.11	766087-766147	1	hypothetical protein (BLGT_RS03075)	2
Bifidobacterium longum subsp. longum JCM 1217 DNA (NC_015067.1)	21.19	2254131-2254067	-1	glycosyl hydrolase family 43 (BLLJ_RS09555)	31
	18.58	216505-216563	1	PEGA domain-containing protein (BLLJ_RS00875)	10
	15.38	1456774-1456826	1	aldose 1-epimerase (BLLJ_RS06415)	23
Bifidobacterium longum subsp. longum JDM301 (NC_014169.1)	20.6	1608192-1608228	1	serpin family protein (BLJ_RS06860)	5
	16.71	1864966-1865027	1	gfo/Idh/MocA family oxidoreductase (BLJ_RS07880)	36
	16.38	239921-239972	1	IS3 family transposase (BLJ_RS00980)	183
	16.01	814071-814139	1	GntR family transcriptional regulator (BLJ_RS03330)	168
Bifidobacterium longum subsp. longum KACC 91563 (NC_017221.1)	18.58	2053635-2053577	-1	PEGA domain-containing protein (BLNIAS_RS08605)	10

Organism (Genome ID)	Bit score	ANTAR-target RNA co-ordinates	Strand	Annotation of the gene linked to ANTAR-target RNAs (Locus Tag)	Distance (nt) of RNA from gene linked to ANTAR-target RNA
	15.38	858755-858703	-1	aldose 1-epimerase (BLNIAS_RS03470)	23
	15.11	1643808-1643748	-1	hypothetical protein (BLNIAS_RS06980)	2
Bifidobacterium longum subsp. longum strain AH1206 (NZ_CP016019.1)	21.19	2295601-2295537	-1	glycosyl hydrolase family 43 (BL1206_RS09965)	31
	15.38	1497218-1497270	1	aldose 1-epimerase (BL1206_RS06775)	23
	15.35	134683-134747	1	sugar ABC transporter substrate-binding protein (BL1206_RS00535)	29
Bifidobacterium longum subsp. longum strain CCUG30698 (NZ_CP011965.1)	21.19	1681266-1681330	1	glycosyl hydrolase family 43 (BBL306_RS07430)	31
	18.58	203778-203836	1	serine protease (BBL306_RS00850)	10
	16.34	637500-637561	1	hypothetical protein (BBL306_RS02550)	2
	15.38	1554627-1554679	1	aldose 1-epimerase (BBL306_RS07005)	23
	18.58	193570-193628	1	serine protease (B8809_RS00770)	10
	15.38	1367183-1367235	1	aldose 1-epimerase (B8809_RS05795)	23
Bifidobacterium pseudocatenulatum DSM 20438 = JCM 1200 = LMG 10505 DNA (NZ_AP012330.1)	20.21	2073057-2073001	-1	sugar ABC transporter substrate-binding protein (BBPC_RS08455)	146
	18.36	1670440-1670384	-1	MFS transporter (BBPC_RS06975)	54
	16.79	210641-210700	1	alpha-arabinofuranosidase (BBPC_RS00790)	21
	16.61	429639-429691	1	MFS transporter (BBPC_RS01680)	13
	16.14	582184-582242	1	sugar ABC transporter substrate-binding protein (BBPC_RS02385)	38
	15.46	1217386-1217319	-1	hypothetical protein (BBPC_RS05115)	18
Bifidobacterium pseudolongum PV8-2 (NZ_CP007457.1)	19.39	254651-254719	1	alpha-N-arabinofuranosidase (AH67_RS01070)	10
	19.19	59994-59941	-1	ABC transporter substrate-binding protein	2

Organism (Genome ID)	Bit score	ANTAR-target RNA co-ordinates	Strand	Annotation of the gene linked to ANTAR-target RNAs (Locus Tag)	Distance (nt) of RNA from gene linked to ANTAR-target RNA
				(AH67_RS00270)	
Bifidobacterium pseudolongum strain DSM 20092 (NZ_CP017695.1)	15.7	1636840-1636906	1	LacI family transcriptional regulator (BP20092_RS06750)	5
Bifidobacterium pseudolongum strain UMB-MBP-01 (NZ_CP022544.1)	19.19	60846-60793	-1	sugar ABC transporter ATP-binding protein (BPSOL_RS00270)	2
	15.18	1884464-1884403	-1	ABC transporter substrate-binding protein (BPSOL_RS08000)	4
Bifidobacterium scardovii JCM 12489 = DSM 13734 DNA (NZ_AP012331.1)	15.74	1087095-1087156	1	hypothetical protein (BBSC_RS04465)	2
	15.53	2861565-2861616	1	amidohydrolase family protein (BBSC_RS11610)	6
	15.32	2977285-2977227	-1	beta-glucosidase (BBSC_RS12000)	11
Brachybacterium sp. P6-10-X1 (NZ_CP017297.1)	20.33	3019349-3019293	-1	HNH endonuclease (BH708_RS13655)	24
	20.33	753990-754046	1	HNH endonuclease (BH708_RS03375)	24
	20.33	3849341-3849285	-1	hypothetical protein (BH708_RS17135)	24
Brevibacterium aurantiacum strain SMQ-1335 (NZ_CP017150.1)	20.67	3576760-3576710	-1	DNA-binding response regulator (BLSMQ_RS16455)	56
	17.36	2225991-2226056	1	SDR family NAD(P)-dependent oxidoreductase (BLSMQ_RS10360)	34
	17.32	1661339-1661274	1	SAF domain-containing protein (BLSMQ_RS07595)	84
	16.49	3773876-3773930	1	50S ribosomal protein L36 (BLSMQ_RS17335)	2
Brevibacterium linens strain BS258 (NZ_CP014869.1)	18.51	2010339-2010393	1	hypothetical protein (A2T55_RS08990)	23
	16.46	2170380-2170321	-1	30S ribosomal protein S1 (A2T55_RS09725)	100
	15.22	378305-378358	1	TetR family transcriptional regulator (A2T55_RS01660)	167

Organism (Genome ID)	Bit score	ANTAR-target RNA co-ordinates	Strand	Annotation of the gene linked to ANTAR-target RNAs (Locus Tag)	Distance (nt) of RNA from gene linked to ANTAR-target RNA
Candidatus Nanopelagicus hibericus isolate MMS-21-160 (NZ_CP016771.1)	23.73	508869-508923	1	DNA-binding response regulator (B1s21160_RS02710)	358
	15.66	1070146-1070090	-1	NAD(P)/FAD-dependent oxidoreductase (B1s21160_RS05485)	67
Candidatus Planktophila dulcis isolate MMS-21-155 (NZ_CP016770.1)	17.48	10956-11027	1	orotate phosphoribosyltransferase (A1s21155_RS00055)	25
	17.48	10974-11045	1	orotate phosphoribosyltransferase (A1sIA53_RS00060)	25
Candidatus Planktophila dulcis isolate MMS-IIA-65 (NZ_CP016777.1)	22.95	10943-11014	1	orotate phosphoribosyltransferase (A1sIIA65_RS00055)	25
Candidatus Planktophila sulfonica isolate MMS-IA-56 (NZ_CP016773.1)	26.26	11044-11115	1	orotate phosphoribosyltransferase (A1sIA56_RS00055)	25
	17.87	883253-883188	-1	30S ribosome-binding factor RbfA (A1sIA56_RS04440)	53
	16.85	315907-315957	1	alpha/beta hydrolase (A1sIA56_RS01595)	154
Candidatus Planktophila versatilis isolate MMS-IA-105 (NZ_CP016775.1)	20.38	10992-11063	1	orotate phosphoribosyltransferase (A1sIA105_RS00055)	25
	15.56	288544-288594	1	MarR family transcriptional regulator (A1sIA105_RS01510)	345
Candidatus Rhodoluna planktonica strain MWH-Dar1 (NZ_CP015208.1)	16.19	226162-226228	1	2-keto-myo-inositol dehydratase (A4Z71_RS01140)	9
Catenulispora acidiphila DSM 44928 (NC_013131.1)	19.55	8698218-8698252	1	MarR family transcriptional regulator (CACI_RS37065)	8
	18.91	3728433-3728363	-1	peptidase S53 (CACI_RS16775)	166
Cellulomonas gilvus ATCC 13127 (NC_015671.1)	22.44	2826924-2826867	-1	alpha-galactosidase (CELGI_RS12885)	4
Clavibacter michiganensis strain	16.87	2513441-2513384	-1	extracellular solute-binding protein	5

Organism (Genome ID)	Bit score	ANTAR-target RNA co-ordinates	Strand	Annotation of the gene linked to ANTAR-target RNAs (Locus Tag)	Distance (nt) of RNA from gene linked to ANTAR-target RNA
PF008 (NZ_CP012573.1)				(AES38_RS11725)	
Clavibacter michiganensis subsp. insidiosus strain R1-1 (NZ_CP011043.1)	17.58	2403997-2403940	-1	carbohydrate-binding protein (VO01_RS11345)	5
Clavibacter michiganensis subsp. michiganensis NCPPB 382 complete genome (NC_009480.1)	19.13	2749746-2749689	-1	carbohydrate-binding protein (CMM_RS12990)	5
	18.53	2521590-2521533	-1	sugar ABC transporter substrate-binding protein (CMN_RS11770)	5
Cnuibacter physcomitrellae strain XA(T) (NZ_CP020715.1)	15.94	3524655-3524708	1	hypothetical protein (B5808_RS16425)	25
Collinsella aerofaciens strain indica (NZ_CP024160.1)	22.55	94937-94997	1	LysR family transcriptional regulator (CSV91_RS00445)	135
	17.26	1874478-1874557	1	hypothetical protein (CSV91_RS08165)	303
	16.75	245313-245260	-1	ketopantoate reductase family protein (CSV91_RS01110)	76
	16.69	1415774-1415725	-1	LacI family DNA-binding transcriptional regulator (CSV91_RS06205)	139
Conexibacter woesei DSM 14684 (NC_013739.1)	17.68	1067939-1067888	-1	hypothetical protein (CWOE_RS05070)	186
Corynebacterium ammoniagenes DSM 20306 strain 9.6 (NZ_CP009244.1)	19.53	2657735-2657682	-1	30S ribosomal protein S6 (CAMM_RS12160)	113
	19.1	2166420-2166366	-1	thymidylate kinase (CAMM_RS09935)	26
	16.84	2331655-2331593	-1	UDP-N-acetylglucosamine 1-carboxyvinyltransferase (CAMM_RS10675)	104
	15.22	2511464-2511408	-1	aldehyde dehydrogenase (CAMM_RS11515)	102
Corynebacterium ammoniagenes	19.53	2744317-2744264	-1	30S ribosomal protein S6	113

Organism (Genome ID)	Bit score	ANTAR-target RNA co-ordinates	Strand	Annotation of the gene linked to ANTAR-target RNAs (Locus Tag)	Distance (nt) of RNA from gene linked to ANTAR-target RNA
strain KCCM 40472 (NZ_CP019705.1)				(CA40472_RS12530)	
	19.1	2253006-2252952	-1	thymidylate kinase (CA40472_RS10300)	26
	16.84	2418238-2418176	-1	UDP-N-acetylglucosamine 1-carboxyvinyltransferase (CA40472_RS11040)	104
	15.22	2598046-2597990	-1	aldehyde dehydrogenase (CA40472_RS11885)	102
Corynebacterium argentoratense DSM 44202 (NC_022198.1)	18.42	34993-34937	-1	hypothetical protein (CARG_RS00190)	349
	15.49	288997-289056	1	adenylate kinase (CARG_RS01425)	97
	15.27	841374-841433	1	30S ribosomal protein S1 (CARG_RS04130)	85
Corynebacterium atypicum strain R2070 (NZ_CP008944.1)	19.2	1161209-1161153	-1	PaaI family thioesterase (CATYP_RS05335)	31
Corynebacterium aurimucosum ATCC 700975 (NC_012590.1)	18.4	844165-844219	1	peptidyl-prolyl cis-trans isomerase (CAURI_RS04070)	44
Corynebacterium callunae DSM 20147 (NC_020506.1)	16.89	97710-97647	-1	hypothetical protein (H924_RS00450)	276
	16.27	1149035-1149091	1	hypothetical protein (H924_RS05470)	7
Corynebacterium camporealensis strain DSM 44610 (NZ_CP011311.1)	15.73	906581-906635	1	DUF4245 domain-containing protein (UL81_RS04305)	149
Corynebacterium casei LMG S-19264 (NZ_CP004350.1)	19.66	282854-282795	-1	glycosyltransferase family 2 protein (CCASEI_RS01445)	421
	15.69	2910386-2910434	1	hypothetical protein (CCASEI_RS13210)	188
Corynebacterium crudilactis strain JZ16 (NZ_CP015622.1)	18.11	2138588-2138650	1	tRNA-Asn (ccrud_RS09975)	6
Corynebacterium cystitidis strain NCTC11863 1 (NZ_LT906473.1)	18.98	15053-15109	1	nucleotide exchange factor GrpE (CKV99_RS00075)	103
Corynebacterium deserti GIMN1.010	16.66	2493014-2492961	-1	glucosamine-6-phosphate deaminase	4

Organism (Genome ID)	Bit score	ANTAR-target RNA co-ordinates	Strand	Annotation of the gene linked to ANTAR-target RNAs (Locus Tag)	Distance (nt) of RNA from gene linked to ANTAR-target RNA
(NZ_CP009220.1)				(CDES_RS11560)	
	15.31	1150457-1150513	1	DEAD/DEAH box helicase (CDES_RS05425)	7
Corynebacterium diphtheriae 241 (NC_016782.1)	16.24	540599-540658	1	membrane protein (CD241_RS02710)	3
	15.1	1194999-1194943	-1	FAD-linked oxidase (CD241_RS05755)	39
Corynebacterium diphtheriae 31A (NC_016799.1)	18.12	2366685-2366748	1	hypothetical protein (CD31A_RS11190)	71
	17.21	608792-608851	1	membrane protein (CD31A_RS03035)	384
Corynebacterium diphtheriae BH8 (NC_016800.1)	18.12	2305800-2305863	1	hypothetical protein (CDBH8_RS11005)	71
	16.24	562111-562170	1	membrane protein (CDBH8_RS02830)	3
Corynebacterium diphtheriae C7 (beta) (NC_016801.1)	17.21	564481-564540	1	membrane protein (CDC7B_RS02855)	384
Corynebacterium diphtheriae CDCE 8392 (NC_016785.1)	17.21	554666-554725	1	membrane protein (CDCE8392_RS02825)	3
	15.25	1806319-1806266	-1	hypothetical protein (CDCE8392_RS11940)	18
Corynebacterium diphtheriae genome assembly NCTC113971 (NZ_LN831026.1)	17.21	528602-528661	1	membrane protein (AT687_RS02655)	384
Corynebacterium diphtheriae HC01 (NC_016786.1)	16.24	540570-540629	1	membrane protein (CDHC01_RS02700)	3
	15.1	1194968-1194912	-1	FAD-linked oxidase (CDHC01_RS05760)	39
Corynebacterium diphtheriae HC02 (NC_016802.1)	17.37	2293876-2293939	1	transposase (CDHC02_RS12255)	71
	16.24	562291-562350	1	membrane protein (CDHC02_RS02800)	3
Corynebacterium diphtheriae HC03 (NC_016787.1)	17.37	2287584-2287647	1	transposase (CDHC03_RS12065)	71
	17.21	542020-542079	1	membrane protein (CDHC03_RS02695)	384
Corynebacterium diphtheriae HC04 (NC_016788.1)	17.37	2311402-2311465	1	transposase (CDHC04_RS12195)	71
	17.21	530537-530596	1	membrane protein (CDHC04_RS02615)	384

Organism (Genome ID)	Bit score	ANTAR-target RNA co-ordinates	Strand	Annotation of the gene linked to ANTAR-target RNAs (Locus Tag)	Distance (nt) of RNA from gene linked to ANTAR-target RNA
	15.1	1194413-1194357	-1	FAD-linked oxidase (CDHC04_RS05780)	39
Corynebacterium diphtheriae INCA 402 (NC_016783.1)	16.24	544750-544809	1	membrane protein (CDB402_RS02690)	3
Corynebacterium diphtheriae NCTC 13129 (NC_002935.2)	17.21	582246-582305	1	membrane protein (DIP_RS14365)	3
	15.25	1881495-1881442	-1	hypothetical protein (DIP_RS23555)	18
Corynebacterium diphtheriae PW8 (NC_016789.1)	17.21	609691-609750	1	membrane protein (CDPW8_RS03110)	384
Corynebacterium diphtheriae strain B-D-16-78 (NZ_CP018331.1)	16.24	2272880-2272939	1	membrane protein (BS112_RS11015)	3
Corynebacterium diphtheriae strain FDAARGOS_197 (NZ_CP020410.1)	17.21	2449468-2449527	1	membrane protein (A6J36_RS11710)	384
	15.25	1259703-1259650	-1	hypothetical protein (A6J36_RS05950)	18
Corynebacterium diphtheriae VA01 (NC_016790.1)	17.37	2208578-2208641	1	transposase (CDVA01_RS11645)	71
	17.21	519377-519436	1	membrane protein (CDVA01_RS02540)	384
	15.1	1161803-1161747	-1	FAD-linked oxidase (CDVA01_RS05515)	39
Corynebacterium efficiens YS-314 DNA (NC_004369.1)	20.35	2288328-2288390	1	hypothetical protein (CE_RS14970)	245
Corynebacterium efficiens YS-314 DNA (NC_004369.1)	19.22	2797301-2797242	-1	nucleotide exchange factor GrpE (CE_RS13010)	20
Corynebacterium epidermidicanis strain DSM 45586 (NZ_CP011541.1)	15.51	542196-542146	-1	O-acetyl-L-homoserine sulfhydrolase (CEPID_RS02575)	95
Corynebacterium falsenii DSM 44353 strain BL 8171 (NZ_CP007156.1)	16.91	1654540-1654593	1	amino acid-binding ACT domain protein (BI88_RS07010)	97
Corynebacterium flavescens strain OJ8 (NZ_CP009246.1)	16.26	1947958-1948017	1	ABC transporter ATP-binding protein (CFLV_RS08970)	294
Corynebacterium glaucum strain DSM 30827 (NZ_CP019688.1)	15.95	718849-718909	1	hypothetical protein (CGLAU_RS03530)	42

Organism (Genome ID)	Bit score	ANTAR-target RNA co-ordinates	Strand	Annotation of the gene linked to ANTAR-target RNAs (Locus Tag)	Distance (nt) of RNA from gene linked to ANTAR-target RNA
Corynebacterium glutamicum ATCC 13032 (NC_003450.3)	17.48	2984618-2984562	-1	heat shock protein GrpE (NCgl2701)	21
	15.4	2735190-2735137	-1	ABC transporter permease (NCgl2484)	5
	15.31	1209941-1209997	1	helicase (NCgl1109)	7
Corynebacterium glutamicum ATCC 13032, IS fingerprint type 4-5 (NC_006958.1)	17.48	2957923-2957867	-1	nucleotide exchange factor GrpE (CGTRNA_RS13835)	21
	15.4	2706860-2706808	-1	phosphate ABC transporter, permease protein PstA (CGTRNA_RS12735)	47
	15.31	1211409-1211465	1	DEAD/DEAH box helicase (CGTRNA_RS05780)	7
Corynebacterium glutamicum ATCC 14067 (NZ_CP022614.1)	16.55	638609-638553	-1	UDP-glucose 4-epimerase GalE (CEY17_RS03130)	64
	15.4	2681755-2681703	-1	phosphate ABC transporter, permease protein PstA (CEY17_RS13150)	47
	15.31	1289628-1289684	1	DEAD/DEAH box helicase (CEY17_RS06335)	7
	15.24	1188383-1188440	1	MFS transporter (CEY17_RS05865)	43
Corynebacterium glutamicum DNA, strain: AJ1511 (NZ_AP017557.1)	15.4	2685573-2685521	-1	phosphate ABC transporter, permease protein PstA (CGBL_RS12700)	47
	15.31	1316640-1316696	1	ATP-dependent RNA helicase (CGBL_RS06275)	7
	15.24	1218594-1218651	1	MFS transporter (CGBL_RS05825)	43
Corynebacterium glutamicum DNA, strain: N24 (NZ_AP017369.1)	18.59	3179973-3179917	-1	nucleotide exchange factor GrpE (N24_RS14630)	21
	16.52	1657111-1657172	1	MerR family transcriptional regulator (N24_RS07815)	15
	15.68	2337810-2337750	-1	NADP-specific glutamate dehydrogenase (N24_RS10865)	67

Organism (Genome ID)	Bit score	ANTAR-target RNA co-ordinates	Strand	Annotation of the gene linked to ANTAR-target RNAs (Locus Tag)	Distance (nt) of RNA from gene linked to ANTAR-target RNA
	15.31	1359688-1359744	1	ATP-dependent RNA helicase (N24_RS06480)	7
	15.21	2843690-2843740	1	FadR family transcriptional regulator (N24_RS13155)	82
	15.12	3237214-3237161	-1	PRD domain-containing protein (N24_RS14870)	10
Corynebacterium glutamicum K051 complete genome, strain ATCC 13032, sub-strain K051 (NC_020519.1)	17.48	2984617-2984561	-1	nucleotide exchange factor GrpE (WA5_RS14000)	21
	15.4	2735189-2735137	-1	phosphate ABC transporter, permease protein PstA (WA5_RS12905)	47
	15.31	1209940-1209996	1	helicase (WA5_RS05750)	7
Corynebacterium glutamicum MB001 (NC_022040.1)	17.48	2754470-2754414	-1	nucleotide exchange factor GrpE (CGP_RS12880)	21
	15.4	2503403-2503351	-1	phosphate ABC transporter, permease protein PstA (CGP_RS11780)	47
	15.31	1212837-1212893	1	helicase (CGP_RS05770)	7
Corynebacterium glutamicum R DNA (NC_009342.1)	20.62	714117-714064	-1	PaaI family thioesterase (CGR_RS03420)	385
	17.48	2977118-2977062	-1	nucleotide exchange factor GrpE (CGR_RS13900)	21
	16.55	735566-735510	-1	UDP-glucose 4-epimerase GalE (CGR_RS03515)	64
	16.4	1256913-1256970	1	MFS transporter (CGR_RS05950)	43
	15.67	3018403-3018350	-1	PRD domain-containing protein (CGR_RS14090)	10
	15.4	2734181-2734129	-1	phosphate ABC transporter, permease protein PstA (CGR_RS12865)	47
	15.31	1352549-1352605	1	DEAD/DEAH box helicase	7

Organism (Genome ID)	Bit score	ANTAR-target RNA co-ordinates	Strand	Annotation of the gene linked to ANTAR-target RNAs (Locus Tag)	Distance (nt) of RNA from gene linked to ANTAR-target RNA
				(CGR_RS06390)	
Corynebacterium glutamicum SCgG1 (NC_021351.1)	20.62	701116-701063	-1	PaaI family thioesterase (C624_RS03385)	385
	17.48	3001152-3001096	-1	nucleotide exchange factor GrpE (C624_RS13910)	21
	16.55	722574-722518	-1	UDP-glucose 4-epimerase GalE (C624_RS03480)	64
	15.67	3044637-3044584	-1	PRD domain-containing protein (C624_RS14110)	10
	15.4	2753889-2753837	-1	phosphate ABC transporter, permease protein PstA (C624_RS12865)	47
	15.31	1401709-1401765	1	DEAD/DEAH box helicase (C624_RS06640)	7
Corynebacterium glutamicum SCgG2 (NC_021352.1)	20.62	701117-701064	-1	PaaI family thioesterase (C629_RS03385)	385
	17.48	3001151-3001095	-1	nucleotide exchange factor GrpE (C629_RS13905)	21
	16.55	722575-722519	-1	UDP-glucose 4-epimerase GalE (C629_RS03480)	64
	15.67	3044636-3044583	-1	PRD domain-containing protein (C629_RS14105)	10
	15.4	2753888-2753836	-1	phosphate ABC transporter, permease protein PstA (C629_RS12860)	47
	15.31	1401708-1401764	1	DEAD/DEAH box helicase (C629_RS06635)	7
Corynebacterium glutamicum strain AR1 (NZ_CP007724.1)	20.62	626016-625963	-1	PaaI family thioesterase (CGLAR1_RS03095)	385
	17.25	1164052-1164109	1	MFS transporter (CGLAR1_RS05565)	44
	15.4	2573691-2573639	-1	phosphate ABC transporter, permease	47

Organism (Genome ID)	Bit score	ANTAR-target RNA co-ordinates	Strand	Annotation of the gene linked to ANTAR-target RNAs (Locus Tag)	Distance (nt) of RNA from gene linked to ANTAR-target RNA
				protein PstA (CGLAR1_RS12090)	
	15.31	1259623-1259679	1	DEAD/DEAH box helicase (CGLAR1_RS06005)	7
Corynebacterium glutamicum strain ATCC 13869 (NZ_CP016335.1)	15.4	2685568-2685516	-1	phosphate ABC transporter, permease protein PstA (BBD29_RS12560)	47
	15.31	1314970-1315026	1	ATP-dependent RNA helicase (BBD29_RS06195)	7
	15.24	1216979-1217036	1	MFS transporter (BBD29_RS05735)	43
Corynebacterium glutamicum strain ATCC 21831 (NZ_CP007722.1)	20.62	656832-656779	-1	PaaI family thioesterase (AR0_RS03220)	385
	17.25	1194869-1194926	1	MFS transporter (AR0_RS05680)	44
	15.4	2604096-2604044	-1	phosphate ABC transporter, permease protein PstA (AR0_RS12200)	47
	15.31	1290440-1290496	1	DEAD/DEAH box helicase (AR0_RS06120)	7
Corynebacterium glutamicum strain B253 (NZ_CP010451.1)	15.4	2601352-2601300	-1	phosphate ABC transporter, permease protein PstA (SB89_RS12100)	47
	15.31	1286056-1286112	1	DEAD/DEAH box helicase (SB89_RS06035)	7
	15.24	1179688-1179745	1	MFS transporter (SB89_RS05560)	43
Corynebacterium glutamicum strain CP (NZ_CP012194.1)	15.4	2729849-2729797	-1	phosphate ABC transporter, permease protein PstA (AC079_RS12905)	47
	15.31	1355779-1355835	1	DEAD/DEAH box helicase (AC079_RS06490)	7
	15.24	1257734-1257791	1	MFS transporter (AC079_RS06035)	43
Corynebacterium glutamicum strain TQ2223 (NZ_CP020658.1)	17.48	3060506-3060562	1	nucleotide exchange factor GrpE (B7P23_RS14495)	21
	15.4	28885-28938	1	phosphate ABC transporter, permease	5

Organism (Genome ID)	Bit score	ANTAR-target RNA co-ordinates	Strand	Annotation of the gene linked to ANTAR-target RNAs (Locus Tag)	Distance (nt) of RNA from gene linked to ANTAR-target RNA
				protein PstA (B7P23_RS00130)	
	15.31	1522687-1522631	-1	helicase (B7P23_RS07145)	7
Corynebacterium glutamicum strain USDA-ARS-USMARC-56828 (NZ_CP013991.1)	17.48	2931080-2931024	-1	nucleotide exchange factor GrpE (APT58_RS13695)	21
	15.67	2972568-2972515	-1	transcription antiterminator BglG (APT58_RS13885)	10
	15.61	672726-672673	-1	PaaI family thioesterase (APT58_RS03280)	385
	15.4	2683030-2682978	-1	phosphate ABC transporter, permease protein PstA (APT58_RS12645)	47
	15.31	1310879-1310935	1	DEAD/DEAH box helicase (APT58_RS06245)	7
	15.27	438395-438335	-1	esterase family protein (APT58_RS02155)	368
Corynebacterium glutamicum strain WM001 (NZ_CP022394.1)	15.4	2237589-2237537	-1	phosphate ABC transporter, permease protein PstA (CGB98_RS10580)	47
	15.31	865646-865702	1	ATP-dependent RNA helicase (CGB98_RS04160)	7
	15.24	767600-767657	1	MFS transporter (CGB98_RS03710)	43
Corynebacterium glutamicum strain XV (NZ_CP018175.1)	15.4	2705759-2705707	-1	phosphate ABC transporter, permease protein PstA (BSP99_RS12860)	47
	15.4	2718514-2718462	-1	phosphate ABC transporter, permease protein PstA (BSP99_RS12945)	47
	15.31	1323843-1323899	1	ATP-dependent RNA helicase (BSP99_RS06370)	7
	15.24	1225797-1225854	1	MFS transporter (BSP99_RS05915)	43
Corynebacterium glutamicum strain YI (NZ_CP014984.1)	16.55	637028-636972	-1	UDP-glucose 4-epimerase GalE (A3654_RS03070)	64

Organism (Genome ID)	Bit score	ANTAR-target RNA co-ordinates	Strand	Annotation of the gene linked to ANTAR-target RNAs (Locus Tag)	Distance (nt) of RNA from gene linked to ANTAR-target RNA
	15.4	2676018-2675966	-1	phosphate ABC transporter, permease protein PstA (A3654_RS13005)	47
	15.31	1289003-1289059	1	DEAD/DEAH box helicase (A3654_RS06260)	7
	15.24	1187758-1187815	1	MFS transporter (A3654_RS05790)	43
Corynebacterium glutamicum ZL-6 (NZ_CP004062.1)	15.4	2716163-2716111	-1	phosphate ABC transporter, permease protein PstA (C627_RS12795)	47
	15.31	1332273-1332329	1	ATP-dependent RNA helicase (C627_RS06325)	7
	15.24	1234226-1234283	1	MFS transporter (C627_RS05865)	43
Corynebacterium glyciniphilum AJ 3170 (NZ_CP006842.1)	20.66	641835-641767	-1	acyl-CoA oxidase (CGLY_RS02960)	249
Corynebacterium humireducens NBRC 106098 = DSM 45392 (NZ_CP005286.1)	17.13	2213593-2213539	-1	tRNA-Asp (B842_RS10925)	38
Corynebacterium imitans strain DSM 44264 (NZ_CP009211.1)	15.08	817633-817686	1	TrkA family potassium uptake protein (CIMIT_RS03730)	88
Corynebacterium imitans strain NCTC13015 1 (NZ_LT906467.1)	15.08	899599-899652	1	TrkA family potassium uptake protein (CKV97_RS04135)	40
Corynebacterium jeikeium strain FDAARGOS_328 (NZ_CP022054.1)	19.04	276098-276155	1	hypothetical protein (CEQ06_RS01215)	32
	15.47	2197898-2197951	1	hypothetical protein (CEQ06_RS09770)	34
	15.14	1717433-1717377	-1	nucleotide exchange factor GrpE (CEQ06_RS07760)	8
Corynebacterium kroppenstedtii DSM 44385 (NC_012704.1)	16.99	1893895-1893951	1	hypothetical protein (CKROP_RS07925)	5
	16.31	1439672-1439725	1	IclR family transcriptional regulator (CKROP_RS05980)	370
Corynebacterium lactis RW2-5	21.46	144577-144541	-1	glycosyltransferase family 2 protein	159

Organism (Genome ID)	Bit score	ANTAR-target RNA co-ordinates	Strand	Annotation of the gene linked to ANTAR-target RNAs (Locus Tag)	Distance (nt) of RNA from gene linked to ANTAR-target RNA
(NZ_CP006841.1)				(CLAC_RS00545)	
	19.94	2631157-2631106	-1	hypothetical protein (CLAC_RS11575)	54
Corynebacterium marinum DSM 44953 plasmid pCmarinum2 (NZ_CP007791.1)	17.39	8792-8740	-1	replicase RepA (B840_RS13300)	357
Corynebacterium mustelae strain DSM 45274 (NZ_CP011542.1)	16.62	1400440-1400500	1	hypothetical protein (CMUST_RS06530)	54
Corynebacterium phocae strain M408/89/1 (NZ_CP009249.1)	15.58	723165-723214	1	bifunctional folylpolyglutamate synthase/dihydrofolate synthase (CPHO_RS03305)	93
Corynebacterium pseudotuberculosis 1/06-A (NC_017308.1)	16.04	1945271-1945324	1	cardiolipin synthase (CP106_RS08885)	26
Corynebacterium pseudotuberculosis 1002 (NC_017300.1)	16.51	1995873-1995926	1	cardiolipin synthase (CP1002_RS09075)	26
Corynebacterium pseudotuberculosis 258 (NC_017945.2)	16.04	2028099-2028152	1	cardiolipin synthase (CP258_RS09260)	26
Corynebacterium pseudotuberculosis 267 (NC_017462.1)	16.04	1998436-1998489	1	cardiolipin synthase (CP267_RS09070)	26
Corynebacterium pseudotuberculosis 3/99-5 (NC_016781.1)	16.04	1998501-1998554	1	cardiolipin synthase (CP3995_RS09100)	26
Corynebacterium pseudotuberculosis 316 (NC_016932.1)	16.04	1969760-1969813	1	cardiolipin synthase (CP316_RS08945)	26
Corynebacterium pseudotuberculosis 42/02-A (NC_017306.1)	16.04	1998385-1998438	1	cardiolipin synthase (CP4202_RS09095)	26
Corynebacterium pseudotuberculosis C231 (NC_017301.1)	16.04	1988938-1988991	1	cardiolipin synthase (CPC231_RS19465)	26
Corynebacterium pseudotuberculosis CIP 52.97 (NC_017307.2)	16.04	2026638-2026691	1	cardiolipin synthase (CPCIP5297_RS09505)	26

Organism (Genome ID)	Bit score	ANTAR-target RNA co-ordinates	Strand	Annotation of the gene linked to ANTAR-target RNAs (Locus Tag)	Distance (nt) of RNA from gene linked to ANTAR-target RNA
Corynebacterium pseudotuberculosis Cp162 (NC_018019.2)	16.04	2024914-2024967	1	cardiolipin synthase (CP162_RS09460)	26
Corynebacterium pseudotuberculosis FRC41 (NC_014329.1)	16.04	1998568-1998621	1	cardiolipin synthase (CPFRC_RS09100)	26
Corynebacterium pseudotuberculosis I19 (NC_017303.2)	16.04	1998109-1998162	1	cardiolipin synthase (CPI19_RS09295)	26
Corynebacterium pseudotuberculosis P54B96 (NC_017031.1)	16.04	1998340-1998393	1	cardiolipin synthase (CPP54B96_RS09090)	26
Corynebacterium pseudotuberculosis PAT10 (NC_017305.1)	16.04	1995966-1996019	1	cardiolipin synthase (CPPAT10_RS09080)	26
Corynebacterium pseudotuberculosis strain 1002B (NZ_CP012837.1)	16.51	854171-854118	-1	cardiolipin synthase (Cp1002B_RS03865)	26
Corynebacterium pseudotuberculosis strain 12C (NZ_CP011474.1)	16.51	1998115-1998168	1	cardiolipin synthase (Cp12C_RS09100)	26
Corynebacterium pseudotuberculosis strain 226 (NZ_CP010889.1)	16.04	1998442-1998495	1	cardiolipin synthase (CP226_RS09090)	26
Corynebacterium pseudotuberculosis strain 262 (NZ_CP012022.2)	16.04	2021693-2021746	1	cardiolipin synthase (Cp262_RS09400)	26
Corynebacterium pseudotuberculosis strain 29156 (NZ_CP010795.1)	16.04	854831-854778	-1	cardiolipin synthase (Cp29156_RS03900)	26
Corynebacterium pseudotuberculosis strain 32 (NZ_CP015183.1)	16.04	2060899-2060952	1	cardiolipin synthase (A4R72_RS09470)	26
Corynebacterium pseudotuberculosis strain 33 (NZ_CP015184.1)	16.04	2060913-2060966	1	cardiolipin synthase (A4R71_RS09485)	26
Corynebacterium pseudotuberculosis strain 34 (NZ_CP015192.1)	16.04	2060808-2060861	1	cardiolipin synthase (A4R69_RS09465)	26
Corynebacterium pseudotuberculosis strain 35 (NZ_CP015185.1)	16.04	2060864-2060917	1	cardiolipin synthase (A4R68_RS09485)	26

Organism (Genome ID)	Bit score	ANTAR-target RNA co-ordinates	Strand	Annotation of the gene linked to ANTAR-target RNAs (Locus Tag)	Distance (nt) of RNA from gene linked to ANTAR-target RNA
Corynebacterium pseudotuberculosis strain 36 (NZ_CP015186.1)	16.04	2060782-2060835	1	cardiolipin synthase (A4R67_RS09460)	26
Corynebacterium pseudotuberculosis strain 38 (NZ_CP015187.1)	16.04	2060890-2060943	1	cardiolipin synthase (A4R66_RS09460)	26
Corynebacterium pseudotuberculosis strain 39 (NZ_CP015188.1)	16.04	2060955-2061008	1	cardiolipin synthase (A4R65_RS09455)	26
Corynebacterium pseudotuberculosis strain 43 (NZ_CP015189.1)	16.04	2022444-2022497	1	cardiolipin synthase (A4R64_RS09255)	26
Corynebacterium pseudotuberculosis strain 46 (NZ_CP015190.1)	16.04	2023915-2023968	1	cardiolipin synthase (A4R63_RS09235)	26
Corynebacterium pseudotuberculosis strain 48 (NZ_CP015191.1)	16.04	2060676-2060729	1	cardiolipin synthase (A4R62_RS09470)	26
Corynebacterium pseudotuberculosis strain 48252 (NZ_CP008922.1)	16.04	214647-214700	1	cardiolipin synthase (CPTA_RS01030)	26
Corynebacterium pseudotuberculosis strain ATCC 19410 (NZ_CP021251.1)	16.04	854231-854178	-1	cardiolipin synthase (CpATCC19410_RS03970)	26
Corynebacterium pseudotuberculosis strain Cp13 (NZ_CP014998.1)	16.51	2002902-2002955	1	cardiolipin synthase (CpCp13_RS09135)	26
Corynebacterium pseudotuberculosis strain CS_10 (NZ_CP008923.1)	16.04	1381691-1381638	-1	cardiolipin synthase (CPTB_RS06210)	26
Corynebacterium pseudotuberculosis strain E19 (NZ_CP012136.1)	16.04	2026360-2026413	1	cardiolipin synthase (CpE19_RS09260)	26
Corynebacterium pseudotuberculosis strain E55 (NZ_CP014341.1)	16.04	853706-853653	-1	cardiolipin synthase (CpE55_RS03870)	26
Corynebacterium pseudotuberculosis strain E56 (NZ_CP013699.1)	16.04	853708-853655	-1	cardiolipin synthase (AN398_RS03860)	26
Corynebacterium pseudotuberculosis	16.04	1018334-1018281	-1	cardiolipin synthase (CPTC_RS04600)	26

Organism (Genome ID)	Bit score	ANTAR-target RNA co-ordinates	Strand	Annotation of the gene linked to ANTAR-target RNAs (Locus Tag)	Distance (nt) of RNA from gene linked to ANTAR-target RNA
strain Ft_2193/67 (NZ_CP008924.1)					
Corynebacterium pseudotuberculosis strain I37 (NZ_CP017384.1)	16.04	2027288-2027341	1	cardiolipin synthase (CPI37_RS09430)	26
Corynebacterium pseudotuberculosis strain MB11 (NZ_CP013260.1)	16.04	2131290-2131343	1	cardiolipin synthase (ATN02_RS09730)	26
Corynebacterium pseudotuberculosis strain MB14 (NZ_CP013261.1)	16.04	2027869-2027922	1	cardiolipin synthase (ATN03_RS09285)	26
Corynebacterium pseudotuberculosis strain MB20 (NZ_CP016829.1)	16.04	971322-971269	-1	cardiolipin synthase (CPMB20_RS04670)	26
Corynebacterium pseudotuberculosis strain MB278 (NZ_CP023395.1)	16.04	2027958-2028011	1	cardiolipin synthase (BFF96_RS09510)	26
Corynebacterium pseudotuberculosis strain MB30 (NZ_CP013262.2)	16.04	2026744-2026797	1	cardiolipin synthase (ATN04_RS09500)	26
Corynebacterium pseudotuberculosis strain MB302 (NZ_CP021982.1)	16.04	2025938-2025991	1	cardiolipin synthase (BFG01_RS09500)	26
Corynebacterium pseudotuberculosis strain MB66 (NZ_CP013263.1)	16.04	971242-971189	-1	cardiolipin synthase (ATN05_RS04530)	26
Corynebacterium pseudotuberculosis strain MEX1 (NZ_CP017711.1)	16.04	854185-854132	-1	cardiolipin synthase (CpMEX1_RS03945)	26
Corynebacterium pseudotuberculosis strain MEX25 (NZ_CP013697.1)	16.04	854204-854151	-1	cardiolipin synthase (AN397_RS03870)	26
Corynebacterium pseudotuberculosis strain MEX29 (NZ_CP016826.1)	16.04	854229-854176	-1	cardiolipin synthase (CpMEX29_RS03870)	26
Corynebacterium pseudotuberculosis strain MEX30 (NZ_CP017291.1)	16.04	2026292-2026345	1	cardiolipin synthase (CpMEX30_RS09515)	26
Corynebacterium pseudotuberculosis strain MEX31 (NZ_CP017292.1)	16.04	2026049-2026102	1	cardiolipin synthase (CpMEX31_RS09480)	26
Corynebacterium pseudotuberculosis	16.04	854213-854160	-1	cardiolipin synthase (CpMEX9_RS03890)	26

Organism (Genome ID)	Bit score	ANTAR-target RNA co-ordinates	Strand	Annotation of the gene linked to ANTAR-target RNAs (Locus Tag)	Distance (nt) of RNA from gene linked to ANTAR-target RNA
strain MEX9 (NZ_CP014543.1)					
Corynebacterium pseudotuberculosis strain MIC6 (NZ_CP019769.1)	16.51	1997836-1997889	1	cardiolipin synthase (CpMIC6_RS09295)	26
Corynebacterium pseudotuberculosis strain N1 (NZ_CP013146.1)	16.04	854467-854414	-1	cardiolipin synthase (CpN1_RS03870)	26
Corynebacterium pseudotuberculosis strain PA01 (NZ_CP013327.1)	16.04	1998430-1998483	1	cardiolipin synthase (CpPA01_RS09085)	26
Corynebacterium pseudotuberculosis strain PA02 (NZ_CP015309.1)	16.51	1989095-1989148	1	cardiolipin synthase (CpPA02_RS09055)	26
Corynebacterium pseudotuberculosis strain PA04 (NZ_CP019587.1)	16.04	1998550-1998603	1	cardiolipin synthase (CpPa04_RS09295)	26
Corynebacterium pseudotuberculosis strain phoP (NZ_CP019768.1)	16.51	854156-854103	-1	cardiolipin synthase (CpphoP_RS03960)	26
Corynebacterium pseudotuberculosis strain PO222/4-1 (NZ_CP013698.1)	16.04	854197-854144	-1	cardiolipin synthase (AK970_RS03870)	26
Corynebacterium pseudotuberculosis strain PO269-5 (NZ_CP012695.1)	16.04	854164-854111	-1	cardiolipin synthase (AN902_RS03875)	26
Corynebacterium pseudotuberculosis strain SigmaE (NZ_CP020356.1)	16.51	854123-854070	-1	cardiolipin synthase (CpSigmaE_RS03950)	26
Corynebacterium pseudotuberculosis strain T1 (NZ_CP015100.1)	16.51	1997875-1997928	1	cardiolipin synthase (CpT1_RS09115)	26
Corynebacterium pseudotuberculosis strain VD57 (NZ_CP009927.1)	16.51	1997850-1997903	1	cardiolipin synthase (cpvd57_RS09075)	26
Corynebacterium resistens DSM 45100 (NC_015673.1)	23.06	784296-784358	1	bifunctional folylpolyglutamate synthase/dihydrofolate synthase (CRES_RS03270)	84
Corynebacterium simulans strain PES1 (NZ_CP014634.1)	19.51	2038187-2038240	1	alanine:cation symporter family protein (WM42_RS09435)	422

Organism (Genome ID)	Bit score	ANTAR-target RNA co-ordinates	Strand	Annotation of the gene linked to ANTAR-target RNAs (Locus Tag)	Distance (nt) of RNA from gene linked to ANTAR-target RNA
	15.04	1825255-1825308	1	hypoxanthine phosphoribosyltransferase (WM42_RS08480)	31
Corynebacterium simulans strain Wattiau (NZ_CP014635.1)	19.51	2075643-2075696	1	alanine:cation symporter family protein (AWU68_RS09625)	422
Corynebacterium singulare strain IBS B52218 (NZ_CP010827.1)	15.06	379234-379174	-1	ABC transporter permease (CSING_RS01710)	51
Corynebacterium sp. ATCC 6931 (NZ_CP008913.1)	19.04	2170035-2170092	1	hypothetical protein (DR71_RS09350)	32
	15.57	2171805-2171866	1	phosphoglycerate dehydrogenase (DR71_RS09355)	158
	15.29	533505-533558	1	CBS domain-containing protein (DR71_RS02185)	6
Corynebacterium sp. NML98-0116 genome (NZ_CP017639.1)	19.17	282870-282926	1	nucleotide exchange factor GrpE (BJP05_RS01180)	39
Corynebacterium stationis strain 622=DSM 20302 (NZ_CP009251.1)	19.79	301620-301569	-1	peptidase M23 (CSTAT_RS01510)	372
	19.05	658233-658185	-1	ribonuclease HI (CSTAT_RS03225)	5
Corynebacterium striatum strain KC-Na-01 (NZ_CP021252.1)	19.51	737452-737505	1	alanine:cation symporter family protein (CBE89_RS03575)	422
	16.4	192948-193016	1	hypothetical protein (CBE89_RS01090)	25
Corynebacterium terpenotabidum Y-11 (NC_021663.1)	18.6	266084-266134	1	N-carbamoylsarcosine amidase (A606_RS01080)	361
Corynebacterium testudinoris strain DSM 44614 (NZ_CP011545.1)	16.85	2111774-2111716	-1	TetR/AcrR family transcriptional regulator (CTEST_RS10110)	71
Corynebacterium ulcerans 0102 DNA (NC_018101.1)	15.6	1773540-1773605	1	serine/threonine protein kinase (CULC0102_RS08195)	315
Corynebacterium ulcerans 809 (NC_017317.1)	15.6	1691258-1691323	1	serine/threonine protein kinase (CULC809_RS07755)	315
Corynebacterium ulcerans BR-AD22	15.6	1694931-1694996	1	serine/threonine protein kinase	315

Organism (Genome ID)	Bit score	ANTAR-target RNA co-ordinates	Strand	Annotation of the gene linked to ANTAR-target RNAs (Locus Tag)	Distance (nt) of RNA from gene linked to ANTAR-target RNA
(NC_015683.1)				(CULC22_RS07730)	
Corynebacterium ulcerans FRC58 (NZ_CP011913.1)	15.6	1677923-1677988	1	serine/threonine protein kinase (CULFRC58_RS07655)	315
	15.17	330052-330103	1	hypothetical protein (CULFRC58_RS01565)	193
Corynebacterium ulcerans strain 05146 (NZ_CP009716.1)	16.55	2091358-2091409	1	Na+/H+ antiporter subunit E (CUL05146_RS09480)	39
	15.6	1642146-1642211	1	serine/threonine protein kinase (CUL05146_RS07450)	315
Corynebacterium ulcerans strain 131001 (NZ_CP010818.1)	15.6	1681236-1681301	1	serine/threonine protein kinase (Cul131001_RS07640)	315
Corynebacterium ulcerans strain 210931 (NZ_CP009583.1)	16.55	2122575-2122626	1	Na+/H+ antiporter subunit E (CUL210931_RS09775)	39
	15.6	1637127-1637192	1	serine/threonine protein kinase (CUL210931_RS07495)	315
Corynebacterium ulcerans strain 210932 (NZ_CP009500.1)	15.6	1681546-1681611	1	serine/threonine protein kinase (CUL210932_RS07655)	315
Corynebacterium ulcerans strain NCTC7910 1 (NZ_LT906443.1)	15.6	661088-661153	1	serine/threonine protein kinase (CKV68_RS02940)	315
Corynebacterium ulcerans strain PO100/5 (NZ_CP021417.1)	17.7	1615651-1615601	-1	terminase (CBE74_RS07650)	24
	15.45	2207204-2207255	1	Na+/H+ antiporter subunit E (CBE74_RS10395)	39
	15.24	2530763-2530701	-1	aldose epimerase (CBE74_RS11790)	278
Corynebacterium urealyticum DSM 7109 complete genome (NC_010545.1)	21.91	2341876-2341820	-1	NUDIX domain-containing protein (CU_RS10045)	487
	19.64	547036-547092	1	biotin--[acetyl-CoA-carboxylase] ligase (CU_RS02260)	435

Organism (Genome ID)	Bit score	ANTAR-target RNA co-ordinates	Strand	Annotation of the gene linked to ANTAR-target RNAs (Locus Tag)	Distance (nt) of RNA from gene linked to ANTAR-target RNA
	15.54	657867-657914	1	PDZ domain-containing protein (CU_RS02720)	28
Corynebacterium urealyticum DSM 7111 (NC_020230.1)	19.64	536435-536491	1	biotin--[acetyl-CoA-carboxylase] ligase (CU7111_RS02240)	435
	15.54	637433-637480	1	PDZ domain-containing protein (CU7111_RS02665)	28
Corynebacterium urealyticum strain NCTC12011 1 (NZ_LT906481.1)	21.91	1654930-1654874	-1	NUDIX domain-containing protein (CKV82_RS07305)	487
	19.64	2232424-2232480	1	biotin--[acetyl-CoA-carboxylase] ligase (CKV82_RS09705)	435
	15.54	2346331-2346378	1	PDZ domain-containing protein (CKV82_RS10185)	28
Corynebacterium uterequi strain DSM 45634 (NZ_CP011546.1)	16.32	857566-857513	-1	glycogen synthase (CUTER_RS04050)	88
	15.31	1596657-1596597	-1	heme-copper oxidase subunit III (CUTER_RS07405)	340
Cryobacterium arcticum strain PAMC 27867 1 (NZ_CP016282.1)	22.35	3698660-3698598	-1	LacI family transcriptional regulator (PA27867_RS16790)	6
	16.53	2539464-2539403	-1	alcohol dehydrogenase (PA27867_RS11370)	33
Cryobacterium sp. LW097 (NZ_CP021992.1)	15.46	403166-403220	1	sugar ABC transporter substrate-binding protein (B7495_RS01925)	52
Cryptobacterium curtum DSM 15641 (NC_013170.1)	18.86	1081472-1081416	-1	glucan-binding protein (CCUR_RS04640)	18
	18.81	205749-205805	1	glucan-binding protein (CCUR_RS00825)	18
Curtobacterium pusillum strain AA3 (NZ_CP018783.1)	15.84	3814213-3814266	1	peptide ABC transporter substrate-binding protein (BUE88_RS18120)	14
Curtobacterium sp. MR_MD2014 (NZ_CP009755.1)	18.57	210772-210718	-1	gfo/Idh/MocA family oxidoreductase (NI26_RS01060)	7

Organism (Genome ID)	Bit score	ANTAR-target RNA co-ordinates	Strand	Annotation of the gene linked to ANTAR-target RNAs (Locus Tag)	Distance (nt) of RNA from gene linked to ANTAR-target RNA
	16.03	27231-27283	1	sugar phosphate isomerase/epimerase (NI26_RS00130)	5
Cutibacterium acnes strain KCOM 1861 (= ChDC B594) (NZ_CP012647.1)	19.1	246678-246614	-1	hypothetical protein (RN83_RS01045)	45
Cutibacterium avidum 44067 (NC_021064.1)	20.99	2362551-2362500	-1	LacI family transcriptional regulator (PALO_RS10760)	45
Cutibacterium avidum strain DPC 6544 (NZ_CP016954.1)	17.48	1895877-1895946	1	hypothetical protein (BFS79_RS08475)	30
	15.35	2604770-2604819	1	endo-beta-N-acetylglucosaminidase (BFS79_RS11695)	17
	15.21	1301579-1301520	-1	WhiB family transcriptional regulator (BFS79_RS05970)	292
Cutibacterium granulosum strain NCTC11865 1 (NZ_LT906441.1)	19.21	1211974-1212044	1	putative lysophospholipase (CKV91_RS05110)	23
Denitrobacterium detoxificans strain NPOH1 (NZ_CP011402.1)	17.65	1421804-1421754	-1	3-methyl-2-oxobutanoate dehydrogenase subunit VorB (AAY81_RS06090)	5
	15.74	1266680-1266733	1	ribonuclease HII (AAY81_RS05420)	63
Dermabacter vaginalis strain AD1-86 (NZ_CP012117.1)	20.61	21017-21071	1	restriction endonuclease subunit S (DAD186_RS10515)	26
	15.6	2034083-2034033	-1	cytoplasmic protein (DAD186_RS08980)	6
Dermacoccus nishinomiyaensis strain M25 (NZ_CP008889.1)	15.87	2422894-2422838	-1	TetR/AcrR family transcriptional regulator (HX89_RS10945)	23
Dermatophilus congolensis strain NCTC13039 1 (NZ_LT906453.1)	15.04	256097-256134	1	citrate synthase (CKV89_RS01045)	15
Eggerthella lenta DSM 2243 (NC_013204.1)	15.58	1642309-1642246	-1	XRE family transcriptional regulator (ELEN_RS06870)	234
	15.47	3050747-3050800	1	hypothetical protein (ELEN_RS13155)	70

Organism (Genome ID)	Bit score	ANTAR-target RNA co-ordinates	Strand	Annotation of the gene linked to ANTAR-target RNAs (Locus Tag)	Distance (nt) of RNA from gene linked to ANTAR-target RNA
	15.09	1311510-1311568	1	GGDEF domain-containing protein (ELEN_RS05405)	68
Eggerthella lenta strain C592 (NZ_CP021140.1)	15.53	2885114-2885061	-1	HNH endonuclease (CAB18_RS12525)	29
	15.09	2331573-2331515	-1	GGDEF domain-containing protein (CAB18_RS10165)	68
Eggerthella sp. YY7918 DNA (NC_015738.1)	23.92	2403484-2403432	-1	formate-dependent nitrite reductase (EGYY_RS10130)	141
	18.25	898918-898968	1	FAD-binding protein (EGYY_RS03605)	408
	16.05	2989223-2989276	1	serine/threonine-protein phosphatase (EGYY_RS12650)	11
Eggerthellaceae bacterium Marseille-P3241 contig00001 (NZ_LT821227.1)	17.89	813267-813313	1	hypothetical protein (B5449_RS03580)	28
	17.02	201358-201305	-1	translation initiation factor IF-3 (B5449_RS00945)	98
	16.58	1387731-1387665	-1	hypothetical protein (B5449_RS06055)	281
Frankia symbiont of Datisca glomerata (NC_015656.1)	16.08	3985397-3985335	-1	ATPase (FSYMDG_RS16640)	17
Frondihabitans sp. PAMC28766 (NZ_CP014513.1)	22.62	1628207-1628135	-1	Alpha-L-arabinofuranosidase (AX769_RS07985)	7
	19.17	4176083-4176027	-1	alpha-L-arabinofuranosidase (AX769_RS19925)	7
	15.88	210467-210404	-1	hypothetical protein (AX769_RS00955)	17
	15.34	1468942-1468876	-1	nitronate monooxygenase (AX769_RS07210)	4
Gardnerella vaginalis ATCC 14018 = JCM 11026 DNA (NZ_AP012332.1)	20.55	713559-713494	-1	MFS transporter (GAVG_RS02705)	39
	17.78	852571-852627	1	elongation factor Ts (GAVG_RS03270)	58
	17.14	310381-310435	1	ATPase (GAVG_RS01165)	22

Organism (Genome ID)	Bit score	ANTAR-target RNA co-ordinates	Strand	Annotation of the gene linked to ANTAR-target RNAs (Locus Tag)	Distance (nt) of RNA from gene linked to ANTAR-target RNA
	16.71	84407-84463	1	LacI family transcriptional regulator (GAVG_RS00345)	107
	16.53	1391978-1391921	-1	hypothetical protein (GAVG_RS05255)	6
	16.15	351111-351171	1	glycosyl transferase (GAVG_RS01325)	34
Gardnerella vaginalis ATCC 14019 (NC_014644.1)	20.55	852666-852601	-1	L-fucose permease (HMPREF0421_20693)	39
	17.78	991686-991742	1	elongation factor EF1B (HMPREF0421_20808)	58
	17.14	449487-449541	1	ribulokinase (HMPREF0421_20372)	22
	16.71	223569-223625	1	hypothetical protein (HMPREF0421_20197)	107
	16.53	1531081-1531024	-1	putative cytoplasmic protein (HMPREF0421_21236)	6
	16.15	490215-490275	1	group 2 glycosyl transferase (HMPREF0421_20405)	34
	15.95	1488885-1488834	-1	ABC transporter ATP-binding protein (HMPREF0421_21220)	91
Gardnerella vaginalis HMP9231 (NC_017456.1)	20.55	1045532-1045597	1	MFS transporter (HMPREF9231_RS04225)	39
	17.78	909641-909585	-1	elongation factor Ts (HMPREF9231_RS03690)	58
	17.14	1437267-1437213	-1	ATPase (HMPREF9231_RS05710)	22
	16.71	86028-86084	1	LacI family DNA-binding transcriptional regulator (HMPREF9231_RS00350)	107
	16.42	324254-324308	1	cytoplasmic protein (HMPREF9231_RS01430)	9
	16.15	1396491-1396431	-1	glycosyltransferase	34

Organism (Genome ID)	Bit score	ANTAR-target RNA co-ordinates	Strand	Annotation of the gene linked to ANTAR-target RNAs (Locus Tag)	Distance (nt) of RNA from gene linked to ANTAR-target RNA
				(HMPREF9231_RS05545)	
Glutamicibacter halophytocola strain KLBMP 5180 (NZ_CP012750.1)	17.99	3057388-3057338	-1	LacI family transcriptional regulator (AOZ07_RS14130)	13
	17.72	1968178-1968238	1	hypothetical protein (AOZ07_RS18180)	23
	17.39	1820450-1820512	1	DNA-binding response regulator (AOZ07_RS08370)	36
	17	2510824-2510880	1	hypothetical protein (AOZ07_RS11560)	277
	15.02	2883212-2883156	-1	spermidine/putrescine ABC transporter substrate-binding protein (AOZ07_RS13370)	434
Gordonia bronchialis DSM 43247 (NC_013441.1)	19.06	3539668-3539601	-1	methyltransferase domain-containing protein (GBRO_RS16465)	181
	18.98	5147979-5147941	-1	ammonium transporter (GBRO_RS23835)	57
	16.24	1890303-1890247	-1	aldehyde oxidase (GBRO_RS08895)	33
Gordonia phthalatica strain QH-11 (NZ_CP011853.1)	19.34	1991447-1991503	1	DUF4193 domain-containing protein (ACH46_RS09315)	70
Gordonia sp. KTR9 plasmid pGKT2 (NC_018580.1)	15.28	9243-9187	-1	hypothetical protein (KTR9_RS00050)	7
Gordonia sp. KTR9 (NC_018581.1)	15.84	5108419-5108476	1	DUF2236 domain-containing protein (KTR9_RS23580)	23
	15.42	5174857-5174799	-1	hypothetical protein (KTR9_RS23900)	11
Gordonibacter sp. Marseille-P2775 contig00001 (NZ_LT827128.1)	18.89	2127191-2127244	1	DNA-binding response regulator (B6F40_RS08985)	79
Gordonibacter urolithinfaciens strain DSM 27213T (NZ_LT900217.1)	16.13	820786-820735	-1	hypothetical protein (BN3560_RS03630)	489
	15.77	2091859-2091910	1	co-chaperone GroES (BN3560_RS08960)	35
	15.58	2055612-2055559	-1	DNA-binding response regulator (BN3560_RS08800)	79

Organism (Genome ID)	Bit score	ANTAR-target RNA co-ordinates	Strand	Annotation of the gene linked to ANTAR-target RNAs (Locus Tag)	Distance (nt) of RNA from gene linked to ANTAR-target RNA
Hoyosella subflava DQS3-9A1 (NC_015564.1)	31.41	4535832-4535773	-1	DUF2236 domain-containing protein (AS9A_RS21055)	5
	22.4	650876-650926	1	alpha/beta hydrolase (AS9A_RS02995)	188
	15.5	932765-932832	1	lipid-transfer protein (AS9A_RS04405)	17
Intrasporangium calvum DSM 43043 (NC_014830.1)	16.22	22911-22856	-1	hypothetical protein (INTCA_RS00110)	23
Janibacter indicus strain YFY001 (NZ_CP013290.1)	16.96	2789103-2789043	-1	hypothetical protein (ASJ30_RS13585)	5
Jonesia denitrificans DSM 20603 (NC_013174.1)	16.56	2323554-2323610	1	arabinogalactan endo-1,4-beta-galactosidase (JDEN_RS10685)	26
	16.15	2592010-2592060	1	anti-sigma factor antagonist (JDEN_RS12130)	428
	15.58	71727-71779	1	DeoR/GlpR transcriptional regulator (JDEN_RS00320)	13
	15.25	558606-558552	-1	sugar ABC transporter substrate-binding protein (JDEN_RS02550)	96
	15.14	144170-144235	1	energy-coupling factor ABC transporter ATP-binding protein (JDEN_RS00660)	76
Jonesia denitrificans strain FDAARGOS_301 (NZ_CP022038.1)	16.56	1554067-1554011	-1	arabinogalactan endo-1,4-beta-galactosidase (CEP80_RS07685)	26
	16.15	1246455-1246405	-1	anti-sigma factor antagonist (CEP80_RS05920)	428
	15.58	1017104-1017052	-1	DeoR/GlpR transcriptional regulator (CEP80_RS04895)	13
	15.25	530242-530296	1	sugar ABC transporter substrate-binding protein (CEP80_RS02640)	96
	15.14	944662-944597	-1	energy-coupling factor ABC transporter ATP-binding protein (CEP80_RS04550)	76

Organism (Genome ID)	Bit score	ANTAR-target RNA co-ordinates	Strand	Annotation of the gene linked to ANTAR-target RNAs (Locus Tag)	Distance (nt) of RNA from gene linked to ANTAR-target RNA
Kitasatospora albolonga strain YIM 101047 (NZ_CP020563.1)	15.64	1997191-1997129	-1	DUF3052 domain-containing protein (B7C62_RS08665)	82
Kitasatospora aureofaciens strain DM-1 (NZ_CP020567.1)	16.58	17269-17329	1	hypothetical protein (B6264_RS00060)	14
	16.54	651063-651111	1	sugar ABC transporter substrate-binding protein (B6264_RS02840)	53
	15.47	6756365-6756418	1	MarR family transcriptional regulator (B6264_RS29625)	449
Kitasatospora setae KM-6054 DNA (NC_016109.1)	15.36	778009-778069	1	carbohydrate-binding protein (KSE_RS03350)	186
Kocuria flava strain HO-9041 (NZ_CP013254.1)	19.86	515543-515592	1	hypothetical protein (AS188_RS02400)	180
Kribbella flavida DSM 17836 (NC_013729.1)	20.29	2485796-2485859	1	ricin B lectin (KFLA_RS11790)	53
Kutzneria albida DSM 43870 (NZ_CP007155.1)	25.05	7090364-7090418	1	PucR family transcriptional regulator (KALB_RS31050)	66
Lawsonella clevelandensis strain X1698 (NZ_CP012390.1)	25.41	604102-604046	-1	50S ribosomal protein L13 (AL705_RS02690)	277
	15.58	1356990-1357058	1	DNA polymerase III subunit delta' (AL705_RS05785)	14
Leifsonia xyli strain SE134 (NZ_CP014761.1)	30.35	2225649-2225592	-1	hypothetical protein (A0130_RS10910)	12
Leifsonia xyli subsp. cynodontis DSM 46306 (NC_022438.1)	19.15	1814548-1814589	1	sugar porter family MFS transporter (O159_RS08685)	2
Lentzea guizhouensis strain DHS C013 (NZ_CP016793.1)	16.42	7916418-7916360	-1	hypothetical protein (BBK82_RS38035)	156
	15.85	824349-824401	1	hypothetical protein (BBK82_RS48620)	17
Luteipulveratus mongoliensis strain MN07-A0370 genome (NZ_CP011112.1)	16.66	3020465-3020530	1	30S ribosomal protein S1 (VV02_RS14365)	79

Organism (Genome ID)	Bit score	ANTAR-target RNA co-ordinates	Strand	Annotation of the gene linked to ANTAR-target RNAs (Locus Tag)	Distance (nt) of RNA from gene linked to ANTAR-target RNA
Microbacterium aurum strain KACC 15219 (NZ_CP018762.1)	16.55	817552-817601	1	TetR/AcrR family transcriptional regulator (BOH66_RS04080)	59
	15.44	3112412-3112459	1	hypothetical protein (BOH66_RS14995)	0
Microbacterium paludicola strain CC3 (NZ_CP018134.1)	17.47	2473648-2473705	1	carbohydrate-binding protein (BO218_RS11725)	17
	15.11	2373937-2373993	1	sugar ABC transporter substrate-binding protein (BO218_RS11280)	4
Microbacterium sp. 1.5R (NZ_CP018151.1)	18.61	1648165-1648105	-1	glycerol kinase (BMW26_RS07710)	9
	16.89	3238250-3238196	-1	sugar ABC transporter substrate-binding protein (BMW26_RS15470)	26
	15.68	3018889-3018838	-1	hypothetical protein (BMW26_RS14505)	33
Microbacterium sp. BH-3-3-3 (NZ_CP017674.1)	15.95	3091177-3091233	1	DUF485 domain-containing protein (BJP65_RS14290)	7
	15.89	2563384-2563433	1	LacI family DNA-binding transcriptional regulator (BJP65_RS11740)	31
Microbacterium sp. CGR1 (NZ_CP012299.1)	18.61	661170-661110	-1	aquaporin family protein (AKG07_RS03080)	9
	16.89	2218112-2218058	-1	sugar ABC transporter substrate-binding protein (AKG07_RS10695)	26
Microbacterium sp. PAMC 28756 (NZ_CP014313.1)	17.56	1226223-1226163	-1	hypothetical protein (AXH82_RS05985)	17
Microbacterium sp. TPU 3598 DNA (NZ_AP017975.1)	18.1	1202932-1202999	1	hypothetical protein (MST3598_RS05635)	45
	16.19	3509660-3509602	-1	sugar ABC transporter ATP-binding protein (MST3598_RS16730)	17
Microbacterium sp. XT11 (NZ_CP013859.1)	17.63	1072688-1072639	-1	ABC transporter substrate-binding protein (AB663_RS05085)	5
	15.67	175856-175802	-1	maltose ABC transporter substrate-binding	39

Organism (Genome ID)	Bit score	ANTAR-target RNA co-ordinates	Strand	Annotation of the gene linked to ANTAR-target RNAs (Locus Tag)	Distance (nt) of RNA from gene linked to ANTAR-target RNA
				protein (AB663_RS00910)	
Microbacterium testaceum StLB037 DNA (NC_015125.1)	18.6	3169543-3169487	-1	transcriptional regulator (MTES_RS14400)	138
Microterricola viridarii strain ERGS5:02 (NZ_CP014145.1)	17.93	3382572-3382517	-1	sugar phosphate isomerase/epimerase (AWU67_RS15480)	17
	17.44	2717332-2717386	1	hypothetical protein (AWU67_RS12465)	2
	17.29	15867-15801	-1	GntR family transcriptional regulator (AWU67_RS00070)	214
	15.34	2872238-2872189	-1	FAD-dependent oxidoreductase (AWU67_RS13115)	8
	15.3	710315-710365	1	hypothetical protein (AWU67_RS03160)	266
Mobiluncus curtisii ATCC 43063 (NC_014246.1)	20.99	26853-26799	-1	succinate-semialdehyde dehydrogenase (NADP(+)) (HMPREF0573_RS00115)	5
	16.49	1518925-1518981	1	50S ribosomal protein L19 (HMPREF0573_RS06470)	48
	15.62	765509-765457	-1	hypothetical protein (HMPREF0573_RS03280)	37
	15.54	1880212-1880265	1	response regulator (HMPREF0573_RS08165)	94
Mycobacterium abscessus (NC_010397.1)	15.93	1789584-1789640	1	Bacteriophage protein (MAB_1787)	81
Mycobacterium abscessus strain FLAC005 (NZ_CP014952.1)	17.3	3098053-3097993	-1	malate:quinone oxidoreductase (A3N97_RS15190)	311
Mycobacterium abscessus strain FLAC013 (NZ_CP014955.1)	15.93	1772773-1772829	1	hypothetical protein (A3O00_RS08870)	81
Mycobacterium abscessus subsp. bolletii 103 (NZ_CP009407.1)	15.93	1774108-1774164	1	hypothetical protein (LA61_RS08890)	81

Organism (Genome ID)	Bit score	ANTAR-target RNA co-ordinates	Strand	Annotation of the gene linked to ANTAR-target RNAs (Locus Tag)	Distance (nt) of RNA from gene linked to ANTAR-target RNA
Mycobacterium abscessus subsp. bolletii 50594 plasmid 2 (NC_021279.1)	16.96	64917-64969	1	hypothetical protein (MASS_RS26665)	100
Mycobacterium abscessus subsp. bolletii strain MA 1948 (NZ_CP009408.1)	15.93	1789073-1789129	1	hypothetical protein (LA62_RS08980)	81
Mycobacterium abscessus subsp. bolletii strain MC1518 (NZ_CP009613.1)	15.93	1781082-1781138	1	hypothetical protein (NF82_RS08935)	81
Mycobacterium africanum GM041182 complete genome (NC_015758.1)	24.77	2187796-2187846	1	monooxygenase (MAF_RS10120)	73
	16.73	183316-183369	1	NAD(P) transhydrogenase subunit beta (MAF_RS00845)	36
Mycobacterium avium subsp. paratuberculosis MAP4 (NC_021200.1)	15.81	2321644-2321703	1	TetR family transcriptional regulator (MAP4_RS10515)	102
Mycobacterium avium subsp. paratuberculosis str. k10 (NC_002944.2)	15.81	1887618-1887559	-1	TetR family transcriptional regulator (MAP_RS08785)	102
Mycobacterium avium subsp. paratuberculosis strain E1 (NZ_CP010113.1)	15.81	2295420-2295479	1	TetR family transcriptional regulator (RC58_RS10490)	102
Mycobacterium avium subsp. paratuberculosis strain E93 (NZ_CP010114.1)	15.81	2298051-2298110	1	TetR family transcriptional regulator (RE97_RS10500)	102
Mycobacterium avium subsp. paratuberculosis strain MAP/TANUVAS/TN/India/2008 (NZ_CP015495.1)	15.81	1887618-1887559	-1	TetR family transcriptional regulator (A0V42_RS08815)	102
Mycobacterium bovis BCG Pasteur	24.77	2189421-2189471	1	monooxygenase (BCG_RS10150)	73

Organism (Genome ID)	Bit score	ANTAR-target RNA co-ordinates	Strand	Annotation of the gene linked to ANTAR-target RNAs (Locus Tag)	Distance (nt) of RNA from gene linked to ANTAR-target RNA
1173P2 (NC_008769.1)	16.73	214866-214919	1	NAD(P) transhydrogenase subunit beta (BCG_RS01000)	36
Mycobacterium bovis BCG str. ATCC 35743 (NZ_CP003494.1)	24.77	1889021-1889071	1	monooxygenase (BCGT_RS08920)	73
	16.73	4234506-4234559	1	NAD(P) transhydrogenase subunit beta (BCGT_RS19940)	36
Mycobacterium bovis BCG str. Korea 1168P (NC_020245.2)	24.77	2159066-2159116	1	monooxygenase (K60_RS09975)	73
	16.73	185199-185252	1	NAD(P) transhydrogenase subunit beta (K60_RS00840)	36
Mycobacterium bovis BCG str. Mexico (NC_016804.1)	24.77	2168786-2168836	1	monooxygenase (BCGMEX_RS10025)	73
	16.73	185199-185252	1	NAD(P) transhydrogenase subunit beta (BCGMEX_RS00840)	36
Mycobacterium bovis BCG str. Moreau RDJ complete genome (NZ_AM412059.1)	24.77	2171338-2171388	1	monooxygenase (BCGM_RS10045)	73
	16.73	185193-185246	1	NAD(P) transhydrogenase subunit beta (BCGM_RS00835)	36
Mycobacterium bovis BCG str. Tokyo 172 DNA (NC_012207.1)	24.77	2171412-2171462	1	monooxygenase (JTY_RS10050)	73
	16.73	185199-185252	1	NAD(P) transhydrogenase subunit beta (JTY_RS00845)	36
Mycobacterium bovis BCG str. Tokyo 172 substrain TRCS (NZ_CP014566.1)	24.77	2171410-2171460	1	monooxygenase (AZH48_RS10030)	73
	16.73	185199-185252	1	NAD(P) transhydrogenase subunit beta (AZH48_RS00835)	36
Mycobacterium bovis BCG strain 3281 (NZ_CP008744.1)	24.77	2169564-2169614	1	monooxygenase (GS11_RS10040)	73
	16.73	185198-185251	1	NAD(P) transhydrogenase subunit beta (GS11_RS00840)	36
Mycobacterium bovis BCG strain Russia 368 (NZ_CP009243.1)	24.77	2171415-2171465	1	monooxygenase (BCGR_RS10045)	73
	16.73	185196-185249	1	NAD(P) transhydrogenase subunit beta (BCGR_RS00835)	36

Organism (Genome ID)	Bit score	ANTAR-target RNA co-ordinates	Strand	Annotation of the gene linked to ANTAR-target RNAs (Locus Tag)	Distance (nt) of RNA from gene linked to ANTAR-target RNA
Mycobacterium bovis strain 1595 (NZ_CP012095.1)	24.77	2183860-2183910	1	monooxygenase (Mb1595_RS10130)	73
	16.73	185167-185220	1	NAD(P) transhydrogenase subunit beta (Mb1595_RS00840)	36
Mycobacterium bovis strain ATCC BAA-935 (NZ_CP009449.1)	24.77	2147621-2147671	1	monooxygenase (LH58_RS10065)	73
	16.73	184747-184800	1	NAD(P) transhydrogenase subunit beta (LH58_RS00850)	36
Mycobacterium bovis strain BCG-1 (Russia) (NZ_CP013741.1)	24.77	2171711-2171761	1	monooxygenase (BOVR_RS10050)	73
	16.73	185196-185249	1	NAD(P) transhydrogenase subunit beta (BOVR_RS00835)	36
Mycobacterium bovis strain SP38 (NZ_CP015773.1)	24.31	1085783-1085833	1	monooxygenase (SZ58_RS04940)	73
	16.73	3435305-3435358	1	NAD(P) transhydrogenase subunit beta (SZ58_RS15965)	36
Mycobacterium canettii CIPT 140010059 complete genome (NC_015848.1)	16.73	191637-191690	1	NAD(P) transhydrogenase subunit beta (MCAN_RS00870)	36
Mycobacterium chelonae CCUG 47445 (NZ_CP007220.1)	15.61	1991172-1991122	-1	leucyl aminopeptidase (BB28_RS09875)	335
Mycobacterium chubuense NBB4 (NC_018027.1)	15.84	2664513-2664572	1	30S ribosomal protein S1 (MYCCH_RS12620)	91
Mycobacterium fortuitum strain CT6 (NZ_CP011269.1)	20.51	6251576-6251522	-1	16S rRNA methyltransferase G (XA26_RS29760)	7
	19.98	4166635-4166579	-1	hypothetical protein (XA26_RS19840)	43
	19.55	3804230-3804294	1	hypothetical protein (XA26_RS18215)	17
	16.6	3617008-3616949	-1	30S ribosomal protein S1 (XA26_RS17315)	91
	15.11	444284-444224	-1	MFS transporter (XA26_RS02045)	72
Mycobacterium gilvum PYR-GCK	22.54	1209116-1209171	1	esterase/ diacyl-glycerol transferase	60

Organism (Genome ID)	Bit score	ANTAR-target RNA co-ordinates	Strand	Annotation of the gene linked to ANTAR-target RNAs (Locus Tag)	Distance (nt) of RNA from gene linked to ANTAR-target RNA
(NC_009338.1)				(MFLV_RS05805)	
	17.57	4909773-4909724	-1	acyl-CoA dehydrogenase (MFLV_RS23155)	23
	16.29	4195679-4195623	-1	dUTPase (MFLV_RS19775)	70
	16.22	3798413-3798354	-1	30S ribosomal protein S1 (MFLV_RS17880)	91
Mycobacterium gilvum Spyr1 (NC_014814.1)	22.54	5213367-5213312	-1	hypothetical protein (MSPYR1_RS24685)	60
	16.29	3405359-3405303	-1	dUTPase (MSPYR1_RS16135)	70
	16.22	3014743-3014684	-1	30S ribosomal protein S1 (MSPYR1_RS14285)	91
	15.78	3577814-3577873	1	aldehyde dehydrogenase (MSPYR1_RS17045)	40
Mycobacterium goodii strain X7B (NZ_CP012150.1)	16.25	5969358-5969296	-1	SAM-dependent methyltransferase (AFA91_RS27860)	99
Mycobacterium haemophilum DSM 44634 strain ATCC 29548 (NZ_CP011883.2)	16.19	408604-408663	1	hypothetical protein (B586_RS01890)	13
	15.56	1112421-1112495	1	hypothetical protein (B586_RS05315)	161
Mycobacterium immunogenum strain CCUG 47286 (NZ_CP011530.1)	17.47	4394659-4394597	-1	AsnC family transcriptional regulator (ABG82_RS21770)	389
	15.18	3148105-3148046	-1	transketolase (ABG82_RS15345)	2
Mycobacterium liflandii 128FXT (NC_020133.1)	20.47	3552989-3553040	1	hypothetical protein (MULP_RS15355)	70
	16.46	2276480-2276530	1	hypothetical protein (MULP_RS09850)	7
	15.34	3623588-3623652	1	TetR family transcriptional regulator (MULP_RS15640)	85
Mycobacterium marinum E11 main genome (NZ_HG917972.2)	20.47	2705668-2705617	-1	hypothetical protein (MMARE11_RS11245)	70
	16.54	2228845-2228895	1	hypothetical protein	7

Organism (Genome ID)	Bit score	ANTAR-target RNA co-ordinates	Strand	Annotation of the gene linked to ANTAR-target RNAs (Locus Tag)	Distance (nt) of RNA from gene linked to ANTAR-target RNA
				(MMARE11_RS09125)	
	15.34	2608576-2608512	-1	TetR family transcriptional regulator (MMARE11_RS10875)	85
Mycobacterium marinum M (NC_010612.1)	20.47	2842763-2842712	-1	hypothetical protein (MMAR_RS11735)	70
	16.46	2346618-2346668	1	hypothetical protein (MMAR_RS09565)	7
	15.34	2745480-2745416	-1	TetR family transcriptional regulator (MMAR_RS11360)	85
Mycobacterium neoaurum VKM Ac-1815D (NC_023036.2)	16.66	4181172-4181233	1	hypothetical protein (D174_RS19485)	63
	15.99	4248495-4248433	-1	F0F1 ATP synthase subunit C (D174_RS19810)	17
Mycobacterium phlei strain CCUG 21000 (NZ_CP014475.1)	16.65	3983075-3983127	1	DNA-3-methyladenine glycosylase (MPHLCCUG_RS19105)	16
	16.6	2811559-2811500	-1	30S ribosomal protein S1 (MPHLCCUG_RS13480)	91
Mycobacterium rhodesiae NBB3 (NC_016604.1)	16.99	4624964-4625023	1	30S ribosomal protein S1 (MYCRHN_RS22435)	91
	16.97	761121-761068	-1	DNA-directed RNA polymerase subunit beta' (MYCRHN_RS03650)	141
Mycobacterium smegmatis genome assembly NCTC81591 (NZ_LN831039.1)	16.41	3141723-3141672	-1	2,5-dichloro-2,5-cyclohexadiene-1,4-diol dehydrogenase (AT701_RS15105)	36
Mycobacterium smegmatis JS623 plasmid pMYCSM02 (NC_019958.1)	17.87	29809-29757	-1	hypothetical protein (MYCSM_RS33235)	385
Mycobacterium smegmatis JS623 (NC_019966.1)	17.36	2977710-2977769	1	30S ribosomal protein S1 (MYCSM_RS14600)	91
	15.17	21645-21589	-1	serine/threonine protein kinase (MYCSM_RS00115)	30

Organism (Genome ID)	Bit score	ANTAR-target RNA co-ordinates	Strand	Annotation of the gene linked to ANTAR-target RNAs (Locus Tag)	Distance (nt) of RNA from gene linked to ANTAR-target RNA
Mycobacterium smegmatis str. MC2 155 (NC_008596.1)	16.41	3078808-3078757	-1	2,5-dichloro-2,5-cyclohexadiene-1,4-diol dehydrogenase (MSMEG_3009)	36
Mycobacterium smegmatis str. MC2 155 (NC_018289.1)	16.41	3080422-3080371	-1	2,5-dichloro-2,5-cyclohexadiene-1,4-diol dehydrogenase (MSMEI_RS14815)	36
Mycobacterium smegmatis str. MC2 155 (NZ_CP009494.1)	16.41	3078853-3078802	-1	2,5-dichloro-2,5-cyclohexadiene-1,4-diol dehydrogenase (LJ00_RS14800)	36
Mycobacterium smegmatis strain INHR1 (NZ_CP009495.1)	16.41	3078887-3078836	-1	2,5-dichloro-2,5-cyclohexadiene-1,4-diol dehydrogenase (LI99_RS14800)	36
Mycobacterium smegmatis strain INHR2 (NZ_CP009496.1)	16.41	3078875-3078824	-1	2,5-dichloro-2,5-cyclohexadiene-1,4-diol dehydrogenase (LI98_RS14795)	36
Mycobacterium sp. EPa45 (NZ_CP011773.1)	17.68	3780340-3780281	-1	30S ribosomal protein S1 (AB431_RS18105)	91
	16.35	4094521-4094465	-1	hypothetical protein (AB431_RS19610)	87
	16.22	572894-572950	1	hypothetical protein (AB431_RS02575)	88
	15.06	5271360-5271312	-1	aminoglycoside phosphotransferase (AB431_RS24895)	3
Mycobacterium sp. JLS (NC_009077.1)	19.01	2700344-2700396	1	ATPase AAA (MJLS_RS12880)	9
	16.1	3939077-3939026	-1	(2Fe-2S)-binding protein (MJLS_RS18635)	133
	15.84	3157092-3157033	-1	30S ribosomal protein S1 (MJLS_RS15115)	91
	15.78	1201711-1201770	1	HAD family hydrolase (MJLS_RS05610)	7
	15.33	1406291-1406347	1	alpha/beta hydrolase (MJLS_RS06560)	11
Mycobacterium sp. KMS (NC_008705.1)	19.01	2714603-2714655	1	ATPase AAA (MKMS_RS12840)	9
	15.84	3195040-3194981	-1	30S ribosomal protein S1 (MKMS_RS15200)	91
	15.78	1198153-1198212	1	hypothetical protein (MKMS_RS05525)	7

Organism (Genome ID)	Bit score	ANTAR-target RNA co-ordinates	Strand	Annotation of the gene linked to ANTAR-target RNAs (Locus Tag)	Distance (nt) of RNA from gene linked to ANTAR-target RNA
Mycobacterium sp. MCS (NC_008146.1)	19.01	2696715-2696767	1	ATPase AAA (MMCS_RS12745)	9
	15.84	3177152-3177093	-1	30S ribosomal protein S1 (MMCS_RS15105)	91
	15.78	1193844-1193903	1	hypothetical protein (MMCS_RS05500)	7
Mycobacterium sp. NRRL B-3805 (NZ_CP011022.1)	16.66	4181243-4181304	1	hypothetical protein (MyAD_RS19495)	63
	15.99	4248566-4248504	-1	F0F1 ATP synthase subunit C (MyAD_RS19820)	17
Mycobacterium sp. QIA-37 (NZ_CP010071.1)	18.38	1151605-1151667	1	cytochrome (Chelonae_RS05625)	91
Mycobacterium sp. VKM Ac-1817D (NZ_CP009914.1)	20.51	6321182-6321128	-1	16S rRNA methyltransferase G (G155_RS30185)	7
	18.79	5424084-5424140	1	hypothetical protein (G155_RS25930)	64
	16.6	3626181-3626122	-1	30S ribosomal protein S1 (G155_RS17390)	91
	15.11	515037-514977	-1	MFS transporter (G155_RS02340)	72
Mycobacterium tuberculosis 49-02 complete genome (NZ_HG813240.1)	24.77	2173566-2173616	1	alkane 1-monooxygenase (MT49_RS10070)	73
	16.73	183230-183283	1	NAD(P) transhydrogenase subunit beta (MT49_RS00840)	36
Mycobacterium tuberculosis 7199-99 complete genome (NC_020089.1)	24.77	2191699-2191749	1	monooxygenase (MT7199_RS10100)	73
	16.73	185188-185241	1	NAD(P) transhydrogenase subunit beta (MT7199_RS00840)	36
Mycobacterium tuberculosis BT1 (NZ_CP002883.1)	24.77	2173137-2173187	1	alkane 1-monooxygenase (HKBT1_RS10085)	73
	16.73	183287-183340	1	NAD(P) transhydrogenase subunit beta (HKBT1_RS00840)	36
Mycobacterium tuberculosis BT2	24.77	2166350-2166400	1	alkane 1-monooxygenase	73

Organism (Genome ID)	Bit score	ANTAR-target RNA co-ordinates	Strand	Annotation of the gene linked to ANTAR-target RNAs (Locus Tag)	Distance (nt) of RNA from gene linked to ANTAR-target RNA
(NZ_CP002882.1)				(HKBT2_RS10060)	
	16.73	183286-183339	1	NAD(P) transhydrogenase subunit beta (HKBT2_RS00840)	36
Mycobacterium tuberculosis CCDC5079 (NC_021251.1)	20.33	2174956-2175006	1	alkane 1-monooxygenase (CFBS_RS10115)	73
	16.73	183231-183284	1	NAD(P) transhydrogenase subunit beta (CFBS_RS00840)	36
Mycobacterium tuberculosis CCDC5180 (NC_017522.1)	24.77	2168763-2168813	1	alkane 1-monooxygenase (CCDC5180_RS10115)	73
	16.73	183230-183283	1	NAD(P) transhydrogenase subunit beta (CCDC5180_RS00840)	36
Mycobacterium tuberculosis CCDC5180 (NZ_CP002885.1)	24.77	2173333-2173383	1	alkane 1-monooxygenase (CFBR_RS10085)	73
	16.73	183230-183283	1	NAD(P) transhydrogenase subunit beta (CFBR_RS00840)	36
Mycobacterium tuberculosis CDC1551 (NC_002755.2)	16.73	185137-185190	1	NAD(P) transhydrogenase subunit beta (MT_RS00840)	36
	15.69	2184593-2184643	1	monooxygenase (MT_RS10100)	73
Mycobacterium tuberculosis CTRI-2 (NC_017524.1)	24.77	2180824-2180874	1	monooxygenase (MTCTRI2_RS10125)	73
	16.73	184725-184778	1	NAD(P) transhydrogenase subunit beta (MTCTRI2_RS00845)	36
Mycobacterium tuberculosis EAI5 (NC_021740.1)	24.77	2181844-2181894	1	monooxygenase (M943_RS10115)	73
	16.73	184973-185026	1	NAD(P) transhydrogenase subunit beta (M943_RS00845)	36
Mycobacterium tuberculosis EAI5/NITR206 (NC_021194.1)	24.77	2181480-2181530	1	monooxygenase (J114_RS10135)	73

Organism (Genome ID)	Bit score	ANTAR-target RNA co-ordinates	Strand	Annotation of the gene linked to ANTAR-target RNAs (Locus Tag)	Distance (nt) of RNA from gene linked to ANTAR-target RNA
Mycobacterium tuberculosis F11 (NC_009565.1)	24.77	2195516-2195566	1	monooxygenase (TBFG_RS10180)	73
	16.73	185285-185338	1	NAD(P) transhydrogenase subunit beta (TBFG_RS00840)	36
Mycobacterium tuberculosis H37Ra (NC_009525.1)	24.77	2197160-2197210	1	monooxygenase (MRA_RS10230)	73
	16.73	186329-186382	1	NAD(P) transhydrogenase subunit beta (MRA_RS00855)	36
Mycobacterium tuberculosis H37Rv (NC_000962.3)	24.77	2187261-2187311	1	monooxygenase (Rv1936)	73
	16.73	184969-185022	1	NAD(P) transhydrogenase subunit beta PntB (Rv0157)	30
Mycobacterium tuberculosis H37Rv (NC_018143.2)	24.77	2187268-2187318	1	monooxygenase (RVBD_RS10160)	73
	16.73	184967-185020	1	NAD(P) transhydrogenase subunit beta (RVBD_RS00840)	36
Mycobacterium tuberculosis H37Rv (NZ_CP009480.1)	24.77	2182565-2182615	1	monooxygenase (LH57_RS10210)	73
	16.73	184815-184868	1	NAD(P) transhydrogenase subunit beta (LH57_RS00845)	36
Mycobacterium tuberculosis H37RvSiena (NZ_CP007027.1)	24.77	2186649-2186699	1	monooxygenase (Y980_RS10165)	73
	16.73	184967-185020	1	NAD(P) transhydrogenase subunit beta (Y980_RS00840)	36
Mycobacterium tuberculosis HKBS1 (NZ_CP002871.1)	24.77	2172407-2172457	1	alkane 1-monooxygenase (HKBS1_RS10075)	73
	16.73	183231-183284	1	NAD(P) transhydrogenase subunit beta (HKBS1_RS00840)	36
Mycobacterium tuberculosis K (NZ_CP007803.1)	20.21	2159806-2159856	1	alkane 1-monooxygenase (MTBK_RS09995)	73
	16.73	183165-183218	1	NAD(P) transhydrogenase subunit beta (MTBK_RS00840)	36
Mycobacterium tuberculosis KZN	24.77	2235812-2235762	-1	monooxygenase (TBMG_RS10750)	73

Organism (Genome ID)	Bit score	ANTAR-target RNA co-ordinates	Strand	Annotation of the gene linked to ANTAR-target RNAs (Locus Tag)	Distance (nt) of RNA from gene linked to ANTAR-target RNA
1435 (NC_012943.1)	16.73	184908-184961	1	NAD(P) transhydrogenase subunit beta (TBMG_RS00845)	36
Mycobacterium tuberculosis KZN 4207 (NC_016768.1)	24.77	2232543-2232493	-1	monooxygenase (TBSG_RS10740)	73
	16.73	184907-184960	1	NAD(P) transhydrogenase subunit beta (TBSG_RS00845)	36
Mycobacterium tuberculosis KZN 605 (NC_018078.1)	24.77	2235693-2235643	-1	monooxygenase (TBXG_RS10755)	73
	16.73	184906-184959	1	NAD(P) transhydrogenase subunit beta (TBXG_RS00845)	36
Mycobacterium tuberculosis str. Beijing/NITR203 (NC_021054.1)	24.99	2187067-2187117	1	monooxygenase (J112_RS10210)	73
	16.73	184956-185009	1	NAD(P) transhydrogenase subunit beta (J112_RS00850)	36
Mycobacterium tuberculosis str. Erdman = ATCC 35801 DNA (NC_020559.1)	24.77	2178334-2178384	1	monooxygenase (ERDMAN_RS10140)	73
	16.73	184282-184335	1	NAD(P) transhydrogenase subunit beta (ERDMAN_RS00840)	36
Mycobacterium tuberculosis str. Haarlem (NC_022350.1)	24.77	2186892-2186942	1	monooxygenase (TBHG_RS10090)	73
	16.73	185287-185340	1	NAD(P) transhydrogenase subunit beta (TBHG_RS00840)	36
Mycobacterium tuberculosis str. Kurono DNA (NZ_AP014573.1)	24.77	2192698-2192748	1	monooxygenase (KURONO_RS10185)	73
	16.73	186176-186229	1	NAD(P) transhydrogenase subunit beta (KURONO_RS00850)	36
Mycobacterium tuberculosis strain 22103 (NZ_CP010339.1)	24.77	2185332-2185382	1	monooxygenase (RN14_RS10075)	73
	16.73	185184-185237	1	NAD(P) transhydrogenase subunit beta (RN14_RS00840)	36
Mycobacterium tuberculosis strain 22115 (NZ_CP010337.1)	24.77	2190192-2190242	1	monooxygenase (RN12_RS10150)	73
	16.73	185165-185218	1	NAD(P) transhydrogenase subunit beta (RN12_RS00835)	36

Organism (Genome ID)	Bit score	ANTAR-target RNA co-ordinates	Strand	Annotation of the gene linked to ANTAR-target RNAs (Locus Tag)	Distance (nt) of RNA from gene linked to ANTAR-target RNA
Mycobacterium tuberculosis strain 26105 (NZ_CP010340.1)	24.77	2199709-2199759	1	alkane 1-monooxygenase (RN15_RS10215)	73
	16.73	185418-185471	1	NAD(P) transhydrogenase subunit beta (RN15_RS00835)	36
Mycobacterium tuberculosis strain 37004 (NZ_CP010338.1)	24.77	2182762-2182812	1	monooxygenase (RN13_RS10115)	73
	16.73	185172-185225	1	NAD(P) transhydrogenase subunit beta (RN13_RS00835)	36
Mycobacterium tuberculosis strain 96075 (NZ_CP009426.1)	24.77	2167819-2167869	1	alkane 1-monooxygenase (LJ70_RS10085)	73
	16.73	182314-182367	1	NAD(P) transhydrogenase subunit beta (LJ70_RS00840)	36
Mycobacterium tuberculosis strain 96121 (NZ_CP009427.1)	24.77	2195453-2195503	1	monooxygenase (HR37_RS10225)	73
	16.73	185032-185085	1	NAD(P) transhydrogenase subunit beta (HR37_RS00845)	36
Mycobacterium tuberculosis strain F28 (NZ_CP010330.1)	24.77	2194218-2194268	1	monooxygenase (RN05_RS10215)	73
	16.73	184967-185020	1	NAD(P) transhydrogenase subunit beta (RN05_RS00835)	36
Mycobacterium tuberculosis strain KIT87190 (NZ_CP007809.1)	24.77	2166335-2166385	1	alkane 1-monooxygenase (ER17_RS10060)	73
	16.73	182372-182425	1	NAD(P) transhydrogenase subunit beta (ER17_RS00840)	36
Mycobacterium tuberculosis strain SCAID 187.0 (NZ_CP012506.1)	24.77	2160992-2161042	1	alkane 1-monooxygenase (AFL40_RS10020)	73
	16.73	181412-181465	1	NAD(P) transhydrogenase subunit beta (AFL40_RS00825)	36
Mycobacterium tuberculosis strain ZMC13-264 (NZ_CP009100.1)	24.77	2187257-2187307	1	alkane 1-monooxygenase (IZ84_RS10135)	73

Organism (Genome ID)	Bit score	ANTAR-target RNA co-ordinates	Strand	Annotation of the gene linked to ANTAR-target RNAs (Locus Tag)	Distance (nt) of RNA from gene linked to ANTAR-target RNA
	16.73	185023-185076	1	NAD(P) transhydrogenase subunit beta (IZ84_RS00845)	36
Mycobacterium tuberculosis strain ZMC13-88 (NZ_CP009101.1)	24.77	2187289-2187339	1	alkane 1-monooxygenase (JE53_RS10145)	73
	16.73	184988-185041	1	NAD(P) transhydrogenase subunit beta (JE53_RS00850)	36
Mycobacterium tuberculosis W-148 (NZ_CP012090.1)	24.77	1954309-1954359	1	alkane 1-monooxygenase (TBPG_RS08905)	73
	16.73	183231-183284	1	NAD(P) transhydrogenase subunit beta (TBPG_RS00835)	36
Mycobacterium vaccae 95051 (NZ_CP011491.1)	26.53	5794907-5794851	-1	esterase/ diacyl-glycerol transferase (MYVA_RS27135)	58
	16	3061381-3061433	1	acyl-CoA synthetase (MYVA_RS14355)	21
	15.36	505581-505518	-1	hypothetical protein (MYVA_RS02330)	7
Mycobacterium vanbaalenii PYR-1 (NC_008726.1)	30	6033960-6033904	-1	esterase/ diacyl-glycerol transferase (MVAN_RS28400)	61
	17.47	3573570-3573511	-1	30S ribosomal protein S1 (MVAN_RS16845)	91
	17.16	380445-380510	1	putative GAF sensor protein (MVAN_RS01770)	22
	15.13	2379314-2379255	-1	aldehyde dehydrogenase (MVAN_RS11160)	40
Neomicrococcus aestuarii strain B18 (NZ_CP018135.1)	19.92	1224527-1224475	-1	ABC transporter permease (BHE16_RS05425)	21
	17.46	1469870-1469937	1	hypothetical protein (BHE16_RS06505)	70
Nocardia brasiliensis ATCC 700358 (NC_018681.1)	16.52	466485-466426	-1	alpha/beta hydrolase (O3I_RS02080)	32
	16.04	3190100-3190039	-1	5-aminolevulinate synthase	148

Organism (Genome ID)	Bit score	ANTAR-target RNA co-ordinates	Strand	Annotation of the gene linked to ANTAR-target RNAs (Locus Tag)	Distance (nt) of RNA from gene linked to ANTAR-target RNA
				(O3I_RS14450)	
Nocardia brasiliensis strain FDAARGOS_352 (NZ_CP022088.1)	16.52	6086749-6086808	1	alpha/beta hydrolase (CEQ30_RS27470)	32
	15.19	3398943-3399004	1	5-aminolevulinate synthase (CEQ30_RS15060)	148
Nocardia cyriacigeorgica GUH-2 complete genome (NC_016887.1)	19.21	425232-425172	-1	alpha/beta hydrolase (NOCYR_RS01960)	32
	15.23	2129230-2129175	-1	alpha/beta hydrolase (NOCYR_RS09530)	1
Nocardia farcinica genome assembly NCTC11134, plasmid : 2 (NZ_LN868939.1)	15.62	363725-363784	1	alpha/beta hydrolase (AMO33_RS18820)	0
Nocardia farcinica IFM 10152 DNA (NC_006361.1)	17.88	389632-389573	-1	alpha/beta hydrolase (NFA_RS01905)	32
	15.47	4424930-4424995	1	hypothetical protein (NFA_RS30625)	58
Nocardia nova SH22a (NZ_CP006850.1)	20.73	7697964-7697910	-1	alpha/beta hydrolase (NONO_RS34805)	26
	15.5	7515908-7515859	-1	hypothetical protein (NONO_RS39425)	33
Nocardia seriolae DNA, strain: UTF1 (NZ_AP017900.1)	22.21	6584176-6584229	1	nitrate reductase molybdenum cofactor assembly chaperone (NSERUTF1_RS30720)	62
	19.19	552477-552424	-1	acetoin dehydrogenase dihydrolipoyllysine-residue acetyltransferase subunit (NSERUTF1_RS02545)	26
Nocardia seriolae DNA, strain: UTF1 (NZ_AP017900.1)	15.8	4227960-4227909	-1	cytochrome P450 (NSERUTF1_RS19455)	34
	15.75	5180104-5180039	-1	KR domain-containing protein (NSERUTF1_RS24110)	79
	15.72	957415-957359	-1	IS4 family transposase (NSERUTF1_RS04405)	398
Nocardia seriolae strain EM150506 (NZ_CP017839.1)	22.21	2300052-2299999	-1	nitrate reductase molybdenum cofactor assembly chaperone (NS506_RS10565)	62

Organism (Genome ID)	Bit score	ANTAR-target RNA co-ordinates	Strand	Annotation of the gene linked to ANTAR-target RNAs (Locus Tag)	Distance (nt) of RNA from gene linked to ANTAR-target RNA
	19.19	204678-204731	1	alpha/beta hydrolase (NS506_RS01055)	26
	15.8	4746174-4746123	-1	cytochrome P450 (NS506_RS21800)	34
	15.75	6052574-6052509	-1	KR domain-containing protein (NS506_RS28010)	79
Nocardia soli strain Y48 (NZ_CP018082.1)	15.77	376014-376071	1	hypothetical protein (BOX37_RS01685)	42
Nocardia terpenica strain NC_YFY_NT001 (NZ_CP023778.1)	17.03	7074978-7075034	1	xylose isomerase (CRH09_RS31310)	7
	16.58	8308608-8308556	-1	alpha/beta hydrolase (CRH09_RS37120)	26
Nonomuraea sp. ATCC 55076 (NZ_CP017717.1)	20	9630594-9630535	-1	DUF397 domain-containing protein (BKM31_RS43705)	1
	16.45	5653656-5653608	-1	sugar ABC transporter (BKM31_RS25565)	30
	16.07	4124855-4124911	1	CBS domain-containing protein (BKM31_RS18760)	79
Olsenella sp. oral taxon 807 strain F0089 (NZ_CP012069.2)	17.57	2778345-2778401	1	hypothetical protein (ADJ70_RS11980)	40
Olsenella uli DSM 7084 (NC_014363.1)	16.27	208494-208553	1	LysR family transcriptional regulator (OLSU_RS08990)	24
	15.29	264364-264414	1	Fic family protein (OLSU_RS01165)	57
Paenarthrobacter aurescens TC1 (NC_008711.1)	17.57	678612-678551	-1	MFS transporter (AAUR_RS03280)	83
	17.13	2184804-2184862	1	sugar ABC transporter substrate-binding protein (AAUR_RS09910)	88
	15.44	4054153-4054097	-1	LacI family transcriptional regulator (AAUR_RS18450)	420
	15.34	2273818-2273883	1	30S ribosomal protein S1 (AAUR_RS10315)	94
	16.11	2729216-2729267	1	hypothetical protein (BAY61_RS12710)	36

Organism (Genome ID)	Bit score	ANTAR-target RNA co-ordinates	Strand	Annotation of the gene linked to ANTAR-target RNAs (Locus Tag)	Distance (nt) of RNA from gene linked to ANTAR-target RNA
	15.6	5375237-5375179	-1	TetR/AcrR family transcriptional regulator (BAY61_RS24705)	17
Propionibacterium acnes ATCC 11828 (NC_017550.1)	19.1	1142938-1142874	-1	hypothetical protein (TIIST44_RS05220)	45
Propionibacterium acnes TypeIA2 P.acn17 (NC_016512.1)	19.1	146843-146779	-1	hypothetical protein (TIA2EST22_RS00625)	45
Propionibacterium acnes TypeIA2 P.acn31 (NC_016511.1)	19.1	146852-146788	-1	hypothetical protein (TIA2EST36_RS00620)	45
Propionibacterium acnes TypeIA2 P.acn33 (NC_016516.1)	19.1	146855-146791	-1	hypothetical protein (TIA2EST2_RS00620)	45
Propionibacterium acnes TypeIA2 P.acn33 (NC_016516.1)	15.02	1290367-1290317	-1	2-deoxyribose-5-phosphate aldolase (TIA2EST2_RS05990)	24
Propionibacterium freudenreichii strain P.UF1 (NZ_CP018002.1)	17.83	2035851-2035913	1	pyruvate oxidase (BMR99_RS09195)	44
	16.05	722929-722867	-1	methylmalonyl-CoA mutase small subunit (BMR99_RS03065)	224
	15.39	1543147-1543107	-1	transcriptional regulator (BMR99_RS06855)	8
	15.07	2595505-2595555	1	citrate synthase (BMR99_RS11540)	45
Propionibacterium freudenreichii subsp. freudenreichii strain DSM 20271 (NZ_CP010341.1)	17.83	510685-510623	-1	pyruvate oxidase (RM25_RS02000)	44
	16.05	852187-852125	-1	methylmalonyl-CoA mutase small subunit (RM25_RS03585)	224
	15.39	1613530-1613478	-1	transcriptional regulator (RM25_RS07000)	20
	15.07	2613515-2613565	1	citrate synthase (RM25_RS11475)	45
Propionibacterium freudenreichii subsp. shermanii CIRM-BIA1 (NC_014215.1)	17.83	540373-540311	-1	pyruvate oxidase (PFREUD_RS02185)	44
	16.05	876756-876694	-1	methylmalonyl-CoA mutase small subunit (PFREUD_RS03765)	224
	15.39	1644669-1644617	-1	transcriptional regulator	20

Organism (Genome ID)	Bit score	ANTAR-target RNA co-ordinates	Strand	Annotation of the gene linked to ANTAR-target RNAs (Locus Tag)	Distance (nt) of RNA from gene linked to ANTAR-target RNA
				(PFREUD_RS07175)	
	15.07	2579483-2579533	1	citrate synthase (PFREUD_RS11405)	5
Propionibacterium freudenreichii subsp. shermanii isolate PFREUDJS1 (NZ_LN997841.1)	17.83	2329779-2329841	1	pyruvate oxidase (PFREUDJS001_RS10290)	44
	16.05	755246-755184	-1	methylmalonyl-CoA mutase small subunit (PFREUDJS001_RS03180)	224
	15.07	2620242-2620292	1	citrate synthase (PFREUDJS001_RS11525)	45
Propionibacterium sp. oral taxon 193 strain F0672 (NZ_CP017040.1)	15	580710-580763	1	homoserine O-acetyltransferase (BCB70_RS02700)	477
Propionimicrobium sp. Marseille-P3275 strain Marseille-P3275T contig00001 (NZ_LT706985.1)	23.61	1778691-1778741	1	LacI family transcriptional regulator (CZ356_RS08345)	23
	19.78	764873-764823	-1	glycosyltransferase family 2 protein (CZ356_RS03665)	76
	18.17	581857-581803	-1	DNA polymerase III subunit epsilon (CZ356_RS02670)	255
	16.68	1275582-1275638	1	MFS transporter (CZ356_RS06130)	4
Pseudarthrobacter chlorophenolicus A6 plasmid pACHL01 (NC_011879.1)	17.72	279963-279898	-1	hypothetical protein (ACHL_RS21730)	16
Pseudarthrobacter chlorophenolicus A6 (NC_011886.1)	25	676358-676423	1	methylmalonate-semialdehyde dehydrogenase (CoA acylating) (ACHL_RS03150)	2
	21.97	95577-95640	1	tripartite tricarboxylate transporter TctB family protein (ACHL_RS00470)	23
	17.17	404622-404675	1	alpha-N-arabinofuranosidase (ACHL_RS01895)	14
	15.61	357905-357964	1	dihydroxyacetone kinase subunit DhaK (ACHL_RS01655)	73

Organism (Genome ID)	Bit score	ANTAR-target RNA co-ordinates	Strand	Annotation of the gene linked to ANTAR-target RNAs (Locus Tag)	Distance (nt) of RNA from gene linked to ANTAR-target RNA
	15.34	2039538-2039603	1	30S ribosomal protein S1 (ACHL_RS09090)	94
Pseudarthrobacter phenanthrenivorans Sphe3 plasmid pASPHE302 (NC_015147.1)	18.43	75616-75561	-1	ferredoxin--NAD(+) reductase (ASPHE3_RS21045)	183
Pseudarthrobacter phenanthrenivorans Sphe3 (NC_015145.1)	18.35	3706123-3706068	-1	sugar ABC transporter substrate-binding protein (ASPHE3_RS17190)	3
	17.88	2273699-2273754	1	LacI family DNA-binding transcriptional regulator (ASPHE3_RS10460)	17
	17.13	618889-618838	-1	2-hydroxyacid dehydrogenase (ASPHE3_RS03000)	485
	17.04	3203787-3203847	1	two-component sensor histidine kinase (ASPHE3_RS21255)	17
	15.27	1917982-1918047	1	30S ribosomal protein S1 (ASPHE3_RS08800)	94
Pseudarthrobacter sulfonivorans strain Ar51 (NZ_CP013747.1)	24.63	2250041-2249980	-1	sugar ABC transporter ATP-binding protein (AU252_RS10090)	5
	18.68	1830214-1830155	-1	hypothetical protein (AU252_RS08050)	0
	16.76	4278609-4278677	1	ribonuclease HII (AU252_RS19425)	74
	16.6	3355696-3355746	1	ABC transporter substrate-binding protein (AU252_RS15200)	35
	16.06	2996339-2996282	-1	copper oxidase (AU252_RS13495)	45
	15.65	267906-267857	-1	aldehyde dehydrogenase (AU252_RS01160)	7
	15.65	262426-262377	-1	aldehyde dehydrogenase (AU252_RS01140)	7
	15.62	3777164-3777116	-1	type I pantothenate kinase (AU252_RS17175)	33

Organism (Genome ID)	Bit score	ANTAR-target RNA co-ordinates	Strand	Annotation of the gene linked to ANTAR-target RNAs (Locus Tag)	Distance (nt) of RNA from gene linked to ANTAR-target RNA
	15.27	4860429-4860364	-1	30S ribosomal protein S1 (AU252_RS22125)	94
Pseudonocardia sp. EC080610-09 plasmid pBCI2-1 (NZ_CP012182.1)	19.87	279077-279030	-1	VOC family protein (AD006_RS29515)	68
Pseudonocardia sp. EC080619-01 plasmid pBCI1-2 (NZ_CP012185.1)	19.87	769514-769561	1	VOC family protein (AD017_RS31735)	68
Pseudopropionibacterium propionicum F0230a (NC_018142.1)	16.43	301894-301959	1	hypothetical protein (HMPREF9154_RS01395)	12
Renibacterium salmoninarum ATCC 33209 (NC_010168.1)	18.03	1074795-1074848	1	hypothetical protein (RSAL33209_RS05485)	10
	16.29	1070998-1070939	-1	hypothetical protein (RSAL33209_RS05450)	64
	16.23	1005759-1005815	1	membrane protein (RSAL33209_RS05135)	92
	15.18	1611245-1611182	-1	methylmalonate-semialdehyde dehydrogenase (CoA acylating) (RSAL33209_RS08115)	17
	15.14	2957917-2957855	-1	Nif3-like dinuclear metal center hexameric protein (RSAL33209_RS14620)	231
Rhodococcus aetherivorans strain IcdP1 (NZ_CP011341.1)	16.81	2510931-2510983	1	malate:quinone oxidoreductase (AAT18_RS11550)	36
Rhodococcus erythropolis CCM2595 (NC_022115.1)	18.48	137578-137631	1	TetR family transcriptional regulator (O5Y_RS00625)	7
	16.01	3652209-3652256	1	cytosol aminopeptidase (O5Y_RS16480)	255
Rhodococcus erythropolis PR4 DNA (NC_012490.1)	16.01	3959281-3959328	1	cytosol aminopeptidase (RER_RS20095)	255
	15.3	2862098-2862037	-1	D-Ala-D-Ala carboxypeptidase (RER_RS15290)	34
Rhodococcus erythropolis R138	16.01	3214398-3214351	-1	cytosol aminopeptidase (H351_RS15050)	255

Organism (Genome ID)	Bit score	ANTAR-target RNA co-ordinates	Strand	Annotation of the gene linked to ANTAR-target RNAs (Locus Tag)	Distance (nt) of RNA from gene linked to ANTAR-target RNA
(NZ_CP007255.1)					
Rhodococcus erythropolis strain BG43 (NZ_CP011295.1)	18.48	137863-137916	1	TetR family transcriptional regulator (XU06_RS00630)	7
	16.01	3690230-3690277	1	cytosol aminopeptidase (XU06_RS16800)	255
Rhodococcus fascians D188 (NZ_CP015235.1)	16.47	133189-133127	-1	gamma-aminobutyraldehyde dehydrogenase (A3L23_RS00625)	418
Rhodococcus jostii RHA1 (NC_008268.1)	16.62	7784307-7784251	-1	NADH dehydrogenase (RHA1_RS35505)	363
	16.19	2609128-2609176	1	alanine racemase (RHA1_RS12030)	50
Rhodococcus opacus B4 DNA (NC_012522.1)	15.99	4334716-4334769	1	hypothetical protein (ROP_RS19920)	49
	15.69	7609996-7610064	1	hypothetical protein (ROP_RS40775)	144
Rhodococcus sp. B7740 (NZ_CP010797.1)	18.19	4970838-4970892	1	MFS transporter (NY08_RS23335)	23
	16.04	3887305-3887364	1	DNA-binding protein (NY08_RS18010)	96
Rhodococcus sp. PBTS2 (NZ_CP015220.1)	16.47	3411253-3411315	1	gamma-aminobutyraldehyde dehydrogenase (A3Q41_RS16235)	418
Rhodoluna lacicola strain MWH-Ta8 (NZ_CP007490.1)	15.68	1202741-1202801	1	branched-chain amino acid ABC transporter permease (RHOLA_RS05925)	17
	15.24	330217-330150	-1	acetate--CoA ligase (RHOLA_RS01590)	14
Rothia dentocariosa ATCC 17931 (NC_014643.1)	17.47	1473946-1474000	1	bifunctional methylenetetrahydrofolate dehydrogenase/methenyltetrahydrofolate cyclohydrolase (HMPREF0733_RS06405)	39
Rothia mucilaginosa DNA, strain: NUM-Rm6536 (NZ_AP014938.1)	15.97	437430-437480	1	RNA-binding S4 domain-containing protein (RM6536_RS01640)	88
Rothia mucilaginosa DY-18 DNA (NC_013715.1)	18.33	83293-83238	-1	ornithine cyclodeaminase (RMDY18_RS00360)	5
	15.45	1021609-1021661	1	hypothetical protein (RMDY18_RS04100)	42
Rothia mucilaginosa strain	15.97	761512-761562	1	RNA-binding S4 domain-containing	88

Organism (Genome ID)	Bit score	ANTAR-target RNA co-ordinates	Strand	Annotation of the gene linked to ANTAR-target RNAs (Locus Tag)	Distance (nt) of RNA from gene linked to ANTAR-target RNA
FDAARGOS_369 (NZ_CP023510.1)				protein (CO690_RS03200)	
Saccharomonospora viridis DSM 43017 (NC_013159.1)	17.42	3078960-3079028	1	XRE family transcriptional regulator (SVIR_RS13855)	12
Saccharothrix espanaensis DSM 44229 complete genome (NC_019673.1)	19.42	5436887-5436821	-1	hypothetical protein (BN6_RS23530)	2
	16.17	3146208-3146270	1	hypothetical protein (BN6_RS42935)	21
Sanguibacter keddieii DSM 10542 (NC_013521.1)	19.26	337201-337151	-1	ABC transporter substrate-binding protein (SKED_RS01520)	10
Scardovia inopinata JCM 12537 DNA (NZ_AP012334.1)	18.1	1529587-1529653	1	putative holin-like toxin (SCIP_RS07835)	20
	15.07	1500669-1500618	-1	hypothetical protein (SCIP_RS06140)	36
Sinomonas atrocyanea strain KCTC 3377 (NZ_CP014518.1)	16.5	1024306-1024253	-1	hypothetical protein (SA2016_RS04845)	33
	15.66	1617992-1617930	-1	nitrite reductase (SA2016_RS07460)	68
Slackia heliotrinireducens DSM 20476 (NC_013165.1)	23.09	1700501-1700444	-1	3'-phosphoadenosine 5'-phosphosulfate sulfotransferase (SHEL_RS07250)	413
	21.59	1987700-1987750	1	3-methyl-2-oxobutanoate dehydrogenase subunit VorB (SHEL_RS08610)	5
	20.42	1226733-1226789	1	recombinase RecA (SHEL_RS05250)	82
Stackebrandtia nassauensis DSM 44728 (NC_013947.1)	20.8	4871519-4871574	1	NAD-dependent malic enzyme (SNAS_RS22550)	29
	18.92	1917023-1917082	1	hypothetical protein (SNAS_RS09010)	30
Streptomyces albus J1074 (NC_020990.1)	15.09	2012095-2012045	-1	ABC transporter permease (XNR_RS08435)	171
Streptomyces albus strain SM254 (NZ_CP014485.1)	15.09	2289125-2289075	-1	ABC transporter permease (Salbus254_RS09635)	171
Streptomyces avermitilis MA-4680 = NBRC 14893 DNA (NC_003155.5)	15.7	2358373-2358320	-1	membrane protein (SAVERM_RS10160)	398
	15.36	2967279-2967226	-1	mannosyltransferase (SAVERM_RS12700)	159

Organism (Genome ID)	Bit score	ANTAR-target RNA co-ordinates	Strand	Annotation of the gene linked to ANTAR-target RNAs (Locus Tag)	Distance (nt) of RNA from gene linked to ANTAR-target RNA
Streptomyces bingchenggensis BCW-1 (NC_016582.1)	22.79	1325634-1325572	-1	molecular chaperone DnaK (SBI_RS04410)	32
Streptomyces cyaneogriseus subsp. noncyanogenus strain NMWT 1 (NZ_CP010849.1)	16.78	2043973-2043899	-1	transcriptional regulator (TU94_RS08155)	51
Streptomyces fulvissimus DSM 40593 (NC_021177.1)	15.64	2195512-2195450	-1	hypothetical protein (SFUL_RS09650)	82
Streptomyces glaucescens strain GLA.O (NZ_CP009438.1)	15.45	2684874-2684812	-1	hypothetical protein (SGLAU_RS11560)	55
Streptomyces globisporus C-1027 (NZ_CP013738.1)	15.24	2202150-2202088	-1	hypothetical protein (WQO_RS09685)	82
Streptomyces griseus subsp. griseus NBRC 13350 DNA (NC_010572.1)	15.24	6048357-6048419	1	hypothetical protein (SGR_RS25590)	82
Streptomyces lividans TK24 (NZ_CP009124.1)	15.45	7213814-7213751	-1	LacI family transcriptional regulator (SLIV_RS32445)	91
Streptomyces lydicus A02 (NZ_CP007699.1)	21.31	6061131-6061192	1	LacI family transcriptional regulator (T261_RS27005)	9
	18.01	2624882-2624829	-1	hypothetical protein (T261_RS11520)	302
	15.49	9065496-9065546	1	type III effector protein (T261_RS39710)	274
Streptomyces parvulus strain 2297 (NZ_CP015866.1)	16.28	4484722-4484667	-1	decaprenylphosphoryl-beta-D-ribose oxidase (Spa2297_RS20440)	18
Streptomyces pratensis ATCC 33331 (NC_016114.1)	17.7	4725819-4725761	-1	hypothetical protein (SFLA_RS20365)	27
Streptomyces pristinaespiralis strain HCCB 10218 (NZ_CP011340.1)	18.83	8476818-8476874	1	exonuclease SbcC (SPRI_RS36235)	97
	18.83	55775-55719	-1	exonuclease sbcCD subunit D (SPRI_RS00190)	97
	17.8	7513420-7513468	1	sugar ABC transporter (SPRI_RS32325)	72

Organism (Genome ID)	Bit score	ANTAR-target RNA co-ordinates	Strand	Annotation of the gene linked to ANTAR-target RNAs (Locus Tag)	Distance (nt) of RNA from gene linked to ANTAR-target RNA
	15.35	5557595-5557641	1	agmatinase (SPRI_RS23705)	11
Streptomyces scabiei 87.22 complete genome (NC_013929.1)	16.26	9274780-9274829	1	L-asparagine permease (SCAB_RS39965)	7
Streptomyces sp. 4F (NZ_CP013142.1)	17.52	3516700-3516640	-1	class II fructose-bisphosphate aldolase (ASR50_RS15530)	32
Streptomyces sp. CFMR 7 strain CFMR-7 (NZ_CP011522.1)	15.24	6226773-6226835	1	hypothetical protein (ABE83_RS26035)	82
Streptomyces sp. CNQ-509 (NZ_CP011492.1)	18.89	3795481-3795431	-1	50S ribosomal protein L9 (AA958_RS16065)	1
Streptomyces sp. PAMC26508 (NC_021055.1)	19.99	2739778-2739819	1	hypothetical protein (F750_RS12050)	35
Streptomyces sp. S10(2016) (NZ_CP015098.1)	16.09	5995834-5995780	-1	MarR family transcriptional regulator (A4E84_RS26725)	155
Streptomyces sp. SAT1 (NZ_CP015849.1)	15.79	5047550-5047500	-1	hypothetical protein (A8713_RS21920)	38
Streptomyces sp. SirexAA-E (NC_015953.1)	16.1	1565172-1565240	1	TetR family transcriptional regulator (SACTE_RS06915)	25
	15.43	1508400-1508345	-1	transcriptional regulator (SACTE_RS06635)	32
Streptomyces venezuelae strain ATCC 15439 (NZ_CP013129.1)	18.23	6497952-6497896	-1	hypothetical protein (AQF52_RS29165)	30
	16.07	3448862-3448808	-1	hypothetical protein (AQF52_RS15265)	24
Streptomyces vietnamensis strain GIM4.0001 (NZ_CP010407.1)	18.68	142442-142501	1	hypothetical protein (SVTN_RS00745)	49
	16.41	2463738-2463683	-1	pullulanase-type alpha-1,6-glucosidase (SVTN_RS10835)	143
	15.51	7881746-7881797	1	succinyl-CoA--3-ketoacid-CoA transferase (SVTN_RS35175)	24
	15.38	3227892-3227837	-1	hypothetical protein (SVTN_RS14300)	24

Organism (Genome ID)	Bit score	ANTAR-target RNA co-ordinates	Strand	Annotation of the gene linked to ANTAR-target RNAs (Locus Tag)	Distance (nt) of RNA from gene linked to ANTAR-target RNA
Tessaracoccus sp. NSG39 (NZ_CP019606.1)	17.06	2506818-2506764	-1	DUF3263 domain-containing protein (BW730_RS11675)	9
	15.8	3143239-3143292	1	MgtC/SapB family protein (BW730_RS14350)	14
Tessaracoccus sp. T2.5-30 (NZ_CP019229.1)	16.52	146536-146474	-1	CsbD family protein (BKM78_RS00755)	10
	16	1577319-1577267	-1	aquaporin family protein (BKM78_RS07235)	7
Trueperella pyogenes strain 2012CQ-ZSH (NZ_CP012649.1)	22.62	324777-324715	-1	dihydroxyacetone kinase (AN946_RS01315)	17
	21.51	1421966-1422025	1	BMP family ABC transporter substrate-binding protein (AN946_RS06170)	5
	19.79	305936-305991	1	GntP family permease (AN946_RS01245)	16
	19.48	1400555-1400498	-1	hypothetical protein (AN946_RS06105)	29
	17.11	1076589-1076642	1	ABC transporter ATP-binding protein (AN946_RS04625)	46
	16.74	1566665-1566719	1	L-fucose isomerase (AN946_RS06865)	6
	16.33	2151628-2151567	-1	sugar (glycoside-pentoside-Hexuronide) transporter (AN946_RS09515)	5
	16.18	883231-883287	1	FMN-binding negative transcriptional regulator (AN946_RS03735)	217
	15.69	1750466-1750525	1	30S ribosomal protein S1 (AN946_RS07755)	91
	15.68	277245-277195	-1	hypothetical protein (AN946_RS01135)	326
Trueperella pyogenes strain TP6375 (NZ_CP007519.1)	21.51	848407-848348	-1	ABC transporter ATP-binding protein (CQ11_RS04015)	5
	19.79	1995302-1995247	-1	GntP family permease (CQ11_RS09140)	16
	19.48	869818-869875	1	hypothetical protein (CQ11_RS10670)	29

Organism (Genome ID)	Bit score	ANTAR-target RNA co-ordinates	Strand	Annotation of the gene linked to ANTAR-target RNAs (Locus Tag)	Distance (nt) of RNA from gene linked to ANTAR-target RNA
	17.78	1401608-1401552	-1	FMN-binding negative transcriptional regulator (CQ11_RS06540)	218
	17.75	1203837-1203784	-1	ABC transporter ATP-binding protein (CQ11_RS05610)	46
	16.92	1802328-1802264	-1	hypothetical protein (CQ11_RS08330)	83
	16.74	659505-659451	-1	L-fucose isomerase (CQ11_RS03075)	6
	16.33	469089-469028	-1	hypothetical protein (CQ11_RS02190)	5
	15.69	65751-65810	1	30S ribosomal protein S1 (CQ11_RS00345)	91
	15.68	2022040-2022090	1	MgtC/SapB family protein (CQ11_RS09240)	325
Trueperella pyogenes TP8 (NZ_CP007003.1)	21.51	794541-794600	1	BMP family ABC transporter substrate-binding protein (X956_RS03620)	5
	20.13	1952893-1952949	1	sugar ABC transporter substrate-binding protein (X956_RS08895)	29
	19.79	1911013-1911068	1	GntP family permease (X956_RS08740)	16
	19.48	773131-773074	-1	TetR/AcrR family transcriptional regulator (X956_RS10475)	29
	17.11	426192-426245	1	ABC transporter ATP-binding protein (X956_RS01940)	46
	16.74	956842-956896	1	L-fucose isomerase (X956_RS04470)	6
	16.33	1534199-1534138	-1	sugar (glycoside-pentoside-Hexuronide) transporter (X956_RS07145)	5
	16.18	232466-232522	1	FMN-binding negative transcriptional regulator (X956_RS01025)	217
	15.69	1174479-1174420	-1	30S ribosomal protein S1 (X956_RS05495)	91

Organism (Genome ID)	Bit score	ANTAR-target RNA co-ordinates	Strand	Annotation of the gene linked to ANTAR-target RNAs (Locus Tag)	Distance (nt) of RNA from gene linked to ANTAR-target RNA
	15.68	82570-82620	1	hypothetical protein (X956_RS00375)	325
	15.68	1882450-1882400	-1	hypothetical protein (X956_RS08635)	325
Verrucosispora maris AB-18-032 (NC_015434.1)	15.07	4457207-4457151	-1	hypothetical protein (VAB18032_RS19750)	14
Xylanimonas cellulosilytica DSM 15894 (NC_013530.1)	19.67	474824-474871	1	sugar ABC transporter substrate-binding protein (XCEL_RS02040)	17

Table S3: Alignment of actinobacterial ANTAR-target RNAs predicted using Infernal.
Structure based alignment of ANTAR-target RNAs obtained using Infernal are shown sorted according to the bit scores. Alignment is edited using RALEE v0.8 (PMID: 15377506). The stems are shaded in pink and blue. Secondary structure of RNAs is represented by WUSS notation.

Accession/Start-End	Sequence
NC_022567/1325289-1325349	AAAAAAUCUCUUGCAAUGAU.GCAACGGuc...............ggGUUAUCAUAGCA..AAGCUGCUUGAGCGGAAG
NC_015564/4535773-4535832	AAUGAACUAUCUACAACGAU.GUAGAAUu................ggUGAACUCAUGCA..AAGGAGCAUGCGAUGCCA
NZ_CP014761/2225592-2225649	AUCAUGGUCUCUACAACGUU.GUAGUAC..................aACGUUGUAGACA..AGGGUGUCACACACCGCG
NC_008726/6033904-6033960	AGAGAGAUCACCACAAAGGU.GUGGUUG...................ACUACACGGGCA..AAGUGGCCGGUUUUCGGG
NZ_CP007443/1784660-1784721	AAAACGGCGACCGCAAAGCU.GCGGUUGcc..............guaACAGAUACGGCA..AUGGGGCCGGAAAACCAG
NC_014218/792299-792362	UGCGUGAUAUGCACGAUGAC.GUGCUUUuau............acgcAUCACUAUGUCA..AUGGAGACACUAGAAAAG
NZ_CP011491/5794851-5794907	AAAGAGAUCGCCACAAAGGU.GUGGUUG...................ACUACACGGGCA..CAGUGGCCGUUCGUGGGA
NZ_CP016773/11044-11115	AACAAAUUCUUAACAAGGCU.GUUGUCCacggcaa....agucaucuUGUCUUCAGGCA..AGGAAGCCGAUUACUACG
NZ_CP012390/604046-604102	AGGUUCUCGACCGCAUUGGU.GUGAUCG...................UUGGACAGGGCA..ACGAUGCCAUGAACGACA
NZ_CP010437/2041213-2041274	AAAACGGCGACCGCAAAGCU.GCGGUCGcc..............guaACCGAUACGGCA..AUGGGGCCGGAAAACCAG
NZ_CP007155/7090364-7090418	AAAAUGCAUUCGUGAACGAA.CACGAA-...................-AAAUGAUCGCA..AUGUAGCGGUUUUUGACA
NC_011886/676358-676423	AUAUUCGCAACCAGAAUGAC.CUGGGUCacga..........aagccGGAUUACAUGCG..AAGGGGCAUCCAGCCAUG
NC_021054/2187067-2187117	ACAAAUUCCGAUGCAAAGAA.GCACU--...................----UGUGUACA..ACGAAGUAUCUUGGUAGU
NC_015758/2187796-2187846	ACAAAUUCCCAUGCAAAGAA.GCACU--...................----UGUGUACA..ACGAAGUAUCUUGGUAGU
NC_008769/2189421-2189471	ACAAAUUCCCAUGCAAAGAA.GCACU--...................----UGUGUACA..ACGAAGUAUCUUGGUAGU
NZ_CP003494/1889021-1889071	ACAAAUUCCCAUGCAAAGAA.GCACU--...................----UGUGUACA..ACGAAGUAUCUUGGUAGU
NC_020245/2159066-2159116	ACAAAUUCCCAUGCAAAGAA.GCACU--...................----UGUGUACA..ACGAAGUAUCUUGGUAGU
NC_016804/2168786-2168836	ACAAAUUCCCAUGCAAAGAA.GCACU--...................----UGUGUACA..ACGAAGUAUCUUGGUAGU
NZ_AM412059/2171338-2171388	ACAAAUUCCCAUGCAAAGAA.GCACU--...................----UGUGUACA..ACGAAGUAUCUUGGUAGU
NC_012207/2171412-2171462	ACAAAUUCCCAUGCAAAGAA.GCACU--...................----UGUGUACA..ACGAAGUAUCUUGGUAGU
NZ_CP014566/2171410-2171460	ACAAAUUCCCAUGCAAAGAA.GCACU--...................----UGUGUACA..ACGAAGUAUCUUGGUAGU
NZ_CP008744/2169564-2169614	ACAAAUUCCCAUGCAAAGAA.GCACU--...................----UGUGUACA..ACGAAGUAUCUUGGUAGU
NZ_CP009243/2171415-2171465	ACAAAUUCCCAUGCAAAGAA.GCACU--...................----UGUGUACA..ACGAAGUAUCUUGGUAGU
NZ_CP012095/2183860-2183910	ACAAAUUCCCAUGCAAAGAA.GCACU--...................----UGUGUACA..ACGAAGUAUCUUGGUAGU
NZ_CP009449/2147621-2147671	ACAAAUUCCCAUGCAAAGAA.GCACU--...................----UGUGUACA..ACGAAGUAUCUUGGUAGU
NZ_CP013741/2171711-2171761	ACAAAUUCCCAUGCAAAGAA.GCACU--...................----UGUGUACA..ACGAAGUAUCUUGGUAGU
NZ_HG813240/2173566-2173616	ACAAAUUCCCAUGCAAAGAA.GCACU--...................----UGUGUACA..ACGAAGUAUCUUGGUAGU
NC_020089/2191699-2191749	ACAAAUUCCCAUGCAAAGAA.GCACU--...................----UGUGUACA..ACGAAGUAUCUUGGUAGU
NZ_CP002883/2173137-2173187	ACAAAUUCCCAUGCAAAGAA.GCACU--...................----UGUGUACA..ACGAAGUAUCUUGGUAGU
NZ_CP002882/2166350-2166400	ACAAAUUCCCAUGCAAAGAA.GCACU--...................----UGUGUACA..ACGAAGUAUCUUGGUAGU
NC_017522/2168763-2168813	ACAAAUUCCCAUGCAAAGAA.GCACU--...................----UGUGUACA..ACGAAGUAUCUUGGUAGU
NZ_CP002885/2173333-2173383	ACAAAUUCCCAUGCAAAGAA.GCACU--...................----UGUGUACA..ACGAAGUAUCUUGGUAGU
NC_017524/2180824-2180874	ACAAAUUCCCAUGCAAAGAA.GCACU--...................----UGUGUACA..ACGAAGUAUCUUGGUAGU
NC_021740/2181844-2181894	ACAAAUUCCCAUGCAAAGAA.GCACU--...................----UGUGUACA..ACGAAGUAUCUUGGUAGU
NC_021194/2181480-2181530	ACAAAUUCCCAUGCAAAGAA.GCACU--...................----UGUGUACA..ACGAAGUAUCUUGGUAGU
NC_009565/2195516-2195566	ACAAAUUCCCAUGCAAAGAA.GCACU--...................----UGUGUACA..ACGAAGUAUCUUGGUAGU
NC_009525/2197160-2197210	ACAAAUUCCCAUGCAAAGAA.GCACU--...................----UGUGUACA..ACGAAGUAUCUUGGUAGU
NZ_CP009480/2182565-2182615	ACAAAUUCCCAUGCAAAGAA.GCACU--...................----UGUGUACA..ACGAAGUAUCUUGGUAGU
NC_018143/2187268-2187318	ACAAAUUCCCAUGCAAAGAA.GCACU--...................----UGUGUACA..ACGAAGUAUCUUGGUAGU
NC_000962/2187261-2187311	ACAAAUUCCCAUGCAAAGAA.GCACU--...................----UGUGUACA..ACGAAGUAUCUUGGUAGU
NZ_CP007027/2186649-2186699	ACAAAUUCCCAUGCAAAGAA.GCACU--...................----UGUGUACA..ACGAAGUAUCUUGGUAGU
NZ_CP002871/2172407-2172457	ACAAAUUCCCAUGCAAAGAA.GCACU--...................----UGUGUACA..ACGAAGUAUCUUGGUAGU
NC_012943/2235762-2235812	ACAAAUUCCCAUGCAAAGAA.GCACU--...................----UGUGUACA..ACGAAGUAUCUUGGUAGU
NC_016768/2232493-2232543	ACAAAUUCCCAUGCAAAGAA.GCACU--...................----UGUGUACA..ACGAAGUAUCUUGGUAGU
NC_018078/2235643-2235693	ACAAAUUCCCAUGCAAAGAA.GCACU--...................----UGUGUACA..ACGAAGUAUCUUGGUAGU
NC_020559/2178334-2178384	ACAAAUUCCCAUGCAAAGAA.GCACU--...................----UGUGUACA..ACGAAGUAUCUUGGUAGU
NC_022350/2186892-2186942	ACAAAUUCCCAUGCAAAGAA.GCACU--...................----UGUGUACA..ACGAAGUAUCUUGGUAGU
NZ_AP014573/2192698-2192748	ACAAAUUCCCAUGCAAAGAA.GCACU--...................----UGUGUACA..ACGAAGUAUCUUGGUAGU
NZ_CP010339/2185332-2185382	ACAAAUUCCCAUGCAAAGAA.GCACU--...................----UGUGUACA..ACGAAGUAUCUUGGUAGU
NZ_CP010337/2190192-2190242	ACAAAUUCCCAUGCAAAGAA.GCACU--...................----UGUGUACA..ACGAAGUAUCUUGGUAGU
NZ_CP010340/2199709-2199759	ACAAAUUCCCAUGCAAAGAA.GCACU--...................----UGUGUACA..ACGAAGUAUCUUGGUAGU
NZ_CP010338/2182762-2182812	ACAAAUUCCCAUGCAAAGAA.GCACU--...................----UGUGUACA..ACGAAGUAUCUUGGUAGU
NZ_CP009426/2167819-2167869	ACAAAUUCCCAUGCAAAGAA.GCACU--...................----UGUGUACA..ACGAAGUAUCUUGGUAGU
NZ_CP009427/2195453-2195503	ACAAAUUCCCAUGCAAAGAA.GCACU--...................----UGUGUACA..ACGAAGUAUCUUGGUAGU
NZ_CP010330/2194218-2194268	ACAAAUUCCCAUGCAAAGAA.GCACU--...................----UGUGUACA..ACGAAGUAUCUUGGUAGU
NZ_CP007809/2166335-2166385	ACAAAUUCCCAUGCAAAGAA.GCACU--...................----UGUGUACA..ACGAAGUAUCUUGGUAGU
NZ_CP012506/2160992-2161042	ACAAAUUCCCAUGCAAAGAA.GCACU--...................----UGUGUACA..ACGAAGUAUCUUGGUAGU
NZ_CP009100/2187257-2187307	ACAAAUUCCCAUGCAAAGAA.GCACU--...................----UGUGUACA..ACGAAGUAUCUUGGUAGU
NZ_CP009101/2187289-2187339	ACAAAUUCCCAUGCAAAGAA.GCACU--...................----UGUGUACA..ACGAAGUAUCUUGGUAGU
NZ_CP012090/1954309-1954359	ACAAAUUCCCAUGCAAAGAA.GCACU--...................----UGUGUACA..ACGAAGUAUCUUGGUAGU
NZ_CP013747/2249980-2250041	CCAGAACCCCCGACAAUGAU.GUCGAUCcu..............cacAUUCUCAUCACG..AAGGAGUGACCAGUGACA
NZ_CP015773/1085783-1085833	ACAAAUUCCCAUGCAAAGAA.GCACU--...................----UGUGUACA..ACGAAGUAUUUUGGUAGU
NZ_CP012590/627231-627295	ACGGGGCGGAUGACAACGAU.GUCAUCGgaau...........agaaCUGUACCUGACA..ACGUUGUCUCCGGUCAAG
NC_008541/399351-399411	AUGAAGCCCACACCAAAGAU.GUUGUGGca...............aaAGAACAUAUACA..AAGGGGUAGAAGUUCAAU
NC_015738/2403432-2403484	UCAAUGAUCCCAACAGUGAA.GUUGC--...................--CAAAAAGGCA..GCGAAGCUUCAAACGGAA

```
NZ_CP016771/508869-508923     AACCUCUCAGCGGCAAAGAC.GCCGUA-...................-CUGACAGGUCA..CUGAUGACAAAACUAAAA
NZ_AP012327/2246903-2246953   ACGCUCAUAAUCGAAACGAU.UCGAA--...................----AGUAAGCG..AAGACGCUGAAAACGAAG
NZ_CP007287/1150528-1150582   GCAAGUAAGGGCUCAAAGUU.GAGCCA-...................-UGAUGAUUGCA..AUGUAGCAAAAUAACAAG
NZ_LT706985/1778691-1778741   ACCUAAAUUGAAACAAAGGA.GUUU---...................---GUCGUGGCA..AAGAAGCCUCCCACUCGA
NZ_CP008953/3466226-3466279   AAAGUAACAGAUACAACGAC.GUAUC--...................-CCUUAUCCAUG..ACGGUAUGCUCAGCGAAG
NZ_CP013200/2964252-2964307   AUUUAUAGCAUC-UAAUGGU.GCGAUAU...................AUGCACAAUUCA..AAGGAGAAUCAUGACAGA
NZ_CP006018/1129860-1129914   GCAAGUAAGGGCUCAAAGUU.GAGCCG-...................-UGAUGAUUGCA..AUGUAGCAAAAUAACAAG
NC_013165/1700444-1700501     GUACGUGAAAGAGCAACGGU.GCUUUUU..................gAAGGCAAGAGCA..AUGGUGGUUCUAUAAAAG
NC_015673/784296-784358       CUUGAUAACGCAGCAAAGAA.GCUGAGCaac.............gagUCCUUCUUGGCA..AAGGCGCCAGAGAAGGUG
NC_008541/3967947-3968005     UUCAUUCCGAAGCCAACGAC.GUCUUCUa.................cGGAGGUAGAACA..AUGAAGUUUCCUGCCAUG
NZ_CP016777/10943-11014       AACAAAUUCUUAACAAGGCU.GUUGUCCacggcaa....aguaauuuUGUCUUCAGGCA..AAGAGGCUGAUUACUACG
NC_017219/918010-918066       UAGCGAUUUCGUGCAAUGAU.GCACGAG...................CCGAUCCAAGCA..AAGGGGCUGACCAUGAGC
NC_011593/918219-918275       UAGCGAUUUCGUGCAAUGAU.GCACGAG...................CCGAUCCAAGCA..AAGGGGCUGACCAUGAGC
NC_016582/1325572-1325634     ACAAUAAAACUCACAAAGGU.GUUGUUUuau.............agcAUUGAGCAUGAA..UCGGAUCACUCUGUGAAG
NC_014218/625569-625621       GUUCAUUCAUUCUCAAUGAA.GAGAA--...................--AGAUACAACA..AAGGAGUUGUAGGCGCAU
NZ_CP014513/1628135-1628207   UGACGCCGAUUUACAACGAU.GUAAUAGucgaugga...gcacccacCCCUACGAAGCA..AUGGAGCUCCAUGUCGAC
NZ_CP012649/324715-324777     AAAAAGCCGUAGCCAAAGCU.GUUUAUGcga.............aagGAAACAAGAACA..AUGACGUUUUUGGUUAAC
NZ_CP024160/94937-94997       GCGAUGGCAGCGGCGAUGAU.GCCGUUGga...............gcCAAACAAUAGCA..AUGCUGCUAAGUACGUAA
NC_009338/1209116-1209171     UGAGAGAUCACCACAAAGGU.GUGGUU-...................GACUACAGGGCA..AUUGGGCCGGACUCGGGG
NC_014814/5213312-5213367     UGAGAGAUCACCACAAAGGU.GUGGUU-...................GACUACAGGGCA..AUUGGGCCGGACUCGGGG
NC_015671/2826867-2826924     CAUGGUUCCUCUACAACGAU.GUAGAAC..................gACGACGAUCGCG..AGGGAGCGAGUCGAUGGG
NC_015564/650876-650926       UACGUCUUAAGCGCAACGAC.GCGCU--...................----UACCGGCA..AUGCUGCCGGAGCCACGA
NZ_CP016282/3698598-3698660   AACGAUUCCAUGGAAACGAU.UCCAUAGgau.............ccaCGCACGUACACA..AGGGAGUGGCUGUGGAAG
NZ_AP017900/6584176-6584229   CUUCCACCAACGGCAACGGC.GCCGC--...................-UGGCAAUGGCA..ACGGAGCCAACGGGAAUG
NZ_CP017839/2299999-2300052   CUUCCACCAACGGCAACGGC.GCCGC--...................-UGGCAAUGGCA..ACGGAGCCAACGGGAAUG
NC_011886/95577-95640         AUUCUAUGAAAUGCAAUGAC.GCGCAGUgaa............aaggAAUCCCAUGACA..ACGGAGUCAGUGACGACG
NC_010545/2341820-2341876     ACGGUUCCCACGGCAAGGGC.GCCGAUA...................AGGGUGCAGGCA..ACGGUGCCGACAAGGGUG
NZ_LT906481/1654874-1654930   ACGGUUCCCACGGCAAGGGC.GCCGAUA...................AGGGUGCAGGCA..ACGGUGCCGACAAGGGUG
NZ_CP007443/2169079-2169133   GUCAUAUCAAUCUAAGCGGU.UAGAUU-...................-GAUAUGAAUCA..AAGGAGAUUUCAAUGAGG
NZ_AP012325/603559-603619     CUAACAGAAUUUGCAACGUU.GCAAAUCcc...............gaAACAGACGGGCG..ACGUUGCCAUAGAAGAAA
NZ_CP017812/1397258-1397314   ACAAAGCUUAUGGCAAAGGU.GCCAUUA...................UGCGUUUGGGUG..AAGAUACUCGUCCCCAAG
NC_013165/1987700-1987750     AUCUGAUGUCUGAGAAAGUC.CUCAUG-...................-----AAGGGCA..ACGAGGCCAUGGCUGAAG
NZ_CP012649/1421966-1422025   AAUGUGAUCACUGCAGAGGA.GCAGUGAa................gcAGACCACUUACG..AAGGAGUAACCUGUGAGG
NZ_CP007519/848348-848407     AAUGUGAUCACUGCAGAGGA.GCAGUGAa................gcAGACCACUUACG..AAGGAGUAACCUGUGAGG
NZ_CP007003/794541-794600     AAUGUGAUCACUGCAGAGGA.GCAGUGAa................gcAGACCACUUACG..AAGGAGUAACCUGUGAGG
NZ_CP006841/144531-144587     CCGGCCUCGUCGACGAAGUU.GUCGAAG...................CCUACGAAGGCA..AAGAGGCCGCCGACAAGG
NC_008618/1609471-1609525     GUAAUAUCAAUCUAAGCGGU.UAGAUU-...................-GAUAUGAAUCA..AAGGAGAUUUCAAUGAGG
NZ_CP007699/6061131-6061192   UACACGUUGGCCGCAACGAC.GCGGACGgc..............acaAGCAGAAUGGCA..ACGGCGCCAUGGCGAACA
NZ_AP014658/2161620-2161684   AUAACAAAUAUUGCGAUGGC.GCAACGAugau...........ccauCACAGUAUUGCA..AAGAAGCAAGGACCCAAG
NC_014656/1762029-1762093     AUAACAAAUAUUGCGAUGGC.GCAACGAugau...........ccauCACAGUAUUGCA..AAGAAGCAAGGACCCAAG
NC_015067/2254067-2254131     AUAACAAAUAUUGCGAUGGC.GCAACGAugau...........ccauCACAGUAUUGCA..AAGAAGCAAGGACCCAAG
NZ_CP016019/2295537-2295601   AUAACAAAUAUUGCGAUGGC.GCAACGAugau...........ccauCACAGUAUUGCA..AAGAAGCAAGGACCCAAG
NZ_CP011965/1681266-1681330   AUAACAAAUAUUGCGAUGGC.GCAACGAugau...........ccauCACAGUAUUGCA..AAGAAGCAAGGACCCAAG
NZ_CP008802/1251722-1251774   ACGUUUCCAUAAACAAAGUA.GUUU---...................-GACACCCUGCA..GCGAGGCAGAUACAUAUG
NZ_CP006713/1022299-1022374   AUUGCAGCAGUAACAAAGAU.GUUGUGUauugcauuccuuuaaggagAGUGAACGAGCA..UGGGCGCUGCAGAAAAAA
NC_020517/419361-419436       AUUGCAGCAGUAACAAAGAU.GUUGUGUauugcauuccuuuaaggagAGUGAACGAGCA..UGGGCGCUGCAGAAAAAA
NZ_CP022436/2248139-2248195   CCACUGAUCAACACAACGUC.GUGUAAA...................ACCCCGGUGGCG..AAGUAGCCGCCGACCAGG
NC_021064/2362500-2362551     UAAUCCAUUUUUACAACGAU.GUAA---...................-CGGAACAUGCA..A-GGNGCACGCCGAGACG
NC_014246/26799-26853         UGGAUGCAUAUCCCAACGAU.GUGAUA-...................-CCGAACUGUCA..AAGGAGACUGUGAUGUCC
NZ_CP006715/665275-665329     GCAGGGAUUCAUACAGUGAU.GUAUGA-...................-ACCAUUUGGCA..AUGAUGCUGAGAGAAAGG
NC_013947/4871519-4871574     UUGAAUUGAGUCGCAAAGGA.GCGAAG-...................AUGUCUGUGGCC..AAGUUGCCCAGUGCCGGU
NZ_CP006850/7697910-7697964   GAUCAGUAACACACAAAGGA.GUGUUC-...................-UGAUGCAGGCA..UCGUUGCCCAGCAUGGUG
NZ_CP017150/3576710-3576760   AAAAAGACGGUGACAAUGAC.GUUGA--...................----CGGUGACA..AGGGUGUCAGCACAGUGG
NZ_CP006842/641767-641835     AUCAACAGCCCGGCAAUGAU.GCCGACAgcguag.......gacaccAGCAAGGAGUCG..ACGAAGACCUCACUGAAU
NC_009342/714064-714117       CCGUCGAUAUCGACAAAGUU.GUCGC--...................-GCUUGUUUGCA..CCGGAGCACAUGGCGAGU
NC_021351/701063-701116       CCGUCGAUAUCGACAAAGUU.GUCGC--...................-GCUUGUUUGCA..CCGGAGCACAUGGCGAGU
NC_021352/701064-701117       CCGUCGAUAUCGACAAAGUU.GUCGC--...................-GCUUGUUUGCA..CCGGAGCACAUGGCGAGU
NZ_CP007724/625963-626016     CCGUCGAUAUCGACAAAGUU.GUCGC--...................-GCUUGUUUGCA..CCGGAGCACAUGGCGAGU
NZ_CP007722/656779-656832     CCGUCGAUAUCGACAAAGUU.GUCGC--...................-GCUUGUUUGCA..CCGGAGCACAUGGCGAGU
NZ_CP012117/21017-21071       AAGUAGUCCUAAGCAAUGGC.GCUCUC-...................-UGCCUAAGUCA..GCGAUGACAGCGACGGGA
NZ_CP006711/1516055-1516111   CAGGGGCUUCGUGCAAUGAU.GCACGAG...................CCGAUCCAAGCA..AAGGGGCUGACCAUGAGC
NZ_CP006715/1592334-1592390   CAGGGGCUUCGUGCAAUGAU.GCACGAG...................CCGAUCCAAGCA..AAGGGGCUGACCAUGAGC
NC_017218/652499-652555       CAGGGGCUUCGUGCAAUGAU.GCACGAG...................CCGAUCCAAGCA..AAGGGGCUGACCAUGAGC
NZ_AP012324/1590628-1590684   CAGGGGCUUCGUGCAAUGAU.GCACGAG...................CCGAUCCAAGCA..AAGGGGCUGACCAUGAGC
NZ_CP006712/1542383-1542439   CAGGGGCUUCGUGCAAUGAU.GCACGAG...................CCGAUCCAAGCA..AAGGGGCUGACCAUGAGC
NZ_CP006713/1627645-1627701   CAGGGGCUUCGUGCAAUGAU.GCACGAG...................CCGAUCCAAGCA..AAGGGGCUGACCAUGAGC
NZ_CP006714/1543740-1543796   CAGGGGCUUCGUGCAAUGAU.GCACGAG...................CCGAUCCAAGCA..AAGGGGCUGACCAUGAGC
NZ_CP006716/1587761-1587817   CAGGGGCUUCGUGCAAUGAU.GCACGAG...................CCGAUCCAAGCA..AAGGGGCUGACCAUGAGC
NZ_CP010413/521165-521221     CAGGGGCUUCGUGCAAUGAU.GCACGAG...................CCGAUCCAAGCA..AAGGGGCUGACCAUGAGC
NZ_CP019596/1647336-1647392   CAGGGGCUUCGUGCAAUGAU.GCACGAG...................CCGAUCCAAGCA..AAGGGGCUGACCAUGAGC
NC_020517/1647321-1647377     CAGGGGCUUCGUGCAAUGAU.GCACGAG...................CCGAUCCAAGCA..AAGGGGCUGACCAUGAGC
NZ_CP008885/1607611-1607667   CAGGGGCUUCGUGCAAUGAU.GCACGAG...................CCGAUCCAAGCA..AAGGGGCUGACCAUGAGC
```

NC_014169/1608182-1608238	CAGGGGCUUCGUGCAAUGAU.GCACGAG...................CCGAUCCAAGCA..AAGGGGCUGACCAUGAGC
NZ_AP012332/713494-713559	AGAAUUAUUCUCACACAGGA.GUGACGAugac..........uacaaAAGAAAACGACA..AUGCCGUCGUUAAGAAUA
NC_014644/852601-852666	AGAAUUAUUCUCACACAGGA.GUGACGAugac..........uacaaAAGAAAACGACA..AUGCCGUCGUUAAGAAUA
NC_017456/1045532-1045597	AGAAUUAUUCUCACACAGGA.GUGACGAugac..........uacaaAAGAAAACGACA..AUGCCGUCGUUAAGAAUA
NZ_CP011269/6251522-6251576	GAGUUCUUAGUUACAAAGGU.GUAGUU-...................-CGAUUGCGGCG..ACGGUGCCGUAGGGAGUG
NZ_CP009914/6321128-6321182	GAGUUCUUAGUUACAAAGGU.GUAGUU-...................-CGAUUGCGGCG..ACGGUGCCGUAGGGAGUG
NZ_CP015732/54428-54483	UACUUGCGGAAUUCAACGGU.GAAUAU-...................UCAUAAGGAGCA..AGGGUGCUUUCUCAAGAC
NZ_AP012322/1863064-1863117	AUUUUCUGUGGCAAACAGCA.UUGUU--...................-UUAACGAUGCA..AUGGCGCAUUGGGCCAGA
NZ_AP012325/1858280-1858333	AUUUUCUGUGGCAAACAGCA.UUGUU--...................-UUAACGAUGCA..AUGGCGCAUUGGGCCAGA
NC_020133/3552989-3553040	GUUAUACCGACGGCAGAGUG.GUCGU--...................---GCCUUUGCA..AAGGCGCAGCCAACUAAG
NZ_HG917972/2705617-2705668	GUUAUACCGACGGCAGAGUG.GUCGU--...................---GCCUUUGCA..AAGGCGCAGCCAACUAAG
NC_010612/2842712-2842763	GUUAUACCGACGGCAGAGUG.GUCGU--...................---GCCUUUGCA..AAGGCGCAGCCAACUAAG
NZ_CP007456/334670-334721	UGCGAAAUCAGCACAAUGAA.GUGCA--...................---UAUCAUGCA..UAGGAGCAGUAUUUUCCG
NC_013165/1226733-1226789	AUAAGAAGUUCGGCAAAGGU.GCCAUCA...................UGAAGCUUGGCG..AAGAUGCCGAAAAGCUGG
NZ_CP023445/4013130-4013182	UCCUGCUGAUCCGCAAUGGC.GCGA---...................-AGGAGCUGGCA..AUGCCGCCCAGGCCGAGG
NZ_CP016775/10992-11063	AACAAAUCCGCAACAAAGCU.GUUGUCCacggcaa....agugauccUAUCUUCCGGUA..UCGAAGCUGAUUACUACG
NC_004369/2288328-2288390	AAGACAUGAUCCACAUCGCC.GUGGCUGcug.............acaGCACCGUCGGCG..AUGAUGCUUGCUGCGACG
NZ_CP017297/3019293-3019349	GGGAACACGUCGACAUCGAG.GUCGAUG...................GUGUUGUCCGCA..GUGGUGCGGGCGAUGAUU
NZ_CP017297/753990-754046	GGGAACACGUCGACAUCGAG.GUCGAUG...................GUGUUGUCCGCA..GUGGUGCGGGCGAUGAUU
NZ_CP017297/3849285-3849341	GGGAACACGUCGACAUCGAG.GUCGAUG...................GUGUUGUCCGCA..GUGGUGCGGGCGAUGAUU
NC_021251/2174956-2175006	ACAAAUUCCCAUGCAAAAAA.GCACU--...................----UGUGUACA..ACGAAGUAUCUUGGUAGU
NC_013729/2485796-2485859	AAAGUUGCUCUUGCAUCGAU.GCAAUUCgcu............agugAAGAUGCUUGCA..ACGAUGCAAACCGGCCAC
NC_014550/976738-976792	UUGAUCUGCUCCACAGCGGU.GUGGAU-...................-GGUCCCUUCCA..AAGUCGGAUGAGGUGUAU
NZ_CP007456/2161708-2161764	GUAUCCAUAAUCGAAACGAU.UCGAAAG...................UAAGUGAAGGCG..UUGUAGCCGAAGCGAAGA
NC_017219/47851-47907	GUAUCCAUAAUCGAAACGAU.UCGAAAG...................UAAGUGAAGGCG..UUGUAGCCGAAGCGAAGA
NC_011593/48060-48116	GUAUCCAUAAUCGAAACGAU.UCGAAAG...................UAAGUGAAGGCG..UUGUAGCCGAAGCGAAGA
NZ_AP012330/2073001-2073057	GUAUCCAUAAUCGAAACGAU.UCGAAAG...................UAAGUGAAGGCG..UUGUAGCCGAAGCGAAGA
NZ_CP007803/2159806-2159856	ACAAAUUCCCAUGUAAAGAA.GCACU--...................----UGUGUACA..ACGAAGUAUCUUGGUAGU
NZ_CP007003/1952893-1952949	CAAUAGUUCUUCACAAAGGA.GAGAAGG...................AUGAACAAGACA..AGGUUGUCGCGUUCCGCG
NC_013714/299223-299280	GACAAUUCAUUUACAACGAU.GUACUAU..................aGCGCAUAUUACA..ACGUUGUACAAAUUGACA
NZ_AP012326/299225-299282	GACAAUUCAUUUACAACGAU.GUACUAU..................aGCGCAUAUUACA..ACGUUGUACAAAUUGACA
NZ_AP012322/787294-787346	UUGAUCCAAGGCGCAAGGCC.GCUCUU-...................---GACACUGCA..UUGGCGCAGGUGGAGAAG
NZ_CP014241/1353009-1353061	UUGAUCCAAGGCGCAAGGCC.GCUCUU-...................---GACACUGCA..UUGGCGCAGGUGGAGAAG
NZ_CP017717/9630535-9630594	GAUGCUCUGGCGGCGGUGAC.GCCGACAc................guCAUUAAAAGGCA..AUGGAGCCUGGGAAAAUU
NC_021055/2739768-2739826	CCAUUCGUCGCAACAAUGAC.GUUGCUCg.................aAAUUCGAAUGCA..ACGUAGCGUUUCUCGUAU
NZ_CP011269/4166579-4166635	UAGGUGAACUCAGCAAAGCU.GCUGCUU...................GGGCGCCGCGCA..ACGUAGCGCUGACCGGUC
NZ_CP006841/2631106-2631157	GCCUUGGCAACGGCAACGCU.GCUGG--...................---CAUUCGGCA..UUGUCGCCUGUGGUGAUG
NZ_CP018135/1224475-1224527	CCAUGUUUGGCCUCAAUGCU.GGGGCU-...................---AAGCGCGCG..AAGAAGCGUGCCGCCAAA
NZ_CP012182/279030-279077	AUCGAAUUUGCCCCACGGGU.GGGG---...................------UAGGCA..AGGACGCCGCGGAGCAGA
NZ_CP012185/769514-769561	AUCGAAUUUGCCCCACGGGU.GGGG---...................------UAGGCA..AGGACGCCGCGGAGCAGA
NZ_CP013254/515543-515592	CUUACACUACGUGCAAUGGU.GCACA--...................-----CAGUGCA..AAGUUGCACGGCGUGCAA
NZ_CP020468/414732-414788	GCUGGAUGCGCCAGAACGAG.CUGGCUG...................ACAUCCAAGACA..ACGCAGUCCCUCUCGAAA
NZ_CP009251/301569-301620	UCGGUUAUCUCUUAAACGUU.UGAGU--...................---AAGAAAGCA..AAGCUGCUGUACAUAAAU
NZ_CP012649/305936-305991	UAGAAGCUCUAGGCAAAGAU.GCCUAA-...................GCAUCCAUCUCA..AAGUCGAGUGAACCCUCC
NZ_CP007519/1995247-1995302	UAGAAGCUCUAGGCAAAGAU.GCCUAA-...................GCAUCCAUCUCA..AAGUCGAGUGAACCCUCC
NZ_CP007003/1911013-1911068	UAGAAGCUCUAGGCAAAGAU.GCCUAA-...................GCAUCCAUCUCA..AAGUCGAGUGAACCCUCC
NZ_LT706985/764823-764873	AACUUCCUAAUGACAUAGAA.GUUAU--...................----CAUCGUCA..AUGAUGGCUCAACUGAUA
NC_014218/40752-40799	CUCUAUCCACCGGCAAUGAU.GCCG---...................------AUCUCA..UAGAGGAGUUAAAAGAAC
NC_013530/474820-474871	UCCGACACUUCC-CACUGGA.GAGA---...................-AGGACAACGCA..AUGAAGCGUCUUACAAGG
NZ_CP004350/282795-282854	GUCAAACGCUCCGCCCCGAA.GUGGUUGa................agCUAAUCUCCGCA..AUGGCGCGGCGGACCAAG
NC_010545/547036-547092	UACAUGUCCAGGGCGAUGAU.GCCUUCC...................UUGAGGCGGGCA..CCGGCGCCGUCGUAGAAA
NC_020230/536435-536491	UACAUGUCCAGGGCGAUGAU.GCCUUCC...................UUGAGGCGGGCA..CCGGCGCCGUCGUAGAAA
NZ_LT906481/2232424-2232480	UACAUGUCCAGGGCGAUGAU.GCCUUCC...................UUGAGGCGGGCA..CCGGCGCCGUCGUAGAAA
NC_013131/8698208-8698262	CGGCUAGAAUCGACAAUGGU.GUUGUC-...................-CUAAAUAGACA..AUGAGGUUGUCGAUCGUG
NZ_CP011269/3804230-3804294	UCGAGUUCAUCCCCAAUGCC.GGGGCGUgcuu...........ggcgACGACCAACGUG..AUGGAACGUCGUGGCAAG
NZ_CP012477/315774-315838	UCAUGCCAGUCAUCAAAGAC.GGUGACGgucg...........accgGAGGUUAUAUCA..AUGAAGAUCACCACCAAA
NZ_CP009244/2657682-2657735	AACUUGCAAACUACAACGGU.GUAAU--...................-UUUGCGGAGCA..AUUGCGCUGGUGUAGGGG
NZ_CP019705/2744264-2744317	AACUUGCAAACUACAACGGU.GUAAU--...................-UUUGCGGAGCA..AUUGCGCUGGUGUAGGGG
NZ_CP014634/2038187-2038240	UCGAUUCCCUCACCAAUGAC.GUUGA--...................-GGUUGCCAUCA..GUGAUGAUGGCGGCCACC
NZ_CP014635/2075643-2075696	UCGAUUCCCUCACCAAUGAC.GUUGA--...................-GGUUGCCAUCA..GUGAUGAUGGCGGCCACC
NZ_CP021252/737452-737505	UCGAUUCCCUCACCAAUGAC.GUUGA--...................-GGUUGCCAUCA..GUGAUGAUGGCGGCCACC
NZ_CP012649/1400498-1400555	UCUGUCAGCGUCGCGAAGGU.GCGAAUG..................uAUGAAAGGUGCA..AAGAUGCCCCAUUGAAA
NZ_CP007519/869818-869875	UCUGUCAGCGUCGCGAAGGU.GCGAAUG..................uAUGAAAGGUGCA..AAGAUGCCCCAUUGAAA
NZ_CP007003/773074-773131	UCUGUCAGCGUCGCGAAGGU.GCGAAUG..................uAUGAAAGGUGCA..AAGAUGCCCCAUUGAAA
NC_013203/420130-420185	UUCAUUUCUUACAAAAAGAC.UUGUAG-...................AAGUGAUCUGCU..AUGAAGCAUAAAAUUUCA
NC_019673/5436821-5436887	UGAUAUUUGAACGCAACGUA.GCGUUUCgacua.........ucagaAACAACGGAGCA..CAGGAGCUGAGCACGAUG
NC_013714/2559797-2559866	AAUGACUUACUUACCAUGGA.GUAGUUAggaauu......cugccccGACACAGUAGCA..AAGGAGCUCACGAACAUG
NZ_AP012326/2559099-2559168	AAUGACUUACUUACCAUGGA.GUAGUUAggaauu......cugccccGACACAGUAGCA..AAGGAGCUCACGAACAUG
NZ_CP007457/254651-254719	CCGAACAAUUGUACAACGUU.GUAUAACcugcua.......uauucaACGAUGUAAACA..UUGGCGUUUAUGAGAAAG
NZ_AP017457/676069-676125	ACUUACUUCAACAAAAUGGA.UUGUCCG...................CGGUUCCGGGCA..AAUUCGCCACUAGACUUU
NC_019395/2388576-2388629	GUCGCCUUACUUGCAUCGGC.GCAAC--...................-AUUUGGAAACA..AUGGAGUUGUGAAAAAUU

```
NZ_CP014352/962972-963025      GUCGCCUUACUUGCAUCGGC.GCAAC--...................-AUUUGGAAACA..AUGGAGUUGUGAAAAAUU
NZ_CP013126/2384398-2384451    GUCGCCUUACUUGCAUCGGC.GCAAC--...................-AUUUGGAAACA..AUGGAGUUGUGAAAAAUU

NZ_CP015970/1261613-1261666    GUCGCCUUACUUGCAUCGGC.GCAAC--...................-AUUUGGAAACA..AUGGAGUUGUGAAAAAUU
NZ_CP019400/905646-905699      GUCGCCUUACUUGCAUCGGC.GCAAC--...................-AUUUGGAAACA..AUGGAGUUGUGAAAAAUU
NZ_CP011853/1991447-1991503    UGAAGGCUCGUCGCAAUGAA.GCGAAAG...................CCGCUGCGGUCG..ACGUCGACGAGGGCGAUA
NC_013521/337144-337211        CAACGUUGUCUUACAACGUU.GUAAGUGggauu.........cccugaAGGACACCGCA..AUGAAGCGCAUCCCCUCC
NZ_CP011005/1447414-1447470    AGCGCCUGCACCACAAUGUC.GUGGCCA...................CGGUUUUGUUCA..AUGGUGAGAAAGCAGAAA
NC_004369/2797242-2797301      GUGCAGCAGACGACAAUGUC.GUCGACGc................cgAGGUCGUGGACG..AGGAUGUCUCCGAGGAGA
NZ_LT906441/1211974-1212044    UAUUUAUGUUCCACAGCAUU.GUGGGAUgaagcca.....aggaggaAAAUACAUGUCA..AAGAAGACUCAUGUGGCA
NC_016887/425172-425232        GUGACGGUAGACACAAAGGA.GUGCUCUga...............ugCUGGUGAAGUCG..AUGAAGACGCUGGCGCAG
NZ_CP008944/1161153-1161209    AACAACGCGACCUCAACGAU.GAGGAAC...................UGGCGCAGGUCA..ACGAGGAUCUCUACGGCC
NZ_CP007457/59941-59994        AUCGAGAUCACAGCAGAGAC.GCUGU--...................-ACAUUCCGUCG..AAGGAGAUGGAGCAGAUG
NZ_CP022544/60793-60846        AUCGAGAUCACAGCAGAGAC.GCUGU--...................-ACAUUCCGUCG..AAGGAGAUGGAGCAGAUG
NZ_AP017900/552424-552477      UCUAGUAGUAACACAAAGGA.GUGUC--...................-CUAUGCGGGCA..AUGCUGCCUCACUCUUCG
NZ_CP017839/204678-204731      UCUAGUAGUAACACAAAGGA.GUGUC--...................-CUAUGCGGGCA..AUGCUGCCUCACUCUUCG
NZ_CP017639/282870-282926      AGGCAGCCGACGACAACGUC.GUCGACG...................CCGAGGUUGUCG..ACGAAGACACCGACGAAA
NZ_CP014513/4176027-4176083    UUGACGGCGUGUACAUCGAU.GUACAUA...................CUGGCUAUUACA..ACGAUGUACUCGAAGGAG
NZ_CP013200/2477270-2477335    CCAUCAAGUACUUCAACGAC.GGAGAUCucgu..........cgaagGUGUAGUUGUCA..AGGUUGACCGCGACGAAG
NZ_CP012171/1710341-1710406    AACGUACUUGCGGGGAUGAU.CCUGUUAcccc..........gcaccACCGCAAAAGCA..AUGGUGCUUCUCUCGGUG
NZ_CP013297/2161633-2161698    AACGUACUUGCGGGGAUGAU.CCUGUUAcccc..........gcaccACCGCAAAAGCA..AUGGUGCUUCUCUCGGUG
NC_022438/1814538-1814594      GCAGCAUUUCUCCUAAUGUC.GGGACAU...................AGUUAUAUUGCA..AAGGAGCACUAUGUCCUG
NC_009480/2749689-2749746      GCGCUUCGCAUUACAACGAU.GUAAAGA..................uCGUCGCAUCACA..AAGGAGUGGUCAAUGGAU
NZ_CP009244/2166366-2166420    CUUAAUGAAAAGGCAACGGA.GCUUCU-...................-CGAAGACGUCG..AAGAAGACUAUGACCUCG
NZ_CP019705/2252952-2253006    CUUAAUGAAAAGGCAACGGA.GCUUCU-...................-CGAAGACGUCG..AAGAAGACUAUGACCUCG
NZ_CP012647/246614-246678      CUGAUGAGAGCAGCACAGCA.GCUGUGAgcuc...........gaguCCUUUGGAAGCA..AAGAGGCUUAUGACCAGC
NC_017550/1142874-1142938      CUGAUGAGAGCAGCACAGCA.GCUGUGAgcuc...........gaguCCUUUGGAAGCA..AAGAGGCUUAUGACCAGC
NC_016512/146779-146843        CUGAUGAGAGCAGCACAGCA.GCUGUGAgcuc...........gaguCCUUUGGAAGCA..AAGAGGCUUAUGACCAGC
NC_016511/146788-146852        CUGAUGAGAGCAGCACAGCA.GCUGUGAgcuc...........gaguCCUUUGGAAGCA..AAGAGGCUUAUGACCAGC
NC_016516/146791-146855        CUGAUGAGAGCAGCACAGCA.GCUGUGAgcuc...........gaguCCUUUGGAAGCA..AAGAGGCUUAUGACCAGC
NZ_CP019304/3170621-3170678    AUUAUUAGUUGGAAAGCGUU.UUCCUCA..................uCCUUCAAGAUCA..AAGAAGAUCGGAGUGAAC
NC_013441/3539601-3539668      AUCAUGUUCCUGGCAGGGAC.GUCGUUGaaggg........agcuugAGAAUGGCGGCA..AUGAAGCCACGUACUGGG
NZ_CP009251/658185-658236      GCGUUUACACGCGCCACGAU.GUGCU--...................---UAGUACGCA..AGGAUGCGGUUUACUAGU
NZ_CP022054/276098-276155      AUCAAAUCAUUU-CACGGAG.GUAAAUGu.................gCGAGUAUCCGCA..AUGACGCGGCGCGUGACG
NZ_CP008913/2170035-2170092    AUCAAAUCAUUU-CACGGAG.GUAAAUGu.................gCGAGUAUCCGCA..AUGACGCGGCGCGUGACG
NC_009077/2700344-2700396      AACAUCUGUUCCGCAUUAGG.GUGGAU-...................---CGGACCGCA..AGGGUGCGGACGACGAAA
NC_008705/2714603-2714655      AACAUCUGUUCCGCAUUAGG.GUGGAU-...................---CGGACCGCA..AGGGUGCGGACGACGAAA
NC_008146/2696715-2696767      AACAUCUGUUCCGCAUUAGG.GUGGAU-...................---CGGACCGCA..AGGGUGCGGACGACGAAA
NZ_CP014196/506425-506490      ACCGUGCUUUCCACAGAGAA.GUGAAAGucca..........aagcgAGAGAGUGUUCA..AUGAAGAAUAAAAUCCUG
NZ_LT906473/15053-15109        GAACUAACGACGACAACGUC.GUUGACG...................CCGAGGUUGUCG..AAGACGACGAGGCUGACA
NC_013441/5147932-5147989      UCGACUAUCGCGACAACGUC.GUCGCAU..................aUCUCCGCGGGCA..AUGACGCCCGCAUCCCGG
NC_013947/1917023-1917082      UUAACGAAAACCACAAGGAG.GUUGCAGu................cgUGAGAAGCAGCA..ACGGCGCUCUCAACAGAU
NC_013131/3728363-3728433      CACUAUUCGCCGACAAAGGC.GUUAUUGAcccuuu.....gugcgggCUCUGAUACGCA..UGGAAGCGUGAAGUCACG
NZ_LT827128/2127191-2127244    AGGAAGGCUUCUACACCGAC.GUGGU--...................-CGACAACGGCG..AAGAAGCCCUGGCCCUGG
NZ_CP011492/3795431-3795481    UGAAGAUCAUCCUCAAGGGU.GAGGU--...................----CUCCGGCC..UCGGUGCCGCGGGCGAUG
NC_013170/1081416-1081472      CAGAGGACAGGAGCAAUAGC.GCUCUCA...................CUGCUGAUAGCA..GUGGCGCUACUUCCUAUA
NZ_CP011340/8476818-8476874    AGAUAGCCGUCGGCAACGAC.GUCGAUG...................CUGUCCUCAUCG..CUGGCGAUCUCUACGAGA
NZ_CP011340/55719-55775        AGAUAGCCGUCGGCAACGAC.GUCGAUG...................CUGUCCUCAUCG..CUGGCGAUCUCUACGAGA
NC_013170/205749-205805        CAGAGGACAGGAGCAAUAGC.GCUCUCA...................CUGCUGAUAGCA..GUGGCGCUGCUUCCUAUA
NZ_CP009914/5424084-5424140    AACUUCUACCAGACAGCGGU.GUCGUGC...................AUGGACACGGCG..UGGAAGCCCCUGAUCACU
NC_022523/1727699-1727752      AUCAAUGGCUGAGCAAAUGU.GUUCC--...................-GAUUGCAUGCA..AUGAAGCAAACAUCUGCG
NZ_CP013747/1830155-1830214    GUAAAAGUCACAUCAAAGAG.GCUGUGGc................gcAGGACGUAUACA..AAGGGGUAGUAGUUCAAA
NZ_CP010407/142442-142501      AGCGACUGCUUGACAAGGAA.GUCAAGCa................guCGCUUGAGUUCA..AGGGUGAGGCCAGAGAAG
NC_018720/1405348-1405400      GAUUGGUACGGCUCAAUGAU.GAGCC--...................--ACUGAUUGCA..AGGCUGCACACGAAGGAG
NZ_CP017696/1186101-1186153    GAUUGGUACGGCUCAAUGAU.GAGCC--...................--ACUGAUUGCA..AGGCUGCACACGAAGGAG
NZ_CP018151/1648105-1648165    ACAAGUGCCAGGGCUAAGGG.GCCCUGGca...............cuCAAGAAAGGUCA..AUGAAGACAUGACUGAAG
NZ_CP012299/661110-661170      ACAAGUGCCAGGGCUAAGGG.GCCCUGGca...............cuCAAGAAAGGUCA..AUGAAGACAUGACUGAAG
NC_021663/266084-266134        ACUUUCUCGACGGUGACGAU.GCCGU--...................----CCUGGUCA..AGGUAGACGUCGGUGAAG
NC_015125/3169487-3169543      ACGAAUACAUCGAGAAGGCA.CUCGCGG...................AGCUGAAGGACG..AAGGCGUCACCGUCGACG
NZ_CP007456/1763522-1763578    AACCAGCUAUUUACCAAGUG.GUAGUUA...................GCUGACUGGGCA..GAGAAGCCCUAUCGGAAA
NZ_AP017369/3179917-3179973    GUGCUGCAGAUGACAAUGUU.GUUGACG...................CUGAAGUUGUCG..AAGACGACGCAGCUGACA
NC_010816/1451004-1451062      AAUGCGUAUUCUUCAAUGAA.GAAGGCAu.................cUGCUUCACCUCG..AAGAAGAGAUUAAGGAAA
NZ_AP014658/198738-198796      AAUGCGUAUUCUUCAAUGAA.GAAGGCAu.................cUGCUUCACCUCG..AAGAAGAGAUUAAGGAAA
NZ_CP013673/208320-208378      AAUGCGUAUUCUUCAAUGAA.GAAGGCAu.................cUGCUUCACCUCG..AAGAAGAGAUUAAGGAAA
NZ_CP010453/221198-221256      AAUGCGUAUUCUUCAAUGAA.GAAGGCAu.................cUGCUUCACCUCG..AAGAAGAGAUUAAGGAAA
NC_015052/201502-201560        AAUGCGUAUUCUUCAAUGAA.GAAGGCAu.................cUGCUUCACCUCG..AAGAAGAGAUUAAGGAAA
NZ_LN824140/2159815-2159873    AAUGCGUAUUCUUCAAUGAA.GAAGGCAu.................cUGCUUCACCUCG..AAGAAGAGAUUAAGGAAA
NC_014656/1432755-1432813      AAUGCGUAUUCUUCAAUGAA.GAAGGCAu.................cUGCUUCACCUCG..AAGAAGAGAUUAAGGAAA
NZ_CP006741/282168-282226      AAUGCGUAUUCUUCAAUGAA.GAAGGCAu.................cUGCUUCACCUCG..AAGAAGAGAUUAAGGAAA
NC_015067/216505-216563        AAUGCGUAUUCUUCAAUGAA.GAAGGCAu.................cUGCUUCACCUCG..AAGAAGAGAUUAAGGAAA
NC_017221/2053577-2053635      AAUGCGUAUUCUUCAAUGAA.GAAGGCAu.................cUGCUUCACCUCG..AAGAAGAGAUUAAGGAAA
NZ_CP011965/203778-203836      AAUGCGUAUUCUUCAAUGAA.GAAGGCAu.................cUGCUUCACCUCG..AAGAAGAGAUUAAGGAAA
```

NZ_CP011964/193570-193628	AAUGCGUAUUCUUCAAUGAA.GAAGGCAu..................cUGCUUCACCUCG..AAGAAGAGAUUAAGGAAA
NZ_CP009755/210718-210772	AUGAUCGAUUUGGCAACAUU.GCCGUC-....................-ACAUUCACGCA..AGGGAGCGAGAUGACCAC
NC_020891/2521533-2521590	GCGCUCCUCAUUACAACGAU.GUAAUGA...................uCACUGCAUCACG..AAGGAGUGGUCAAUGGAU
NZ_CP014869/2010339-2010393	CAUUAAACAUCAUCACUGGA.GAUGAC-....................-GAACGAUGACA..UGGGAGUUCCGAGCCACG
NC_022115/137578-137631	ACGAGAACGGCGGCGAGGAU.GCCGC--....................-AAUUCUUGACA..UUGACGUCAACGUCAACA
NZ_CP011295/137863-137916	ACGAGAACGGCGGCGAGGAU.GCCGC--....................-AAUUCUUGACA..UUGACGUCAACGUCAACA
NZ_CP011005/507759-507816	GGAGAAGUGGUAUCAGCGAU.GAUACCU...................uCUGCUUAUGGCA..AUGACGCUUCGCGUCAAC
NC_017999/647226-647282	AUGAUGCGAUGCGCGAUGUG.GCGUUCG....................AUGUCAAAAACA..GCGAUGUUCCAACCUUUU
NC_008538/84098-84153	AAACAUUCCCGCGUGAAGAU.ACGUCC-....................CAGGAGUGAUCA..AUGCGGAUCGAAGCCAAG
NC_015147/75561-75616	AAACAUUCCCGCGUGAAGAU.ACGUCC-....................CAGGAGUGAUCA..AUGCGGAUCGAAGCCAAG
NC_022198/34937-34993	CCGAAACCCACGACAAUGAC.GUCGUAG....................GUUUGCUCAGCG..GUGUUGCUUGGGGUGCUG
NC_012590/844165-844219	AUAAUCCCCAAUGGAAAAGC.CCGUAA-....................-UUGAGCCGGCA..ACGGAGCCUGCUCCCCAG
NZ_CP010071/1151605-1151667	UUACCGAUAGCGCCAAUGGC.GGCGUGCcgg..............ugaGGAGAAUUGUCA..AUGACGACAGUGCAUGAG
NZ_AP012325/1553467-1553523	AACCAGCUAGUUACCAAGUG.GUAGUUA....................GUUGAUUGGGCA..GAGAAGCCCUAUCGGAAA
NZ_AP012330/1670384-1670440	AACCAGCUAGUUACCAAGUG.GUAGUUA....................GUUGAUUGGGCA..GAGAAGCCCUAUCGGAAA
NC_015145/3706068-3706123	GCUCACACAGCCCCAAAGGU.GGGGUG-....................AUCACGAACACA..AGGGAGUGUUGUUCAUGC
NC_013715/83238-83293	ACCACCAGAUGCACAAAGAA.GUUUUG-....................UGCGGAUAUUCA..AAGGAGAAAAUCAUGACU
NZ_AP012323/63901-63952	AUGGUGACCGAGGCAGUGAA.GCCUA--....................---UGGCCGGCG..AUGUGGCCGGGAAUGACA
NC_014616/480016-480067	AUGGUGACCGAGGCAGUGAA.GCCUA--....................---UGGCCGGCG..AUGUGGCCGGGAAUGACA
NZ_CP006713/481305-481356	AUGGUGACCGAGGCAGUGAA.GCCUA--....................---UGGCCGGCG..AUGUGGCCGGGAAUGACA
NZ_CP010453/510303-510354	AUGGUGACCGAGGCAGUGAA.GCCUA--....................---UGGCCGGCG..AUGUGGCCGGGAAUGACA
NZ_CP010411/1097476-1097527	AUGGUGACCGAGGCAGUGAA.GCCUA--....................---UGGCCGGCG..AUGUGGCCGGGAAUGACA
NZ_CP010411/1998475-1998526	AUGGUGACCGAGGCAGUGAA.GCCUA--....................---UGGCCGGCG..AUGUGGCCGGGAAUGACA
NC_022567/1426157-1426207	GUUCAAUGGCUGAGAAAGUA.CUCAUG-....................-----AAGGGCA..ACGAGGCCAUGGCCGAGG
NZ_CP015407/352862-352928	AUAUACAAAACC-CUAGGCG.GCGGACGcguuu.........gcgccgACGUCUAGGGCA..AUGGAGCCACUUGACAGA
NC_015738/898918-898968	AGCGAGACGUGAGCAAAGGU.GCUGA--....................----CAAAGGCA..GUGAUGCCUUCGUAGCGC
NC_022657/9254943-9254997	GACAUCGGUGCUGCAACGGG.GCAGCU-....................-CGACGAACGCG..AAGGAGCGGUGCAUGUAC
NZ_CP013129/6497896-6497952	UGGAUGUCGACGUCAAUGCC.GAUGCCG....................AUGUCGCAGUCG..AUGACGAUGACGAUGACG
NZ_CP010453/2252785-2252838	UAAUUACAAACAUCAGAGAG.GAUGU--....................-GUCUCCAUGCA..UUGGAGCAAAGGAAAGAA
NZ_CP010797/4970838-4970892	AACAAAUGCUACGUAAAGGU.GCGUCA-....................-AAGAGAUGGUA..ACCGAGCCUGAAGUGACC
NC_020517/396033-396084	AUGGUGACCGAGGCAGUGAA.GCCUA--....................---UGGCCGGCG..AUGUGGCCGGGAACGACA
NC_017219/321389-321440	AUGGUGACCGAGGCAGUGAA.GCCUA--....................---UGGCCGGCG..AUGUGGCCGGGAACGACA
NC_011593/321598-321649	AUGGUGACCGAGGCAGUGAA.GCCUA--....................---UGGCCGGCG..AUGUGGCCGGGAACGACA
NZ_LT706985/581803-581857	UUCUAAUUCAUCAUGGAGAU.AUGACA-....................-UAUUUCAAACA..AUGGUGUUACAGCUAAAU
NC_018531/2363737-2363795	GCAUCCGGGGCUGCAAAGUG.GCAGUCCg..................gACUUGGGAAUCA..AAGGAGAUUAUGGUGACU
NC_016799/2366685-2366748	ACAAAAGAAAACGCAAAGUU.GCGCGAAgaa.............cgcgAUAUCCUACGCA..AGGCCGCGAAAUAUUUUG
NC_016800/2305800-2305863	ACAAAAGAAAACGCAAAGUU.GCGCGAAgaa.............cgcgAUAUCCUACGCA..AGGCCGCGAAAUAUUUUG
NZ_CP015622/2138588-2138650	UUUGUAUGUUUUUCAAUGUU.GAAGAUAuag..............uuaCAUCUCGUUGCA..AGGAAGCAGUUCCUGAAA
NZ_CP011489/1875760-1875820	AACAAUUGACCGCCCAUGGC.GGCGCAAug................caAUGUCUUUUGCA..GUGAUGCAGGGGCUCAGA
NZ_AP017975/1202932-1202999	AUGAUCGCAUAUGAAUUGCA.UUAUGUGcauca.........uggauuCAAUAGAUGGCA..ACGGAGCCGAAGACCAUC
NZ_AP012334/1529587-1529653	AUAAUAUUAUUAGCAGAAAA.GUUAAUGacggu..........ugguaAAUCCAAUCACA..AAGAGGUGGUCGCCUAUG
NC_010168/1074795-1074848	GUAACUGUCAGUUCAAGGAC.GAACA--....................-GACCUUUCGCA..AUGACGCGUGGGGUCGGA
NZ_CP006712/578515-578576	CAUAUAGCAGCUGCAAUGGG.GCAGCACac...............aauGGCAAGGAUUCA..AAGGAGAAUCAUGGGCAC
NC_019395/1187953-1188010	ACUUUUAUAAAUACAGCUCA.GUACUGU...................aUUGUUAUCGGCA..UGGACGCCAAUGACGAUG
NZ_CP013126/3485203-3485260	ACUUUUAUAAAUACAGCUCA.GUACUGU...................aUUGUUAUCGGCA..UGGACGCCAAUGACGAUG
NZ_CP019400/3442746-3442803	ACUUUUAUAAAUACAGCUCA.GUACUGU...................aUUGUUAUCGGCA..UGGACGCCAAUGACGAUG
NZ_CP007699/2624829-2624882	UCCACGACCGCGAGAUAGAU.CUCGA--....................-UGAAGUGCGCG..AAGUCGCGGAAGGUGAAG
NZ_CP012750/3057331-3057388	AGAAAGAAUUUGACAACGAU.GUCAGAA...................cUCAAUGUCGGCG..GCGGUGCCAAAUCCCGCC
NZ_CP014145/3382517-3382572	ACGAGGAGAAUU-CAAUGAC.GAAAAUC....................GGCGUACAGGCC..AUGAUGCUGAAAGACAAU
NZ_CP012479/1014978-1015039	AAAUGGCUAGCGACAGUGAC.GUUGUAGcc...............cuuUUGGAUGAAGUA..AAGUUACUCGGUAUAUCG
NZ_LT821227/813267-813315	AGAAUUUUUAUAACAAAGAC.GUUGUUU............................UUGA..AAAGCCAAAUGCGAUGAA
NC_006361/389573-389632	GUGACGGCAGACACAAAGGA.GUGUGCGa.................ugCUGGUGACGUCG..AUGAAGACCCUGGCGGGG
NC_015145/2273699-2273754	CAAGCAACAUACAAAACGUU.UUGCAA-....................AGGAACAGGUCA..AUGGCGACGAAGGUAAAC
NZ_CP023445/3895160-3895225	GAAUCAGUGAACGCAACGUA.GCGUUUGcgau...........gucagAAACAACGAGCG..CAGGAGCUGGGAACCAUG
NZ_CP016773/883188-883253	AUGCAACUGAAGUCAAGGAA.GGCUUUGagug...........cgguaUCGGCGUAGGCA..AGGGUGCUGAUCUUCAAG
NC_019958/29757-29809	UCAACGAAGAAGACAUCGAC.GUCUU--....................--CUUGAACGCA..UUGUCGCGACAAGGCAGC
NZ_CP018002/2035851-2035913	AACAAGGAGAGGUCAACGAU.GACGAAGguc..............aacGCUUCGAAUGCC..AUGCUGCAGGUGCUCAAG
NZ_CP010341/510623-510685	AACAAGGAGAGGUCAACGAU.GACGAAGguc..............aacGCUUCGAAUGCC..AUGCUGCAGGUGCUCAAG
NC_014215/540311-540373	AACAAGGAGAGGUCAACGAU.GACGAAGguc..............aacGCUUCGAAUGCC..AUGCUGCAGGUGCUCAAG
NZ_LN997841/2329779-2329841	AACAAGGAGAGGUCAACGAU.GACGAAGguc..............aacGCUUCGAAUGCC..AUGCUGCAGGUGCUCAAG
NZ_CP013200/1608213-1608269	UUCCUAAUGGCCACAAUGCG.GUGGAAG....................CGGCCCCUGUCA..AGGGUGAUAUAACCCGAC
NZ_CP011340/7513420-7513468	CCGCAUGCGGUGUCAACGAU.GACAU--....................------AGGUCA..ACGAUGACACCGCUGAAG
NZ_AP012325/279728-279782	UGCGAAAUCAGCACAAUGAA.GUUCUG-....................-CAUAUCGUGCA..UAGGAGCAGUGUCUUUCG
NZ_AP012332/852571-852627	UGAUGGACGUUAAGAAGGCU.CUUACCG....................AAGCCGAAGGCG..AUGUAGCUCGCGCUAAGG
NC_014644/991686-991742	UGAUGGACGUUAAGAAGGCU.CUUACCG....................AAGCCGAAGGCG..AUGUAGCUCGCGCUAAGG
NC_017456/909585-909641	UGAUGGACGUUAAGAAGGCU.CUUACCG....................AAGCCGAAGGCG..AUGUAGCUCGCGCUAAGG
NZ_CP007519/1401552-1401608	AUGGUAGCCUCGGCAAGGAC.GCCGCUG....................AGGUAGCUGCCG..AUGGAGGCGAGAGGGUCG
NZ_CP007519/1203784-1203837	ACCGGUAUUUCCGCCAUGAU.GCGGU--....................-UACAGGGGUCA..AUGCCGACAUCGAAGCCG
NC_013714/1424860-1424909	UAGAAAUAAAUUAGAACGUU.CUAAUU-....................------AUGACA..AAGUGGUCGACAAAGGAA
NZ_AP012326/1424863-1424912	UAGAAAUAAAUUAGAACGUU.CUAAUU-....................------AUGACA..AAGUGGUCGACAAAGGAA
NZ_CP012750/1968178-1968238	ACGAAGACGACGACACUGUA.GUCGAGCgc................ccUGACCAUGGCU..AAGCAGCCCUUGUCCCA

NC_011879/279898-279963	CUGAAUGCAUCGACAACGAC.GUUGCCAgccu..........gaccuGCGUCUGCGGCA..ACUCCGCCUCUGAUGAGG
NZ_CP021417/1615601-1615651	ACGAAAACCUGAACGAAGAG.GUUU---...................---GUAUGGCCA..AAGACGGCACCAACCGUG
NC_016114/4725761-4725819	CCAUUCGUCGCAACAAUGUC.GUUGCUCg.................aACUUCGAAUGCA..ACGUAGCGUUUCUCAGAU
NC_013739/1067888-1067939	AUCCGGGUAGCGAUACCGGA.AUCGC--...................---UAGUAGGCA..ACGAUGCCGGAAACGGUU

NZ_CP011773/3780281-3780340	AAUACUUCAACGAUGGCGAC.AUCGUUGa................ggGGACCAUCGUCA..AGGUUGACCGUGACGAAG
NZ_CP006713/560435-560496	CAUAUAGCAGCUGCAAUGGG.GCAGCACgc..............aauGGAAAGGAUUCA..AGGGAGAAUCAUGGGCAC
NZ_CP011402/1421754-1421804	UAGGUAUGGCUGAGAAAGUA.CUUAUG-...................-----AAGGGCA..ACGAGGCCCUUGCCGCAG
NZ_CP013859/1072639-1072688	CCCAGAUUUCUGGUAACGAU.ACCAA--...................-----CCGAACA..AGGGAGUUCUGAAUGAUU
NZ_CP014196/523749-523796	UACGUCCGGUCCGCAAUGAC.GCGA---...................------AGGGUA..AAGGAGCCCGCAUUGAAA
NZ_CP017421/3552882-3552929	UACGUCCGGUCCGCAAUGAC.GCGA---...................------AGGGUA..AAGGAGCCCGCAUUGAAA
NC_017834/1905417-1905476	GAGAAACACUUCACAGAGCA.GUGGAAGg................uuGUGGCCUAGUCG..AAGACGACGGUUGCAAAC
NZ_CP015407/750467-750526	GAGAAACACUUCACAGAGCA.GUGGAAGg................uuGUGGCCUAGUCG..AAGACGACGGUUGCAAAC
NZ_CP006711/775729-775796	CAAAAAGACAUUGAAACGCA.UCAAUAAauaa.........ucaaggAUAUCCAACGCA..AUGGCGCGUCAGACGGCA
NZ_CP006715/872535-872602	CAAAAAGACAUUGAAACGCA.UCAAUAAauaa.........ucaaggAUAUCCAACGCA..AUGGCGCGUCAGACGGCA
NC_017218/1334083-1334150	CAAAAAGACAUUGAAACGCA.UCAAUAAauaa.........ucaaggAUAUCCAACGCA..AUGGCGCGUCAGACGGCA
NZ_AP012324/788354-788421	CAAAAAGACAUUGAAACGCA.UCAAUAAauaa.........ucaaggAUAUCCAACGCA..AUGGCGCGUCAGACGGCA
NZ_CP006712/816764-816831	CAAAAAGACAUUGAAACGCA.UCAAUAAauaa.........ucaaggAUAUCCAACGCA..AUGGCGCGUCAGACGGCA
NZ_CP006714/819771-819838	CAAAAAGACAUUGAAACGCA.UCAAUAAauaa.........ucaaggAUAUCCAACGCA..AUGGCGCGUCAGACGGCA
NZ_CP006716/860681-860748	CAAAAAGACAUUGAAACGCA.UCAAUAAauaa.........ucaaggAUAUCCAACGCA..AUGGCGCGUCAGACGGCA
NC_020517/907358-907425	CAAAAAGACAUUGAAACGCA.UCAAUAAauaa.........ucaaggAUAUCCAACGCA..AUGGCGCGUCAGACGGCA
NZ_CP010433/1704565-1704615	UUCCGAUUGCAUGCAAUGAA.GCAAA--...................----CAUCUGCG..AUGAAGCAGUGGAAGAAG
NZ_CP017098/1704773-1704823	UUCCGAUUGCAUGCAAUGAA.GCAAA--...................----CAUCUGCG..AUGAAGCAGUGGAAGAAG
NZ_CP007755/1693904-1693954	UUCCGAUUGCAUGCAAUGAA.GCAAA--...................----CAUCUGCG..AUGAAGCAGUGGAAGAAG
NC_011835/830043-830093	UUCCGAUUGCAUGCAAUGAA.GCAAA--...................----CAUCUGCG..AUGAAGCAGUGGAAGAAG
NC_017866/1699110-1699160	UUCCGAUUGCAUGCAAUGAA.GCAAA--...................----CAUCUGCG..AUGAAGCAGUGGAAGAAG
NC_017214/396411-396461	UUCCGAUUGCAUGCAAUGAA.GCAAA--...................----CAUCUGCG..AUGAAGCAGUGGAAGAAG
NC_017867/1699329-1699379	UUCCGAUUGCAUGCAAUGAA.GCAAA--...................----CAUCUGCG..AUGAAGCAGUGGAAGAAG
NC_012814/1699274-1699324	UUCCGAUUGCAUGCAAUGAA.GCAAA--...................----CAUCUGCG..AUGAAGCAGUGGAAGAAG
NC_021593/1698898-1698948	UUCCGAUUGCAUGCAAUGAA.GCAAA--...................----CAUCUGCG..AUGAAGCAGUGGAAGAAG
NC_017216/1698919-1698969	UUCCGAUUGCAUGCAAUGAA.GCAAA--...................----CAUCUGCG..AUGAAGCAGUGGAAGAAG
NC_017215/1703861-1703911	UUCCGAUUGCAUGCAAUGAA.GCAAA--...................----CAUCUGCG..AUGAAGCAGUGGAAGAAG
NC_012815/1698991-1699041	UUCCGAUUGCAUGCAAUGAA.GCAAA--...................----CAUCUGCG..AUGAAGCAGUGGAAGAAG
NZ_CP007522/1698736-1698786	UUCCGAUUGCAUGCAAUGAA.GCAAA--...................----CAUCUGCG..AUGAAGCAGUGGAAGAAG
NZ_CP009045/1698908-1698958	UUCCGAUUGCAUGCAAUGAA.GCAAA--...................----CAUCUGCG..AUGAAGCAGUGGAAGAAG
NC_017217/1704560-1704610	UUCCGAUUGCAUGCAAUGAA.GCAAA--...................----CAUCUGCG..AUGAAGCAGUGGAAGAAG
NZ_CP011043/2403940-2403997	GCGCUCCGCAUUACAACGAU.GUAAUGA..................uCACUGCAUCACG..AAGGAGUGGUCAAUGGAU
NC_018531/648604-648665	GUUCUUCGAGACCCAACGGU.GGGUCACcc..............guuCACCAGGAGGCA..AUGUGGCCCUCAAUCUCA
NZ_CP007456/560537-560584	AACAUGUUUGCUGCANN---.GCAGCG-...................-----CUACGCA..AUGAAGCGCCCAAUCGAA
NC_009338/4909724-4909773	UAACAUUCGACGACAAUGGG.GUCGA--...................-----CGUGGCA..AUCGUGCCGUCCCCGAGU
NZ_CP012069/2778345-2778401	UUAAGUAUAACGUCAAUGGU.GACAUUG...................CUGAAAUAGUCG..AUGCGGAUGGCAUUGAGG
NC_008711/678551-678612	GUUCUUCGAGACCCAACGGU.GGGUCACcc..............guuCACCAGGAGGCA..AUGUGGCCCUCAAUCUCA
NZ_CP010437/431761-431813	AUAUAUUUAUCAACAAAAAA.GUUG---...................-AGACGGUGACA..ACGACGUUCUACCAAGAA
NZ_AP012325/392162-392214	AUAUAUUUAUCAACAAAAAA.GUUG---...................-AGACGGUGACA..ACGACGUUCUACCAAGAA
NZ_CP014313/1226163-1226223	CAGUCGCGGGUGGCAACGAC.GCCACUGac...............cuCGGGAGUCCUCA..AUGACGAGUGAAAAGACC
NZ_CP007456/2261564-2261614	UACCGCAUGGUUCCAAUGGU.GGAAC--...................----UCCAUACA..GCGAAGUAAGGAGAAAUA
NZ_CP013142/3516640-3516700	AGGAUGUGACCGACAAUGAC.GUCGCAUcc...............aaGGUUCCAGGUCA..GGGCCGACAGUACGCAGC
NZ_CP016770/10956-11027	AACAAAUUCUUAACAAGGCU.GUUGUCCacggcaa....agugauuuUGUCCUCAGGUA..AAGAGGCUGAUUACUACG
NZ_CP016772/10974-11045	AACAAAUUCUUAACAAGGCU.GUUGUCCacggcaa....agugauuuUGUCCUCAGGUA..AAGAGGCUGAUUACUACG
NC_003450/2984562-2984618	GCGCUGCAGAUGACAAUGUU.GUUGACG...................CUGAAGUUGUCG..AAGACGACGCAGCUGACA
NC_006958/2957867-2957923	GCGCUGCAGAUGACAAUGUU.GUUGACG...................CUGAAGUUGUCG..AAGACGACGCAGCUGACA
NC_020519/2984561-2984617	GCGCUGCAGAUGACAAUGUU.GUUGACG...................CUGAAGUUGUCG..AAGACGACGCAGCUGACA
NC_022040/2754414-2754470	GCGCUGCAGAUGACAAUGUU.GUUGACG...................CUGAAGUUGUCG..AAGACGACGCAGCUGACA
NC_009342/2977062-2977118	GCGCUGCAGAUGACAAUGUU.GUUGACG...................CUGAAGUUGUCG..AAGACGACGCAGCUGACA
NC_021351/3001096-3001152	GCGCUGCAGAUGACAAUGUU.GUUGACG...................CUGAAGUUGUCG..AAGACGACGCAGCUGACA
NC_021352/3001095-3001151	GCGCUGCAGAUGACAAUGUU.GUUGACG...................CUGAAGUUGUCG..AAGACGACGCAGCUGACA
NZ_CP020658/3060506-3060562	GCGCUGCAGAUGACAAUGUU.GUUGACG...................CUGAAGUUGUCG..AAGACGACGCAGCUGACA
NZ_CP013991/2931024-2931080	GCGCUGCAGAUGACAAUGUU.GUUGACG...................CUGAAGUUGUCG..AAGACGACGCAGCUGACA
NZ_CP016954/1895894-1895946	AACAUGAUGAAUGUGAGGAU.GUAU---...................-GACUACCGGCA..AUGAAGCCACGGGGCUAA
NZ_CP010437/550779-550840	GCCAUCAUUUUGUCGAUGAU.GACUUGGuc..............aauAAGGAGAAGACA..AUGAAGUUCAAAACCAUU
NZ_CP018134/2473648-2473705	AUGAUUCGAGCCGCAAUGAG.GCGAUCG..................gAGGACCCAGGUG..AUGGAGCCGGUGUGGAAC
NZ_CP011530/4394597-4394659	UUGAACUGCGCGACAACGGC.GUUGCCAcgc..............agaACGAUUCCGCCA..UUGGCGGCGUAGACCAUG
NC_008726/3573511-3573570	AAUACUUCAACGAUGGCGAC.AUCGUCGa................ggGAACCAUCGUCA..AGGUUGACCGUGACGAAG
NC_014643/1473946-1474000	AAAGGAAAAACUGCUAUGCC.GCAGAU-...................-UCUUGAUGGUA..AGGCAACCGCAGCCGCCA
NZ_CP019304/4972392-4972446	GCUCACGUAACCCCAAGGGU.GGGGUG-...................-UCACGAACACA..AGGGAGUGUUGUUCAUGA
NZ_CP018135/1469870-1469937	AUACAGAUUCCAGCAACGAU.GCUGAGAugaca........ggcgccACGGCGGAGAAG..AAGGAUUCCGCAAAGAAC
NZ_CP014145/2717332-2717386	UGCGGGAUCACCGCGAAGAC.GCGGGU-...................-GAGAAUCUACG..AAGGUGUAGGAAAUCAUG
NZ_CP019304/1250002-1250061	ACGGCUUCGAUGACGAUGAA.GUCGCCAu................caCGGUCAACGGCA..AGGAGGCCCGGUACGACC
NC_013159/3078960-3079028	ACCCUGAGGUUCACAACGAC.GUGGAUUccgggu.......ucuugaUCGUUCCCGGCA..AGGAAGUCCUUCACGGGG
NZ_CP007791/8740-8792	CCACUGUUUAUCUCAAGGAG.GAGA---...................-CGUGGAAGGCG..AUGAAGCUCAUGACCAUC
NZ_CP012750/1820450-1820512	CAAAUUCAAUCACCAAGGAU.GGUGUUGugc.............ggcUUUUGAUCGUCG..AGGACGACCAGGCAGUUG
NC_016802/2293876-2293939	ACAAAAGAAAACGCAAAGUU.GCGCGAAgaa............cacgAUAUCCUGCGCA..AGGCCGCGAAAUAUUUUG

NC_016787/2287584-2287647	ACAAAAGAAAACGCAAAGUU.GCGCGAAgaa............cacgAUAUCCUGCGCA..AGGCCGCGAAAUAUUUUG
NC_016788/2311402-2311465	ACAAAAGAAAACGCAAAGUU.GCGCGAAgaa............cacgAUAUCCUGCGCA..AGGCCGCGAAAUAUUUUG
NC_016790/2208578-2208641	ACAAAAGAAAACGCAAAGUU.GCGCGAAgaa............cacgAUAUCCUGCGCA..AGGCCGCGAAAUAUUUUG
NZ_CP017150/2225991-2226056	AAACUAUCACCGACGAUGUC.GUCGAAGcagc..........cgcgaGCAUCCUCGUCA..UCGGCGACUAACACCAUC
NC_019966/2977710-2977769	AAUACUUCAACGAUGGCGAC.AUCGUCGa................ggGAACAAUCGUCA..AGGUUGACCGUGACGAGG
NC_013714/166080-166138	AGUCACUUGAUGGAAACGGU.UCCAUCAa.................aCGUGAGAAAACA..UCGAGGUUUUCUCACAAG
NZ_AP012326/166081-166139	AGUCACUUGAUGGAAACGGU.UCCAUCAa.................aCGUGAGAAAACA..UCGAGGUUUUCUCACAAG
NZ_CP017150/1661329-1661374	AUGGGAAAUUUUCCAAAGGU.GGAGAU-...................---------GCA..AUGGAGC-UCUCAAAACG
NZ_CP012072/276999-277055	UCAAAGUGUGUCACAAUCGU.GUGAACC...................UAAUCAUGCUCA..AUGAGGAGUGAUCUAUGC
NZ_CP014952/3097993-3098053	AUGUUCAUGGCGCGAACGCC.CGCAUUUgc...............guAAGUCCAGCGCA..AAGAAGCGAUAACCCCGA
NZ_CP014145/15801-15867	GCGAUGGCCGCGGCAGCGAU.GCUGACUcgccg.........aagggAUCUAAACGACA..UUGUUGUCCUCCAUAAAG
NZ_CP010433/1662665-1662716	GAGUAAUCUGCGACGACGAU.GUCGC--...................---GGACCCGCA..ACGAAGCGAGGUGCUGUC
NZ_CP017098/1662873-1662924	GAGUAAUCUGCGACGACGAU.GUCGC--...................---GGACCCGCA..ACGAAGCGAGGUGCUGUC
NZ_CP007755/1652004-1652055	GAGUAAUCUGCGACGACGAU.GUCGC--...................---GGACCCGCA..ACGAAGCGAGGUGCUGUC
NZ_CP015407/1036608-1036659	GAGUAAUCUGCGACGACGAU.GUCGC--...................---GGACCCGCA..ACGAAGCGAGGUGCUGUC
NC_011835/788300-788351	GAGUAAUCUGCGACGACGAU.GUCGC--...................---GGACCCGCA..ACGAAGCGAGGUGCUGUC
NC_022523/1678573-1678624	GAGUAAUCUGCGACGACGAU.GUCGC--...................---GGACCCGCA..ACGAAGCGAGGUGCUGUC
NC_017866/1657210-1657261	GAGUAAUCUGCGACGACGAU.GUCGC--...................---GGACCCGCA..ACGAAGCGAGGUGCUGUC
NC_017214/354579-354630	GAGUAAUCUGCGACGACGAU.GUCGC--...................---GGACCCGCA..ACGAAGCGAGGUGCUGUC
NC_017867/1657429-1657480	GAGUAAUCUGCGACGACGAU.GUCGC--...................---GGACCCGCA..ACGAAGCGAGGUGCUGUC
NC_012814/1657373-1657424	GAGUAAUCUGCGACGACGAU.GUCGC--...................---GGACCCGCA..ACGAAGCGAGGUGCUGUC
NC_021593/1656999-1657050	GAGUAAUCUGCGACGACGAU.GUCGC--...................---GGACCCGCA..ACGAAGCGAGGUGCUGUC
NC_017216/1657019-1657070	GAGUAAUCUGCGACGACGAU.GUCGC--...................---GGACCCGCA..ACGAAGCGAGGUGCUGUC
NC_017215/1661994-1662045	GAGUAAUCUGCGACGACGAU.GUCGC--...................---GGACCCGCA..ACGAAGCGAGGUGCUGUC
NC_012815/1657091-1657142	GAGUAAUCUGCGACGACGAU.GUCGC--...................---GGACCCGCA..ACGAAGCGAGGUGCUGUC
NZ_CP007522/1656836-1656887	GAGUAAUCUGCGACGACGAU.GUCGC--...................---GGACCCGCA..ACGAAGCGAGGUGCUGUC
NZ_CP009045/1657008-1657059	GAGUAAUCUGCGACGACGAU.GUCGC--...................---GGACCCGCA..ACGAAGCGAGGUGCUGUC
NC_017217/1662660-1662711	GAGUAAUCUGCGACGACGAU.GUCGC--...................---GGACCCGCA..ACGAAGCGAGGUGCUGUC
NZ_CP024160/1874478-1874528	AAAAAGGGGCUCGCAAAGAU.GCGGCG-...................-----CCGGUUA..AAGGCAACCAAGGCAACG
NZ_CP007724/1164052-1164109	UUUUAUAUCCUAGCAAGGGU.GUUGCAU..................gAUGCAAUAAACA..UGGUAGUUUGUGUUCAUA
NZ_CP007722/1194869-1194926	UUUUAUAUCCUAGCAAGGGU.GUUGCAU..................gAUGCAAUAAACA..UGGUAGUUUGUGUUCAUA
NC_016799/608792-608851	AAUGCGGUGUCGCCAAAGCU.GGUGAAAg................cuCGAUAUUGGGCA..GUGGUGCCUUUUCUCGUU
NC_016801/564481-564540	AAUGCGGUGUCGCCAAAGCU.GGUGAAAg................cuCGAUAUUGGGCA..GUGGUGCCUUUUCUCGUU
NC_016785/554666-554725	AAUGCGGUGUCGCCAAAGCU.GGUGAAAg................cuCGAUAUUGGGCA..GUGGUGCCUUUUCUCGUU
NZ_LN831026/528602-528661	AAUGCGGUGUCGCCAAAGCU.GGUGAAAg................cuCGAUAUUGGGCA..GUGGUGCCUUUUCUCGUU
NC_016787/542020-542079	AAUGCGGUGUCGCCAAAGCU.GGUGAAAg................cuCGAUAUUGGGCA..GUGGUGCCUUUUCUCGUU
NC_016788/530537-530596	AAUGCGGUGUCGCCAAAGCU.GGUGAAAg................cuCGAUAUUGGGCA..GUGGUGCCUUUUCUCGUU
NC_002935/582246-582305	AAUGCGGUGUCGCCAAAGCU.GGUGAAAg................cuCGAUAUUGGGCA..GUGGUGCCUUUUCUCGUU
NC_016789/609691-609750	AAUGCGGUGUCGCCAAAGCU.GGUGAAAg................cuCGAUAUUGGGCA..GUGGUGCCUUUUCUCGUU
NZ_CP020410/2449468-2449527	AAUGCGGUGUCGCCAAAGCU.GGUGAAAg................cuCGAUAUUGGGCA..GUGGUGCCUUUUCUCGUU
NC_016790/519377-519436	AAUGCGGUGUCGCCAAAGCU.GGUGAAAg................cuCGAUAUUGGGCA..GUGGUGCCUUUUCUCGUU
NC_014218/88902-88960	UGGAAAAUACAGACAGCGUA.GUCUCUAu.................gUGGAACAUAUCA..AAGGGGAUAGUGAAAAUA
NC_014218/704515-704578	UUCAAACCGUAUUCAAAGGA.GAAUAUUgug............aaacUCAAGAAAAGCA..UUGGUGCUGUACUUGCCA
NZ_CP006715/602495-602556	CAUAUAGCAGCUGCAAUGGG.GCAGCACac..............aauGGCAAGGAUUCA..AGGGAGAAUCAUGGGCAC
NC_017218/1591103-1591164	CAUAUAGCAGCUGCAAUGGG.GCAGCACac..............aauGGCAAGGAUUCA..AGGGAGAAUCAUGGGCAC
NZ_AP012324/548829-548890	CAUAUAGCAGCUGCAAUGGG.GCAGCACac..............aauGGCAAGGAUUCA..AGGGAGAAUCAUGGGCAC
NZ_CP006714/580132-580193	CAUAUAGCAGCUGCAAUGGG.GCAGCACac..............aauGGCAAGGAUUCA..AGGGAGAAUCAUGGGCAC
NZ_CP006716/624023-624084	CAUAUAGCAGCUGCAAUGGG.GCAGCACac..............aauGGCAAGGAUUCA..AGGGAGAAUCAUGGGCAC
NZ_CP010413/1598844-1598905	CAUAUAGCAGCUGCAAUGGG.GCAGCACac..............aauGGCAAGGAUUCA..AGGGAGAAUCAUGGGCAC
NZ_CP019596/611153-611214	CAUAUAGCAGCUGCAAUGGG.GCAGCACac..............aauGGCAAGGAUUCA..AGGGAGAAUCAUGGGCAC
NC_020517/673821-673882	CAUAUAGCAGCUGCAAUGGG.GCAGCACac..............aauGGCAAGGAUUCA..AGGGAGAAUCAUGGGCAC
NZ_CP017812/254992-255054	AUAUCGUCAGCGCCAAGGUU.GGUGAUCccu.............uggGUCAUGAUGGUC..AUGAGACCCACAGCCAAA
NC_011886/404622-404675	ACAAUGUUUUCUAUAACGUU.GUAAA--...................-UCCAGAGAACG..AGGAUGUUUUUCGUGGCA
NC_008726/380445-380510	UGAUCCACGUCACCAAUGAU.GGUGCCGguua...........guaaaCCGUCAGCCGCA..UCGACGCGUCCCGGGAGG
NZ_AP012332/310381-310435	UACAUAUUUGAAUAAAUGAA.UCUUAU-...................-AAAAGCAUGCA..AAGGAGCAUCAUGUGUGA
NC_014644/449487-449541	UACAUAUUUGAAUAAAUGAA.UCUUAU-...................-AAAAGCAUGCA..AAGGAGCAUCAUGUGUGA
NC_017456/1437213-1437267	UACAUAUUUGAAUAAAUGAA.UCUUAU-...................-AAAAGCAUGCA..AAGGAGCAUCAUGUGUGA
NZ_CP005286/2213539-2213593	UUAAUGACAAGCUCUGUGUA.GAGUUA-...................-CAUCUCGUGCA..ACGGAGCAAGCCGCAAGG
NC_008711/2184804-2184862	GCAUCCGGGGCUGCAAAGUG.GCAGUCCg.................gACUUGGGAAUCA..AGGGAGAUUAUGGUGACU
NC_015145/618819-618869	AUCAGCGGAACGGCAAUGUU.GCCGA--...................-UGGUGAUGGCN..N---NGCCGAUGACCACA
NZ_CP014196/549436-549492	UGAACUUCGCCCGCAAGGUU.GCGGACC...................AGGUGGUGUUCA..UGGACGAAGGAGAAGUUG
NZ_CP017421/3527274-3527330	UGAACUUCGCCCGCAAGGUU.GCGGACC...................AGGUGGUGUUCA..UGGACGAAGGAGAAGUUG
NZ_CP012649/1076589-1076642	ACCGGUAUUUCCGCCAUGAU.GCGGU--...................-UACAGGAGUCA..AUGCCGACGUCGAGGCCG
NZ_CP007003/426192-426245	ACCGGUAUUUCCGCCAUGAU.GCGGU--...................-UACAGGAGUCA..AUGCCGACGUCGAGGCCG
NZ_CP006713/2153758-2153815	CUCGACAUGGCCGCGAAGAA.GCGGCUG..................aCGAACAAGUUCA..AAGCCGAAUACGCCAAGG
NZ_CP019606/2506764-2506818	ACUCUGAUAAUGACAGCGGU.GUGAUU-...................-UGGACGUAGCA..UGGGCGCUAUGUCUGAGG
NC_015145/3203787-3203847	ACUGAUCCGGUCCGAUUGAC.CGGAUGGca...............ucAAGGAGUCGGCA..AUGACGUCACAGGAAAAG
NZ_CP023778/7074978-7075034	AGGAACCAAACCGCAACGUU.GCGGUGG...................UCGGCUGGGUCA..UAGUGGAUCCAUGCCCGA
NZ_AP012323/1140470-1140529	AAUUCCAUGGGCUCAUCGAC.GAGUUCGg................caAAGCCUACCGCG..ACGAUGCGGCAAAAAACG
NC_017999/1082856-1082915	AAUUCCAUGGGCUCAUCGAC.GAGUUCGg................caAAGCCUACCGCG..ACGAUGCGGCAAAAAACG
NC_014638/1134880-1134939	AAUUCCAUGGGCUCAUCGAC.GAGUUCGg................caAAGCCUACCGCG..ACGAUGCGGCAAAAAACG

NC_014616/1104648-1104707	AAUUCCAUGGGCUCAUCGAC.GAGUUCGg................caAAGCCUACCGCG..ACGAUGCGGCAAAAAACG
NZ_CP010412/1159989-1160048	AAUUCCAUGGGCUCAUCGAC.GAGUUCGg................caAAGCCUACCGCG..ACGAUGCGGCAAAAAACG
NZ_LT821227/201305-201358	AAGAAAUUAUGUAUAAUGUU.AUGCG--...................-CGAAGUGAGCA..AAGCCGCUCCACCUUGGU
NZ_CP012750/2510824-2510880	UCCAAGUCAACACCGAUGAC.GGUGUCG...................AUCUUGCCGGCA..UCGGCGUCGGCGAGCAUC
NC_012704/1893895-1893951	ACGUUCACAACACCAAUGGU.GGUGUAA...................CACCAAUUAUCC..AGGGGGCUGCUGAUGAAU
NC_016604/4624964-4625023	AAUACUUCAACGAUGGCGAC.AUCGUCGa................agGGACCAUCGUCA..AGGUUGACCGUGACGAAG

NZ_CP014196/2015589-2015651	AUCAUGAAACCAGCAAAGAC.GCUGACAgga.............guaACACUGCUGGCA..GCGGUGUCGCUUGCGCUC
NZ_CP011005/2933728-2933785	CUACCGUUAUUAACAAAGGA.GUUAAUU..................uCACUAUGAAACG..ACGUAGUUUCUUCUCUAC
NC_016604/761068-761121	AGUCGCUGUGCCUCAACGUC.GAGGU--...................-GUUGUCGAGCG..ACGGUGCUGCGAUCGAAA
NZ_CP013290/2789043-2789103	UGUUUGAUUCGUGCAAUGAG.GCGCCGCgg...............cgCCAUGGUCUGCA..AGGAGGCAGUCAAUGAAG
NC_021279/64917-64969	CAACGAGCAACAGCACCGCA.GCUG---...................-CGAUGCUGCCA..ACGAAGGCUUUACCGAUG
NZ_CP007519/1802264-1802328	CUCAACCCGCCGUCGAAGGA.GACGGCGcgac...........gaaaGGAAUAGAAGCA..AUGGAGCUUCAAAAAAUU
NZ_CP007156/1654540-1654593	UGGAUAUCGUGGGCAAUGAU.GCCGA--...................-UGGCACGGUCA..UCGACGACAUCAUCGUGA
NZ_AP012323/686128-686184	AUGAUGCGAUGCGCGAUGUG.GCGUUCG...................AUGUCAAAAACA..GCGAUGUUCCAACGUUUU
NC_014638/681907-681963	AUGAUGCGAUGCGCGAUGUG.GCGUUCG...................AUGUCAAAAACA..GCGAUGUUCCAACGUUUU
NC_020506/97647-97710	UUAAUACACUGUGCAUUGAG.GCCCAAAuau............accuAAAACCAGAGCA..AUGGAGCUAAAGGCUAAA
NZ_CP018151/3238196-3238250	ACCACCGGCAUCUCAACGAG.GAGAAG-...................-AAAUGUCCGCG..AAGAAGCGCAUUCGCAUC
NZ_CP012299/2218058-2218112	ACCACCGGCAUCUCAACGAG.GAGAAG-...................-AAAUGUCCGCG..AAGAAGCGCAUUCGCAUC
NC_013714/672199-672249	UACUAGACCACAUGAAAGAA.CAUGUG-...................-----GUUCUCA..AGGAAGAGAAAUCGCAAG
NZ_AP012326/672200-672250	UACUAGACCACAUGAAAGAA.CAUGUG-...................-----GUUCUCA..AGGAAGAGAAAUCGCAAG
NZ_AP012327/2142256-2142313	AGCAAGUAAGCUAUACCGAU.UUAGCGU..................aGAGUGACAAGCA..AAGUGGCUAUCAAUGACA
NZ_CP012573/2513384-2513441	UGCGCUCGCAUUACAACGAU.GUAAUGA..................uCAUCGCAUCACG..AAGGAGUGGUCAAUGGAA
NZ_CP016773/315907-315957	AUCGCUUCAGGAAGAACGGU.CUUUA--...................----CAUUGGCU..UUGGUGCCGACAGGAAUA
NZ_CP011545/2111716-2111774	AAAACAGCUGUU-CAAAGCC.GAAAUGUu................gcUGGUAGAAGGCA..GCGGUGCCAGCCACAUUU
NZ_CP009244/2331593-2331655	UCGCAUUGGGCCACACAAAU.GUGGAAGcgg.............cagAAGAAAUGGGCA..UUGGCGCCGAGACCGUCA
NZ_CP019705/2418176-2418238	UCGCAUUGGGCCACACAAAU.GUGGAAGcgg.............cagAAGAAAUGGGCA..UUGGCGCCGAGACCGUCA
NZ_CP011341/2510931-2510983	GCAGUGCGGAACGCAACGGC.GCGU---...................-GCGAACAGACA..AGGAAGUCGAUCCUCCAG
NC_017219/2786609-2786662	UAUCCUUAUACUAAAGCGAU.UUAGU--...................-UCCGACCGGCA..AUGGUGCCAAAGAUGGUC
NC_011593/2790608-2790661	UAUCCUUAUACUAAAGCGAU.UUAGU--...................-UCCGACCGGCA..AUGGUGCCAAAGAUGGUC
NZ_CP010411/686899-686952	UAUCCUUAUACUAAAGCGAU.UUAGU--...................-UCCGACCGGCA..AUGGUGCCAAAGAUGGUC
NZ_AP012330/210641-210700	ACAUUGAUUUGUACAACGAU.GUAAUUUc................uuGUAUAGUGUACA..ACGUUGUAAAGAAAAGUC
NZ_CP010849/2043899-2043964	AAGAAUUACGCAACAAUGGC.GUUGUGAaaaacgu.........cggcgaggcucGGGA..GACC-CCCGUGGGGGCCC
NZ_CP013747/4278609-4278677	CGCACCUGGAAUCCAAUGGC.GGAUUCGucgacc.......ucguggACGUAGAGGGCA..AAGCCGCCGUCAUCGCAU
NZ_CP024160/245260-245313	CCCAGGCACUCGGCAACGAU.GCCGU--...................-CGAAUACGUCA..UGGACGACGCUCGCUUUG
NZ_CP012649/1566665-1566719	GGCGAAAUCGCUACAACGUA.GUAGCG-...................-CGAGACUUACA..AGGGAGUAGUCAUGGGAA
NZ_CP007519/659451-659505	GGCGAAAUCGCUACAACGUA.GUAGCG-...................-CGAGACUUACA..AGGGAGUAGUCAUGGGAA
NZ_CP007003/956842-956896	GGCGAAAUCGCUACAACGUA.GUAGCG-...................-CGAGACUUACA..AGGGAGUAGUCAUGGGAA
NC_015758/183316-183369	AUCGUCACCGACCGAAUGCU.CGGCA--...................-UGUUCAAGGCC..AAGAAGCCCGCCGUGCCA
NC_008769/214866-214919	AUCGUCACCGACCGAAUGCU.CGGCA--...................-UGUUCAAGGCC..AAGAAGCCCGCCGUGCCA
NZ_CP003494/4234506-4234559	AUCGUCACCGACCGAAUGCU.CGGCA--...................-UGUUCAAGGCC..AAGAAGCCCGCCGUGCCA
NC_020245/185199-185252	AUCGUCACCGACCGAAUGCU.CGGCA--...................-UGUUCAAGGCC..AAGAAGCCCGCCGUGCCA
NC_016804/185199-185252	AUCGUCACCGACCGAAUGCU.CGGCA--...................-UGUUCAAGGCC..AAGAAGCCCGCCGUGCCA
NZ_AM412059/185193-185246	AUCGUCACCGACCGAAUGCU.CGGCA--...................-UGUUCAAGGCC..AAGAAGCCCGCCGUGCCA
NC_012207/185199-185252	AUCGUCACCGACCGAAUGCU.CGGCA--...................-UGUUCAAGGCC..AAGAAGCCCGCCGUGCCA
NZ_CP014566/185199-185252	AUCGUCACCGACCGAAUGCU.CGGCA--...................-UGUUCAAGGCC..AAGAAGCCCGCCGUGCCA
NZ_CP008744/185198-185251	AUCGUCACCGACCGAAUGCU.CGGCA--...................-UGUUCAAGGCC..AAGAAGCCCGCCGUGCCA
NZ_CP009243/185196-185249	AUCGUCACCGACCGAAUGCU.CGGCA--...................-UGUUCAAGGCC..AAGAAGCCCGCCGUGCCA
NZ_CP012095/185167-185220	AUCGUCACCGACCGAAUGCU.CGGCA--...................-UGUUCAAGGCC..AAGAAGCCCGCCGUGCCA
NZ_CP009449/184747-184800	AUCGUCACCGACCGAAUGCU.CGGCA--...................-UGUUCAAGGCC..AAGAAGCCCGCCGUGCCA
NZ_CP013741/185196-185249	AUCGUCACCGACCGAAUGCU.CGGCA--...................-UGUUCAAGGCC..AAGAAGCCCGCCGUGCCA
NZ_CP015773/3435305-3435358	AUCGUCACCGACCGAAUGCU.CGGCA--...................-UGUUCAAGGCC..AAGAAGCCCGCCGUGCCA
NC_015848/191637-191690	AUCGUCACCGACCGAAUGCU.CGGCA--...................-UGUUCAAGGCC..AAGAAGCCCGCCGUGCCA
NZ_HG813240/183230-183283	AUCGUCACCGACCGAAUGCU.CGGCA--...................-UGUUCAAGGCC..AAGAAGCCCGCCGUGCCA
NC_020089/185188-185241	AUCGUCACCGACCGAAUGCU.CGGCA--...................-UGUUCAAGGCC..AAGAAGCCCGCCGUGCCA
NZ_CP002883/183287-183340	AUCGUCACCGACCGAAUGCU.CGGCA--...................-UGUUCAAGGCC..AAGAAGCCCGCCGUGCCA
NZ_CP002882/183286-183339	AUCGUCACCGACCGAAUGCU.CGGCA--...................-UGUUCAAGGCC..AAGAAGCCCGCCGUGCCA
NC_021251/183231-183284	AUCGUCACCGACCGAAUGCU.CGGCA--...................-UGUUCAAGGCC..AAGAAGCCCGCCGUGCCA
NC_017522/183230-183283	AUCGUCACCGACCGAAUGCU.CGGCA--...................-UGUUCAAGGCC..AAGAAGCCCGCCGUGCCA
NZ_CP002885/183230-183283	AUCGUCACCGACCGAAUGCU.CGGCA--...................-UGUUCAAGGCC..AAGAAGCCCGCCGUGCCA
NC_002755/185137-185190	AUCGUCACCGACCGAAUGCU.CGGCA--...................-UGUUCAAGGCC..AAGAAGCCCGCCGUGCCA
NC_017524/184725-184778	AUCGUCACCGACCGAAUGCU.CGGCA--...................-UGUUCAAGGCC..AAGAAGCCCGCCGUGCCA
NC_021740/184973-185026	AUCGUCACCGACCGAAUGCU.CGGCA--...................-UGUUCAAGGCC..AAGAAGCCCGCCGUGCCA
NC_009565/185285-185338	AUCGUCACCGACCGAAUGCU.CGGCA--...................-UGUUCAAGGCC..AAGAAGCCCGCCGUGCCA
NC_009525/186329-186382	AUCGUCACCGACCGAAUGCU.CGGCA--...................-UGUUCAAGGCC..AAGAAGCCCGCCGUGCCA
NZ_CP009480/184815-184868	AUCGUCACCGACCGAAUGCU.CGGCA--...................-UGUUCAAGGCC..AAGAAGCCCGCCGUGCCA
NC_018143/184967-185020	AUCGUCACCGACCGAAUGCU.CGGCA--...................-UGUUCAAGGCC..AAGAAGCCCGCCGUGCCA
NC_000962/184969-185022	AUCGUCACCGACCGAAUGCU.CGGCA--...................-UGUUCAAGGCC..AAGAAGCCCGCCGUGCCA
NZ_CP007027/184967-185020	AUCGUCACCGACCGAAUGCU.CGGCA--...................-UGUUCAAGGCC..AAGAAGCCCGCCGUGCCA
NZ_CP002871/183231-183284	AUCGUCACCGACCGAAUGCU.CGGCA--...................-UGUUCAAGGCC..AAGAAGCCCGCCGUGCCA
NZ_CP007803/183165-183218	AUCGUCACCGACCGAAUGCU.CGGCA--...................-UGUUCAAGGCC..AAGAAGCCCGCCGUGCCA
NC_012943/184908-184961	AUCGUCACCGACCGAAUGCU.CGGCA--...................-UGUUCAAGGCC..AAGAAGCCCGCCGUGCCA

NC_016768/184907-184960	AUCGUCACCGACCGAAUGCU.CGGCA--...................-UGUUCAAGGCC..AAGAAGCCCGCCGUGCCA
NC_018078/184906-184959	AUCGUCACCGACCGAAUGCU.CGGCA--...................-UGUUCAAGGCC..AAGAAGCCCGCCGUGCCA
NC_021054/184956-185009	AUCGUCACCGACCGAAUGCU.CGGCA--...................-UGUUCAAGGCC..AAGAAGCCCGCCGUGCCA
NC_020559/184282-184335	AUCGUCACCGACCGAAUGCU.CGGCA--...................-UGUUCAAGGCC..AAGAAGCCCGCCGUGCCA
NC_022350/185287-185340	AUCGUCACCGACCGAAUGCU.CGGCA--...................-UGUUCAAGGCC..AAGAAGCCCGCCGUGCCA
NZ_AP014573/186176-186229	AUCGUCACCGACCGAAUGCU.CGGCA--...................-UGUUCAAGGCC..AAGAAGCCCGCCGUGCCA
NZ_CP010339/185184-185237	AUCGUCACCGACCGAAUGCU.CGGCA--...................-UGUUCAAGGCC..AAGAAGCCCGCCGUGCCA
NZ_CP010337/185165-185218	AUCGUCACCGACCGAAUGCU.CGGCA--...................-UGUUCAAGGCC..AAGAAGCCCGCCGUGCCA
NZ_CP010340/185418-185471	AUCGUCACCGACCGAAUGCU.CGGCA--...................-UGUUCAAGGCC..AAGAAGCCCGCCGUGCCA
NZ_CP010338/185172-185225	AUCGUCACCGACCGAAUGCU.CGGCA--...................-UGUUCAAGGCC..AAGAAGCCCGCCGUGCCA
NZ_CP009426/182314-182367	AUCGUCACCGACCGAAUGCU.CGGCA--...................-UGUUCAAGGCC..AAGAAGCCCGCCGUGCCA
NZ_CP009427/185032-185085	AUCGUCACCGACCGAAUGCU.CGGCA--...................-UGUUCAAGGCC..AAGAAGCCCGCCGUGCCA
NZ_CP010330/184967-185020	AUCGUCACCGACCGAAUGCU.CGGCA--...................-UGUUCAAGGCC..AAGAAGCCCGCCGUGCCA
NZ_CP007809/182372-182425	AUCGUCACCGACCGAAUGCU.CGGCA--...................-UGUUCAAGGCC..AAGAAGCCCGCCGUGCCA
NZ_CP012506/181412-181465	AUCGUCACCGACCGAAUGCU.CGGCA--...................-UGUUCAAGGCC..AAGAAGCCCGCCGUGCCA
NZ_CP009100/185023-185076	AUCGUCACCGACCGAAUGCU.CGGCA--...................-UGUUCAAGGCC..AAGAAGCCCGCCGUGCCA
NZ_CP009101/184988-185041	AUCGUCACCGACCGAAUGCU.CGGCA--...................-UGUUCAAGGCC..AAGAAGCCCGCCGUGCCA
NZ_CP012090/183231-183284	AUCGUCACCGACCGAAUGCU.CGGCA--...................-UGUUCAAGGCC..AAGAAGCCCGCCGUGCCA
NZ_CP008885/1867745-1867806	ACCAACCCGUAUUCAAGGAG.GAAUAAUga..............gucGAUUCAAUGGCA..AACGCGCCGAGUUCGAGG
NC_014169/1864966-1865027	ACCAACCCGUAUUCAAGGAG.GAAUAAUga..............gucGAUUCAAUGGCA..AACGCGCCGAGUUCGAGG
NZ_AP012332/84407-84463	AAAACAUCAUAUGCAUAGAU.GCAUAGU...................GUGAUUCUCUCA..AAAGAGAGGAGUAUAUGA
NC_014644/223569-223625	AAAACAUCAUAUGCAUAGAU.GCAUAGU...................GUGAUUCUCUCA..AAAGAGAGGAGUAUAUGA
NC_017456/86028-86084	AAAACAUCAUAUGCAUAGAU.GCAUAGU...................GUGAUUCUCUCA..AAAGAGAGGAGUAUAUGA
NZ_CP024160/1415725-1415774	CAUUGCGAAAUGACAACGUU.GUCAUU-...................------UCGUUA..AUGACAACGUAAGACAUU
NZ_CP012477/285368-285421	AGGGAACCAGUGACAACGGU.GUCAG--...................-UAUUACAUGGA..AAGAGCCGCACGCCAACG
NZ_LT706985/1275582-1275638	UUAGAUACGUUCUCAGAGUC.GAGAGCU...................ACAAAGAUGACA..AGGAGGUCAGCUAAUGUC
NZ_CP009220/2492961-2493014	ACAUCAUUAUCUGCAAAGAC.G-AGCA-...................-AGAAGUUGGCA..AAGAAGCUGCAGCCCUAA
NZ_CP011112/3020465-3020530	CGAUCAAGCACUUCAAUGAC.GGAGACAucgu..........cgaggGUGUCAUCGUCA..AGGUCGACCGGGACGAAG
NC_023036/4181172-4181233	AGCUUGCACGGAUCAAUGGC.GAUCCUGuu..............agcCUUUUGAUCACA..AAGGUGUGUAACAAAUCA
NZ_CP011022/4181243-4181304	AGCUUGCACGGAUCAAUGGC.GAUCCUGuu..............agcCUUUUGAUCACA..AAGGUGUGUAACAAAUCA
NZ_CP014475/3983075-3983127	AAAGAGCCGGCCACAACGAU.GUCGUU-...................---UUUCCGCCA..GUGCUGGCGGUACCCACG
NZ_CP015732/1628239-1628298	UGGAAAGUCACACCAACGAA.GUUGUGAc................aaCGAACUCAUACA..AAGGGGUAAAAGUUCAUG
NZ_CP015603/195101-195172	AACAGAUUCUUAACAAAGCU.GUGGUCCacggcaa....agucaucuUGUCUUCAGGCA..AAGAGGCUGAUUACUACG
NC_014656/304657-304709	ACAAUCUGUUUCACAAAGGA.GUGU---...................-GUACUAUGGCU..UUGAAGCCUCGCACUGGU
NZ_CP011542/1400440-1400500	UGAAGAACUAUCGCAAUGGU.GCGACCAgc...............acCAAUACCCGGCC..AAGCGGCCGCGAACACGA
NC_008268/7784251-7784307	UGCAGGUCCCUGCCAAGGGU.GGCGGGA...................UCGAUGUCGGCA..UGGAAGCCGCGAAUCACU
NZ_AP012330/429639-429691	AUAUGUUUAUCAACAAAAAA.GUUG---...................-AGACGGUGACA..ACGACGUUCUACCAAGAA
NZ_CP011269/3616949-3617008	AAUACUUCAACGAUGGCGAC.AUCGUCGa................ggGAACCAUCGUCA..AGGUUGACCGUGACGAGG
NZ_CP014475/2811500-2811559	AAUACUUCAACGAUGGCGAC.AUCGUCGa................ggGAACCAUCGUCA..AGGUUGACCGUGACGAGG
NZ_CP009914/3626122-3626181	AAUACUUCAACGAUGGCGAC.AUCGUCGa................ggGAACCAUCGUCA..AGGUUGACCGUGACGAGG
NZ_CP013747/3355696-3355746	ACCAAGAGGUAAACAAUGUC.GUUUU--...................----CUCCGUCC..AAGAGGACAAAGGCACUG
NZ_LT821227/1387665-1387731	GUUAGCUGAUUGACAGCGGU.GUCAGACauaaa.........aauuuUACAAGCCGACA..AUGGGGUCAGACAUGAUG
NZ_CP020567/17269-17329	AUCAAUGGCACGACAUUGAU.GUCGCGAca...............ucAAUGACACAACA..UCGAUGUUGUGUCAUGAU
NZ_CP023778/8308556-8308608	AUAGCGGCGAACACAAAGGA.GUGCU--...................--CAUGCUGGUG..AAGAUGCCCGAGGCGAUG
NC_014218/123085-123135	GGAUGAAGCUCAGUAAAGAU.ACUGU--...................----UAUUCGCU..AUGUUGCGGAACUAGAAG
NC_013174/2323554-2323610	CGAUCGGCGUUCUCAACGGA.GAGGAAC...................ACCAUGCACGCA..ACGACGCGAGCAGUCACC
NZ_CP022038/1554011-1554067	CGAUCGGCGUUCUCAACGGA.GAGGAAC...................ACCAUGCACGCA..ACGACGCGAGCAGUCACC
NZ_CP011309/641800-641856	UUGUUGAGCUUCUCAAUGCC.GGUAAAC...................AAGUAGUUGUCA..UUGAUGAUCUUUCCAACA
NZ_CP022614/638553-638609	UUGUUGAGCUUCUCAAUGCC.GGUAAAC...................AAGUAGUUGUCA..UUGAUGAUCUUUCCAACA
NC_009342/735510-735566	UUGUUGAGCUUCUCAAUGCC.GGUAAAC...................AAGUAGUUGUCA..UUGAUGAUCUUUCCAACA
NC_021351/722518-722574	UUGUUGAGCUUCUCAAUGCC.GGUAAAC...................AAGUAGUUGUCA..UUGAUGAUCUUUCCAACA
NC_021352/722519-722575	UUGUUGAGCUUCUCAAUGCC.GGUAAAC...................AAGUAGUUGUCA..UUGAUGAUCUUUCCAACA
NZ_CP014984/636972-637028	UUGUUGAGCUUCUCAAUGCC.GGUAAAC...................AAGUAGUUGUCA..UUGAUGAUCUUUCCAACA
NZ_CP009716/2091358-2091409	GUCAUGGUAGCCACAGUGAU.GUGGG--...................---UGAUGCUCA..UGGGAGAGCUCUCCGUGG
NZ_CP009583/2122575-2122626	GUCAUGGUAGCCACAGUGAU.GUGGG--...................---UGAUGCUCA..UGGGAGAGCUCUCCGUGG
NZ_CP018762/817552-817601	GGAGUGUUCGAUACAGCGCU.GUAUC--...................-----CGAUACA..UAGAUGUAUCCUAAGAGU
NZ_CP020567/651063-651111	GUCAAGCGGAUGUCAACGCU.GACAU--...................------AAGUCA..ACGAUGACAGUGCUGGUA
NZ_HG917972/2228845-2228895	ACAUUUGAGGCGUUAAUGUA.AACGU--...................----CCAAGGCA..AGGGGGCCUUAUGUUCAC
NZ_CP016282/2539403-2539464	AAGCACCUCAACGCAAAGGA.GCGUCAUcg..............ugaAAGCACUCGUCU..ACGACGGCCCCGGUCAGA
NZ_AP012332/1391921-1391978	GAGAUUUACAAAAUAGAGAU.GUUUUUA..................uUUAUUCAAAGCA..AAGGAGCUGUUUAUAUGA
NC_014644/1531024-1531081	GAGAUUUACAAAAUAGAGAU.GUUUUUA..................uUUAUUCAAAGCA..AAGGAGCUGUUUAUAUGA
NZ_AP017369/1657111-1657172	UGCUUAGAAUUAACAACGGU.GUUGUUUug..............aauAAAACAUUGAUA..CUGACAUCGUUGCUGACA
NC_018681/466426-466485	GUGGCGGCAGACACAAAGGA.GUGCUCGa................ugCUGGUGAAGUCG..AUGAAGACGCUGGCGGAC
NZ_CP022088/6086749-6086808	GUGGCGGCAGACACAAAGGA.GUGCUCGa................ugCUGGUGAAGUCG..AUGAAGACGCUGGCGGAC
NZ_CP019229/146474-146536	ACGAUAAGGUCCGCAACGCC.GCUGAAGagg.............ccuCUGGCAAGGUCA..AGGAAGGCGUGGGCAAGG
NC_017300/1995873-1995926	AGAACAGCUUUAUCGACGUC.GAUGA--...................-ACGCUCAGGCA..AGGCAGCUUCCGAUCAUG
NZ_CP012837/854118-854171	AGAACAGCUUUAUCGACGUC.GAUGA--...................-ACGCUCAGGCA..AGGCAGCUUCCGAUCAUG
NZ_CP011474/1998115-1998168	AGAACAGCUUUAUCGACGUC.GAUGA--...................-ACGCUCAGGCA..AGGCAGCUUCCGAUCAUG
NZ_CP014998/2002902-2002955	AGAACAGCUUUAUCGACGUC.GAUGA--...................-ACGCUCAGGCA..AGGCAGCUUCCGAUCAUG
NZ_CP019769/1997836-1997889	AGAACAGCUUUAUCGACGUC.GAUGA--...................-ACGCUCAGGCA..AGGCAGCUUCCGAUCAUG
NZ_CP015309/1989095-1989148	AGAACAGCUUUAUCGACGUC.GAUGA--...................-ACGCUCAGGCA..AGGCAGCUUCCGAUCAUG

NZ_CP019768/854103-854156	AGAACAGCUUUAUCGACGUC.GAUGA--...................-ACGCUCAGGCA..AGGCAGCUUCCGAUCAUG
NZ_CP020356/854070-854123	AGAACAGCUUUAUCGACGUC.GAUGA--...................-ACGCUCAGGCA..AGGCAGCUUCCGAUCAUG
NZ_CP015100/1997875-1997928	AGAACAGCUUUAUCGACGUC.GAUGA--...................-ACGCUCAGGCA..AGGCAGCUUCCGAUCAUG
NZ_CP009927/1997850-1997903	AGAACAGCUUUAUCGACGUC.GAUGA--...................-ACGCUCAGGCA..AGGCAGCUUCCGAUCAUG
NZ_AP017457/1187659-1187715	AGCACAAGUUAGGCAGUGGC.GCUUUCG...................GUGUCGUUUUCA..AAGCUGAGAAAAACGACA
NZ_CP014518/1024253-1024306	UCUGUGACGCCCGCAAUGAC.GCGGC--...................-UCCAGGAGGAG..UCGGAUCCCGUAGGCAGG
NZ_CP008953/1154197-1154251	AUACCUUUGCAAGCAAAGAG.GCUUUG-...................-CUUGCAAAGCA..AAAUAGCUUUCACGCUGA
NC_018720/389675-389732	AUGUCAGAGUUUCCGAUGAU.GGAAUCU..................gAUACUUUGAGCA..UAGAAGCUUGAGUGAUGA
NZ_CP017696/170394-170451	AUGUCAGAGUUUCCGAUGAU.GGAAUCU..................gAUACUUUGAGCA..UAGAAGCUUGAGUGAUGA
NZ_CP017150/3773876-3773930	AAGAACCUAGUAUUAAUGUU.AAUAAU-...................-ACUCGUUCUCA..AUAAGGAGUUCAGCAAUG
NC_014246/1518925-1518981	GAGAAAUAAUGCAGAAGCCU.CUGGUUG...................AGGAAAUCGGCA..AAGCCGCCAUGCGUGAGG
NC_017834/363482-363548	AUAUGCAAAACC-CUAGGCG.GCGGACGcguuu........gcgccgACGUCUAGGGCA..AUGGAGCCACUUGACAGA
NZ_CP015235/133127-133189	CACACCACUUCGACAACGAU.GUCGAAGgag.............ccuGCCGUGAGGACC..ACGUAGUCGAUCGCGUCG
NZ_CP015220/3411253-3411315	CACACCACUUCGACAACGAU.GUCGAAGgag.............ccuGCCGUGAGGACC..ACGUAGUCGAUCGCGUCG
NZ_CP014869/2170321-2170380	AGUACUUCAACGAUGGCGAC.AUCGUUGa................ggGCGAAGUCGUCA..AGGUCGACCGUGACGAAG
NC_020133/2276480-2276530	ACAUAUGAGGCGUUAAUGUA.AACGU--...................----CCAAGGCA..AGGGGGCCUUAUGUUCAC
NC_010612/2346618-2346668	ACAUAUGAGGCGUUAAUGUA.AACGU--...................----CCAAGGCA..AGGGGGCCUUAUGUUCAC
NZ_CP017717/5653608-5653656	ACACAGCCGAUGUCAACGAU.GACAU--...................------AUGUCA..ACGAUGACAUCGAAUCCC
NZ_AP017457/1240808-1240861	GCAAAGUUUUCAAUGAUGCC.GUUGU--...................-GAAUGAGGGCA..AGGUUGCCACCGUCACCU
NC_018142/301894-301959	GGACAAAGCUUAGCAAAGUU.GCUACCGuauc..........gguagUACAACCGAGCA..UGGGAGUUACUACUCAAU
NZ_AP012323/1744913-1744975	AAACCGCUGGAAACACUGGG.GUUUGUGgua.............ccgUCACUGUGUGCG..AGGGCGCACACAGUGACG
NC_017999/1733339-1733401	AAACCGCUGGAAACACUGGG.GUUUGUGgua.............ccgUCACUGUGUGCG..AGGGCGCACACAGUGACG
NC_014638/1740814-1740876	AAACCGCUGGAAACACUGGG.GUUUGUGgua.............ccgUCACUGUGUGCG..AGGGCGCACACAGUGACG
NC_014616/1713216-1713278	AAACCGCUGGAAACACUGGG.GUUUGUGgua.............ccgUCACUGUGUGCG..AGGGCGCACACAGUGACG
NZ_CP010412/1806907-1806969	AAACCGCUGGAAACACUGGG.GUUUGUGgua.............ccgUCACUGUGUGCG..AGGGCGCACACAGUGACG
NC_017456/324254-324308	AUUUACAAAGUAGAGAUGUU.UUUAUU-...................-UAUUCAAAGCA..AAGGAGCUGUUUAUAUGA
NZ_CP016793/7916360-7916418	UGAUGCAAUUUGAGAGCGCU.CUCAUGUu.................cGUCGCCUCGGCA..ACGGUGCCGGUGGCGAUC
NZ_LN831039/3141672-3141723	AGAAAGGCAGUCGAGAUGAA.UCGAU--....................---UGGACGGCA..AGGUCGCCAUCGUCACCG
NC_008596/3078757-3078808	AGAAAGGCAGUCGAGAUGAA.UCGAU--....................---UGGACGGCA..AGGUCGCCAUCGUCACCG
NC_018289/3080371-3080422	AGAAAGGCAGUCGAGAUGAA.UCGAU--....................---UGGACGGCA..AGGUCGCCAUCGUCACCG
NZ_CP009494/3078802-3078853	AGAAAGGCAGUCGAGAUGAA.UCGAU--....................---UGGACGGCA..AGGUCGCCAUCGUCACCG
NZ_CP009495/3078836-3078887	AGAAAGGCAGUCGAGAUGAA.UCGAU--....................---UGGACGGCA..AGGUCGCCAUCGUCACCG
NZ_CP009496/3078824-3078875	AGAAAGGCAGUCGAGAUGAA.UCGAU--....................---UGGACGGCA..AGGUCGCCAUCGUCACCG
NZ_CP010407/2463683-2463738	ACAAGUACCUCCGCAAGGAC.GCGGCC-...................GGGAACGUGACC..UGGGAGUCCGGUGCCAAC
NC_009342/1256913-1256970	AUUUAUAUCCUAGCAGGGGU.GUUGCAU..................cAUGCAAUAAACA..UGGUAGUUUGUGUUCAUA
NZ_CP021252/192948-193016	AAACCCCCACCCGCACUGCU.GUGGUGGcagaac.......ucgucuCUUUGGUGGACA..AGGUCGUCUCCGGUCAGU
NZ_CP006715/258222-258273	CAGGGGAAUCAGACAAUGCU.GUCUU--....................---ACGACAGCA..UUGCGGCUAGAUGGAAAU
NC_017218/282198-282249	CAGGGGAAUCAGACAAUGCU.GUCUU--....................---ACGACAGCA..UUGCGGCUAGAUGGAAAU
NZ_CP006714/253813-253864	CAGGGGAAUCAGACAAUGCU.GUCUU--....................---ACGACAGCA..UUGCGGCUAGAUGGAAAU
NZ_CP006716/285280-285331	CAGGGGAAUCAGACAAUGCU.GUCUU--....................---ACGACAGCA..UUGCGGCUAGAUGGAAAU
NC_020517/300568-300619	CAGGGGAAUCAGACAAUGCU.GUCUU--....................---ACGACAGCA..UUGCGGCUAGAUGGAAAU
NZ_CP008885/239917-239968	CAGGGGAAUCAGACAAUGCU.GUCUU--....................---ACGACAGCA..UUGCGGCUAGAUGGAAAU
NZ_CP010411/1001169-1001220	CAGGGGAAUCAGACAAUGCU.GUCUU--....................---ACGACAGCA..UUGCGGCUAGAUGGAAAU
NC_014169/239921-239972	CAGGGGAAUCAGACAAUGCU.GUCUU--....................---ACGACAGCA..UUGCGGCUAGAUGGAAAU
NZ_AP012322/1197777-1197844	AACGGCACGGCUGCAUUGCU.GCAGUCGugccg........uuuugcAUUCCAUGGGCG..UCGGGGCCGAAUCCCACA
NZ_CP011773/4094465-4094521	GACGAGAAGUCGUCAUCGAC.GACGAGC...................ACCUCGACGUCA..ACGUCGACGUCGACCACG
NZ_AP014658/631129-631190	AUAAAUUAUGGUAUAAUGCAaGUAUGAGuu...............gcUUGGUUUGCGCA..AUGAAGCGGAGGCUCAAU
NC_015052/629092-629153	AUAAAUUAUGGUAUAAUGCAaGUAUGAGuu...............gcUUGGUUUGCGCA..AUGAAGCGGAGGCUCAAU
NZ_LN824140/153738-153799	AUAAAUUAUGGUAUAAUGCAaGUAUGAGuu...............gcUUGGUUUGCGCA..AUGAAGCGGAGGCUCAAU
NZ_CP011965/637500-637561	AUAAAUUAUGGUAUAAUGCAaGUAUGAGuu...............gcUUGGUUUGCGCA..AUGAAGCGGAGGCUCAAU
NZ_CP007287/1691827-1691880	GUGCUCGCCACAACAAAGGA.GUUAU--...................-CCAUGCAGCCA..AUGUCGGCAUUCACCACA
NZ_CP006018/1664773-1664826	GUGCUCGCCACAACAAAGGA.GUUAU--...................-CCAUGCAGCCA..AUGUCGGCAUUCACCACA
NZ_CP012649/2151567-2151628	UACGUCGUCGACGCGAAGGC.GCGUCGCaa..............uuuAGCGAACCUGCA..AGGAUGCACGGGGAGGGA
NZ_CP007519/469028-469089	UACGUCGUCGACGCGAAGGC.GCGUCGCaa..............uuuAGCGAACCUGCA..AGGAUGCACGGGGAGGGA
NZ_CP007003/1534138-1534199	UACGUCGUCGACGCGAAGGC.GCGUCGCaa..............uuuAGCGAACCUGCA..AGGAUGCACGGGGAGGGA
NZ_AP017896/593708-593765	GCUGGAUCCGUCAGAAGGAU.CUGACAC..................gACAUCCAAGACA..ACGCAGUCCCGUCGAAAC
NC_013203/432964-433017	UAAUACCCCUUCACAAAGGA.GUUAA--...................-CUAUGACGAAA..AAGACUUUUAUUGGAAAU
NZ_CP011546/857513-857566	GCAACAUCGACGGCGUUGAC.GUCGA--...................-UGUUCACUGCA..UGGGCGCACCUCGCGAUG
NC_012704/1439672-1439725	UCUUUGAUUUCGGCAACGUC.GCCGG--...................-UCUUGACCCCA..AUGGUGGGGACAUUGUGA
NZ_CP020468/646951-647003	AUGAUCCUACCUAAAUCGAA.UUAG---...................-GCAGGAGGUCA..AUGAUGACUCAGCUCUCC
NC_009338/4195623-4195679	UGAAGGCGCGGCGCAACGAA.GCGCAGU...................CGGCUGUCGUCG..ACGUCGACGAAUCCGAAU
NC_014814/3405303-3405359	UGAAGGCGCGGCGCAACGAA.GCGCAGU...................CGGCUGUCGUCG..ACGUCGACGAAUCCGAAU
NC_010168/1070939-1070998	UUAGACCCCUUGAUGAUGAA.AUUAUUAg................cgUCGAACUUGUCA..AUGGUGACUUUCACCUGA
NZ_CP015866/4484667-4484722	AACUAAUCAUUCGCAUAGGG.GCGAUA-...................CGGUCACCGGCA..UGUCUGCCGACACCGACU
NC_020506/1149035-1149091	AUACCGAGAACGUCAACGGC.GACGUAG...................AACAGCCGAAUA..ACGUAAUUUCGUCGGAAU
NC_014363/208494-208553	UUCGUGGCAGUUGCAGAGAU.GCAAAGCu................ucACCAAGGCAGCC..AAGAUGCUCUUUGUCUCC
NZ_CP013979/3880043-3880103	AACAUCUGAAUGUUGACGUC.AACAUUCcu...............gaUCGGAAGAGACA..ACGGUGUCCACUUUGAAA
NZ_CP006711/1347527-1347579	UCGAUCCCAAGCGCAAGGCG.GCGCU--...................--GGACACUGCU..UUGGCGCAGGUCGAAAAA
NZ_CP006715/1421925-1421977	UCGAUCCCAAGCGCAAGGCG.GCGCU--...................--GGACACUGCU..UUGGCGCAGGUCGAAAAA
NC_017218/822879-822931	UCGAUCCCAAGCGCAAGGCG.GCGCU--...................--GGACACUGCU..UUGGCGCAGGUCGAAAAA
NZ_AP012324/1382356-1382408	UCGAUCCCAAGCGCAAGGCG.GCGCU--...................--GGACACUGCU..UUGGCGCAGGUCGAAAAA

NZ_CP006712/1371253-1371305 UCGAUCCCAAGCGCAAGGCG.GCGCU--...................--GGACACUGCU..UUGGCGCAGGUCGAAAAA
NZ_CP006713/1449468-1449520 UCGAUCCCAAGCGCAAGGCG.GCGCU--...................--GGACACUGCU..UUGGCGCAGGUCGAAAAA
NZ_CP006714/1371223-1371275 UCGAUCCCAAGCGCAAGGCG.GCGCU--...................--GGACACUGCU..UUGGCGCAGGUCGAAAAA
NZ_CP006716/1420517-1420569 UCGAUCCCAAGCGCAAGGCG.GCGCU--...................--GGACACUGCU..UUGGCGCAGGUCGAAAAA
NC_020517/1476724-1476776 UCGAUCCCAAGCGCAAGGCG.GCGCU--...................--GGACACUGCU..UUGGCGCAGGUCGAAAAA
NZ_CP009246/1947958-1948017 ACGUCUUGAGCCUCAAAGGA.GGGGGGCu................caGAGCUGGAGACA..ACGAUGUCCUCUCCCAGC
NC_013929/9274780-9274829 AGCAGUCCCUCGACAAAGAC.GUCGU--...................-----GCAGACC..AGGACGUCCCCGGCGCAC
NZ_CP012150/5969296-5969358 AUCAACGACAUCGCAACGCU.GCGAACGauu.............uucGGUGAUCGGGCG..GCGGUGCUCGCCGAGACG
NZ_CP011489/1614691-1614744 UCACUUCACGUAUCAAUGUU.GAUGU--....................-GAUUGAUGGCA..AAGCUGCUGCCGAGUUGG

NC_016782/540599-540658 AAUGCGGUGUCGCCAAAGCU.GGUGAAAg................cuCGAUAUUGGGCA..GUGGUGCCUUUUCUUGUU
NC_016800/562111-562170 AAUGCGGUGUCGCCAAAGCU.GGUGAAAg................cuCGAUAUUGGGCA..GUGGUGCCUUUUCUUGUU
NC_016786/540570-540629 AAUGCGGUGUCGCCAAAGCU.GGUGAAAg................cuCGAUAUUGGGCA..GUGGUGCCUUUUCUUGUU
NC_016802/562291-562350 AAUGCGGUGUCGCCAAAGCU.GGUGAAAg................cuCGAUAUUGGGCA..GUGGUGCCUUUUCUUGUU
NC_016783/544750-544809 AAUGCGGUGUCGCCAAAGCU.GGUGAAAg................cuCGAUAUUGGGCA..GUGGUGCCUUUUCUUGUU
NZ_CP018331/2272880-2272939 AAUGCGGUGUCGCCAAAGCU.GGUGAAAg................cuCGAUAUUGGGCA..GUGGUGCCUUUUCUUGUU
NC_013441/1890247-1890303 ACCGAACUGUCCGGAGCGAU.CCGAUGA...................GAUUCCAGGUCA..ACGGCGACUCGCUCGAUG
NC_018266/6412928-6412984 UGCCAGGUGACGACAGUGUU.GUCGAUG...................AUGAAGUCGCCG..AUGUUGGCGAGCGCGAUG
NC_017186/6403001-6403057 UGCCAGGUGACGACAGUGUU.GUCGAUG...................AUGAAGUCGCCG..AUGUUGGCGAGCGCGAUG
NC_010168/1005759-1005815 CCAAUUCCGGCUUCAACGUU.GGAGUCA...................GCAGCGUUGGCG..AAGACGCUCAAAAAGCAC
NC_014830/22856-22911 ACAGUCAUCUAUGCAUGGGU.GCAUAG-...................UCAGAUCAUGCA..CUGGUGCAUAGGGCGCCA
NC_009338/3798354-3798413 AAUACUUCAACGAUGGCGAC.AUCGUCGa................agGGACCAUCGUCA..AGGUUGACCGCGACGAAG
NC_014814/3014684-3014743 AAUACUUCAACGAUGGCGAC.AUCGUCGa................agGGACCAUCGUCA..AGGUUGACCGCGACGAAG
NZ_CP011773/572894-572950 GACCGCUGAUUGGCAACGGC.GCCAACG...................GUAAACCGGGCA..CCGGGGCUGACGGUGAAA
NC_013714/1524937-1524990 AGAACCUGAAUCGCAAUGGG.GCGGU--...................-UCGUGAAUGCA..CGGACGCACGAUAGCGUC
NZ_AP012326/1524940-1524993 AGAACCUGAAUCGCAAUGGG.GCGGU--...................-UCGUGAAUGCA..CGGACGCACGAUAGCGUC
NZ_CP010411/1838489-1838541 ACAAUCUGUUUCACAAAGGA.GUGU---...................-GCACUAUGGCU..UUGAAGCCUCGCACUGGU
NZ_CP015208/226162-226228 ACAAUAGUAUCUAGAGCGCU.CUAGAUUgugaa.........ccaccACCAAAAUUACG..GAGAAGUAAUGGCCGAUA
NZ_AP017975/3509602-3509660 AUUCAAUCCGCAGGGAUGAU.CCUGCAGu.................uUAGGUCCGAUCA..AUGACGAUUCCCUCUGGG
NZ_CP011883/408604-408663 CGACGUCAGUCCAGAUGGUU.CUGGUUGa................agGCGAAAACGGCA..ACGGCGCCACCAUAGAUG
NC_008268/2609128-2609176 CAAAAACGCAAU-CAACGUU.GCAUA--...................-----GAGUGCA..ACGUUGCACUUCACGGCA
NC_017834/573353-573407 AUGUACUCAUCGGCAUAGGU.GUCGAG-...................-CGCCCGUUGCA..AUCGUGCAUCGAGACGCG
NZ_CP015407/148239-148293 AUGUACUCAUCGGCAUAGGU.GUCGAG-...................-CGCCCGUUGCA..AUCGUGCAUCGAGACGCG
NZ_CP012649/883231-883287 AUGGUGGCCUCGGCAAGGAC.GCCGCUG...................AGGUAGCUGCCG..AUGGAGGCGAGAGGGUCG
NZ_CP007003/232466-232522 AUGGUGGCCUCGGCAAGGAC.GCCGCUG...................AGGUAGCUGCCG..AUGGAGGCGAGAGGGUCG
NC_019673/3146208-3146270 AGAAGAUGGUCGAGAGAGUC.CUCGUGGacu.............cccUGGCAAACCGCA..ACGGUGCGGGAUUCCGUG
NZ_CP010433/357064-357121 ACCCUAGGCGGCGAACGCGU.UUGCGCCGA--...............-CGUCUAGGGCA..AUGGAGCCACUUGACAGA
NZ_CP017098/357118-357175 ACCCUAGGCGGCGAACGCGU.UUGCGCCGA--...............-CGUCUAGGGCA..AUGGAGCCACUUGACAGA
NZ_CP007755/357421-357478 ACCCUAGGCGGCGAACGCGU.UUGCGCCGA--...............-CGUCUAGGGCA..AUGGAGCCACUUGACAGA
NC_011835/356849-356906 ACCCUAGGCGGCGAACGCGU.UUGCGCCGA--...............-CGUCUAGGGCA..AUGGAGCCACUUGACAGA
NC_022523/357221-357278 ACCCUAGGCGGCGAACGCGU.UUGCGCCGA--...............-CGUCUAGGGCA..AUGGAGCCACUUGACAGA
NC_017866/357061-357118 ACCCUAGGCGGCGAACGCGU.UUGCGCCGA--...............-CGUCUAGGGCA..AUGGAGCCACUUGACAGA
NC_017214/992545-992602 ACCCUAGGCGGCGAACGCGU.UUGCGCCGA--...............-CGUCUAGGGCA..AUGGAGCCACUUGACAGA
NC_017867/357063-357120 ACCCUAGGCGGCGAACGCGU.UUGCGCCGA--...............-CGUCUAGGGCA..AUGGAGCCACUUGACAGA
NC_012814/357062-357119 ACCCUAGGCGGCGAACGCGU.UUGCGCCGA--...............-CGUCUAGGGCA..AUGGAGCCACUUGACAGA
NC_021593/356921-356978 ACCCUAGGCGGCGAACGCGU.UUGCGCCGA--...............-CGUCUAGGGCA..AUGGAGCCACUUGACAGA
NC_017216/356927-356984 ACCCUAGGCGGCGAACGCGU.UUGCGCCGA--...............-CGUCUAGGGCA..AUGGAGCCACUUGACAGA
NC_017215/357074-357131 ACCCUAGGCGGCGAACGCGU.UUGCGCCGA--...............-CGUCUAGGGCA..AUGGAGCCACUUGACAGA
NC_012815/357063-357120 ACCCUAGGCGGCGAACGCGU.UUGCGCCGA--...............-CGUCUAGGGCA..AUGGAGCCACUUGACAGA
NZ_CP007522/356896-356953 ACCCUAGGCGGCGAACGCGU.UUGCGCCGA--...............-CGUCUAGGGCA..AUGGAGCCACUUGACAGA
NZ_CP009045/356897-356954 ACCCUAGGCGGCGAACGCGU.UUGCGCCGA--...............-CGUCUAGGGCA..AUGGAGCCACUUGACAGA
NC_017217/357064-357121 ACCCUAGGCGGCGAACGCGU.UUGCGCCGA--...............-CGUCUAGGGCA..AUGGAGCCACUUGACAGA
NZ_AP012332/351111-351171 AUUAUGGUUUUUAGAAAGGA.UUAACAUug...............cgUGAAACAUUGCA..AAACAGCAAGUGCCCAUG
NC_014644/490215-490275 AUUAUGGUUUUUAGAAAGGA.UUAACAUug...............cgUGAAACAUUGCA..AAACAGCAAGUGCCCAUG
NC_017456/1396431-1396491 AUUAUGGUUUUUAGAAAGGA.UUAACAUug...............cgUGAAACAUUGCA..AAACAGCAAGUGCCCAUG
NC_013174/2592010-2592060 AGAACUUCAAUGUCAAUGAC.GACAC--...................----CGUUCCCA..AUGACGGGUGUGACUCCU
NZ_CP022038/1246405-1246455 AGAACUUCAAUGUCAAUGAC.GACAC--...................----CGUUCCCA..AUGACGGGUGUGACUCCU
NZ_CP007443/542863-542924 GCCAUCAUUUUGUCGAUGAU.GAUUUGGuc..............aauAAGGAGAAGACA..AUGAAGUUCAAAACCAUU
NZ_AP012322/439087-439148 GCCAUCAUUUUGUCGAUGAU.GAUUUGGuc..............aauAAGGAGAAGACA..AUGAAGUUCAAAACCAUU
NZ_CP014241/951500-951561 GCCAUCAUUUUGUCGAUGAU.GAUUUGGuc..............aauAAGGAGAAGACA..AUGAAGUUCAAAACCAUU
NZ_AP012325/500562-500623 GCCAUCAUUUUGUCGAUGAU.GAUUUGGuc..............aauAAGGAGAAGACA..AUGAAGUUCAAAACCAUU
NZ_AP012327/557790-557851 GCCAUCAUUUUGUCGAUGAU.GAUUUGGuc..............aauAAGGAGAAGACA..AUGAAGUUCAAAACCAUU
NZ_AP012330/582181-582242 GCCAUCAUUUUGUCGAUGAU.GAUUUGGuc..............aauAAGGAGAAGACA..AUGAAGUUCAAAACCAUU
NZ_CP010413/1051877-1051923 UAAUUCUCUCCCACAGUGUU.GUUGU--...................--------CGCA..UUGUAGCGAUGAAAGAAC
NZ_CP019596/1120281-1120327 UAAUUCUCUCCCACAGUGUU.GUUGU--...................--------CGCA..UUGUAGCGAUGAAAGAAC
NC_020517/1198160-1198206 UAAUUCUCUCCCACAGUGUU.GUUGU--...................--------CGCA..UUGUAGCGAUGAAAGAAC
NZ_LT900217/820735-820786 CAACGCGGUGAGAUAAUGCC.AUCCA--...................---ACGCAGGCA..AUGGAGCCUGAAAAUAUA
NZ_CP016353/2729216-2729267 UUAAUUACUUUGACAAUGCU.GUCAA--...................---AGUUACGAA..GGGAGUCGACCGUGGAGU
NC_009077/3939026-3939077 ACCACUUUACAUACAAUGUU.GUAUC--...................---UGAGAUACA..UGAGUGUAUAAAGUGAGU
NC_015953/1565172-1565240 GCGAAAUACAACGCAACGUU.GCGUUGCauuuug.......augcuaGGGGGCUGUGCC..AGGAUGCACAACGCCACG
NZ_CP015098/5995780-5995834 GAUCCGUUGUUUCCAACGAU.GGAAAA-...................-GCUACGUUGCA..UUCACGCACCUGGCAACA
NC_015656/3985335-3985397 CUGAUGUCACUGACAUUGAU.GUCACUGaca.............cugAUGUCGCUAACA..UGGAUGUUAGUCUUCGUG

```
NC_022116/9478370-9478426      AACGCGUCCUCGACAAGGGC.GUCGUCA...................UCGCCGGUGACA..UCGGCGUCAGUGUCGUCG
NC_017186/9468514-9468570      AACGCGUCCUCGACAAGGGC.GUCGUCA...................UCGCCGGUGACA..UCGGCGUCAGUGUCGUCG
NC_018266/9478426-9478482      AACGCGUCCUCGACAAGGGC.GUCGUCA...................UCGCCGGUGACA..UCGGCGUCAGUGUCGUCG
NC_014318/9468454-9468510      AACGCGUCCUCGACAAGGGC.GUCGUCA...................UCGCCGGUGACA..UCGGCGUCAGUGUCGUCG
NC_017834/1890201-1890249      UUGCGUAGCAUGACAGCGUA.GUCAC--...................------AUUGCA..ACGACGCAUGCCGAAAGG
NZ_CP015407/765693-765741      UUGCGUAGCAUGACAGCGUA.GUCAC--...................------AUUGCA..ACGACGCAUGCCGAAAGG
NZ_CP015407/894076-894146      GUAGUUCUUGAUAAAACGUU.UUAUCACcaacugg.....cgcguauGCGUCGCAAUCA..AAGAGGAUUGAAGGAAGG
NZ_CP017717/4124855-4124911    AGCUGCUCAUUGACAAGGGC.GUCAGCG...................GCGUACCGGUCC..UGGACGACGACAAUCACG
NZ_CP013129/3448808-3448862    GACAUCUGAUCCACGGGGGA.GUGGUU-...................-CCGCAUGCCCA..ACGGAGGGUUCUGCAAGC
NZ_CP013747/2996282-2996339    AACAUGAAUGUUAGACUGGC.CUAACAA..................aAGUGUUUGGUCA..AUGGAGAUGAAAUGGACG

NC_015738/2989223-2989276      UCGCCCUCAAGAACAACGAC.GUUGU--...................-CAAUGUGGGCG..AUGUCGCCAUUCGAGUGA
NZ_CP018002/722867-722929      ACGAUUUCAGAUGCCGAGGA.GCACCUGuga.............aucACAUUGUCGGCA..ACGUGGCCCGCAAGCACG
NZ_CP010341/852125-852187      ACGAUUUCAGAUGCCGAGGA.GCACCUGuga.............aucACAUUGUCGGCA..ACGUGGCCCGCAAGCACG
NC_014215/876694-876756        ACGAUUUCAGAUGCCGAGGA.GCACCUGuga.............aucACAUUGUCGGCA..ACGUGGCCCGCAAGCACG
NZ_LN997841/755184-755246      ACGAUUUCAGAUGCCGAGGA.GCACCUGuga.............aucACAUUGUCGGCA..ACGUGGCCCGCAAGCACG
NZ_CP014196/2275221-2275286    CCAUCAAGUACUUCAACGAC.GGAGACCucgu..........cgaagGUACUGUCGUCA..AGGUCGACCGCGACGAAG
NZ_CP017421/1988841-1988906    CCAUCAAGUACUUCAACGAC.GGAGACCucgu..........cgaagGUACUGUCGUCA..AGGUCGACCGCGACGAAG
NC_017308/1945271-1945324      AGAACAGCUUUAUCGACGUC.GAUGA--...................-GCGCUCAGGCA..AGGCAGCUUCCGAUCAUG
NC_017945/2028099-2028152      AGAACAGCUUUAUCGACGUC.GAUGA--...................-GCGCUCAGGCA..AGGCAGCUUCCGAUCAUG
NC_017462/1998436-1998489      AGAACAGCUUUAUCGACGUC.GAUGA--...................-GCGCUCAGGCA..AGGCAGCUUCCGAUCAUG
NC_016781/1998501-1998554      AGAACAGCUUUAUCGACGUC.GAUGA--...................-GCGCUCAGGCA..AGGCAGCUUCCGAUCAUG
NC_016932/1969760-1969813      AGAACAGCUUUAUCGACGUC.GAUGA--...................-GCGCUCAGGCA..AGGCAGCUUCCGAUCAUG
NC_017306/1998385-1998438      AGAACAGCUUUAUCGACGUC.GAUGA--...................-GCGCUCAGGCA..AGGCAGCUUCCGAUCAUG
NC_017301/1988938-1988991      AGAACAGCUUUAUCGACGUC.GAUGA--...................-GCGCUCAGGCA..AGGCAGCUUCCGAUCAUG
NC_017307/2026638-2026691      AGAACAGCUUUAUCGACGUC.GAUGA--...................-GCGCUCAGGCA..AGGCAGCUUCCGAUCAUG
NC_018019/2024914-2024967      AGAACAGCUUUAUCGACGUC.GAUGA--...................-GCGCUCAGGCA..AGGCAGCUUCCGAUCAUG
NC_014329/1998568-1998621      AGAACAGCUUUAUCGACGUC.GAUGA--...................-GCGCUCAGGCA..AGGCAGCUUCCGAUCAUG
NC_017303/1998109-1998162      AGAACAGCUUUAUCGACGUC.GAUGA--...................-GCGCUCAGGCA..AGGCAGCUUCCGAUCAUG
NC_017031/1998340-1998393      AGAACAGCUUUAUCGACGUC.GAUGA--...................-GCGCUCAGGCA..AGGCAGCUUCCGAUCAUG
NC_017305/1995966-1996019      AGAACAGCUUUAUCGACGUC.GAUGA--...................-GCGCUCAGGCA..AGGCAGCUUCCGAUCAUG
NZ_CP010889/1998442-1998495    AGAACAGCUUUAUCGACGUC.GAUGA--...................-GCGCUCAGGCA..AGGCAGCUUCCGAUCAUG
NZ_CP012022/2021693-2021746    AGAACAGCUUUAUCGACGUC.GAUGA--...................-GCGCUCAGGCA..AGGCAGCUUCCGAUCAUG
NZ_CP010795/854778-854831      AGAACAGCUUUAUCGACGUC.GAUGA--...................-GCGCUCAGGCA..AGGCAGCUUCCGAUCAUG
NZ_CP015183/2060899-2060952    AGAACAGCUUUAUCGACGUC.GAUGA--...................-GCGCUCAGGCA..AGGCAGCUUCCGAUCAUG
NZ_CP015184/2060913-2060966    AGAACAGCUUUAUCGACGUC.GAUGA--...................-GCGCUCAGGCA..AGGCAGCUUCCGAUCAUG
NZ_CP015192/2060808-2060861    AGAACAGCUUUAUCGACGUC.GAUGA--...................-GCGCUCAGGCA..AGGCAGCUUCCGAUCAUG
NZ_CP015185/2060864-2060917    AGAACAGCUUUAUCGACGUC.GAUGA--...................-GCGCUCAGGCA..AGGCAGCUUCCGAUCAUG
NZ_CP015186/2060782-2060835    AGAACAGCUUUAUCGACGUC.GAUGA--...................-GCGCUCAGGCA..AGGCAGCUUCCGAUCAUG
NZ_CP015187/2060890-2060943    AGAACAGCUUUAUCGACGUC.GAUGA--...................-GCGCUCAGGCA..AGGCAGCUUCCGAUCAUG
NZ_CP015188/2060955-2061008    AGAACAGCUUUAUCGACGUC.GAUGA--...................-GCGCUCAGGCA..AGGCAGCUUCCGAUCAUG
NZ_CP015189/2022444-2022497    AGAACAGCUUUAUCGACGUC.GAUGA--...................-GCGCUCAGGCA..AGGCAGCUUCCGAUCAUG
NZ_CP015190/2023915-2023968    AGAACAGCUUUAUCGACGUC.GAUGA--...................-GCGCUCAGGCA..AGGCAGCUUCCGAUCAUG
NZ_CP015191/2060676-2060729    AGAACAGCUUUAUCGACGUC.GAUGA--...................-GCGCUCAGGCA..AGGCAGCUUCCGAUCAUG
NZ_CP008922/214647-214700      AGAACAGCUUUAUCGACGUC.GAUGA--...................-GCGCUCAGGCA..AGGCAGCUUCCGAUCAUG
NZ_CP021251/854178-854231      AGAACAGCUUUAUCGACGUC.GAUGA--...................-GCGCUCAGGCA..AGGCAGCUUCCGAUCAUG
NZ_CP008923/1381638-1381691    AGAACAGCUUUAUCGACGUC.GAUGA--...................-GCGCUCAGGCA..AGGCAGCUUCCGAUCAUG
NZ_CP012136/2026360-2026413    AGAACAGCUUUAUCGACGUC.GAUGA--...................-GCGCUCAGGCA..AGGCAGCUUCCGAUCAUG
NZ_CP014341/853653-853706      AGAACAGCUUUAUCGACGUC.GAUGA--...................-GCGCUCAGGCA..AGGCAGCUUCCGAUCAUG
NZ_CP013699/853655-853708      AGAACAGCUUUAUCGACGUC.GAUGA--...................-GCGCUCAGGCA..AGGCAGCUUCCGAUCAUG
NZ_CP008924/1018281-1018334    AGAACAGCUUUAUCGACGUC.GAUGA--...................-GCGCUCAGGCA..AGGCAGCUUCCGAUCAUG
NZ_CP017384/2027288-2027341    AGAACAGCUUUAUCGACGUC.GAUGA--...................-GCGCUCAGGCA..AGGCAGCUUCCGAUCAUG
NZ_CP013260/2131290-2131343    AGAACAGCUUUAUCGACGUC.GAUGA--...................-GCGCUCAGGCA..AGGCAGCUUCCGAUCAUG
NZ_CP013261/2027869-2027922    AGAACAGCUUUAUCGACGUC.GAUGA--...................-GCGCUCAGGCA..AGGCAGCUUCCGAUCAUG
NZ_CP016829/971269-971322      AGAACAGCUUUAUCGACGUC.GAUGA--...................-GCGCUCAGGCA..AGGCAGCUUCCGAUCAUG
NZ_CP023395/2027958-2028011    AGAACAGCUUUAUCGACGUC.GAUGA--...................-GCGCUCAGGCA..AGGCAGCUUCCGAUCAUG
NZ_CP013262/2026744-2026797    AGAACAGCUUUAUCGACGUC.GAUGA--...................-GCGCUCAGGCA..AGGCAGCUUCCGAUCAUG
NZ_CP021982/2025938-2025991    AGAACAGCUUUAUCGACGUC.GAUGA--...................-GCGCUCAGGCA..AGGCAGCUUCCGAUCAUG
NZ_CP013263/971189-971242      AGAACAGCUUUAUCGACGUC.GAUGA--...................-GCGCUCAGGCA..AGGCAGCUUCCGAUCAUG
NZ_CP017711/854132-854185      AGAACAGCUUUAUCGACGUC.GAUGA--...................-GCGCUCAGGCA..AGGCAGCUUCCGAUCAUG
NZ_CP013697/854151-854204      AGAACAGCUUUAUCGACGUC.GAUGA--...................-GCGCUCAGGCA..AGGCAGCUUCCGAUCAUG
NZ_CP016826/854176-854229      AGAACAGCUUUAUCGACGUC.GAUGA--...................-GCGCUCAGGCA..AGGCAGCUUCCGAUCAUG
NZ_CP017291/2026292-2026345    AGAACAGCUUUAUCGACGUC.GAUGA--...................-GCGCUCAGGCA..AGGCAGCUUCCGAUCAUG
NZ_CP017292/2026049-2026102    AGAACAGCUUUAUCGACGUC.GAUGA--...................-GCGCUCAGGCA..AGGCAGCUUCCGAUCAUG
NZ_CP014543/854160-854213      AGAACAGCUUUAUCGACGUC.GAUGA--...................-GCGCUCAGGCA..AGGCAGCUUCCGAUCAUG
NZ_CP013146/854414-854467      AGAACAGCUUUAUCGACGUC.GAUGA--...................-GCGCUCAGGCA..AGGCAGCUUCCGAUCAUG
NZ_CP013327/1998430-1998483    AGAACAGCUUUAUCGACGUC.GAUGA--...................-GCGCUCAGGCA..AGGCAGCUUCCGAUCAUG
NZ_CP019587/1998550-1998603    AGAACAGCUUUAUCGACGUC.GAUGA--...................-GCGCUCAGGCA..AGGCAGCUUCCGAUCAUG
NZ_CP013698/854144-854197      AGAACAGCUUUAUCGACGUC.GAUGA--...................-GCGCUCAGGCA..AGGCAGCUUCCGAUCAUG
NZ_CP012695/854111-854164      AGAACAGCUUUAUCGACGUC.GAUGA--...................-GCGCUCAGGCA..AGGCAGCUUCCGAUCAUG
NC_018681/3190039-3190100      GUCAAACCAUCCACAACGAC.GUGGAACug..............cguGAGGCCCUGUCC..AAGGCGAUCGUCGACCCG
NZ_CP010797/3887305-3887364    AACGAUGCCCCGACCAAGGC.GUCGGCCa................agCAGACCAACGCA..AUGUCGCGGAUGUUGACC
```

NZ_CP009755/27231-27283	UAGGAAAGAGGUUCAAGGAC.GAACC--...................--GCGUGAUGCA..AGGGAGCACUCCGUGAGC
NC_008618/534675-534736	GCCAUCAUUUUGUCGAUGAU.GAUUUGGuc..............aauAAGGAGAAGACA..AUGAAGUUCAGAACCAUU
NZ_CP010413/681548-681600	UCGAUCCCAAGCGCAAGGCG.GCGUU--...................--GGACACUGCU..UUGGCGCAGGUCGAAAAA
NZ_CP019596/1476654-1476706	UCGAUCCCAAGCGCAAGGCG.GCGUU--...................--GGACACUGCU..UUGGCGCAGGUCGAAAAA
NZ_CP008885/815425-815493	AAAGUUACACCCCCGCUGGC.GGGGCUGucggcg.......uaacggCGUUGUUUGGCA..UAGGUGCCCCUUCCCGAG
NZ_LN824140/278919-278987	AAAGUUACACCCCCGCUGGC.GGGGCUGucggcg.......uaacggCGUUGUUUGGCA..UAGGUGCCCCUUCCCGAG
NZ_CP010411/1702033-1702086	AAAGCAGCAAUA-UAAUGAA.GAUAGC-...................-GAUUUCGUGCA..AUGAUGCACGAGCCGAUC
NC_014169/814071-814139	AAAGUUACACCCCCGCUGGC.GGGGCUGucggcg.......uaacggCGUUGUUUGGCA..UAGGUGCCCCUUCCCGAG
NC_022115/3652209-3652256	ACAUUCUCACGCACAGAGGU.GUGU---...................------UCGGCC..ACGACGCCGGCGUACGAA
NC_012490/3959281-3959328	ACAUUCUCACGCACAGAGGU.GUGU---...................------UCGGCC..ACGACGCCGGCGUACGAA
NZ_CP007255/3214351-3214398	ACAUUCUCACGCACAGAGGU.GUGU---...................------UCGGCC..ACGACGCCGGCGUACGAA
NZ_CP011295/3690230-3690277	ACAUUCUCACGCACAGAGGU.GUGU---...................------UCGGCC..ACGACGCCGGCGUACGAA
NZ_CP011491/3061381-3061433	GCCGAGAGCUGCGAACUGGU.UUGCC--...................--AUCCACGGCA..AUGGAGCCGGGACGCAGA
NZ_CP019229/1577267-1577319	AAGAGUCAUGCGUCACAGAU.GACGC--...................--CGAUCUGACA..AGGCUGUCAGGAACGGAG
NC_023036/4248433-4248495	GCAGUCCUGGUGGCACCGCC.GCCAGUUaca.............aggAGGAAGAAAGUA..AUGGAACUCGAUCCCAAC
NZ_CP011022/4248504-4248566	GCAGUCCUGGUGGCACCGCC.GCCAGUUaca.............aggAGGAAGAAAGUA..AUGGAACUCGAUCCCAAC
NC_012522/4334716-4334769	UCUACGACCAGGGCAAGGCU.GCCUU--...................-CUGGGACGGCA..AAGGCGUCUGGUGUCACG
NZ_AP014938/437430-437480	GCGAGGUCAUUCAGCACGGC.CUGGU--...................----AAAGGUCA..ACGGCGACAUUGAAGAAG
NZ_CP023510/761512-761562	GCGAGGUCAUUCAGCACGGC.CUGGU--...................----AAAGGUCA..ACGGCGACAUUGAAGAAG
NZ_CP015732/3306175-3306228	GACACAACAUACAAAACGUU.UUGUA--...................-AAGGACUGUCG..AUGGCGACGAAGGUAGGA
NZ_CP022436/1910516-1910566	CCGCCCACCUUGCCAACGGU.GGCA---...................---GCCUUGGCG..AUGGAGCCGAUGCCCAGG
NZ_CP019688/718849-718909	GAAAAGGAGGUGACAACGAU.GUCGAAGgg...............acUUCGCACACGCA..AGUUUGCGCCCGCGAAGA
NC_014644/1488834-1488885	AAGGUAUUCGGCAAAAAGAU.UUGUU--...................---UUGAAUUAA..UCGACUAAUCAAUCGACU
NZ_CP017674/3091177-3091233	UCCCCCAUCGGCGCAACGCU.GCGCCGG...................UCCCAUGAGGCA..AGGAGGCCACAUGACGGA
NZ_CP020715/3524655-3524708	UGAACUACACCGUCAACGCU.GACGU--...................-CUACGCCCGCU..ACGGCGCGGACUUCGACA
NC_010397/1789584-1789640	AUUGCACAAGUCGAAAAGGA.UCGGGCC...................AAGGAAGAGGCG..AUGGCGCCACAAGUAGCG
NZ_CP014955/1772773-1772829	AUUGCACAAGUCGAAAAGGA.UCGGGCC...................AAGGAAGAGGCG..AUGGCGCCACAAGUAGCG
NZ_CP009407/1774108-1774164	AUUGCACAAGUCGAAAAGGA.UCGGGCC...................AAGGAAGAGGCG..AUGGCGCCACAAGUAGCG
NZ_CP009408/1789073-1789129	AUUGCACAAGUCGAAAAGGA.UCGGGCC...................AAGGAAGAGGCG..AUGGCGCCACAAGUAGCG
NZ_CP009613/1781082-1781138	AUUGCACAAGUCGAAAAGGA.UCGGGCC...................AAGGAAGAGGCG..AUGGCGCCACAAGUAGCG
NC_017219/1483251-1483310	GGGAUUAUUACGACGAAGGU.GUCGUCGu................auGCGCCGACUGCA..UCGAAGCGUUGCGAUACG
NC_011593/1483460-1483519	GGGAUUAUUACGACGAAGGU.GUCGUCGu................auGCGCCGACUGCA..UCGAAGCGUUGCGAUACG
NZ_CP008802/1434519-1434577	AAGGUAUAAACUCAAAUUAU.UGAGGUGa.................cAAACUUCGCGCA..AGGGUGCGGGGAAAGGGG
NZ_CP017812/2085428-2085482	CAUUGCUAAUACGUACCGUU.GUGCAA-...................-UUUCGAGGGCA..AUGACGCCAAAUGAAAAU
NZ_CP017674/2563384-2563433	UUCGUCCCCAUGACAACGAU.GUCGG--...................-----AGAUGCG..AUGACGCACGACGAGCGA
NZ_CP022436/4224508-4224564	AAUCAGCAAUCCUCACCGAC.GAGGAUG...................CGGCCCCGGGCG..AAGAGGCUUGGGGCUGAU
NZ_CP007443/217067-217115	AUAAUCAAAAUUACAACGUU.GCAAA--...................------UGAGCA..GGGAGGCUCAAUCGAAAA
NZ_CP010437/227134-227182	AUAAUCAAAAUUACAACGUU.GCAAA--...................------UGAGCA..GGGAGGCUCAAUCGAAAA
NZ_CP014513/210404-210467	AGCAUCCCCACGACAAGGAA.GUCGCUCcgg............cuucCUCUACUCACCA..AUGGAGGUCGAGUUGUCC
NZ_CP008889/2422838-2422894	UGAACGUGUUCAACAGCGUG.GUUGACU...................GAGCGCAUGACC..ACGAAGUCGGAGCAGACG
NZ_CP012479/386793-386851	AAAACCUCAUUUUCAAAGCA.GGAAUAGg.................gUCUUUGCGUGCG..ACGGUGCACGCCCCUCCC
NZ_CP015732/1748515-1748566	UUCCAGCUAAUCCCAUUGAA.GGAGC--...................---UCGACAGCA..AUGACGCUUACAGCAAAU
NZ_CP016793/824349-824401	UCGCGAUGAGCGUCAACGCC.GGCGU--...................--GGACAUGGCG..AUGGAGCCGUCGAACGCG
NC_017219/683926-683987	ACCAACCCGUAUUCAAGGAG.GAAUAAUga..............gucGAUUCAAUGGCA..AGCGCGCCGAGUUCGAGG
NC_011593/684134-684195	ACCAACCCGUAUUCAAGGAG.GAAUAAUga..............gucGAUUCAAUGGCA..AGCGCGCCGAGUUCGAGG
NZ_CP010411/1434113-1434174	ACCAACCCGUAUUCAAGGAG.GAAUAAUga..............gucGAUUCAAUGGCA..AGCGCGCCGAGUUCGAGG
NZ_CP018783/3814213-3814266	AACGUGGAUCCUGCGACGAC.GCAGA--...................-CCGAGAGGACA..ACGAUGUUCAACCCAACC
NC_018581/5108419-5108476	AAACAGACGUUUCCAGAUUC.GGGAGGG..................gUGCUCGAUGGCA..ACGGAGCCUAUCACCAGU
NC_018027/2664513-2664572	AAUACUUCAACGAUGGCGAC.AUCGUCGa................agGGACCAUCGUCA..AGGUCGACCGCGACGAAG
NC_009077/3157033-3157092	AAUACUUCAACGAUGGCGAC.AUCGUCGa................agGGACCAUCGUCA..AGGUCGACCGCGACGAAG
NC_008705/3194981-3195040	AAUACUUCAACGAUGGCGAC.AUCGUCGa................agGGACCAUCGUCA..AGGUCGACCGCGACGAAG
NC_008146/3177093-3177152	AAUACUUCAACGAUGGCGAC.AUCGUCGa................agGGACCAUCGUCA..AGGUCGACCGCGACGAAG
NC_017093/7097475-7097523	AUCAACUCGCCUGCAACGGG.GCAG---...................-----GCCUGCA..ACGGGGUAGGGAAGGAGA
NC_014550/3030140-3030192	AACAGCUACGCCUGAAAGUG.CGUG---...................-GCGUAAACGCA..AAGACGCGUGGGAACACU
NC_021200/2321644-2321703	UAGAGCCAGGCGGCACAGGU.GCCGUCGg................ccGAGGUAAAAGUG..AUGUCGCUUCGAGUCAUG
NC_002944/1887559-1887618	UAGAGCCAGGCGGCACAGGU.GCCGUCGg................ccGAGGUAAAAGUG..AUGUCGCUUCGAGUCAUG
NZ_CP010113/2295420-2295479	UAGAGCCAGGCGGCACAGGU.GCCGUCGg................ccGAGGUAAAAGUG..AUGUCGCUUCGAGUCAUG
NZ_CP010114/2298051-2298110	UAGAGCCAGGCGGCACAGGU.GCCGUCGg................ccGAGGUAAAAGUG..AUGUCGCUUCGAGUCAUG
NZ_CP015495/1887559-1887618	UAGAGCCAGGCGGCACAGGU.GCCGUCGg................ccGAGGUAAAAGUG..AUGUCGCUUCGAGUCAUG
NZ_AP017900/4227909-4227960	AGCGAUUCAGCCGCAACGGU.GCGGC--...................---ACGUGCGAA..GGGAAUCGGCAUCGUGAG
NZ_CP017839/4746123-4746174	AGCGAUUCAGCCGCAACGGU.GCGGC--...................---ACGUGCGAA..GGGAAUCGGCAUCGUGAG
NZ_CP019606/3143239-3143292	AUUCGGUUGACGGGAACGUU.CCCGA--...................-UUUUGGAGGCC..ACGAUGCCGGAUCUUGCA
NZ_CP015849/5047500-5047550	ACAACUACUUGCACAGAGCU.GUGCAG-...................-----UUGCACA..ACGAUGUGCAAUGUACGU
NC_014814/3577814-3577873	ACGAGCGCAUGCUCAUCGAC.GGGGAAUu................gcAGAACACCGGCA..GUGGAGCCACAUUCGACG
NC_009077/1201711-1201770	GUCAUGUGAACAGAAAAGGC.UCUGCGCc................agUCGAUCUCGACG..UCGAUGUCACCACCGACG
NC_008705/1198153-1198212	GUCAUGUGAACAGAAAAGGC.UCUGCGCc................agUCGAUCUCGACG..UCGAUGUCACCACCGACG
NC_008146/1193844-1193903	GUCAUGUGAACAGAAAAGGC.UCUGCGCc................agUCGAUCUCGACG..UCGAUGUCACCACCGACG
NC_021191/1672608-1672658	ACUGUAUCGACCGUAAAGUC.GCGGU--...................----ACGGGGCA..AUCGGGCCUGCGGGAAUG
NZ_LT900217/2091859-2091910	CUAGCAGUCACCGCAACGGA.GUGGU--...................---UAAAUAGCA..AACGUGCUACAACCAGUU
NZ_CP018082/376014-376071	GCCAUCGUAUUGGCAAUGGU.GUCAUUG..................aUCGCUACCGGCG..GCGUGGCCUCGGCGGAAC
NZ_CP014196/4370828-4370883	AAUAAGCAAUGAGCAAUGAU.GCUCUG-...................ACCAUGCGUGGU..CCGGUUCAUGGAACCAAG

NZ_CP017421/4114296-4114351	AAUAAGCAAUGAGCAAUGAU.GCUCUG-...................ACCAUGCGUGGU..CCGGUUCAUGGAACCAAG
NZ_CP017421/304879-304935	AUCAGCAAAUCCUCACCGAC.GAGGAUG...................CGGCCCCGGGCG..AAGAGGCUUGGGGCUGUU
NZ_AP017457/521488-521549	AAAUGGAUCAAGGCAACACC.GCCUUCGug.............cucAUCAGCGCAGCA..CUGGUGCUCCUGAUGACU
NZ_AP017900/5180039-5180104	AACGAUUGGCUCGCGACGGA.GCGCUGGuagc.........aguacAUUUCGGUCGCA..ACGACGCGGCGGCCAAAG
NZ_CP017839/6052509-6052574	AACGAUUGGCUCGCGACGGA.GCGCUGGuagc.........aguacAUUUCGGUCGCA..ACGACGCGGCGGCCAAAG
NZ_AP012331/1087095-1087156	CUUGUAGUUGUUUCAGUGAU.GAAACUAgu.............uacAAAGAAGAACCA..AGGAAGGUUUUCGCAAUG
NZ_CP011402/1266680-1266733	GUGACAAGGUUGGCAAAGCU.GCCAA--...................-GAUCAAGGAAA..AGGCAUUCUAGGCUUAAC
NZ_CP011311/906581-906635	ACCAGCUCCAUCGCAAGGUU.GCGAUC-...................-AGGCAAAUAAG..AAGUUUUAUCGGACAUUG
NC_021252/7469511-7469565	AUACCUUUGCAAGCAAAGAG.GCUUUG-...................-CUUGCAAAGCA..AAAUAGCUUUCAUGCUGA
NZ_AP017900/957359-957415	AAACGGGUGUUCCCGGAGCU.GGGAAGC...................CUUUUGAUUGCA..AAGCUGCAUACGCGUAGU
NZ_CP007456/1901199-1901260	AUAUGAACAAGUACGGGGGU.GUAUCAUug.............augAUAGCGGAGUCA..AUGCUGAUUCCGACGAAC
NZ_CP017695/1636840-1636906	AAACUUUCGUUAUCAUGGCA.GAUAGUAucgca........cccauUCAUUGGCGGCA..AAGGAGCCCCAUAUGCCG
NC_003155/2358320-2358373	AUAUGGCUGUUCACAUCGCC.GUGAA--...................-UGUUCAUGGCG..AUGCGGUCCCGCGCGAAC
NZ_CP004350/2910386-2910434	AUAGAUAGUUCCAAAACGCU.UUUGA--...................------CUAACA..GAGAAGUUUGUGCAUAAU
NC_002755/2184593-2184643	ACAAAUUCCCAUGAAAAGAA.GCACU--...................----UGUGUACA..ACGAAGUAUCUUGGUAGU
NC_012522/7609996-7610064	GUAUUCAGGGUGUCAAAGGU.GAUACGGgugacc......agggcaUUCAGGGAGUCA..AGGGUGACACUGGUGACG
NZ_CP012649/1750466-1750525	AGUACUUCAACGAUGGUGAC.AUCGUUGa...............agGAACCGUAGUCA..AGGUCGACCGCGACGAGG
NZ_CP007519/65751-65810	AGUACUUCAACGAUGGUGAC.AUCGUUGa...............agGAACCGUAGUCA..AGGUCGACCGCGACGAGG
NZ_CP007003/1174420-1174479	AGUACUUCAACGAUGGUGAC.AUCGUUGa...............agGAACCGUAGUCA..AGGUCGACCGCGACGAGG
NC_021252/4847596-4847645	UAGGAGAUGCCGACAAUGGU.GUCGG--...................-----CGAGGCA..GACCAGCCCGAUGUCACG
NZ_AP017369/2337750-2337810	UUUUUCAUAGCCCAAAAUGCG.UGGGAAAgc..............acCUAGGAUAAACA..AUGGUGUUUUAACAGCAG
NZ_CP018151/3018838-3018889	UCCUUUGGGAACGCACAGAU.GCGUU--...................---UUCAACUCA..AAGAGGAGUCCCGAAUGC
NZ_CP007490/1202741-1202801	UGAAAAUUACUAGCAAAGGC.GCGAAUCgc..............gcGCCAAAUCACCA..AUGGAGGUUGGUUUGAAC
NZ_CP012649/277195-277245	CCGAAGAACAUGCCAAUGAU.GGCU--....................---GAAGAGGCC..ACGAUGCCUUCAAUGAGG
NZ_CP007519/2022040-2022090	CCGAAGAACAUGCCAAUGAU.GGCU--....................---GAAGAGGCC..ACGAUGCCUUCAAUGAGG
NZ_CP007003/82570-82620	CCGAAGAACAUGCCAAUGAU.GGCU--....................---GAAGAGGCC..ACGAUGCCUUCAAUGAGG
NZ_CP007003/1882400-1882450	CCGAAGAACAUGCCAAUGAU.GGCU--....................---GAAGAGGCC..ACGAUGCCUUCAAUGAGG
NZ_CP011489/878644-878705	CGAAUGAUUGUCACAGGGGA.GUGAUUAag.............ugaGAACCAACAGCA..AAGCCGCUAACCAUCCAA
NZ_AP017896/773606-773660	GAGCAUGGUUCCGAAACGAU.UCGGAA-...................-CAUCCGUUGCG..AAGGAGCACCACGAUGUC
NC_009342/3018350-3018403	UGAGAGUGCUCAACAAUAAU.GUUGU--...................-GCUUGCCAUCA..AAGACGGUGCCGAGGUGG
NC_021351/3044584-3044637	UGAGAGUGCUCAACAAUAAU.GUUGU--...................-GCUUGCCAUCA..AAGACGGUGCCGAGGUGG
NC_021352/3044583-3044636	UGAGAGUGCUCAACAAUAAU.GUUGU--...................-GCUUGCCAUCA..AAGACGGUGCCGAGGUGG
NZ_CP013991/2972515-2972568	UGAGAGUGCUCAACAAUAAU.GUUGU--...................-GCUUGCCAUCA..AAGACGGUGCCGAGGUGG
NZ_CP013859/175802-175856	GAGAGGCUCACACCAAUGAA.GGUGAA-...................-CAAGAGGGGCA..UCGCCGCCUUCGGCGCGA
NZ_CP017422/154033-154094	GUGAUCACUCUCUCGAAGAC.GAGAAUGcu.............gauUGUCAGCAGACA..AAGGAGUUGGAAUUGGUG
NZ_CP016771/1070090-1070146	CAAAAGCUGGUCAGAAAGUA.CUGAUAC...................UUGAAACAAAUA..AUGAGGUUGGCGGCGCAA
NZ_CP014518/1617930-1617992	AACAUGCCCGUGACAGUGAA.GUCAACGguc............cguUACAUCCGGGCA..ACUGGGCCGAAAUCGCCC
NZ_CP013747/267857-267906	AGGUGGAUGCUGGCAAUGCC.GCCGGU-...................------AUCGCA..ACGAGGCGACAGAUGGAG
NZ_CP013747/262377-262426	AGGUGGAUGCUGGCAAUGCC.GCCGGU-...................------AUCGCA..ACGAGGCGACAGAUGGAG
NZ_CP020563/1997129-1997191	AGAUCGGCUACGACGAAGAC.GUCGAGCugg...........agcUCCGUGAGGGCA..UUGAGGCCACUACCGGCC
NC_021177/2195450-2195512	AGAUCGGCUACGACGAAGAC.GUCGAGCugg...........agcUCCGUGAGGGCA..UUGAGGCCACUACCGGCC
NZ_CP020468/2104690-2104738	ACCGCCGCACUCACAAAGGA.GUGAU--...................------UCUUCC..AUGACGAAGAUCGUGAAC
NZ_AP017896/313728-313776	ACCGCCGCACUCACAAAGGA.GUGAU--...................------UCUUCC..AUGACGAAGAUCGUGAAC
NZ_CP012070/2479486-2479551	CCAUCAAGUACUUCAACGAC.GGAGACAucgu.........cgacgGUGUCAUCGUCA..AGGUCGACCGGGACGAGG
NC_014246/765457-765509	UGAAAGAAGGAUCCAAUGCU.GGAUA--...................--ACUUGUCGAA..AUGCAUCGAUAUGGAAUG
NZ_LN868939/363725-363784	GUGGCGGCAGACACAAAGGA.GUGUGCGa...............ugCUGGUGACGUCG..AUGAAGACCCUGGCGGGG
NZ_CP013747/3777116-3777164	GGCAGAAUCUCUAAAGUGAC.UUUGC--...................------AACGCA..AUGAAGCGAACGGUGAGG
NZ_AP012322/854184-854242	UGAUGGCAAGCGUCAACGAA.GAUGUCAu...............cUUAUGGAUUGCA..AGGUUGCAAUGACACGGG
NZ_CP013991/672673-672726	CCGUCGAUAUCGACAAAGUU.GUCGC--...................-GUUUGUUUGCA..CCAGAGCACAUAGCGAGC
NZ_CP007220/1991122-1991172	AGGAAGAUCUCGUCAUCGGA.GACGU--...................----AGUAGGCG..AUGAGGUCGUCGACGACA
NC_011886/357905-357964	ACCUCGUCACGAUCAACGAA.GAUCCCAc...............guACAUCACGCGCA..AGGACGCGCCCGUGAACG
NC_018101/1773540-1773605	AAAGUAUCAUCCUCGGUGAA.GAGGUACugga.........agaucUCCUCGUCGGUA..AAGCCACCGUCGGAAAGC
NC_017317/1691258-1691323	AAAGUAUCAUCCUCGGUGAA.GAGGUACugga.........agaucUCCUCGUCGGUA..AAGCCACCGUCGGAAAGC
NC_015683/1694931-1694996	AAAGUAUCAUCCUCGGUGAA.GAGGUACugga.........agaucUCCUCGUCGGUA..AAGCCACCGUCGGAAAGC
NZ_CP011913/1677923-1677988	AAAGUAUCAUCCUCGGUGAA.GAGGUACugga.........agaucUCCUCGUCGGUA..AAGCCACCGUCGGAAAGC
NZ_CP009716/1642146-1642211	AAAGUAUCAUCCUCGGUGAA.GAGGUACugga.........agaucUCCUCGUCGGUA..AAGCCACCGUCGGAAAGC
NZ_CP010818/1681236-1681301	AAAGUAUCAUCCUCGGUGAA.GAGGUACugga.........agaucUCCUCGUCGGUA..AAGCCACCGUCGGAAAGC
NZ_CP009583/1637127-1637192	AAAGUAUCAUCCUCGGUGAA.GAGGUACugga.........agaucUCCUCGUCGGUA..AAGCCACCGUCGGAAAGC
NZ_CP009500/1681546-1681611	AAAGUAUCAUCCUCGGUGAA.GAGGUACugga.........agaucUCCUCGUCGGUA..AAGCCACCGUCGGAAAGC
NZ_LT906443/661088-661153	AAAGUAUCAUCCUCGGUGAA.GAGGUACugga.........agaucUCCUCGUCGGUA..AAGCCACCGUCGGAAAGC
NZ_CP012117/2034033-2034083	ACCAAGUAUUGCGCGAUGUU.GCGCA--...................----ACAAGUCA..AGGGAGAUGAAAUGGCGA
NZ_CP016353/5375179-5375237	GUACAGGCAACCGUACUGUC.ACGGUGUg...............cUAAAUUUUGGCU..AUGGUGCCAAAAAAUGAU
NZ_CP014241/1152771-1152827	GCCAUUCCCGGAGCAAAAGC.GUUCUGC...................GCGUCGGGCGCA..UUGGCGCGUCUGCCGAUG
NZ_CP009249/723165-723214	CCAAGAAGCUGGGCAAUGAG.GCUUU--...................-----CUUGGCC..AAGGCGCCCGAUGCCGUG
NC_013204/1642246-1642309	AUUGUAGCGUGAGCAUCGAU.GCUUUUUaac...........acgaACGCAGGUUGCA..AAUUAGCAUCUUCUUGCU
NZ_LT900217/2055559-2055612	AGGAGGGUUUCUACACCGAC.GUGGU--...................-AGACAAUGGCG..AGGAGGCCCUGGCUCUGG
NC_013174/71727-71779	GCUUGACCGAACGCAAAGGC.GCGUC--...................--AUCCAGCGCA..UAGCCGCGCUGACACGAA
NZ_CP022038/1017052-1017104	GCUUGACCGAACGCAAAGGC.GCGUC--...................--AUCCAGCGCA..UAGCCGCGCUGACACGAA
NZ_CP012390/1356990-1357058	UCAUAACCAGUAACAAUGCU.GUUACGGgcguuu......uaucucCGCGAGCUCACA..GCGGCGUGAGCAAACCAG
NZ_CP008913/2171805-2171866	AAAAUAAAACGCGAAGAGUU.UUGCCGAcu.............ugaAUGUCGGGGGCA..ACGGCGCUUACUCCCUAA
NZ_CP016775/288544-288594	AAGUUAGUGAUGCCAUCGAU.GGCA---...................---GCUAAAUCA..UCGACGAUAGGGGAGAUG

NZ_CP011883/1112421-1112495	AUUAGUUUUGCGGUAAGGGC.GCUGAGGuguuagugu.aguugacacGUGUCAAGUGCA..AGGGUGCACGUUCGCAUA
NC_018720/2013775-2013830	UGUCAGCUAUGUGCAUUGAU.GCAUUA-...................CCAUGCACUGCA..AUUUUGCAUUAAAGUAAG
NZ_CP017696/1794532-1794587	UGUCAGCUAUGUGCAUUGAU.GCAUUA-...................CCAUGCACUGCA..AUUUUGCAUUAAAGUAAG
NZ_CP015732/3982498-3982554	CGUAAGCCAUGUGGAAUGAC.CUACUCG...................AUAGCACAUUCA..UUGAAGAAUAUUGGUCCG
NC_010545/657867-657914	AUCUGAAUAGCGGUAACGGU.AUCG---...................------GCGGCA..ACGGCGCCAGCGGUAGCG
NC_020230/637433-637480	AUCUGAAUAGCGGUAACGGU.AUCG---...................------GCGGCA..ACGGCGCCAGCGGUAGCG
NZ_LT906481/2346331-2346378	AUCUGAAUAGCGGUAACGGU.AUCG---...................------GCGGCA..ACGGCGCCAGCGGUAGCG
NC_014246/1880212-1880265	AAACUAUGAAAGAAAACGAU.UUCGA--...................-GGUGGUUGGCG..AAGCUGCUGACGGUGAAG
NZ_AP012327/552725-552772	AACAUGUUUGUUGCANNGCA.GCGC---...................------UACGCA..AUGAAGCGCCCAAUCGAA
NZ_AP012331/2861565-2861616	ACCGUAUAUACGGGAAUGAA.UCUGU--...................---UCGACGGCA..AUGACGUCGAGAACAAGG
NZ_CP021140/2885061-2885114	AAAGAAGCAUCGCAAAUGAA.UGCAA--...................-UGGUAGAGGCC..AUGAAGCCGUACAUCGGC
NZ_CP011541/542146-542196	ACAGAAAAAUAGACAAAGCG.GUUUA--...................----CAUAGACC..AAGCUGUCCGCUAUGGUG
NZ_CP010407/7881746-7881797	UGAAUCCCCAGCGAAACGGG.UCGUU--...................---ACAUGGACA..AAGUGGUCGCCAGUGCCG

NC_015564/932765-932832	GAUCACAUCACUGGGACGGU.CCAGAUCgccgu........gccgcaAGGAGAUGGUCA..AUGACGAUUGCGAACAAA
NZ_CP006850/7515859-7515908	CUAAUCUCUUCCUCAACGAA.GAGAA--...................-----AUCUUCA..AAGAAGAAACAUCUUCGA
NC_022198/288997-289056	AUUUGUUCCGUGCCAACAUU.GGCGAAGg................caCCCCGCUGGGCA..AGGAAGCCAAGGAAUACA
NZ_CP007699/9065496-9065546	AUGUUCCGCUCGACAUCGAU.GUCGA--...................----UCGCGUCC..UUGGAGACGCCGGGCAGG
NZ_CP011502/3448673-3448731	GAGGUUGCCAGAACAGCAAA.GUUCUUUg.................uCGUCUGAACGCA..ACGGAGCGCAUGCUCGAC
NZ_CP022054/2197898-2197951	AUGGAAUUGGCGCCGGUGCU.GGCAA--...................-UGGUGUCGGCA..AUGGCGCCGGUGCCGGAA
NC_013204/3050747-3050800	AGAAACUUGUCGGCAAAGAU.GCUAC--...................-AGUGAUAGUCA..ACCCCGACACCGGGGAGA
NZ_CP020567/6756365-6756418	CAGGAGUACUCCGCAACGCC.GCGGG--...................-CGUCAAGGGCG..AAGAAGUCGGUGAUCGGU
NC_006361/4424930-4424995	ACGAAAUGGGCGUCAACGCU.GACGAACuugc..........cgcugCCUACCGCGUCA..UCGGCGACUCCUGCGAGG
NZ_CP015606/213181-213250	GCAAUGGCAACUUCAAAGAC.GAAGACUcguaag......uauuacgAUCCAGUCGUAA..AGGGAUACAUCAAGAAUU
NZ_AP012330/1217319-1217386	UUACACCCCCUCUCAACGAU.GAGUCGGgcgaa........uaagaaAGGGAUUCAUCA..UGGAUGAUUCAUGUGAAA
NZ_CP021992/403166-403220	UGUCAUACUGUUACAACGUU.GUAAAU-...................-CACUCAGGACG..CGGAUGUCCGACCCGACG
NZ_CP021417/2207204-2207255	GUCUUGGUAGCCACAGUGAU.GUGGG--...................---UGAUGCUCA..UGGGAGAGCUAUCCGUGG
NC_013715/1021609-1021661	GAGGUAUAUUAUGAAAAGUA.UUAUUU-...................---UUCAGAGCA..UAGGUGCUUUUAUCCUCG
NZ_CP009438/2684812-2684874	GCCGAAGUCAUCGCAAGGCA.GCGACUCaua.............gaaGCCUCUUGGUCA..AGGGUGAUGGCAACCAAG
NZ_CP009124/7213751-7213814	CCGCCGCACUUUGCAACGAU.GCAGAGAuga............ugcgUCGCCUUUUGCA..ACGAUGCAAUAGCUGAGU
NC_018531/4204050-4204106	GUCAUGGAGGCGUCAAAGAU.GAUGUCA...................ACGGUGGGGUCA..GCGAUGACGGCGUCCAGG
NZ_AP014658/2064904-2064960	ACGCACUGCUCAACAACGAU.GUUCUUU...................CGAUCAAAUGCG..AAGGCGUAGUCGGCUGAA
NZ_CP013673/2087255-2087311	ACGCACUGCUCAACAACGAU.GUUCUUU...................CGAUCAAAUGCG..AAGGCGUAGUCGGCUGAA
NZ_CP018762/3112412-3112459	AUGCGAAGUAACCCAAAGGA.GGGU---...................------UCGUCC..AUGAAGACCGAAACCAGU
NC_008711/4054097-4054153	GUCAUGGAGGCGUCAAAGAU.GAUGUCA...................ACGGUGGGGUCA..GCGAUGACGGCGUCCAGG
NZ_CP014859/4943111-4943167	GAGUAGUCCGCGCCAUCGAA.GGUGAUC...................AGGUGGAUGGCA..UCGACGCCGUCGAAGGCC
NC_015953/1508345-1508400	AGGAAUUACGCAACAAUGGC.GUUGUG-...................AAAUACACGGGC..GAGGCUCCGCAGGAGGAA
NC_017093/7744823-7744888	GACAUGUCCGCCGCGAAGGU.GCGAGAUgccg..........gcggaCGGGCGACCGCA..ACGGUGCGUUCAAUACAG
NC_018581/5174799-5174857	ACAAUGCGCAUCACAGGGGU.GUGGUCGc.................gCGAAGAUCUGCG..GCGAUGCAAGGCAUGAAG
NZ_CP019304/2070139-2070197	ACAGAGCCCGUUUCAAAGGA.GAAAACCa.................aUGCCCAAAGACA..UCGGCGUCGCCGUCAUCG
NZ_CP011309/2690501-2690553	AAAUCAUGACUAACAAUGUU.GUUACU-...................CCGCGCAUGG--..AUGAG-CCUUUAAAGAAG
NZ_CP004046/2724605-2724657	AAAUCAUGACUAACAAUGUU.GUUACU-...................CCGCGCAUGG--..AUGAG-CCUUUAAAGAAG
NC_003450/2735138-2735190	AAAUCAUGACUAACAAUGUU.GUUACU-...................CCGCGCAUGG--..AUGAG-CCUUUAAAGAAG
NC_006958/2706808-2706860	AAAUCAUGACUAACAAUGUU.GUUACU-...................CCGCGCAUGG--..AUGAG-CCUUUAAAGAAG
NZ_CP022614/2681703-2681755	AAAUCAUGACUAACAAUGUU.GUUACU-...................CCGCGCAUGG--..AUGAG-CCUUUAAAGAAG
NZ_AP017557/2685521-2685573	AAAUCAUGACUAACAAUGUU.GUUACU-...................CCGCGCAUGG--..AUGAG-CCUUUAAAGAAG
NC_020519/2735137-2735189	AAAUCAUGACUAACAAUGUU.GUUACU-...................CCGCGCAUGG--..AUGAG-CCUUUAAAGAAG
NC_022040/2503351-2503403	AAAUCAUGACUAACAAUGUU.GUUACU-...................CCGCGCAUGG--..AUGAG-CCUUUAAAGAAG
NC_009342/2734129-2734181	AAAUCAUGACUAACAAUGUU.GUUACU-...................CCGCGCAUGG--..AUGAG-CCUUUAAAGAAG
NC_021351/2753837-2753889	AAAUCAUGACUAACAAUGUU.GUUACU-...................CCGCGCAUGG--..AUGAG-CCUUUAAAGAAG
NC_021352/2753836-2753888	AAAUCAUGACUAACAAUGUU.GUUACU-...................CCGCGCAUGG--..AUGAG-CCUUUAAAGAAG
NZ_CP007724/2573639-2573691	AAAUCAUGACUAACAAUGUU.GUUACU-...................CCGCGCAUGG--..AUGAG-CCUUUAAAGAAG
NZ_CP016335/2685516-2685568	AAAUCAUGACUAACAAUGUU.GUUACU-...................CCGCGCAUGG--..AUGAG-CCUUUAAAGAAG
NZ_CP007722/2604044-2604096	AAAUCAUGACUAACAAUGUU.GUUACU-...................CCGCGCAUGG--..AUGAG-CCUUUAAAGAAG
NZ_CP010451/2601300-2601352	AAAUCAUGACUAACAAUGUU.GUUACU-...................CCGCGCAUGG--..AUGAG-CCUUUAAAGAAG
NZ_CP012194/2729797-2729849	AAAUCAUGACUAACAAUGUU.GUUACU-...................CCGCGCAUGG--..AUGAG-CCUUUAAAGAAG
NZ_CP020658/28885-28937	AAAUCAUGACUAACAAUGUU.GUUACU-...................CCGCGCAUGG--..AUGAG-CCUUUAAAGAAG
NZ_CP013991/2682978-2683030	AAAUCAUGACUAACAAUGUU.GUUACU-...................CCGCGCAUGG--..AUGAG-CCUUUAAAGAAG
NZ_CP022394/2237537-2237589	AAAUCAUGACUAACAAUGUU.GUUACU-...................CCGCGCAUGG--..AUGAG-CCUUUAAAGAAG
NZ_CP018175/2705707-2705759	AAAUCAUGACUAACAAUGUU.GUUACU-...................CCGCGCAUGG--..AUGAG-CCUUUAAAGAAG
NZ_CP018175/2718462-2718514	AAAUCAUGACUAACAAUGUU.GUUACU-...................CCGCGCAUGG--..AUGAG-CCUUUAAAGAAG
NZ_CP014984/2675966-2676018	AAAUCAUGACUAACAAUGUU.GUUACU-...................CCGCGCAUGG--..AUGAG-CCUUUAAAGAAG
NZ_CP004062/2716111-2716163	AAAUCAUGACUAACAAUGUU.GUUACU-...................CCGCGCAUGG--..AUGAG-CCUUUAAAGAAG
NZ_CP018002/1543095-1543147	AUGAUUUCGGCAACAAUGGU.GUUGUG-...................-----AAAAACAugACGGAGCUCGGAGCGGAG
NZ_CP010341/1613478-1613530	AUGAUUUCGGCAACAAUGGU.GUUGUG-...................-----AAAAACAugACGGAGCUCGGAGCGGAG
NC_014215/1644617-1644669	AUGAUUUCGGCAACAAUGGU.GUUGUG-...................-----AAAAACAugACGGAGCUCGGAGCGGAG
NZ_CP012479/3422884-3422948	CUGACGACCGUGGCGAAGAU.GCCGUUGcccu...........ugccGGGGUCAUUGCA..AGGACGCAGGGCGCCGAA
NC_010816/287383-287435	ACAAUUUGUUCCACAAAGGA.GUGU---...................-GCACUAUGGCU..UUGAAGCCUCGCACUGGU
NZ_AP014658/1353107-1353159	ACAAUUUGUUCCACAAAGGA.GUGU---...................-GCACUAUGGCU..UUGAAGCCUCGCACUGGU
NC_004307/261947-261999	ACAAUUUGUUCCACAAAGGA.GUGU---...................-GCACUAUGGCU..UUGAAGCCUCGCACUGGU
NZ_CP013673/892637-892689	ACAAUUUGUUCCACAAAGGA.GUGU---...................-GCACUAUGGCU..UUGAAGCCUCGCACUGGU
NZ_CP010453/1508758-1508810	ACAAUUUGUUCCACAAAGGA.GUGU---...................-GCACUAUGGCU..UUGAAGCCUCGCACUGGU

NC_015052/1482075-1482127	ACAAUUUGUUCCACAAAGGA.GUGU---...................-GCACUAUGGCU..UUGAAGCCUCGCACUGGU
NZ_LN824140/1010982-1011034	ACAAUUUGUUCCACAAAGGA.GUGU---...................-GCACUAUGGCU..UUGAAGCCUCGCACUGGU
NZ_CP006741/1526578-1526630	ACAAUUUGUUCCACAAAGGA.GUGU---...................-GCACUAUGGCU..UUGAAGCCUCGCACUGGU
NC_015067/1456774-1456826	ACAAUUUGUUCCACAAAGGA.GUGU---...................-GCACUAUGGCU..UUGAAGCCUCGCACUGGU
NC_017221/858703-858755	ACAAUUUGUUCCACAAAGGA.GUGU---...................-GCACUAUGGCU..UUGAAGCCUCGCACUGGU
NZ_CP016019/1497218-1497270	ACAAUUUGUUCCACAAAGGA.GUGU---...................-GCACUAUGGCU..UUGAAGCCUCGCACUGGU
NZ_CP011965/1554627-1554679	ACAAUUUGUUCCACAAAGGA.GUGU---...................-GCACUAUGGCU..UUGAAGCCUCGCACUGGU
NZ_CP011964/1367183-1367235	ACAAUUUGUUCCACAAAGGA.GUGU---...................-GCACUAUGGCU..UUGAAGCCUCGCACUGGU
NZ_CP010407/3227837-3227892	GACAUCUGAUCCACGGGGGA.GUGGUU-...................CCGCUAUGCCCA..ACGGAGGGUUCUGCAAGC
NZ_AP012322/606590-606650	GAGCUUUUUGUCUCAAUGAA.GAGGAAAac...............aaAAGGAAGGUUCA..AUGAUGAACAAGACAAUC
NZ_CP010411/473578-473639	AUUGACGCCGUGGCGGAGUU.GCCGCGGcg..............ucaAGGCAACAAGCA..AGGAGGCUUCAUGCUCAA
NZ_CP011005/3142370-3142426	AAAGCCACAGCCGCCACGGA.GCGGCUG...................GGAAAUCGUACA..AAGAUGUAGGAGAGGUGG
NC_016109/778009-778069	AUCGAUGUCACGACACUGAU.GUCGCGACa...............ccUAUGCGGCGUCA..UCGAUGACGCGACACGAA
NZ_CP011491/505518-505581	UAUUGAAUUAACCCCAUGGC.GGGUUUAcgc............ugcgAGCAUUAAUUCA..ACGCUGAAUGAAAAGAGG

NC_003155/2967226-2967279	UUUAUCUCGGCGACACGGAA.GUCGU--...................-CGUCCACGACC..AGGACGUCAAUCAUCGGG
NC_017834/1206922-1206973	GAAAUGCGUGCGGCAAUGCU.GCUGC--...................---ACAUUUUCC..UUGACGAAUUGACGGAAA
NZ_CP015407/1538414-1538465	GAAAUGCGUGCGGCAAUGCU.GCUGC--...................---ACAUUUUCC..UUGACGAAUUGACGGAAA
NZ_CP016019/134683-134747	UCCGAAUCGAGCGCAGUGAU.GCGGAAGgaga...........uaaaAUGAAGAGGUCA..AAGGAGAUGUUUUCAGCU
NZ_CP016954/2604770-2604819	GUAGUGUUACUUUCACCGCU.GAAAG--...................-----GACCGCU..AUGAAGCGACUACUUAUU
NZ_CP011340/5557595-5557641	UACAAGCUUCUUAAAACGGC.UUAA---...................-------GCGCA..ACGAAGCGCAAGAAAGAA
NZ_CP013200/3870873-3870920	GUGACGGUCUUGGCAAUGUU.GCCG---...................------GUGGCG..AUGACGCCCCAACGCAGG
NC_008541/2327690-2327755	CCAUCAAGUACUUCAACGAC.GGAGACCucgu..........cgaagGUACCGUCGUCA..AGGUCGACCGCGACGAAG
NC_018531/2452695-2452760	CCAUCAAGUACUUCAACGAC.GGAGACCucgu..........cgaagGUACCGUCGUCA..AGGUCGACCGCGACGAAG
NZ_CP022436/2675478-2675543	CCAUCAAGUACUUCAACGAC.GGAGACCucgu..........cgaagGUACCGUCGUCA..AGGUCGACCGCGACGAAG
NZ_CP014513/1468876-1468942	UGAAGACGAGAUUCACCGAU.GGAUUCGgcguc.........cgcgcGCCGAUCAUGCA..AGGGGGCAUGCAGUGGAU
NZ_CP014145/2872189-2872238	AGCACCCCAUCGUCAGCGUC.GACGGU-...................------UUAACA..AUGCAGUUGAAAGCAAGG
NC_020133/3623588-3623652	AUGUGAAGGCCACCAAGGAC.GGUGCCGaccc...........gaucGAGUUCGACGCG..GUGGUGCGCAUUGACACU
NZ_HG917972/2608512-2608576	AUGUGAAGGCCACCAAGGAC.GGUGCCGaccc...........gaucGAGUUCGACGCG..GUGGUGCGCAUUGACACU
NC_010612/2745416-2745480	AUGUGAAGGCCACCAAGGAC.GGUGCCGaccc...........gaucGAGUUCGACGCG..GUGGUGCGCAUUGACACU
NC_008711/2273818-2273883	CCAUCAAGUACUUCAACGAC.GGAGACCucgu..........cgaagGUACCGUCGUCA..AGGUCGACCGCGACGAAG
NC_011886/2039538-2039603	CCAUCAAGUACUUCAACGAC.GGAGACCucgu..........cgaagGUACCGUCGUCA..AGGUCGACCGCGACGAAG
NZ_CP007456/830090-830149	AUAAAGAAAUCGCCAAAGGC.GUUGCCAg................gaAAGUCGAUGGCA..AGGUGGUUGCGGUCAAGG
NC_009077/1406291-1406347	AAAUUCCCCUCGGCGGUGCU.GCCGCCG...................AUGCCGACGGCA..GGGCCGUCCAUGGACAAG
NZ_AP012331/2977227-2977285	ACGAUCCGGCCCCCGAUGA-.GGGGCCGg................auUCGUAUAGGGCA..AUGAUGCUUACGGUAUGA
NZ_CP011309/1296793-1296849	AUACCGAGAACGUCAACGGC.GACGUAG...................AACAGCCGAAUA..ACGUCAUUUCGUCGGAAU
NZ_CP004046/1333526-1333582	AUACCGAGAACGUCAACGGC.GACGUAG...................AACAGCCGAAUA..ACGUCAUUUCGUCGGAAU
NZ_CP013200/1273631-1273692	ACACUAAUAGCAUGAAUGAU.UCUGUGGac..............cagACAUCGCUGGCA..AUGAAGCCUGAGGCUCCC
NZ_CP009220/1150457-1150513	AUACCGAGAACGUCAACGGC.GACGUAG...................AACAGCCGAAUA..ACGUCAUUUCGUCGGAAU
NC_003450/1209941-1209997	AUACCGAGAACGUCAACGGC.GACGUAG...................AACAGCCGAAUA..ACGUCAUUUCGUCGGAAU
NC_006958/1211409-1211465	AUACCGAGAACGUCAACGGC.GACGUAG...................AACAGCCGAAUA..ACGUCAUUUCGUCGGAAU
NZ_CP022614/1289628-1289684	AUACCGAGAACGUCAACGGC.GACGUAG...................AACAGCCGAAUA..ACGUCAUUUCGUCGGAAU
NZ_AP017557/1316640-1316696	AUACCGAGAACGUCAACGGC.GACGUAG...................AACAGCCGAAUA..ACGUCAUUUCGUCGGAAU
NZ_AP017369/1359688-1359744	AUACCGAGAACGUCAACGGC.GACGUAG...................AACAGCCGAAUA..ACGUCAUUUCGUCGGAAU
NC_020519/1209940-1209996	AUACCGAGAACGUCAACGGC.GACGUAG...................AACAGCCGAAUA..ACGUCAUUUCGUCGGAAU
NC_022040/1212837-1212893	AUACCGAGAACGUCAACGGC.GACGUAG...................AACAGCCGAAUA..ACGUCAUUUCGUCGGAAU
NC_009342/1352549-1352605	AUACCGAGAACGUCAACGGC.GACGUAG...................AACAGCCGAAUA..ACGUCAUUUCGUCGGAAU
NC_021351/1401709-1401765	AUACCGAGAACGUCAACGGC.GACGUAG...................AACAGCCGAAUA..ACGUCAUUUCGUCGGAAU
NC_021352/1401708-1401764	AUACCGAGAACGUCAACGGC.GACGUAG...................AACAGCCGAAUA..ACGUCAUUUCGUCGGAAU
NZ_CP007724/1259623-1259679	AUACCGAGAACGUCAACGGC.GACGUAG...................AACAGCCGAAUA..ACGUCAUUUCGUCGGAAU
NZ_CP016335/1314970-1315026	AUACCGAGAACGUCAACGGC.GACGUAG...................AACAGCCGAAUA..ACGUCAUUUCGUCGGAAU
NZ_CP007722/1290440-1290496	AUACCGAGAACGUCAACGGC.GACGUAG...................AACAGCCGAAUA..ACGUCAUUUCGUCGGAAU
NZ_CP010451/1286056-1286112	AUACCGAGAACGUCAACGGC.GACGUAG...................AACAGCCGAAUA..ACGUCAUUUCGUCGGAAU
NZ_CP012194/1355779-1355835	AUACCGAGAACGUCAACGGC.GACGUAG...................AACAGCCGAAUA..ACGUCAUUUCGUCGGAAU
NZ_CP020658/1522631-1522687	AUACCGAGAACGUCAACGGC.GACGUAG...................AACAGCCGAAUA..ACGUCAUUUCGUCGGAAU
NZ_CP013991/1310879-1310935	AUACCGAGAACGUCAACGGC.GACGUAG...................AACAGCCGAAUA..ACGUCAUUUCGUCGGAAU
NZ_CP022394/865646-865702	AUACCGAGAACGUCAACGGC.GACGUAG...................AACAGCCGAAUA..ACGUCAUUUCGUCGGAAU
NZ_CP018175/1323843-1323899	AUACCGAGAACGUCAACGGC.GACGUAG...................AACAGCCGAAUA..ACGUCAUUUCGUCGGAAU
NZ_CP014984/1289003-1289059	AUACCGAGAACGUCAACGGC.GACGUAG...................AACAGCCGAAUA..ACGUCAUUUCGUCGGAAU
NZ_CP004062/1332273-1332329	AUACCGAGAACGUCAACGGC.GACGUAG...................AACAGCCGAAUA..ACGUCAUUUCGUCGGAAU
NZ_CP011546/1596597-1596657	GUGAUAAAACACUCACUGCC.GGGUUUCgc...............cgUUGUCUGAGGCG..AGGAUGCCGGUGAAGGGG
NC_022657/7354127-7354175	CACUGACACGUGACAACGUU.GUCAC--...................------CGGACG..AUGACGUCGUUAACCGGA
NZ_AP017457/749840-749893	CUCACGGAGUUCUCAACGAG.GAGAA--...................-CCCCAGACUCA..AAGGGGAGGCGAGAGAUG
NZ_CP014145/710315-710365	AAGAUCGUGGCGGCAACGGU.GCCGU--...................----CGGCGGCC..AGGAGGCCCGCACCUGCU
NC_012490/2862037-2862098	ACGUCAAGACCAGCAAAGUG.GCUGUGGcc..............ugcAUCGCUACGGCA..UUGCUGCUCUCAGCCUGU
NZ_CP008913/533505-533558	AAUUCCCUCCUAGCAAAGGA.GCUGG--...................-GAUACCUGGAA..AAGGAUCCACAAUGGCAC
NC_014363/264364-264414	GUCAAAUUGCUGCCAACGAC.GGCGC--...................----AUGCUGCA..UGGAUGCAAGAUCGGACG
NC_018580/9187-9243	UUCGUGUUGCGAGCAAGGUC.GUUUCAG...................GACAAGACAGCA..GCGGAGCUGCCGUCGGAA
NZ_CP016076/5692023-5692090	GUGACAUCAUGUCCAACGAU.GGAUUCCgaucg........aguggaUCGAGGAUGUCG..AUGGAGACGCGACUGUUG
NZ_CP016077/5778466-5778533	GUGACAUCAUGUCCAACGAU.GGAUUCCgaucg........aguggaUCGAGGAUGUCG..AUGGAGACGCGACUGUUG
NZ_CP015732/3740542-3740604	AUGACAAGAUCGGCAAUACC.GCCGAGAaga.............ucgCGGGCAAGGGCA..AGGAAGCCGCGGGCGAGG
NC_022198/841374-841433	AGUACUUCAACGAUGGUGAC.AUCGUCGa................agGUACCGUCGUCA..AGGUUGACCACGACGAAG

NZ_CP013991/438335-438395	UCAGCGCAAACCACAAAUUU.GUGGUCCag..............gaCAUUAUUUAGAA..AAGGUUCUUCCUUAAAAU
NC_015145/1917982-1918047	CCAUCAAGUACUUCAACGAC.GGAGACCucgu.........cgaagGUACCGUCGUCA..AGGUCGACCGCGAUGAAG
NZ_CP013747/4860364-4860429	CCAUCAAGUACUUCAACGAC.GGAGACCucgu.........cgaagGUACCGUCGUCA..AGGUCGACCGCGAUGAAG
NZ_CP012479/98837-98904	AAUAUCCGGGCCCCACCGGG.GUGGCGGggcau.......aaugggAAAAGUAAAGCA..AAGGGGCUUUCGGCAUGG
NZ_AP017896/2027415-2027467	AUGAUCCUACCUAAAUCGAA.UUAG---..................-GCAGGAGGUCA..ACGAUGACUCAGCUCUCC
NZ_CP013200/4027396-4027453	UUCACAAAUCCACCAAUCAU.GGUGGAG.................uAGGACGCGGACA..AGGCCGUCAAUGGAGACA
NC_016785/1806266-1806319	ACGAAGCAAAGAAUAAAGGC.AUUGU--..................-UCAAUACGGCA..UGGGUGCUUUGAUCGGUG
NC_002935/1881442-1881495	ACGAAGCAAAGAAUAAAGGC.AUUGU--..................-UCAAUACGGCA..UGGGUGCUUUGAUCGGUG
NZ_CP020410/1259650-1259703	ACGAAGCAAAGAAUAAAGGC.AUUGU--..................-UCAAUACGGCA..UGGGUGCUUUGAUCGGUG
NC_013174/558552-558606	GCAAGGUUAUUUACAUCGAU.GUAGAA-..................-CAAGUGAAUCG..ACGACGAUUCACGACAGU
NZ_CP022038/530242-530296	GCAAGGUUAUUUACAUCGAU.GUAGAA-..................-CAAGUGAAUCG..ACGACGAUUCACGACAGU
NZ_CP011309/1195547-1195604	UUUUAUAUCCUAGCAGGGGU.GUUGCAU.................cAUGCAAUAAACA..UGGUAGUUUGUGUUCACA
NZ_CP004046/1235481-1235538	UUUUAUAUCCUAGCAGGGGU.GUUGCAU.................cAUGCAAUAAACA..UGGUAGUUUGUGUUCACA
NZ_CP022614/1188383-1188440	UUUUAUAUCCUAGCAGGGGU.GUUGCAU.................cAUGCAAUAAACA..UGGUAGUUUGUGUUCACA
NZ_AP017557/1218594-1218651	UUUUAUAUCCUAGCAGGGGU.GUUGCAU.................cAUGCAAUAAACA..UGGUAGUUUGUGUUCACA
NZ_CP016335/1216979-1217036	UUUUAUAUCCUAGCAGGGGU.GUUGCAU.................cAUGCAAUAAACA..UGGUAGUUUGUGUUCACA
NZ_CP010451/1179688-1179745	UUUUAUAUCCUAGCAGGGGU.GUUGCAU.................cAUGCAAUAAACA..UGGUAGUUUGUGUUCACA
NZ_CP012194/1257734-1257791	UUUUAUAUCCUAGCAGGGGU.GUUGCAU.................cAUGCAAUAAACA..UGGUAGUUUGUGUUCACA
NZ_CP022394/767600-767657	UUUUAUAUCCUAGCAGGGGU.GUUGCAU.................cAUGCAAUAAACA..UGGUAGUUUGUGUUCACA
NZ_CP018175/1225797-1225854	UUUUAUAUCCUAGCAGGGGU.GUUGCAU.................cAUGCAAUAAACA..UGGUAGUUUGUGUUCACA
NZ_CP014984/1187758-1187815	UUUUAUAUCCUAGCAGGGGU.GUUGCAU.................cAUGCAAUAAACA..UGGUAGUUUGUGUUCACA
NZ_CP004062/1234226-1234283	UUUUAUAUCCUAGCAGGGGU.GUUGCAU.................cAUGCAAUAAACA..UGGUAGUUUGUGUUCACA
NZ_CP021417/2530701-2530763	AUAGUUGCGCGUUCAAUGAU.GGGGAUUccg............gaaCGGGAGAAGUCA..UCGGUGACGUCGUCGAUA
NZ_CP007490/330150-330217	AAGUUUUUUAUGGCAGGAUU.GCCACAGguuac.......auacccCUAUGGAGGACA..ACGAUGUCUCAGGAAACC
NZ_CP013738/2202088-2202150	AGAUCGGCUACGACGACGAC.GUCGAGCugg............agcUCCGUGAGGGCA..UUGAGGCCACUACCGGCC
NC_010572/6048357-6048419	AGAUCGGCUACGACGACGAC.GUCGAGCugg............agcUCCGUGAGGGCA..UUGAGGCCACUACCGGCC
NZ_CP011522/6226773-6226835	AGAUCGGCUACGACGACGAC.GUCGAGCugg............agcUCCGUGAGGGCA..UUGAGGCCACUACCGGCC
NC_016887/2129175-2129230	AUGGCGCAUUCAGCGCUGAU.GCUGGC-..................UGCAUGGUGACA..ACGAAGUCAACGGCGACU
NZ_CP015604/718815-718864	GAAAAAUCUCAGGGAAAGCC.CCCUU--..................-----CGCGGCA..AAUGUGCCUCGCUUCACA
NZ_CP014869/378305-378358	AGUUUGAUGAUGCCGCCGGC.GGUGA--..................-AGGCGAUGGCA..AAGAUGCCGGCGAUGAUC
NZ_CP009244/2511408-2511464	AAAAGUUGUACCCCAAUGGC.GGGUGUC.................UUGUUAAUUUCA..UAGCCGAAAAAUUCGACU
NZ_CP019705/2597990-2598046	AAAAGUUGUACCCCAAUGGC.GGGUGUC.................UUGUUAAUUUCA..UAGCCGAAAAAUUCGACU
NZ_CP012677/473061-473110	GGAUUUGCAGCC-CACCGUC.GAGG---..................---GCAUCGGCA..AGGAUGCCUCACCAGAAG
NZ_AP012327/2152151-2152210	AAUCUUACUUUAGCAAUGGC.GCUGAAAa...............agGAGAAAACGAUG..AAGCGAUCCCGUAAGAUC
NZ_AP017369/2843690-2843740	UAGAUGAACUCGGUCAAGAU.AUCGU--..................----CAGUGGCA..AGGUUGCUGUUGGCGAAA
NZ_CP016954/1301520-1301576	UAAAUGACGUCAACGGCGGG.GUUGGCG..................AUCAUUUCGGCA..UAGGAGUCAUGGGCGUUG
NZ_LT635457/581874-581940	UUAGCAGGAGCUAAACCGAA.UUAGUUGuuaau........cugugAUCAGAGCCGCA..AAGCAGCGGUAAGGCAGA
NC_013124/1724947-1725012	GCGAUUUCUGCUACAGUGUU.GUCGUGGuguc.........aaccaACCAAGUCGGCA..ACGAAGCCUCGACCACUC
NZ_CP022088/3398943-3399004	GUCAAACCAUCCACAACGAC.GUGGAACug.............cgcGAGGCCCUGUCC..AAGGCGAUCGUCGAUCCG
NZ_CP008802/1274459-1274527	AAGAUGACUGCGGAUGCGGU.UCCGUACagcccg......aagucgUAGUAUUCGGCA..ACGGCGCCCCAAAAACCA
NZ_CP022544/1884403-1884464	UAUAACUGUUGCUCACUGCU.GAGCACGca.............uagAUAGAAGGAACA..AGGAGGUUUCCUCAUGGA
NZ_CP011530/3148046-3148105	GCCAUUGGCGAA-CAAUGCC.GCUUCCUag..............ccUCGCCACCUGCA..AAGGAGCAGAAUCCGAUG
NC_010168/1611182-1611245	CAAGCUAUGCCGGCAACGAU.GCCAAUUcgc...........aaggAGACUCAACGCC..AUGGUGCGCGAACUUUCA
NZ_CP011913/330052-330103	AAUAGGCCGUCU-GAAAGAU.CUAG---..................-CGGAUCGAUCA..UCGGCGAUUUUUGUCAAU
NC_019966/21589-21645	AGUGCGCUGCCUGCAAUGGU.GCAGGUG..................ACGCGGUUGGCC..ACGGAGCCGGGAAGCCGG
NC_008618/1278132-1278189	ACGCGCUUAAUCCCAAUGUU.GGGGUGC.................gCGUGGAACAUCA..AGGAAGAUAAGUGAAGGA
NZ_AP012322/565546-565592	GUAACUAUAUUCUCAAUAAG.GAGAU--..................--------UGCA..AUGUCGCAUAAGAAUAAG
NZ_CP014241/1122454-1122500	GUAACUAUAUUCUCAAUAAG.GAGAU--..................--------UGCA..AUGUCGCAUAAGAAUAAG
NZ_CP014196/3630293-3630349	AAUCAGCAAUCCUCACCGAC.GAGGAUG..................CGGCCCCGGGCG..AAGAGGCUUGGGGCUGUU
NZ_CP022054/1717377-1717433	CUGACGCUGACGACAACGUC.GUUGACG..................CUGAGGUCGUCG..ACGAGGACGAGAAGGACA
NC_013174/144170-144235	UCAACCUCACCAUCAACGAA.GGUGACUuugu.........ugccgUCAUCGGUGGAA..AUGGGUCCGCGAAAACCA
NZ_CP022038/944597-944662	UCAACCUCACCAUCAACGAA.GGUGACUuugu.........ugccgUCAUCGGUGGAA..AUGGGUCCGCGAAAACCA
NC_010168/2957855-2957917	AAGAACAGAUCGAGGAUGAC.CUCUUCGggg............augACCGUCUCGUCA..AAGGUGACCGCAACAACU
NC_008726/2379255-2379314	ACGAGCGCAUGCUCAUCGAC.GGGGAACu...............gcAAAUCACGGGCA..GCGGAGCCACAUUCGACG
NC_013714/212515-212569	AUUAUAGUUGUGGUAACGUU.ACCGCA-..................-UUGAAAUGGUA..AAGGAGCUGAAUUAGAUG
NZ_AP012326/212517-212571	AUUAUAGUUGUGGUAACGUU.ACCGCA-..................-UUGAAAUGGUA..AAGGAGCUGAAUUAGAUG
NZ_AP017369/3237161-3237214	UGAGAGUGCUCAACAAUAAU.GUUGU--..................-GCUCGCCAUCA..AAGACGGUGCCGAGGUGG
NZ_CP016174/3249576-3249634	AACAAACUGGACGCACAGAG.GCGUGGUg................uCUACUAACCGCA..AGGCCGCGAUCCUCGGGA
NZ_CP010453/667887-667947	UAAAUUAUGGUA-UAAUGCA.AGUAUGAau.............ugcUUGGUUUGCGCA..AUGAAGCGGAGGCUCAAU
NZ_CP010411/76144-76204	UAAAUUAUGGUA-UAAUGCA.AGUAUGAau.............ugcUUGGUUUGCGCA..AUGAAGCGGAGGCUCAAU
NZ_CP006741/766087-766147	UAAAUUAUGGUA-UAAUGCA.AGUAUGAau.............ugcUUGGUUUGCGCA..AUGAAGCGGAGGCUCAAU
NC_017221/1643748-1643808	UAAAUUAUGGUA-UAAUGCA.AGUAUGAau.............ugcUUGGUUUGCGCA..AUGAAGCGGAGGCUCAAU
NZ_CP018134/2373937-2373993	GAAGAAUCCGCGGCAUCGGC.GCCGUCG..................CAUUCGUGGGCA..UCGCAGCCGUCGCGCUGG
NZ_CP011269/444224-444284	AUGAUCGCAGUGGCACUGGU.GCCACUGcg..............ccACGACUUCGGCC..UGGAUGCCGCCGAGGUGA
NZ_CP009914/514977-515037	AUGAUCGCAGUGGCACUGGU.GCCACUGcg..............ccACGACUUCGGCC..UGGAUGCCGCCGAGGUGA
NZ_CP012677/125110-125174	AUUGUCAUCAGCGCAGCGU-.GUGCGAUccac.........ccccgCCCGAAAGAACA..AGGGAGUUUUUAAUGAAG
NC_016782/1194943-1194999	AACUACGUGAGCCCACUGAU.GGGGAUU..................GGUGCAGAAGCA..UGUGAGCUACUCGUCACA
NC_016786/1194912-1194968	AACUACGUGAGCCCACUGAU.GGGGAUU..................GGUGCAGAAGCA..UGUGAGCUACUCGUCACA
NC_016788/1194357-1194413	AACUACGUGAGCCCACUGAU.GGGGAUU..................GGUGCAGAAGCA..UGUGAGCUACUCGUCACA
NC_016790/1161747-1161803	AACUACGUGAGCCCACUGAU.GGGGAUU..................GGUGCAGAAGCA..UGUGAGCUACUCGUCACA
NZ_CP010412/732893-732949	AUGAUGCGAUGCGCGAUGUG.GCGUUCG..................AUGUCAGAAACA..GCGGUGUUCCAACGUUUU

```
NC_013204/1311510-1311568       UGAAGCCCCGCG-CAUCGAU.GCCGCUAc................cgGGCUGCUUGGCA..AAGAAGCCUUCUUCGACG
NZ_CP021140/2331515-2331573     UGAAGCCCCGCG-CAUCGAU.GCCGCUAc................cgGGCUGCUUGGCA..AAGAAGCCUUCUUCGACG
NC_020990/2012045-2012095       ACGGUGGCCACGGCAAGGAC.GUCGA--...................----CAACGUCA..AGGUCGACUUCCACGGUG
NZ_CP014485/2289075-2289125     ACGGUGGCCACGGCAAGGAC.GUCGA--...................----CAACGUCA..AGGUCGACUUCCACGGUG
NZ_CP009211/817633-817686       ACGAGCUGAUGGACAACGGC.GUCGA--...................-GGUGCUUGGCA..UCGACGUCGGCGACAAGG
NZ_LT906467/899599-899652       ACGAGCUGAUGGACAACGGC.GUCGA--...................-GGUGCUUGGCA..UCGACGUCGGCGACAAGG
NZ_CP018002/2595505-2595555     GGAUCAUGGAACACACAGCU.GUGUU--...................----AUCGGGCG..AAGGCGCCAAGGCCGAGC
NZ_CP010341/2613515-2613565     GGAUCAUGGAACACACAGCU.GUGUU--...................----AUCGGGCG..AAGGCGCCAAGGCCGAGC
NC_014215/2579483-2579533       GGAUCAUGGAACACACAGCU.GUGUU--...................----AUCGGGCG..AAGGCGCCAAGGCCGAGC
NZ_LN997841/2620242-2620292     GGAUCAUGGAACACACAGCU.GUGUU--...................----AUCGGGCG..AAGGCGCCAAGGCCGAGC
NZ_AP012334/1500618-1500669     GCAGUUGUGUCAGCAACGAU.GCUGU--...................---UCAGCGGCU..GUGGUGCUGUAUCCUCAG
NC_015434/4457151-4457207       GACAUAACUCGUACAACGGC.GUACGGU...................UUAUCUCGAACA..UGAAUGUUCGAGAUGGAG
NZ_CP010827/379174-379234       AAGAUGCGCUCGGCGCUGAC.GCUGUUGgg...............cgUCAUCAUCGGCA..UUGCCGCCGUCAUCGCCA
NZ_CP011773/5271312-5271360     ACUUCAGCGACGGCAGCGGU.GCCGU--...................------CAGGCA..UCGACGCCGUUGACCCGG
NZ_CP017421/3269014-3269070     GCAAGGAGGGUUUCGACGUU.GAAGUAG...................UGGACAACGGCA..GUGAUGCCUUGGUGGAAU
NZ_CP014634/1825255-1825308     CAAAUGCUUACGGCAAAGAC.GUCGA--...................-AGCCGUCCUCA..UUGGCGAGGAAGAGCUGC

NZ_LT906453/256087-256143       UCGAAAGACAUCUCAACGAC.GAGGAGA...................AUCAGAUGAGCA..AUGUGGCUCGCCUGGACC
NZ_CP016174/7928691-7928746     UCGGUUCGUUUCUCAAAGAG.GAGAAC-...................ACGCGAGUGGCA..ACGGUGCUGAAAGAGCUG
NZ_CP012750/2883156-2883212     GCAAUGCGGUCGGCGGUGCC.GCUGAUG...................UCAUCGUUGGCA..ACGACGCCGAUCAUCGCC
NC_016516/1290317-1290367       UGAUCGUUCUGAGCAAAGGA.GUUCA--...................----CAUGAGCA..UUGCCGCUCUCAUCGAUC
NZ_CP017040/580710-580763       ACGAUGACUCCGAUAUCGAU.GUCGU--...................-CGACGUCGACA..AUUCCGUCCCCGAAAACA
RF                              AaaauaucauccaCAAuGaU.Gugguug...................auguaaaagGCA..AuGgaGCcucuaacgAag
SS_cons                         ::::::::::<<<<______.>>>>---...................--------<<<_..____>>>::::::::::
```
